# Temporal trends in serum testosterone and luteinizing hormone levels indicate an ongoing resetting of hypothalamic-pituitary-gonadal function in healthy men: a systematic review

**DOI:** 10.1007/s40618-025-02671-9

**Published:** 2025-08-01

**Authors:** Daniele Santi, Giorgia Spaggiari, Chiara Furini, Valentina Griseta, Eric A. Zizzi, Antonio R.M. Granata, Manuela Simoni

**Affiliations:** 1 Unit of Endocrinology, Department of Medical Specialties, Azienda Ospedaliero-Universitaria of Modena, Modena, 41126 Italy; 2 Unit of Andrology and Sexual Medicine of the Unit of Endocrinology, Department of Medical Specialties, Azienda Ospedaliero-Universitaria of Modena, Modena, 41126 Italy; 3 Department of Biomedical, Metabolic and Neural Sciences, University of Modena and Reggio Emilia, Modena, 41126 Italy; 4 Polito BIO Med Lab, Department of Mechanical and Aerospace Engineering, Politecnico di Torino, Corso Duca degli Abruzzi 24, Torino, 10129 Italy

**Keywords:** Testosterone, Luteinizing hormone, LH, FSH, Temporal trend

## Abstract

**Purpose:**

Male fertility is progressively impairing over time, probably related to a multifactorial genesis. The aim of the study was the evaluation if a Temporal trend in serum testosterone levels exists in healthy men.

**Methods:**

A search of the literature between 1971 and July 2024 was performed, selecting study groups in which testosterone serum levels were measured for any reason in healthy men. Exclusion criteria were: (i) age < 18 years old, (ii) conditions affecting testosterone levels, (iii) subjects’ enrolment based on testosterone serum levels and (iv) blood examinations performed in a time-frame interval > 10 years. Secondary endpoints: luteinising hormone (LH), follicle-stimulating hormone (FSH), sex hormone binding globulin (SHBG) serum levels and body mass index (BMI).

**Results:**

1,256 papers, accounting for 1,504 study groups, were selected, including 1,064,891 subjects (age 42.0 *±* 7.0 years). A significant negative linear regression between testosterone serum levels and year of measurement was detected (*p* = 0.033). The comprehensive decline in testosterone serum levels over the years was confirmed adjusting meta-regression analysis using the number of subjects included in each study, subjects’ age, BMI and the the assay used for testosterone measurement. No temporal trend was observed regarding BMI in this population. LH serum levels showed a significant decline over the years, adjusting for subjects’ age, while no trend emerged considering FSH.

**Conclusion:**

This study is the first comprehensive analysis suggesting a progressive decrease in serum testosterone and LH levels in healthy men, independent of age and BMI. The observed decline in both testosterone and LH levels could be a consequence of an ongoing resetting of the hypothalamic-pituitary-testicular function.

**Supplementary Information:**

The online version contains supplementary material available at 10.1007/s40618-025-02671-9.

## Introduction

In 1992, the first systematic review of the literature to investigate whether semen quality has changed during the past 50 years was performed [1]. Sixty-one papers published from 1930 to 1991 were considered, showing, for the first time, a decline in semen quality over the previous 50 years [1]. In 2017 and 2023, this systematic analysis of the literature has been updated [2, 3], including 288 studies. The result provides evidence of a decline in sperm counts world-wide since 1980 s with an accelerated decline in both sperm concentration and total sperm count after 2000 s [3]. These results have been repeatedly criticized, questioning whether the observed trend is merely a consequence of analytical variability of sperm parameters [4–7], rather than representing a true decline. However, the continuous decrease in sperm characteristics, coupled with the consistency observed across various analyses [8], strongly suggests that this trend could be real and of concern for human fertility. Several authors have posited that this decline could be attributed to various factors, ranging from societal changes to increasing environmental pollution [3].

Considering testicular functions as a whole, the interstitial compartment could also play a role, but very few studies investigated so far serum testosterone trends over the years in limited populations [9, 10]. Clinically, testosterone deficiency is a common condition, affecting men with increasing prevalence as they age [11–14]. Several suggestions are available in favour of an increasing incidence of testosterone deficiency in the last decades. Indeed, androgen replacement therapy is being increasingly prescribed, suggesting that clinicians recognize and diagnose testosterone deficiency more than in the past. In the United States, testosterone prescriptions increased by over 350% from 2001 to 2011 and these prescriptions among patients insured with Medicare increased by 15.5% on average each year [15]. Despite several scientific societies produced guidelines about correct testosterone prescription [16, 17], this rate is seems to be continuously increasing year after year [18, 19]. Thus, the question arises whether this increasing prescription rate is a mirror of a testosterone deficiency rise. Moreover, if the drop in sperm production is real as a sign of decline in testicular function, the question arises of what happens to the interstitial compartment.

With these questions in mind, we designed this study to evaluate the historical trend of testosterone serum levels in men enrolled in the clinical trials published so far. Finding a global testosterone decline could have broad implications both in reproductive medicine and considering the general male health decline suggested by many authors in recent years [20].

## Methods

A systematic review was performed and two datasets, i.e. MEDLINE and Embase (Excerpta Medica database), were queried up to July 10th, 2024 for peer-reviewed, English-language publications. Since the first publication reporting testosterone serum levels dated back in 1970, we included all publications between January 1th, 1970, and the date of the literature search.

Following the recommendation of the Cochrane Handbook for Systematic Reviews, we considered the index (MeSH) terms ‘testosterone’ and ‘androgen’ and we searched in titles and abstracts for both MeSH terms, keeping the widest possible literature search. The detailed search strategy is reported in the Supplementary Material (page 2). The literature search was performed aiming at identifying all articles that reported primary data on testosterone serum levels in humans.

### Studies selection

All studies that reported primary data on testosterone serum levels in healthy subjects were considered eligible for abstract screening. We evaluated the eligibility of all subgroups within a study. For example, in a case-control study, the control group was eligible for inclusion even though, based on our exclusion criteria, the case group was not. Similarly, a cohort prospective study was included if testosterone serum levels were reported at baseline, before any experimental intervention. Thus, the inclusion criteria considered were (i) healthy, eugonadal males, (ii) older than 18 years, (iii) in which testosterone serum levels were measured and reported. Otherwise, a study was excluded if study participants were selected based on testosterone serum levels (i.e. studies selecting hypogonadal men), or if enrolled men were exposed to condition that may affect testosterone production, such as occupational exposure, drug use and oncological patients, of if they were athletes or trained men. Moreover, studies were excluded if the blood examination were performed in a time-frame interval longer than 10 years.

First, based on the title and abstract, the publication was either excluded or advanced to full text screening. Any publication without an abstract was automatically referred for full text screening. Second, we reviewed the full text and assigned it to exclusion or data extraction. We then confirmed study eligibility and identified multiple publications from the same study to ensure that estimates from the same population were not used more than once.

### Data extraction

The primary endpoint was testosterone serum levels. Thus, summary statistics on testosterone measurement was extracted (mean, standard deviation (SD), standard error (SE), minimum, maximum, median, and percentiles), together with sample size, subjects’ age, and blood collection year. Testosterone serum levels were extracted as nmol/L. When reported differently, they were converted accordingly.

Secondary endpoints were luteinising hormone (LH), follicle-stimulating hormone (FSH) serum levels, sex hormone binding globulin (SHBG) and body mass index (BMI), when available. Additional confounders, such as the laboratory methodology used to measure serum testosterone levels and the country of origin of the study population, were also extracted.

The literature screening and extraction was conducted independently by two reviewers (CF and VG), with disagreements resolved through discussion within the review team members (DS, GS, ARMG, CF and VG). The dataset created was cleaned and checked for completeness by DS and GS. Any discrepancies and unreasonable values were flagged and clarifications were sought by the responsible investigators. All included studies were individually assessed for quality using a predesigned assessment form by four reviewers independently (DS, GS, CF and VG).

### Environmental data

To assess the potential influence of the environment on meta-regression analyses between serum hormone levels and the years of blood collection, we accessed available online datasets (Table 1). Specifically, we searched the Statistical Review by the Energy Institute to gather data on energy production, consumption, trade, and emissions (https://www.energyinst.org/statistical-review).

**Table 1:** Data regarding environment and population growth considered as factors possibly influencing testosterone trends

Environmental parameters	Population-growth related parameters
Total natural resource rents	Annual population growth
Primary energy consumption	Population number
Natural gas flaring	Life expectancy at birth
Total liquid oil consumption	Mortality rate (total, cardiovascular diseases, cancer, diabetes, and attributed to household and ambient air pollution)
Hydro consumption	Lower secondary school completion rate
Energy use (kilograms of oil equivalent per capita)	Number of neonatal deaths
CO2 emissions (metric tons per capita)	Logistics performance index: Ability to track and trace consignments
CO2 emissions from transport	Adolescent fertility rate
CO2 emissions from flaring and liquid sources	Mobile cellular subscriptions
Oil production and renewables consumption	Annual birth rate
PM2.5 air pollution (mean annual exposure in micrograms per cubic meter)	Fertility rate
Fossil fuel energy consumption	Contributing family workers
CO2 emissions from gaseous fuel consumption	Adjusted net national income
Nitrous oxide emissions	Pre-primary education
Electric power consumption (kilowatt-hours per capita)	Number of maternal deaths
Gas production	Births attended by skilled health staff
Coal consumption	Diabetes prevalence
Gas consumption	Prevalence of anaemia among women of reproductive age
	Adjusted net national income per capita
	Labor force participation rate
	Human capital index
	Life expectancy at birth
	Adjusted net national income per capita

*CO2: *Carbon Dioxide*; PM: *particulate matter

Additionally, to describe the potential effect of demographic changes on meta-regression analyses between hormone levels and the years of blood collection, we extracted data on societal characteristics from online datasets provided by the World Health Organization (WHO) (https://www.who.int/data/global-health-estimates) (Table 1).

The data were available online and reported annually, categorized by country. We linked these data with our dataset. Specifically, for each study included in the analysis, data from both environmental and population growth-related parameters were combined, considering the year of blood collection for testosterone serum analysis and the country where the study was conducted.

### Quality control

The literature search was conducted by a team of two reviewers (C.F. and V.G.). After the selection, other two authors performed the evaluation of inclusion and exclusion criteria in each study (D.S., and G.S.). A team of four reviewers performed the data extraction from the final pool of selected studies (C.F., V.G., D.S., and G.S.).

All data were entered into digital spreadsheets with explicit permissible values (no open-ended entries) to increase consistency. After data extraction, an additional round of data editing and quality control of all studies was conducted by D.S. The process ensured that each study was evaluated by at least two different reviewers.

### Statistical analysis

As reported above, the primary outcome of our analyses was total testosterone serum levels extracted from studies available in the literature. The data from individual studies were synthesised considering the aggregated data extracted as means and SD after conversion to the same unit of measurement (i.e. nmol/L). For each study, the number of subjects enrolled was reported, together with the years in which the blood samples were collected for hormone measurement. When the blood collection was performed in an interval of years, the midpoint of the range was considered (‘collection year’). For biochemical measurements performed on blood samples collected years earlier, we used the year of blood collection rather than the year of laboratory analysis. Moreover, aggregated data for other variables extracted were added as mean and SD for each study, when available.

Data analysis first provide a qualitative and quantitative description of data collected. Then, linear regression analyses were performed using Rho’s Spearman correlation. In this step, aggregated data were considered for each study included, combining total testosterone serum levels with other variables extracted, such as BMI, subjects’ age, LH and FSH, using Rho’s Spearman correlation. The second step of the analysis provided the evaluation of testosterone serum levels trend along years of observation. First, to test whether a trend among hormonal data collected could be evaluated, autocorrelation analyses were performed by Durbin Watson (DW) analysis. A score equal to 2 represented the absence of autocorrelation, the value between 0 and 2 a positive autocorrelation and the value comprised between 2 and 4 a negative autocorrelation. Then, meta-regression analysis was performed using a mixed-effect model and the restricted maximum likelihood estimator (REML) [21], using the midpoint of the testosterone measurement period as the moderator (independent variable: ‘collection year’), and mean testosterone serum levels as the observed effect size (dependent variable), associated with the reported or calculated sampling variances and number of subjects in each study. The Knapp-Hartung (KH) method [22] was applied to test the individual coefficients by accounting for the uncertainty in residual heterogeneity estimation. Since the age of the subjects is known to be an influencing factor for testosterone serum levels, we performed two additional sets of analyses: firstly, the meta-regression was repeated by explicitly including the mean age of the study subjects as an additional moderator in the model. Secondly, the analysis was again repeated after grouping the study groups into six distinct age groups: the first included patients from 18 to 30 years, the second from 31 to 40, the third from 41 to 50, the fourth from 51 to 60, the fifth from 61 to 70 and the sixth above 70 years. Each age group was then individually analysed using meta-regression, considering the mean testosterone serum levels as the observed effect size and the collection year as the only moderator. Similarly, considering that testosterone serum levels could be influenced by the laboratory method used, the meta-regression analysis was further performed after dividing the dataset according to the assay used for testosterone measurement. To this purpose, four groups were created, considering the following methods: immunometric assay (IA), enzyme-linked immunosorbent assay (ELISA); radio-immunoassay (RIA); and mass-spectrometry (MS).

Given the known interaction between BMI and subjects’ age [23, 24], a further meta-regression analysis was performed to evaluate the correlation between testosterone serum levels and the subjects’ BMI, using the same methodology reported above, namely considering mean BMI and mean subjects’ age as continuous moderators and mean serum testosterone levels as the effect size (outcome). In addition, considering the strict connection between LH and testosterone serum levels, LH trend across years was evaluated through meta-regression analysis following the same methodology reported above, using mean LH serum levels as the effect size and the midpoint of the LH measurement period as the continuous moderator. Similarly, meta-regression analyses were repeated using FSH and SHBG as dependent variable, years of blood collection as covariates, and subjects’ age and BMI as cofactors.

Finally, in order to evaluate the possible influence of environmental and social factors on hormones’ trend across years, we used in-house python code to look up such data for the geographic region and year of each sampled study (data obtained as described above). After merging the data, we performed an initial filtering by extracting the top 10 environmental and social factors showing the highest negative or positive correlation with mean testosterone levels. This allowed us to perform additional meta-regression analyses with the same methodology described above (mixed-effects model with REML and KH method), using the extracted environmental and social factors and the mean subjects’ age as moderators and the mean serum testosterone levels as effect size. Study groups for which such data could not be determined, i.e. not available for the specific year and geographic region of the sampling, were excluded from this analysis. Thus, these further sub-analyses were limited to a reduced dataset.

Statistical analysis was performed using the ‘Statistical Package for the Social Sciences’ software for Windows (version 28.0; SPSS Inc, Chicago, IL) and the *metafor* package [25] in R version 4.4.0 (R Core Team (2021). R: A language and environment for statistical computing. R Foundation for Statistical Computing, Vienna, Austria.URL https://www.R-project.org/). For all comparisons, p-values < 0.05 were considered statistically significant.

## Results

**Fig. 1:**
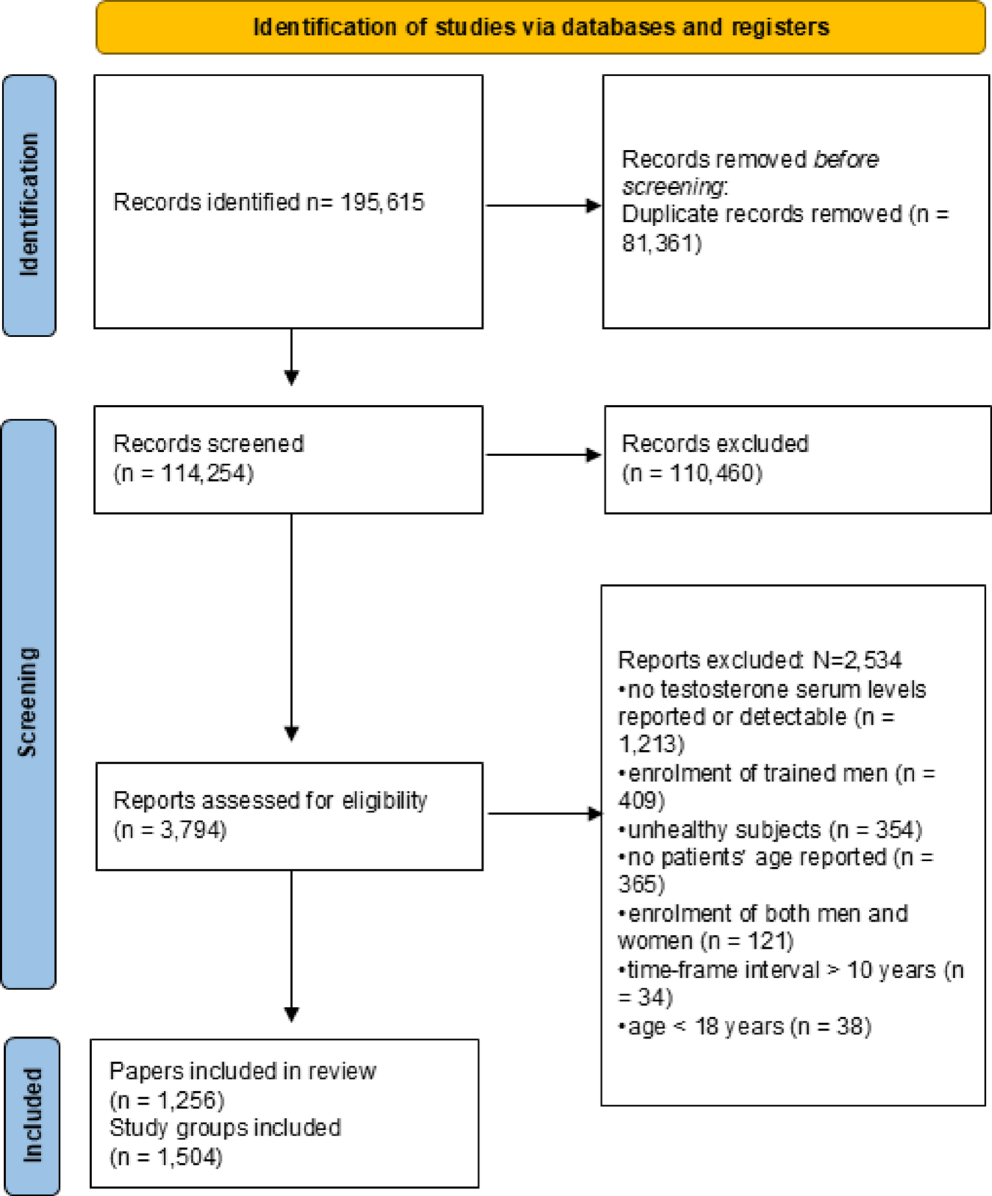
Literature search flow-chart

**Fig. 2:**
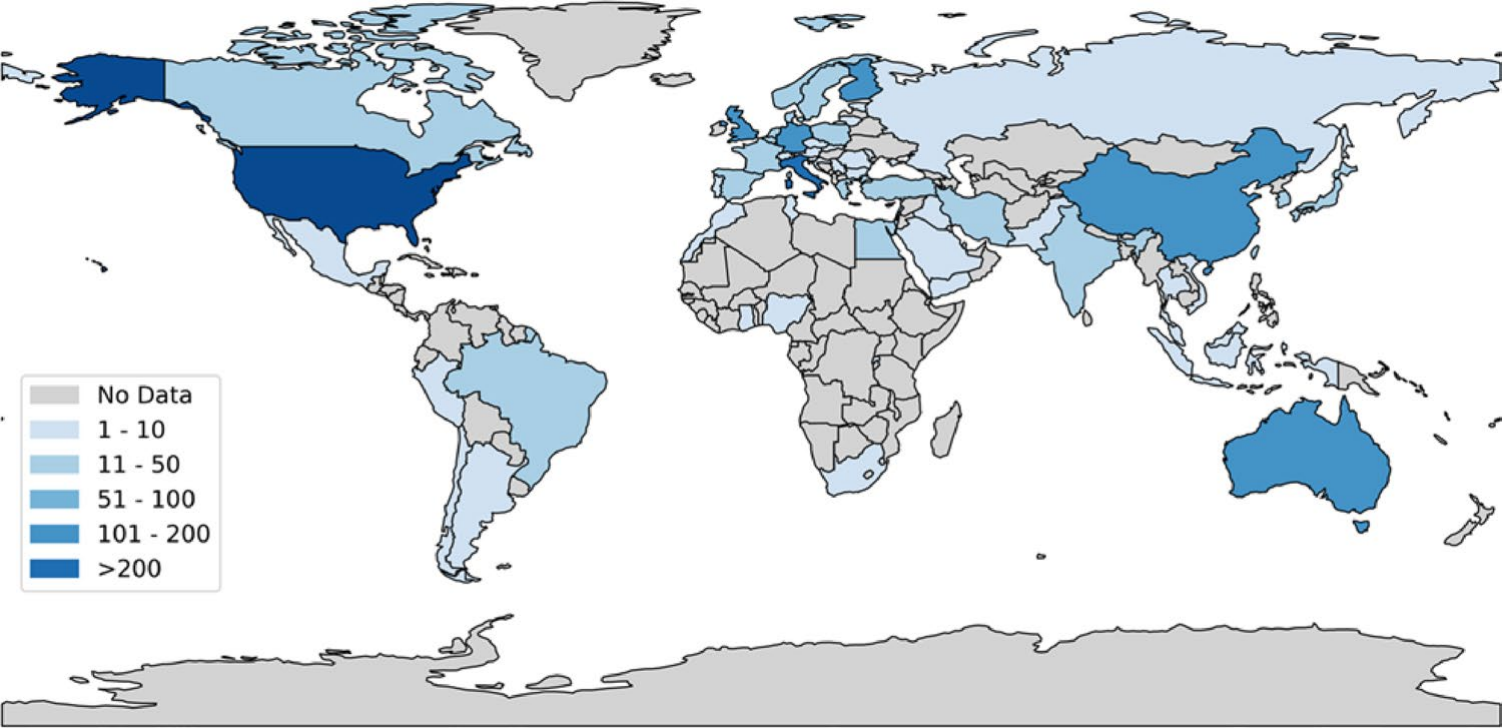
Geographical distribution of study groups included in the analyses

We identified 114,264 papers meeting our inclusion criteria since 1970 (Fig. 1). Out of these, 110,460 were excluded since no data useful for the analysis were reported, and 3,794 papers were selected for full-text evaluation (Fig. 1). During full-text examination, 2,534 papers were further excluded for various reasons: no reported or detectable testosterone serum levels (1,213 papers), enrolment of trained men (409 papers), inclusion of unhealthy subjects (354 papers), absence of patients’ age (365 papers), enrolment of both men and women (121 papers), studies conducted over a time-frame longer than 10 years (34 papers), and inclusion of men younger than 18 years (38 papers). Finally, 1,256 papers, comprising 1,504 study groups (Fig. 1), were included, involving a total of 1,064,891 subjects with a mean age of 42.0 ± 7.0 years (minimum 18.5– maximum 93.2) (all references are reported in supplementary material, page 20). Figure 2 reports the geographical distribution of papers included.

Among the included papers, 556 were prospective clinical trials (36.9%), 708 were case-control studies (47.1%), and 240 were cross-sectional studies (16.0%). Testosterone serum levels were available for all study groups (18.5 ± 4.5 nmol/L, from 8.4 to 39.5 nmol/L), while LH was reported in 492 study groups (5.2 ± 2.8 IU/L, 1.1–19.3 IU/L), FSH in 422 (5.5 ± 3.6 IU/L, 1.0-19.9 IU/L), SHBG in 489 (818.8 ± 1728.9 nmol/L, 0.3–3821.0 nmol/L) and BMI in 746 (25.7 ± 2.6 kg/m^2^, 13.3–44.0 kg/m^2^).

### Body mass index (BMI) and sex hormone binding globulin (SHBG)

**Fig. 3:**
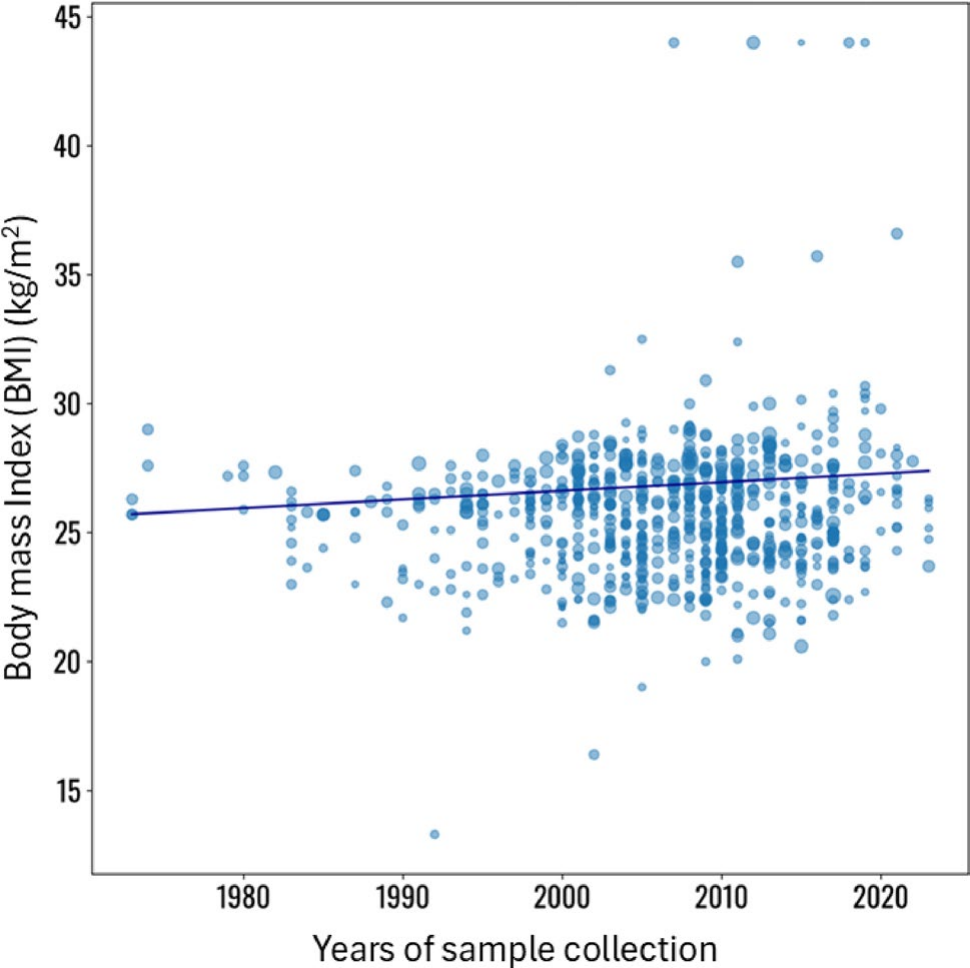
Meta-regression analysis using body mass index (BMI) as effect of the regression, the years of sample collection as covariates and subjects’ number and age as co-factors. Statistical details are reported in Supplementary material

BMI was reported in 746 study groups (number of subjects = 712,289). Despite the well-known increasing rate of obesity worldwide, the BMI in the studies evaluated here did not show any significant change across the years of observation (Coefficient 0.1, SE: 0.1, *p* = 0.617) (Fig. 3, and supplementary material, page 3). Similarly, SHBG (489 study groups, number of subjects = 312,498) did not show any significant trend across years of observation (Coefficient − 0.2, SE 0.3, *p* = 0.453) (Supplementary Fig. 1 and supplementary material, page 4–5, for statistical analysis details).

### Total testosterone serum levels

Regression analyses revealed that the mean serum testosterone levels were inversely related to the years of sample collection (*p* = 0.033) and subjects’ age (*p* < 0.001). Moreover, a positive direct relationship was found between testosterone and SHBG (*p* = 0.007) and LH serum levels (*p* = 0.007) (Table 2).

**Table 2:** Regression analyses using mean testosterone serum levels as dependent variable

	B	Standard error	Beta	*p*-value	95%CI	
					Lower limit	Upper limit
*Constant*	*159.307*	*63.940*		*0.014*	*32.837*	*285.778*
Year of sample collection	**−0.069**	**0.032**	**−0.155**	**0.033**	**−0.132**	**−0.006**
Age	**−0.131**	**0.026**	**−0.522**	**< 0.001**	**−0.181**	**−0.080**
SHBG	**0.047**	**0.017**	**0.200**	**0.007**	**0.013**	**0.081**
LH	**0.453**	**0.166**	**0.275**	**0.007**	**0.124**	**0.782**
FSH	−0.252	0.159	−0.205	0.114	−0.566	0.062
BMI	**−0.560**	**0.211**	**−0.278**	**0.009**	**−0.979**	**−0.141**

Italics data represent the constant of the mathematical formula

Bold charactersrepresents significant regression

*BMI = *body mass index*; FSH =* follicle-stimulating hormone*; LH = *luteinising hormone*; SHBG =* sex hormone binding globulin

The DW analysis indicated a slight, positive autocorrelation between testosterone serum levels and years of blood collection (DW: 1.697). The demonstration of an autocorrelation between data confirmed the potential existence of a trend across the years.

**Fig. 4:**
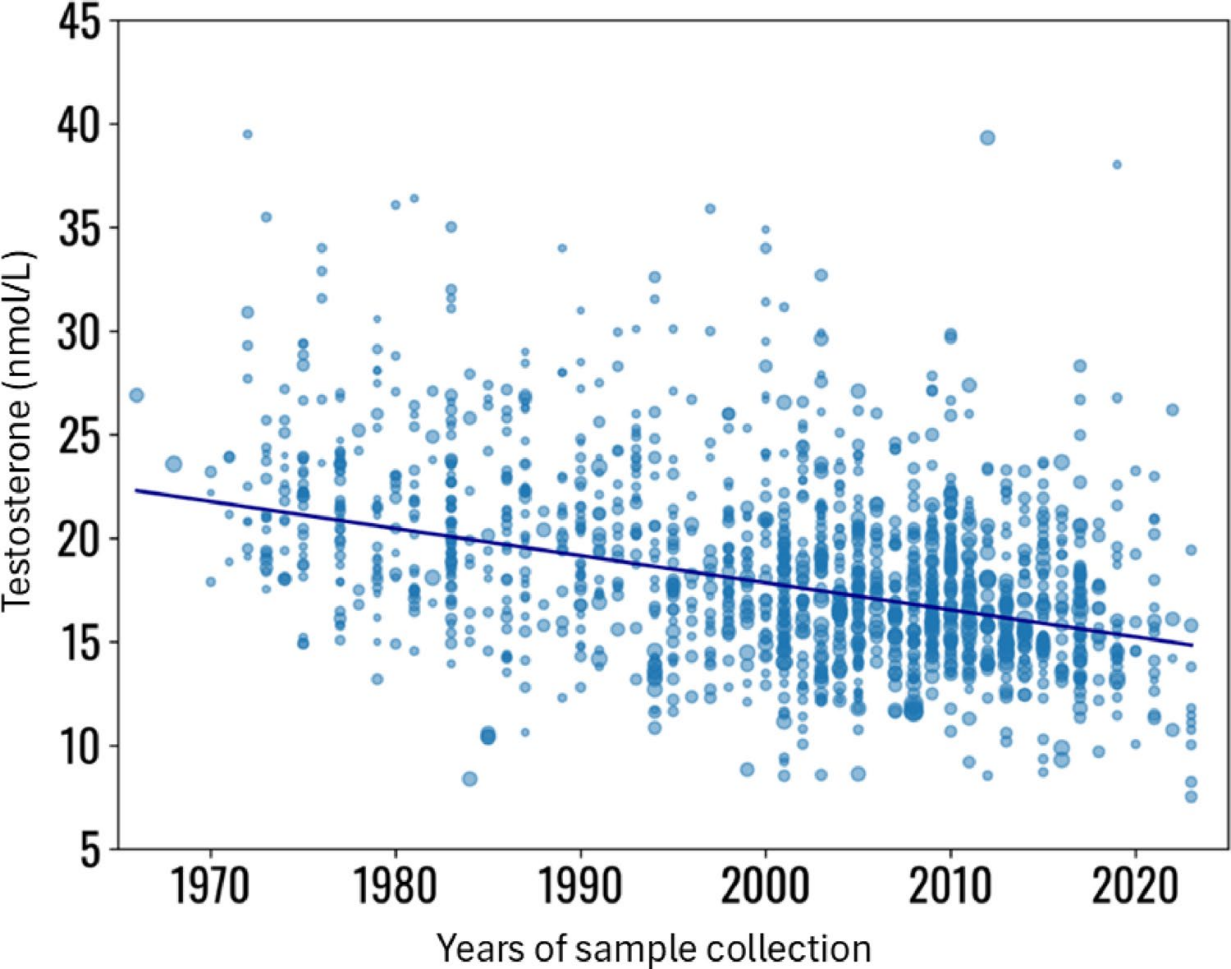
Meta-regression analysis, using testosterone serum levels as effect of the regression, the years of sample collection as covariates and subjects’ number, age and BMI as co-factors (BMI was available for 742 study groups). Statistical details are reported in Supplementary material

Meta-regression analysis was conducted with the random-effects model and using mean testosterone serum levels as the effect and the SD^2 as the variance. The years of sample collection were considered as a covariate, while the number of subjects enrolled, subjects’ age and BMI as factors affecting the regression. A significant reduction in testosterone serum levels across the years of measurement was observed (Coefficient − 0.6, SE 0.2, *p* < 0.001) (see supplementary material, page 6, for more details about meta-regression analysis) (Fig. 4), independent from BMI and age suggesting a genuine testosterone decrease. This significant trend remained also when only study groups with BMI < 25 kg/m^2^ were considered (Coefficient − 0.2, SE 0.1, *p* = 0.041) (Supplementary material, page 7),

Meta-regression analyses were repeated based on the assay used for testosterone measurement. The decline in total testosterone serum levels across years was confirmed for study groups employing IA, ELISA, and RIA (Table 3). Conversely, when considering only study groups using MS, the decline in total testosterone serum levels across years was not confirmed (Table 3). However, among the 117 papers using MS, only five (comprising eight study groups) were conducted before 2000, specifically between 1991 and 1998. While the study groups in groups 1, 2, and 3 were equally distributed across fifty years of observations, the subgroup applying MS evaluated the testosterone trend over a limited timeframe of approximately thirty years. Considering the limited number of studies, the analysis of testosterone trend measured by MS requires more data over a longer observation period.

**Table 3:** Meta-regression analyses of total testosterone serum levels across years of blood sample collection, considering study groups grouped according to the methodology applied for hormones measurement

Group	Assay	Years (median [min, max])	Number study groups (subjects)	Chi-squared	*p*-value	I^2^
Total testosterone serum levels						
1	Immuno-assay (IA)	2008 (1966–2023)	668 (870489)	393.0	< 0.001	44.5
2	ELISA	2014 (1994–2023)	61 (14731)	51.1	< 0.001	90.1
3	Radio-IA (RIA)	2010 (2001–2022)	658 (79782)	787.6	< 0.001	62.8
4	Mass-spectrometry	1992 (1968–2021)	117 (99889)	8.8	0.845	0.0

*ELISA = *Enzyme-linked immunosorbent assay

**Fig. 5:**
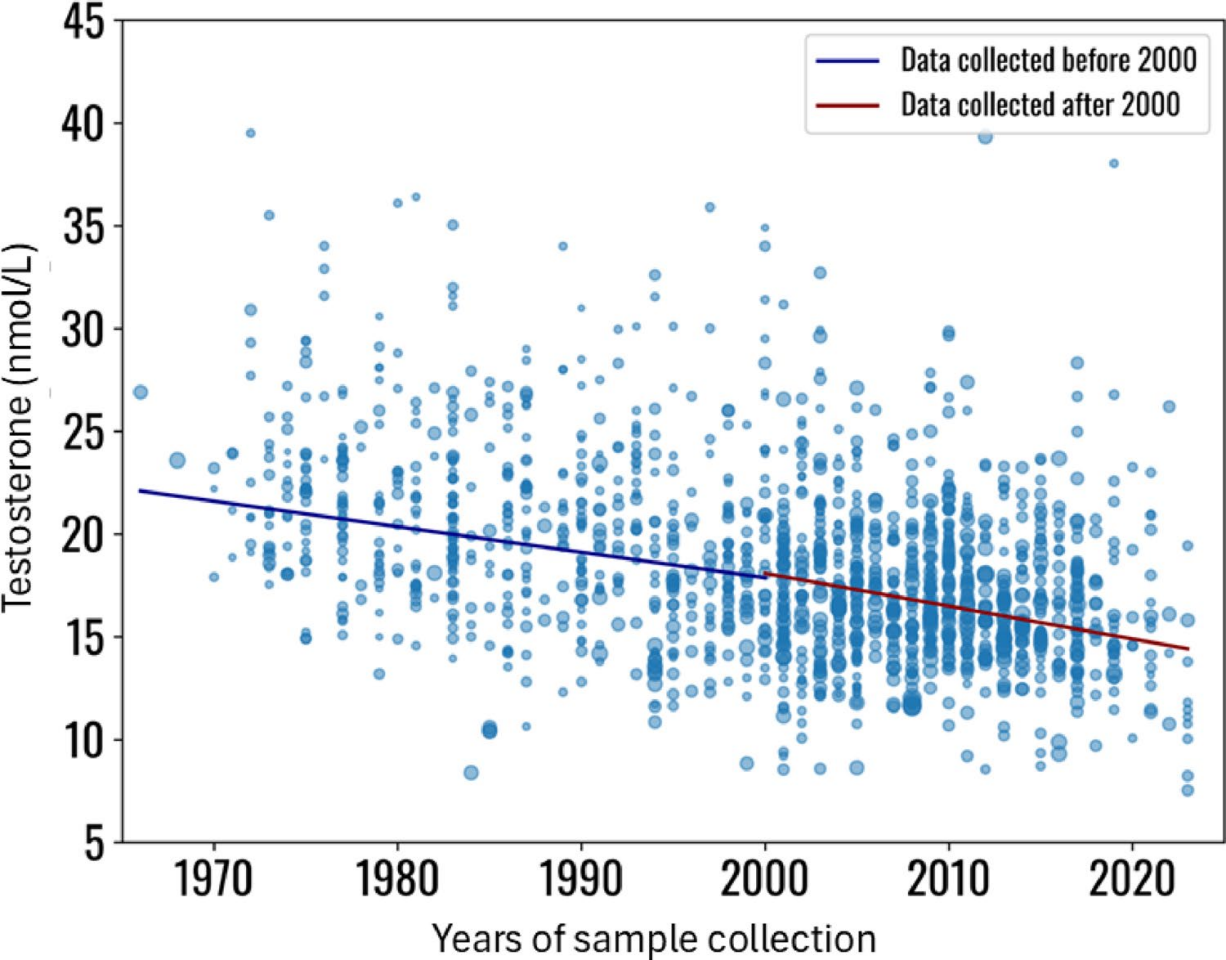
Meta-regression analysis, using testosterone serum levels as effect of the regression, the years of sample collection as covariates and dividing the dataset in two groups, according to the year of blood collection, before or after 2000. Statistical details are reported in Supplementary material

Meta-regression analyses for testosterone serum levels were repeated dividing the entire dataset in two groups. The first considered study groups in which blood samples were collected before 2000 and the second after 2000. Interestingly, the two meta-regression analyses confirmed the testosterone decline across years (pre-2000: Coefficient: −0.1, SE: 0.1, *p* < 0.001, post-2000: Coefficient: −0.2, SE: 0.1, *p* < 0.001), although with different trends (Fig. 5, supplementary material, page 8).

Finally, considering the uneven distribution of study groups around the globe, meta-regression analysis between testosterone and years of blood collection (with subjects’ age as cofactor) were repeated considering only papers published in USA. The significant negative trend of testosterone serum levels across years of observations was confirmed (coefficient − 0.14, SE 0.03, p-value < 0.001) (number of study groups = 366, number of subjects = 128,840, number of studies with reported BMI = 156) (Supplementary Fig. 2, and supplementary material, page 9–10, for more statistical analysis details).

### Gonadotropin serum levels

LH serum levels were reported in 492 (number of subjects = 114,961), while FSH was reported in 422 (number of subjects = 88,064) study groups. Regression analyses were performed using LH serum levels as the dependent variable, revealing a negative relationship between LH and years of blood collection (*p* = 0.014), and a positive relationship with testosterone (*p* = 0.007) and FSH serum levels (*p* < 0.001) (Table 4).

**Table 4:** Regression analyses using mean luteinising hormone (LH) serum levels as dependent variable

	B	Standard error	Beta	*p*-value	95%CI	
					Lower limit	Upper limit
*Constant*	*80.023*	*32.431*		*0.015*	*15.876*	*144.17*
**Year of sample collection**	**−0.040**	**0.016**	**−0.148**	**0.014**	**−0.072**	**−0.008**
Age	−0.022	0.014	−0.142	0.126	−0.049	0.006
SHBG	0.011	0.009	0.077	0.219	−0.007	0.029
**Testosterone**	**0.116**	**0.043**	**2.272**	**0.007**	**0.032**	**0.201**
**FSH**	**0.635**	**0.060**	**10.647**	**< 0.001**	**0.517**	**0.753**
BMI	0.135	0.072	0.132	0.064	−0.008	0.278

*BMI = *body mass index;* FSH =* follicle-stimulating hormone; *SHBG = *sex hormone binding globulin

At DW analyses, LH and years of blood collection showed a slight, positive autocorrelation, confirming the potential existence of a trend across the years.

**Fig. 6:**
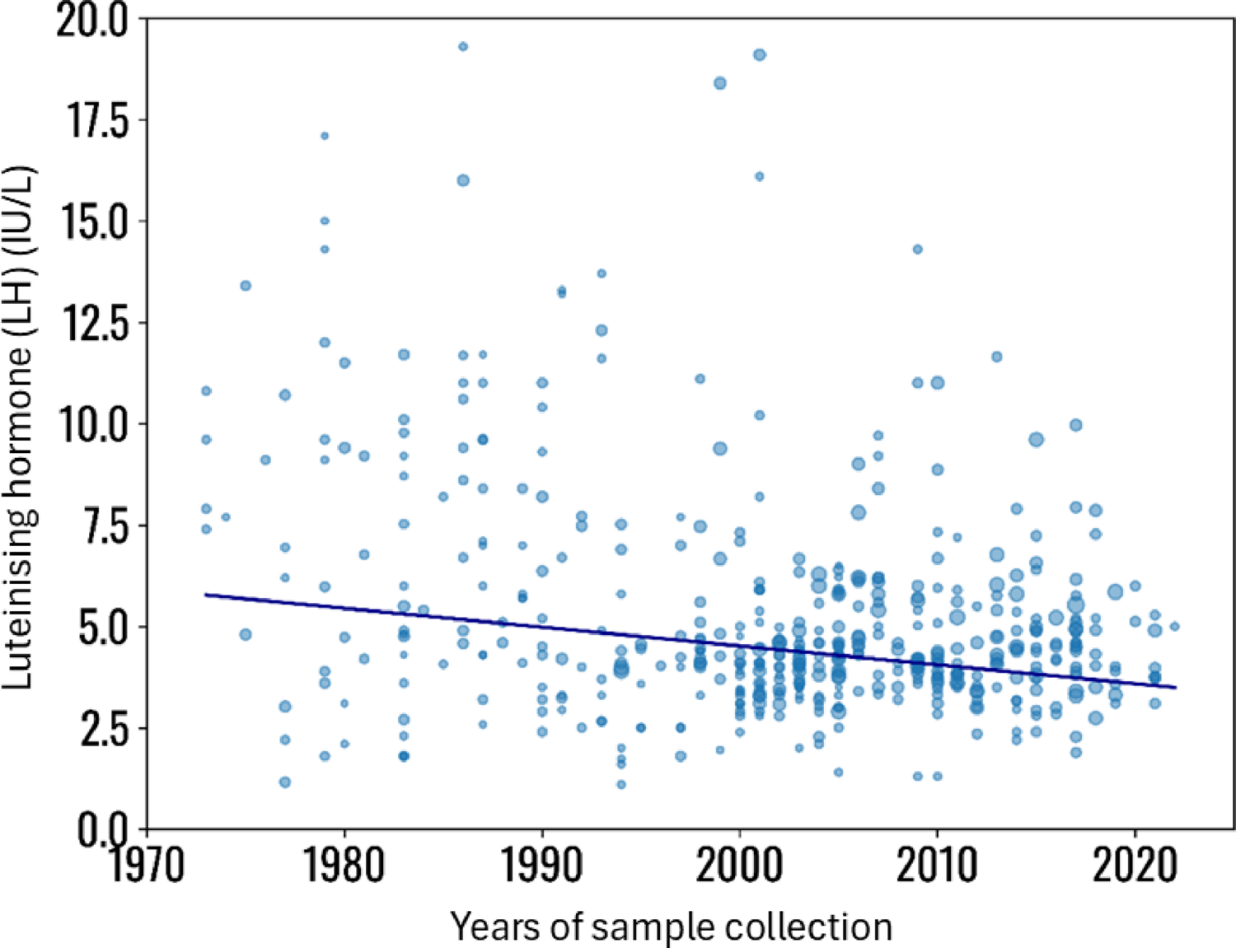
Meta-regression analysis, using luteinising hormone (LH) serum levels as effect of the regression, the years of sample collection as covariates and patients’ number and age as co-factors. The analysis was not corrected for BMI, since this parameter was available only for 52 study groups. Statistical details are reported in Supplementary material

LH serum levels showed a significant decline over the years (Coefficient − 0.1, SE: 0.1, *p* < 0.001), with patients’ age as the adjusting variable (Fig. 6, and supplementary material, page 11). The meta-regression analysis was not adjusted for BMI, since only 52 studies reported both LH and BMI, significantly reducing the sample size for adjusted analyses. Conversely, FSH serum levels did not show any change across the years of observation (Coefficient − 0.1, SE: 0.1, *p* = 0.143), even after adjustment for subjects’ age (Supplementary Fig. 3, and supplementary material, page 12–13, for more statistical analysis details).

### The role of the subjects’ age

**Fig. 7:**
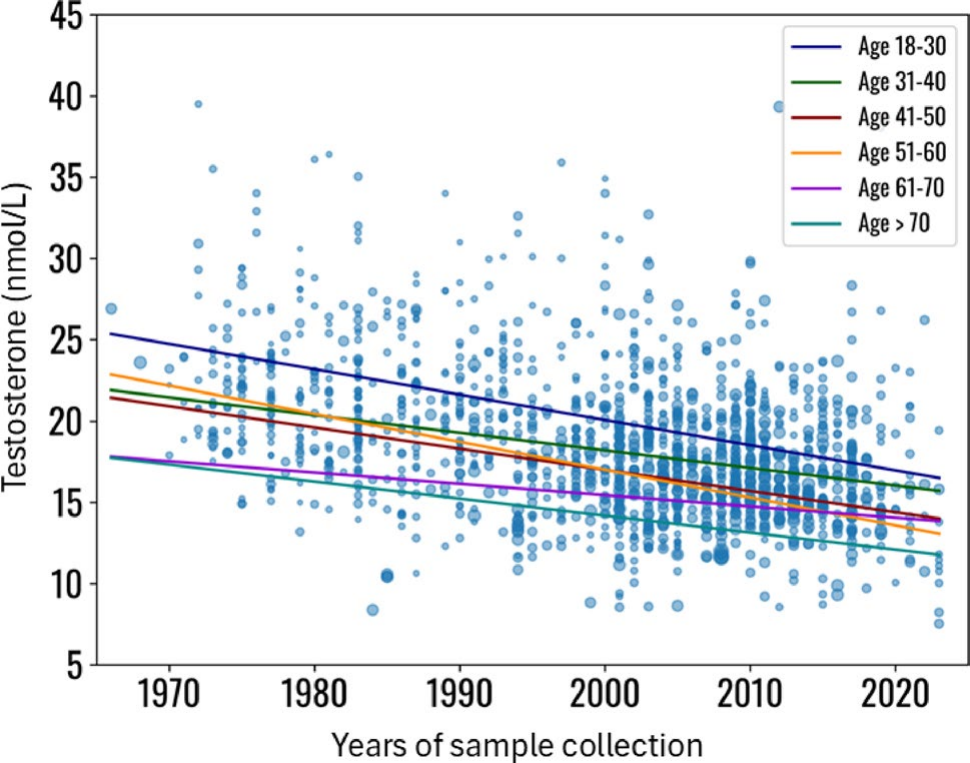
Meta-regression analysis, using testosterone serum levels as effect of the regression, the years of sample collection as covariates and dividing the dataset in groups according to subjects’ age. Statistical details are reported in Supplementary material

The subdivision of study groups according to subjects’ age created six groups, in which the significant decline in testosterone serum levels across the years of observation was confirmed (Table 5; Fig. 7, supplementary material, page 14–16). Similarly, a decrease in LH serum levels was confirmed in three out of six groups (Table 5 and supplementary material, page 17–19).

**Table 5:** Meta-regression analyses of total testosterone and luteinising hormone serum levels across years of blood sample collection, considering study groups grouped according to subjects’ age

Group	Subjects’ Age	Number study groups (subjects)	Coefficient	*p*-value	I^2^
**Total testosterone serum levels**					
1	18–30 years	384 (39674)	−0.15	**< 0.001**	26.8
2	31–40 years	416 (80305)	−0.11	**< 0.001**	17.0
3	41–50 years	237 (85081)	−0.13	**< 0.001**	5.4
4	51–60 years	249 (613509)	−0.17	**< 0.001**	20.1
5	61–70 years	147 (217699	−0.07	**0.005**	9.3
6	> 70 years	75 (28713)	−0.11	**0.003**	17.8
**LH serum levels**					
1	18–30 years	152 (19997)	−0.03	**0.045**	79.3
2	31–40 years	190 (45605)	−0.07	**< 0.001**	68.3
3	41–50 years	58 (9869)	−0.02	0.249	65.5
4	51–60 years	45 (27638)	0.01	0.711	0.0
5	61–70 years	24 (5025)	−0.03	0.500	93.0
6	> 70 years	23 (9514)	0.16	**0.002**	19.5

Bold characters represent p-value <0.05, showing statistical significance

*LH = *luteinising hormone

### The role of the environment

To explore whether the decline in total testosterone serum levels was related to environmental factors, data on energy production, consumption, trade, and emissions were included in meta-regression analyses as moderators. After adjusting for these variables, the decline in total testosterone serum levels across years was confirmed (136 study groups, Chi-squared 148.8, *p* = 0.006, I^2^ 28.7%). Similarly, the decline in LH serum levels across years was confirmed after adjustment for environmental data (134 study groups, Chi-squared 43.0, *p* = 0.001, I^2^ 56.8%).

The decline in testosterone serum levels across years of observation was also confirmed when considering variables related to population change (Chi-squared 177.0, *p* < 0.001, I^2^ 48.9%). Conversely, the decline in LH serum levels was not confirmed after adjustment for demographic parameters (Chi-squared 28.7, *p* = 0.324, I^2^ 0.0%). However, these results were based on 181 study groups for the first adjustment (testosterone) and 75 for the second one (LH), clearly limiting the strength of the results. To further investigate this, the meta-regression analysis was repeated with a reduced number of covariates, retaining only variables clearly related to population growth, such as life expectancy at birth, mortality and death rate, annual birth rate, fertility rate, and population number. This further analysis was performed for LH on 399 study groups, and the significant trend in LH levels across years was confirmed (Chi-squared 302.6, *p* < 0.001, I^2^ 47.3%). Thus, it appears that the trend in LH serum levels is not affected by population changes, at least when considering population growth-related parameters.

## Discussion

This analysis describes, for the first time, a significant decrease in mean total testosterone serum levels in over one million healthy men over the last 55 years. This result completes the complex picture of testosterone decline across years suggested in other work evaluating smaller cohorts [9, 26, 27]. This declining trend is associated with a significant decrease in LH serum levels, indicating that the testosterone decline does not primarily originate in the testis.

The relationship between obesity and testosterone serum levels is well-documented [28], and the global rise in obesity prevalence could be the first hypothesis at explaining the testosterone trend detected here. However, our data suggests otherwise. First, BMI did not show a significant increase across the years of observations in the population studied. Second, after adjusting for subjects’ BMI, the trends in testosterone and LH levels remained significant, indicating that factors other than body weight are likely contributing to these hormonal changes. However, since BMI data were available only in a subgroup of studies, we cannot definitively exclude the role of BMI in the observed decrease in testosterone levels. An interesting experimental model of metabolic syndrome demonstrated that rabbits fed a high-fat diet exhibit a form of functional hypogonadotropic hypogonadism, attributed to hypothalamic inflammation affecting the region where gonadotropin-releasing hormone (GnRH) neurons are located [29]. However, the subjects in our study were neither hypogonadal nor affected by metabolic syndrome. Overall, our results indicate that patients included in the analyzed studies did not show an increasing trend in BMI over the observation period. Although this finding contrasts with the well-documented epidemiological evidence of rising obesity prevalence in the general population, it represents a further strength of our analysis. While the association between increasing BMI and decreasing testosterone levels is well-established, our findings confirm a decline in testosterone levels that is entirely independent of obesity incidence. Specifically, by analyzing a large cohort of patients classified as healthy in the original studies, without selection based on testosterone or BMI levels, the absence of a BMI trend over the evaluation period ensures that our cohort is free from the influence of obesity on testosterone production.

This testosterone decline over the years should be considered together with the significant decrease in sperm parameters described by others. Indeed, the most recent systematic reviews show a significant decline in both sperm concentration and total sperm number in North America, Europe and Australia since 1973 [2, 3], although the topic remains controversial [5–7, 30]. Our data, although not reporting semen parameters, add another suggestion in the alarming picture regarding male fertility decline. Several hypotheses have been proposed to explain the decline in sperm production over the years, especially advocating a primary testicular damage due to exogenous factors, such as environment, pollution and endocrine disruptors [31]. Our results suggests that the decreasing testicular function could be the result of impaired pituitary stimulation/pulsatile GnRH secretion, resulting in reduction of serum testosterone [32].

The progressive reduction in both LH and testosterone serum levels could be related to a central resetting of the hypothalamic regulation of LH secretion for some still unknown reason. If the pituitary gland was primarily involved in this change, for instance due to endocrine disruptors/pollution acting at the pituitary level, we might expect to observe similar Temporal trends in other pituitary hormones. However, in the literature, only few studies evaluated Temporal trends in other pituitary hormones, showing temporal trends for growth hormone [33–36], prolactin [37] and thyroid-stimulating hormone levels [38–40], although with a significantly lower sample size than that used in our analyses. No demonstration of gonadotropin trends in adults were available so far. The decline in LH levels in healthy men described here is not accompanied by corresponding changes in serum FSH levels. This should be interpreted considering several confounding factors, such as the variability in FSH assays and the selection of subjects of unknown fertility status. Furthermore, additional comments on the temporal trend of FSH are beyond the scope of our analysis, as other variables related to Sertoli cell activity (e.g., Inhibin B) are required to better understand potential changes in FSH action. Thus, the lack of FSH temporal trend must be cautiously viewed, since the selection of subjects could not be appropriate for that aim. The discrepancy between the trends observed in LH and FSH levels over time suggests that the underlying mechanism could involve the hypothalamic rather than pituitary function. We speculate that the decline in testosterone serum levels over time is dependent on a reduction in LH, which, in turn, could be the consequence of subtle changes in GnRH pulsatility. The secretion of LH and FSH is known to be regulated differently by GnRH. Indeed, the secretion of LH is dependent on GnRH pulse frequency, while the secretion of FSH is more dependent on the pulse amplitude [41]. Thus, the declining trend in LH but not FSH serum levels, suggests a central, hypothalamic site of action of (a) unknown, ongoing, disturbing factor(s).

The possible role of exogenous factors, such as endocrine disruptors [42], was advocated to explain potential hormonal trends in the literature. Here, we used data on energy production, consumption, trade, emissions, and air pollution, but the decline in testosterone and LH levels over the years remained unchanged after these adjustments. Therefore, the trend in LH and testosterone serum levels described here is apparently not related to environmental factors, at least considering the parameters available for our analyses. Similarly, the correction for the progressive world population growth and related factors did not lead to any significant change and were not informative. In this context, factors known to potentially cause so-called functional hypogonadism, such as lifestyle habits and stress [43–46], should be considered when interpreting the hormonal trends observed in our data. However, these factors are difficult to collect consistently on a global scale, making it challenging to assess their role in the decline of testosterone levels over time. While the role of environmental pollution and population density cannot be excluded, the mechanism behind the decrease in hypothalamic-pituitary gonadal function in healthy men described here remains unknown. The question remains whether the progressive decline in testosterone and more generally testicular function is an adaptation to overpopulation. Neither our analyses, nor the current literature suggest this, but if population dynamics play a role, the forecast of the world population growth with a reduction in global population (and pollution) in the next 5–10 decades should result in a reversal of the temporal trends in testicular function and the current decline in birth rates affecting modern society [47] will lead to a new equilibrium. The declining trend in testosterone serum levels could be an adaptation to the current disequilibrium, necessary to achieve a new population homeostasis.

This study has several limitations. First, our analyses are based on data from healthy subjects obtained from studies available in the literature. Therefore, potential selection and participation biases cannot be ruled out, despite the rigorous literature search and quality checks performed. Indeed, biases related to the retrospective design of the study and the heterogeneity of subjects could not be eliminated. Second, we adjusted the meta-regression analyses with BMI, environmental and population-related parameters. However, these data were available for only a proportion of the studies included, limiting the statistical power of the correlation analyses. Finally, limitations inherent to the applied methodology (i.e., meta-regression analysis) cannot be excluded in the interpretation and discussion. In this systematic review of the literature, we sought to obtain more robust data by applying meta-regression analyses. However, these statistical approaches rely on aggregate data, which restricts the use of more rigorous methodological statistical techniques.

In conclusion, we describe here a temporal trend in testosterone serum levels in men over the last 50 years that could be interpreted as an adaptation of the hypothalamic-pituitary-gonadal axis. While subjects’ life-styles, population dynamics, global overpopulation and pollution might play a role on this trend, the data currently available do not reveal a significant role of such factors. Whatever the mechanism, the parallel decline of testosterone and LH in healthy men emerges from our results, suggesting an action at the hypothalamic-pituitary level possibly with a suggestive resetting of the GnRH pulse generator. Testosterone serum levels have been progressively declining over the years in men, although they remain within reference ranges. The contribution of the declining testicular function, both in terms of testosterone and sperm, to the global reduction of fecundity/fertility remains to be investigated.

## Supplementary Information

Below is the link to the electronic supplementary material.

Supplementary Material 1

## Data Availability

De-identified data are available and can be requested to the corresponding author.

## References

[1] Carlsen E, Giwercman A, Keiding N, Skakkebaek NE (1992) Evidence for decreasing quality of semen during past 50 years. BMJ (Clinical Res Ed) 305(6854):609–613 10.1136/bmj.305.6854.609 PMC1883354 1393072

[2] Levine H, Jorgensen N, Martino-Andrade A, Mendiola J, Weksler-Derri D, Mindlis I, Pinotti R, Swan SH (2017) Temporal trends in sperm count: a systematic review and meta-regression analysis. Hum Reprod Update 23(6):646–659. 10.1093/humupd/dmx022 28981654 10.1093/humupd/dmx022 PMC6455044

[3] Levine H, Jørgensen N, Martino-Andrade A, Mendiola J, Weksler-Derri D, Jolles M, Pinotti R, Swan SH (2023) Temporal trends in sperm count: a systematic review and meta-regression analysis of samples collected globally in the 20th and 21st centuries. Hum Reprod Update 29(2):157–176. 10.1093/humupd/dmac035 36377604 10.1093/humupd/dmac035

[4] Boulicault M, Perret M, Galka J, Borsa A, Gompers A, Reiches M, Richardson S (2022) The future of sperm: a biovariability framework for Understanding global sperm count trends. Hum Fertil (Cambridge England) 25(5):888–902. 10.1080/14647273.2021.1917778 10.1080/14647273.2021.1917778 33969777

[5] te Velde E, Burdorf A, Nieschlag E, Eijkemans R, Kremer JA, Roeleveld N, Habbema D (2010) Is human fecundity declining in Western countries? Human reproduction. (Oxford England) 25(6):1348–1353. 10.1093/humrep/deq085 10.1093/humrep/deq085 20395222

[6] Nieschlag E, Lerchl A (1996) Declining sperm counts in European men–fact or fiction? Andrologia 28(6):305–306. 10.1111/j.1439-0272.1996.tb02805.x 9021040 10.1111/j.1439-0272.1996.tb02805.x

[7] Nieschlag E, Lerchl A (2013) Sperm crisis: what crisis? Asian J Androl 15(2):184–186. 10.1038/aja.2012.90 23001441 10.1038/aja.2012.90 PMC3739166

[8] Zuvela E, Matson P (2024) Effect of the technical variability of counting chambers upon the interpretation of sperm concentration results. Reprod Biomed Online 48(5):103777. 10.1016/j.rbmo.2023.103777 38460281 10.1016/j.rbmo.2023.103777

[9] Andersson AM, Jensen TK, Juul A, Petersen JH, Jørgensen T, Skakkebaek NE (2007) Secular decline in male testosterone and sex hormone binding Globulin serum levels in Danish population surveys. J Clin Endocrinol Metab 92(12):4696–4705. 10.1210/jc.2006-2633 17895324 10.1210/jc.2006-2633

[10] Chodick G, Epstein S, Shalev V (2020) Secular trends in testosterone- findings from a large state-mandate care provider. Reproductive Biology Endocrinology: RB&E 18(1):19. 10.1186/s12958-020-00575-2 32151259 10.1186/s12958-020-00575-2 PMC7063751

[11] Harman SM, Metter EJ, Tobin JD, Pearson J, Blackman MR (2001) Longitudinal effects of aging on serum total and free testosterone levels in healthy men. Baltimore longitudinal study of aging. J Clin Endocrinol Metab 86(2):724–731. 10.1210/jcem.86.2.7219 11158037 10.1210/jcem.86.2.7219

[12] Auerbach JM, Moghalu OI, Das R, Horns J, Campbell A, Hotaling J, Pastuszak AW (2022) Evaluating incidence, prevalence, and treatment trends in adult men with hypogonadism in the united States. Int J Impot Res 34(8):762–768. 10.1038/s41443-021-00471-2 34845356 10.1038/s41443-021-00471-2

[13] Handelsman DJ (2000) Androgen Physiology, Pharmacology, Use and Misuse. In: Feingold, K.R., Anawalt, B., Blackman, M.R., Boyce, A., Chrousos, G., Corpas, E., de Herder, W.W., Dhatariya, K., Dungan, K., Hofland, J., Kalra, S., Kaltsas, G., Kapoor, N., Koch, C., Kopp, P., Korbonits, M., Kovacs, C.S., Kuohung, W., Laferrère, B., Levy, M., McGee, E.A., McLachlan, R., New, M., Purnell, J., Sahay, R., Shah, A.S., Singer, F., Sperling, M.A., Stratakis, C.A., Trence, D.L., Wilson, D.P. (eds.) Endotext. MDText.com, Inc. Copyright © 2000–2024, MDText.com, Inc., South Dartmouth (MA)

[14] Yeap BB, Grossmann M, McLachlan RI, Handelsman DJ, Wittert GA, Conway AJ, Stuckey BG, Lording DW, Allan CA, Zajac JD, Burger HG (2016) Endocrine society of Australia position statement on male hypogonadism (part 1): assessment and indications for testosterone therapy. Med J Australia 205(4):173–178. 10.5694/mja16.00393 27510348 10.5694/mja16.00393

[15] Zhou CK, Advani S, Chaloux M, Gibson JT, Yu M, Bradley M, Hoover RN, Cook MB (2020) Trends and patterns of testosterone therapy among U.S. Male medicare beneficiaries, 1999 to 2014. J Urol 203(6):1184–1190. 10.1097/ju.0000000000000744 31928462 10.1097/JU.0000000000000744 PMC7211140

[16] Bhasin S, Brito JP, Cunningham GR, Hayes FJ, Hodis HN, Matsumoto AM, Snyder PJ, Swerdloff RS, Wu FC, Yialamas MA (2018) Testosterone therapy in men with hypogonadism: an endocrine society clinical practice guideline. J Clin Endocrinol Metab 103(5):1715–1744. 10.1210/jc.2018-00229 29562364 10.1210/jc.2018-00229

[17] Mulhall JP, Trost LW, Brannigan RE, Kurtz EG, Redmon JB, Chiles KA, Lightner DJ, Miner MM, Murad MH, Nelson CJ, Platz EA, Ramanathan LV, Lewis RW (2018) Evaluation and management of testosterone deficiency: AUA guideline. J Urol 200(2):423–432. 10.1016/j.juro.2018.03.115 29601923 10.1016/j.juro.2018.03.115

[18] Sellke N, Omil-Lima D, Sun HH, Tay K, Rhodes S, Loeb A, Thirumavalavan N (2023) Trends in testosterone prescription during the release of society guidelines. Int J Impot Res. 10.1038/s41443-023-00709-1 37130972 10.1038/s41443-023-00709-1 PMC10152018

[19] Carter IV, Callegari MJ, Jella TK, Mahran A, Cwalina TB, Muncey W, Loeb A, Thirumavalavan N (2023) Trends in testosterone prescription amongst medical specialties: a 5-year CMS data analysis. Int J Impot Res 35(4):1–5. 10.1038/s41443-021-00497-6 34992225 10.1038/s41443-021-00497-6

[20] Fallara G, Pozzi E, Belladelli F, Boeri L, Capogrosso P, Corona G, D’Arma A, Alfano M, Montorsi F, Salonia A (2024) A systematic review and Meta-analysis on the impact of infertility on men’s general health. Eur Urol Focus 10(1):98–106. 10.1016/j.euf.2023.07.010 37573151 10.1016/j.euf.2023.07.010

[21] Kim S, Alahmad ME, Oh T, Love A (2024) Athletic justice: scale development and validation. Heliyon 10(2):e24359. 10.1016/j.heliyon.2024.e24359 38293548 10.1016/j.heliyon.2024.e24359 PMC10827502

[22] Knapp G, Hartung J (2003) Improved tests for a random effects meta-regression with a single covariate. Stat Med 22(17):2693–2710. 10.1002/sim.1482 12939780 10.1002/sim.1482

[23] Goodarzynejad H, Meaney C, Brauer P, Greiver M, Moineddin R, Monavvari AA (2022) Recent trends in adult body mass index and prevalence of excess weight: data from the Canadian primary care Sentinel surveillance network. Can Fam Physician 68(2):128–138. 10.46747/cfp.6802128 35177505 10.46747/cfp.6802128 PMC9842178

[24] Jarrett B, Bloch GJ, Bennett D, Bleazard B, Hedges D (2010) The influence of body mass index, age and gender on current illness: a cross-sectional study. Int J Obes 34(3):429–436. 10.1038/ijo.2009.258 10.1038/ijo.2009.258 20010903

[25] Viechtbauer W (2010) Conducting Meta-Analyses in R with the metafor package. J Stat Softw 36(3):1–48. 10.18637/jss.v036.i03

[26] Travison TG, Araujo AB, Hall SA, McKinlay JB (2009) Temporal trends in testosterone levels and treatment in older men. Current opinion in endocrinology, diabetes, and obesity 16(3):211–217. 10.1097/med.0b013e32832b6348 19396984 10.1097/med.0b013e32832b6348

[27] Hsu B, Cumming RG, Hirani V, Blyth FM, Naganathan V, Le Couteur DG, Seibel MJ, Waite LM, Handelsman DJ (2016) Temporal trend in androgen status and androgen-Sensitive outcomes in older men. J Clin Endocrinol Metab 101(4):1836–1846. 10.1210/jc.2015-3810 26918290 10.1210/jc.2015-3810

[28] Corona G, Giagulli VA, Maseroli E, Vignozzi L, Aversa A, Zitzmann M, Saad F, Mannucci E, Maggi M (2016) THERAPY OF ENDOCRINE DISEASE: testosterone supplementation and body composition: results from a meta-analysis study. Eur J Endocrinol/Eur Fed Endocr Soc 174(3):R99–116. 10.1530/eje-15-0262 10.1530/EJE-15-0262 26537862

[29] Corona G, Rastrelli G, Morelli A, Sarchielli E, Cipriani S, Vignozzi L, Maggi M (2020) Treatment of functional hypogonadism besides Pharmacological substitution. World J Men’s Health 38(3):256–270. 10.5534/wjmh.190061 31496147 10.5534/wjmh.190061 PMC7308235

[30] Nyante SJ, Graubard BI, Li Y, McQuillan GM, Platz EA, Rohrmann S, Bradwin G, McGlynn KA (2012) Trends in sex hormone concentrations in US males: 1988–1991 to 1999–2004. Int J Androl 35(3):456–466. 10.1111/j.1365-2605.2011.01230.x 22150314 10.1111/j.1365-2605.2011.01230.x PMC4137971

[31] Wdowiak N, Wójtowicz K, Wdowiak-Filip A, Pucek W, Wróbel A, Wróbel J, Wdowiak A (2024) Environmental Factors as the Main Hormonal Disruptors of Male Fertility. J Clin Med. 10.3390/jcm13071986 38610751 10.3390/jcm13071986 PMC11012640

[32] Santi D, Crépieux P, Reiter E, Spaggiari G, Brigante G, Casarini L, Rochira V, Simoni M (2020) Follicle-stimulating Hormone (FSH) Action on Spermatogenesis: A Focus on Physiological and Therapeutic Roles. J Clin Med. 10.3390/jcm9041014 32260182 10.3390/jcm9041014 PMC7230878

[33] Hughes IP, Choong CS, Cotterill A, Harris M, Davies PS (2010) The influence of secular trend for height on ascertainment and eligibility for growth hormone treatment. Clin Endocrinol 73(6):760–768. 10.1111/j.1365-2265.2010.03874.x 10.1111/j.1365-2265.2010.03874.x 20846295

[34] Juul A, Magnusdottir S, Scheike T, Prytz S, Skakkebaek NE (2007) Age at voice break in Danish boys: effects of pre-pubertal body mass index and secular trend. Int J Androl 30(6):537–542. 10.1111/j.1365-2605.2007.00751.x 17459124 10.1111/j.1365-2605.2007.00751.x

[35] Aksglaede L, Sørensen K, Petersen JH, Skakkebaek NE, Juul A (2009) Recent decline in age at breast development: the Copenhagen puberty study. Pediatrics 123(5):e932–939. 10.1542/peds.2008-2491 19403485 10.1542/peds.2008-2491

[36] Hoyo C, Murtha AP, Schildkraut JM, Jirtle RL, Demark-Wahnefried W, Forman MR, Iversen ES, Kurtzberg J, Overcash F, Huang Z, Murphy SK (2011) Methylation variation at IGF2 differentially methylated regions and maternal folic acid use before and during pregnancy. Epigenetics 6(7):928–936. 10.4161/epi.6.7.16263 21636975 10.4161/epi.6.7.16263 PMC3154433

[37] Ostberg JE, Thomas EL, Hamilton G, Attar MJ, Bell JD, Conway GS (2005) Excess visceral and hepatic adipose tissue in Turner syndrome determined by magnetic resonance imaging: Estrogen deficiency associated with hepatic adipose content. J Clin Endocrinol Metab 90(5):2631–2635. 10.1210/jc.2004-1939 15713713 10.1210/jc.2004-1939

[38] Chen Z, Xu W, Huang Y, Jin X, Deng J, Zhu S, Liu H, Zhang S, Yu Y (2013) Associations of noniodized salt and thyroid nodule among the Chinese population: a large cross-sectional study. Am J Clin Nutr 98(3):684–692. 10.3945/ajcn.112.054353 23842457 10.3945/ajcn.112.054353

[39] Hollowell JG, Staehling NW, Flanders WD, Hannon WH, Gunter EW, Spencer CA, Braverman LE, Serum TSH (2002) T(4), and thyroid antibodies in the united States population (1988 to 1994): National health and nutrition examination survey (NHANES III). J Clin Endocrinol Metab 87(2):489–499. 10.1210/jcem.87.2.8182 11836274 10.1210/jcem.87.2.8182

[40] Huang Y, Cai L, Zheng Y, Pan J, Li L, Zong L, Lin W, Liang J, Huang H, Wen J, Chen G (2019) Association between lifestyle and thyroid dysfunction: a cross-sectional epidemiologic study in the she ethnic minority group of Fujian Province in China. BMC Endocr Disorders 19(1):83. 10.1186/s12902-019-0414-z 10.1186/s12902-019-0414-z PMC6668292 31362731

[41] Dalkin AC, Burger LL, Aylor KW, Haisenleder DJ, Workman LJ, Cho S, Marshall JC (2001) Regulation of gonadotropin subunit gene transcription by gonadotropin-releasing hormone: measurement of primary transcript ribonucleic acids by quantitative reverse transcription-polymerase chain reaction assays. Endocrinology 142(1):139–146. 10.1210/endo.142.1.7881 11145576 10.1210/endo.142.1.7881

[42] Woodruff TJ (2024) Health effects of fossil Fuel-Derived endocrine disruptors. N Engl J Med 390(10):922–933. 10.1056/NEJMra2300476 38446677 10.1056/NEJMra2300476

[43] Corona G, Goulis DG, Huhtaniemi I, Zitzmann M, Toppari J, Forti G, Vanderschueren D, Wu FC (2020) European academy of andrology (EAA) guidelines on investigation, treatment and monitoring of functional hypogonadism in males: endorsing organization: European society of endocrinology. Andrology 8(5):970–987. 10.1111/andr.12770 32026626 10.1111/andr.12770

[44] Corona G, Rastrelli G, Sparano C, Vignozzi L, Sforza A, Maggi M (2024) Advances in the treatment of functional male hypogonadism. Expert Rev Endocrinol Metab 19(2):163–177. 10.1080/17446651.2023.2296022 38117229 10.1080/17446651.2023.2296022

[45] Huhtaniemi IT, Wu FCW (2022) Ageing male (part I): pathophysiology and diagnosis of functional hypogonadism. Best Pract Res Clin Endocrinol Metab 36(4):101622. 10.1016/j.beem.2022.101622 35210191 10.1016/j.beem.2022.101622

[46] Louters M, Pearlman M, Solsrud E, Pearlman A (2022) Functional hypogonadism among patients with obesity, diabetes, and metabolic syndrome. Int J Impot Res 34(7):714–720. 10.1038/s41443-021-00496-7 34775481 10.1038/s41443-021-00496-7

[47] Global fertility (2024) In 204 countries and territories, 1950–2021, with forecasts to 2100: a comprehensive demographic analysis for the global burden of disease study 2021. Lancet. 10.1016/s0140-6736(24)00550-6 10.1016/S0140-6736(24)00550-6 PMC11122687 38521087

