# Supplementary material for: Temporal trends in serum testosterone and luteinizing hormone levels indicate an ongoing resetting of hypothalamic-pituitary-gonadal function in healthy men: a systematic review
Source: J Endocrinol Invest. 2025 Aug 1;48(11):2721–34. doi: 10.1007/s40618-025-02671-9 (PMC12602663; doi:10.1007/s40618-025-02671-9)
Supplement: Supplementary file 1 — Supplementary Material 1 [file 40618_2025_2671_MOESM1_ESM.docx]

**Supplementary material**

**Index**

- Literature search strategy
- Results
  - Meta-regression analysis: body mass index (BMI) and year of blood collection
  - Meta-regression analysis: sex hormone binding globulin (SHBG) and year of blood collection
  - Meta-regression analysis: testosterone and year of blood collection
  - Meta-regression analysis: testosterone and year of blood collection, considering studies with body mass index (BMI) < 25 kg/m^2^
  - Meta-regression analysis: testosterone and year of blood collection, considering year of blood collection before and after 2000
  - Meta-regression analysis: testosterone and year of blood collection, considering only studies published in USA
  - Meta-regression analysis: luteinising hormone (LH) and year of blood collection
  - Meta-regression analysis: follicle-stimulating hormone (FSH) and year of blood collection
  - Meta-regression analysis: testosterone and year of blood collection, dividing studies in groups according to subjects’ age
  - Meta-regression analysis: testosterone and luteinising hormone (LH) serum levels, dividing studies in groups according to subjects’ age.
  - References of studies included in the analysis

**Literature search strategy**

The search strategy applied for the literature search is detailed below:

- "testosterone"[MeSH Terms] OR "testosterone"[All Fields] OR "testosteron"[All Fields] OR "testosterones"[All Fields] OR "testosterone s"[All Fields]
- "androgens"[All Fields] OR "androgene"[All Fields] OR "androgenes"[All Fields] OR "androgenic"[All Fields] OR "androgenicity"[All Fields] OR "androgenized"[All Fields] OR "androgenizing"[All Fields] OR "androgenous"[All Fields] OR "androgens"[Pharmacological Action] OR "androgens"[MeSH Terms] OR "androgens"[All Fields] OR "androgen"[All Fields] OR "virilism"[MeSH Terms] OR "virilism"[All Fields] OR "androgenization"[All Fields]
- "testosterone"[MeSH Terms] OR "testosterone"[All Fields] OR "testosteron"[All Fields] OR "testosterones"[All Fields] OR "testosterone s"[All Fields] OR "androgen s"[All Fields] OR "androgene"[All Fields] OR "androgenes"[All Fields] OR "androgenic"[All Fields] OR "androgenicity"[All Fields] OR "androgenized"[All Fields] OR "androgenizing"[All Fields] OR "androgenous"[All Fields] OR "androgens"[Pharmacological Action] OR "androgens"[MeSH Terms] OR "androgens"[All Fields] OR "androgen"[All Fields] OR "virilism"[MeSH Terms] OR "virilism"[All Fields] OR "androgenization"[All Fields]

**Results**

*Meta-regression analysis: body mass index (BMI) and year of blood collection*

Meta-regression analysis using body mass index (BMI) as dependent variable and the year of blood collection as independent variable.

Mixed-Effects Model (k = 156; tau^2 estimator: REML):

- logLik: -430.0541
- deviance: 860.1081
- AIC: 866.1081
- BIC: 875.2190
- AICc: 866.2681

Results

- tau^2 (estimated amount of residual heterogeneity): 0 (SE = 1.5119)
- tau (square root of estimated tau^2 value): 0
- I^2 (residual heterogeneity / unaccounted variability): 0.00%
- H^2 (unaccounted variability / sampling variability): 1.00
- R^2 (amount of heterogeneity accounted for: 0.00%
- Test for Residual Heterogeneity: QE (df = 154) = 48.9112, p-val = 1.0000
- Test of Moderators (coefficient 2): F (df1 = 1, df2 = 154) = 0.2513, p-val = 0.6169

Model Results:

|  | Estimate | SE | Tval | Df | p-value | ci.lb | ci.ub |
| --- | --- | --- | --- | --- | --- | --- | --- |
| Intercept | 5.75 | 41.85 | 0.14 | 154 | 0.891 | -76.93 | 88.43 |
| Year of blood collection | 0.01 | 0.02 | 0.50 | 154 | 0.617 | -0.03 | 0.05 |

*Meta-regression analysis: sex hormone binding globulin (SHBG) and year of blood collection*

Meta-regression analysis, using sex hormone binding globulin (SHBG) as dependent variable, the year of blood collection as covariate and the subjects’ age and body mass index (BMI) as cofactors.

Mixed-Effects Model (k = 325; tau^2 estimator: REML):

- Loglik: -1278.8987
- Deviance: 2557.7974
- AIC: 2567.7974
- BIC: 2586.6546
- AICc: 2567.9878

Results:

- tau^2 (estimated amount of residual heterogeneity): 65.4136 (SE = 13.8196)
- tau (square root of estimated tau^2 value): 8.0879
- I^2 (residual heterogeneity / unaccounted variability): 68.07%
- H^2 (unaccounted variability / sampling variability): 3.13
- R^2 (amount of heterogeneity accounted for): 0.51%
- Test for Residual Heterogeneity: QE (df = 321) = 2219.3862, p-val < .0001
- Test of Moderators (coefficients 2:4): F (df1 = 3, df2 = 321) = 11.8536, p-val < .0001

Model Results:

|  | Estimate | SE | Tval | Df | p-value | ci.lb | ci.ub |
| --- | --- | --- | --- | --- | --- | --- | --- |
| Intercept | -314.54 | 139.15 | -2.26 | 321 | **0.024** | -588.30 | -40.78 |
| Year of blood collection | 0.17 | 0.07 | 2.47 | 321 | **0.014** | 0.03 | 0.31 |
| BMI | -0.25 | 0.34 | -0.75 | 321 | 0.453 | -0.92 | 0.41 |
| Subjects’ age | 0.22 | 0.04 | 5.20 | 321 | **<0.001** | 0.14 | 0.31 |

**Supplementary Figure 1**. Meta-regression analysis using sex hormone binding globulin (SHBG) as effect of the regression, the year of sample collection as covariates and subjects’ number, age and BMI as co-factors.


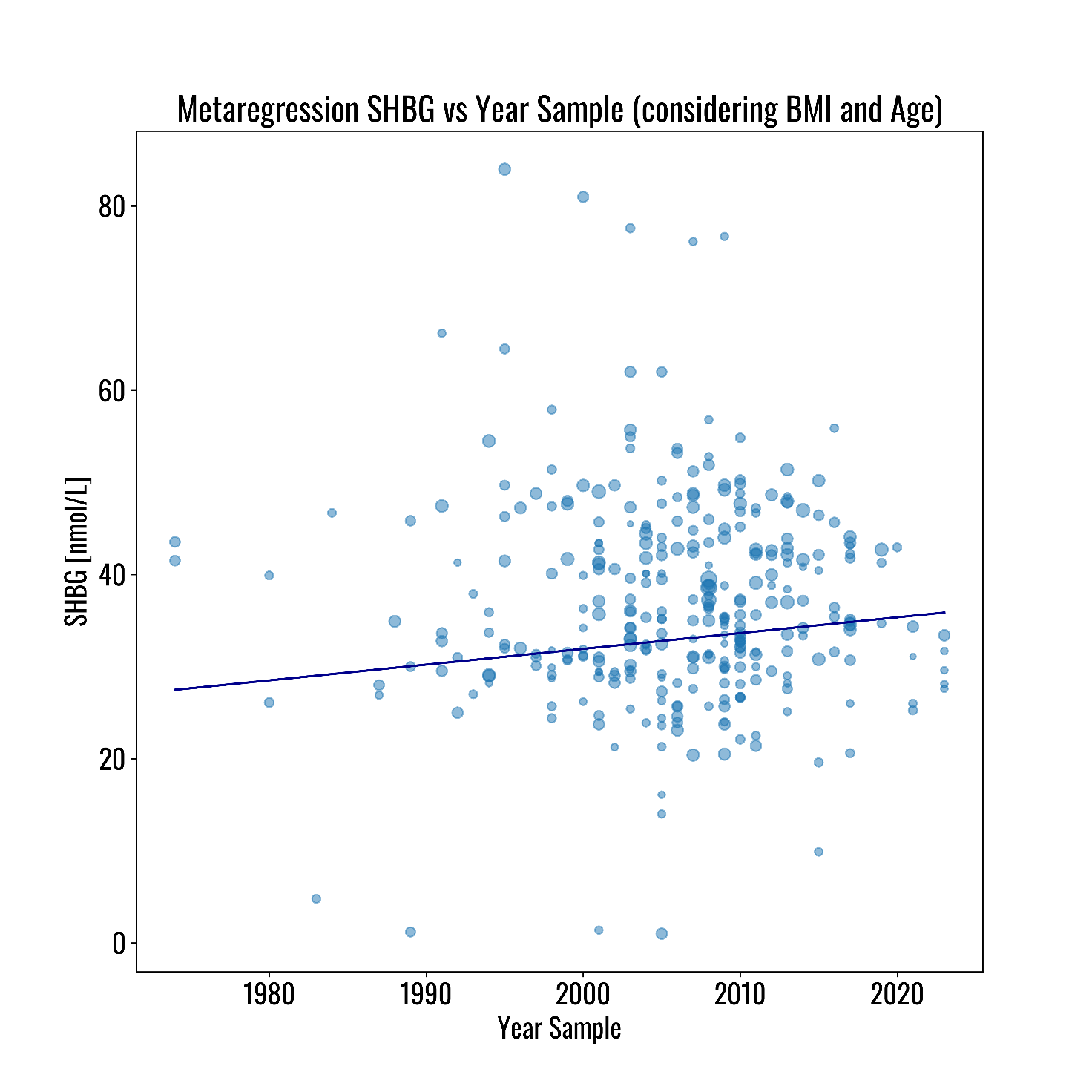


**Comment to figure**: the figure shows the lack of significant trend in sex hormone binding globulin (SHBG) serum levels across year of blood collection. Although an increasing trend is observed, it does not reach the statistical significance.

*Meta-regression analysis: testosterone and year of blood collection*

Meta-regression analysis using total testosterone serum levels as dependent variable, the year of blood collection as covariate and the subjects’ age and body mass index (BMI) as cofactors.

Mixed-Effects Model (k = 745; tau^2 estimator: REML):

- Lolik: -2162.3626
- Deviance: 4324.7253
- AIC: 4334.7253
- BIC: 4357.7653
- AICc: 4334.8069

Results:

- tau^2 (estimated amount of residual heterogeneity): 3.1770 (SE = 0.9599)
- tau (square root of estimated tau^2 value): 1.7824
- I^2 (residual heterogeneity / unaccounted variability): 15.54%
- H^2 (unaccounted variability / sampling variability): 1.18
- R^2 (amount of heterogeneity accounted for): 38.16%
- Test for Residual Heterogeneity: QE (df = 741) = 533.0572, p-val = 1.0000
- Test of Moderators (coefficients 2:4): F (df1 = 3, df2 = 741) = 73.1336, p-val < .0001

Model Results:

|  | Estimate | SE | Tval | Df | p-value | ci.lb | ci.ub |
| --- | --- | --- | --- | --- | --- | --- | --- |
| Intercept | 279.73 | 30.93 | 9.04 | 741 | **<0.001** | 219.01 | 340.45 |
| Year of blood collection | -0.12 | 0.01 | -8.08 | 741 | **<0.001** | -0.15 | -0.09 |
| BMI | -0.33 | 0.06 | -5.73 | 741 | **<0.001** | -0.44 | -0.22 |
| Subjects’ age | -0.08 | 0.01 | -8.36 | 741 | **<0.001** | -0.10 | 0.06 |

*Meta-regression analysis: testosterone and year of blood collection, considering studies with body mass index (BMI) < 25 kg/m^2^*

Meta-regression analysis using total testosterone serum levels as dependent variable, the year of blood collection as covariate and the subjects’ age and body mass index (BMI) as cofactors, considering only studies in which BMI was < 25 kg/m^2^.

Mixed-Effects Model (k = 286; tau^2 estimator: REML):

- Loglik: -855.1922
- Deviance: 1710.3845
- AIC: 1720.3845
- BIC: 1738.5940
- AICc: 1720.6019

Results:

- tau^2 (estimated amount of residual heterogeneity): 6.4416 (SE = 2.1443)
- tau (square root of estimated tau^2 value): 2.5380
- I^2 (residual heterogeneity / unaccounted variability): 26.41%
- H^2 (unaccounted variability / sampling variability): 1.36
- R^2 (amount of heterogeneity accounted for): 16.67%
- Test for Residual Heterogeneity: QE (df = 282) = 283.7030, p-val = 0.4603
- Test of Moderators (coefficients 2:4): F (df1 = 3, df2 = 282) = 14.0126, p-val < .0001

Model Results:

|  | Estimate | SE | Tval | Df | p-value | ci.lb | ci.ub |
| --- | --- | --- | --- | --- | --- | --- | --- |
| Intercept | 350.61 | 66.63 | 5.26 | 282 | **<0.001** | 219.45 | 471.77 |
| Year of blood collection | <0.16 | 0.03 | -4.85 | 282 | **<0.001** | -0.23 | -0.10 |
| BMI | -0.13 | 0.20 | -0.65 | 282 | **0.041** | -0.53 | 0  27 |
| Subjects’ age | -0.07 | 0.02 | -3.75 | 282 | **<0.001** | -0.10 | -0.03 |

*Meta-regression analysis: testosterone and year of blood collection, considering year of blood collection before and after 2000*

Meta-regression analysis using total testosterone serum levels as dependent variable, the year of blood collection as covariates and the subjects’ age as cofactor. The analysis was performed dividing the dataset according to the year of blood collection before and after 2000.

**Pre-2000:**

Mixed-Effects Model (k = 621; tau^2 estimator: REML)

- logLik: -1877.5061
- deviance: 3755.0121
- AIC: 3763.0121
- BIC: 3780.7181
- AICc: 3763.0774

Results:

- tau^2 (estimated amount of residual heterogeneity): 4.9346 (SE = 1.2097)
- tau (square root of estimated tau^2 value): 2.2214
- I^2 (residual heterogeneity / unaccounted variability): 22.49%
- H^2 (unaccounted variability / sampling variability): 1.29
- R^2 (amount of heterogeneity accounted for): 31.45%
- Test for Residual Heterogeneity: QE (df = 618) = 571.1619, p-value = 0.9112
- Test of Moderators (coefficients 2:3): F (df1 = 2, df2 = 618) = 88.9699, p-value < .0001

Model Results:

|  | Estimate | SE | Tval | Df | p-value | ci.lb | ci.ub |
| --- | --- | --- | --- | --- | --- | --- | --- |
| Intercept | 270.64 | 38.61 | 7.01 | 618 | **<0.001** | 194.81 | 346.47 |
| Year | -0.12 | 0.02 | -6.37 | 618 | **<0.001** | -0.16 | -0.09 |
| Subjects’ age | -0.11 | 0.01 | -10.21 | 618 | **<0.001** | -0.13 | -0.09 |

**Post-2000:**

Mixed-Effects Model (k = 880; tau^2 estimator: REML)

- logLik: -2550.0386
- deviance: 5100.0772
- AIC: 5108.0772
- BIC: 5127.1832
- AICc: 5108.1231

Results:

- tau^2 (estimated amount of residual heterogeneity): 3.0750 (SE = 0.8352)
- tau (square root of estimated tau^2 value): 1.7536
- I^2 (residual heterogeneity / unaccounted variability): 15.99%
- H^2 (unaccounted variability / sampling variability): 1.19
- R^2 (amount of heterogeneity accounted for): 33.80%
- Test for Residual Heterogeneity: QE (df = 877) = 635.7633, p-value = 1.0000
- Test of Moderators (coefficients 2:3): F (df1 = 2, df2 = 877) = 112.2286, p-value < .0001

Model Results:

|  | Estimate | SE | Tval | Df | p-value | ci.lb | ci.ub |
| --- | --- | --- | --- | --- | --- | --- | --- |
| Intercept | 341.64 | 41.04 | 8.32 | 877 | **<0.001** | 261.09 | 422.18 |
| Year | -0.16 | 0.02 | -7.82 | 877 | **<0.001** | -0.20 | -0.12 |
| Subjects’ age | -0.10 | 0.01 | -13.62 | 877 | **<0.001** | -0.12 | -0.09 |

*Meta-regression analysis: testosterone and year of blood collection, considering only studies published in USA*

Meta-regression analysis using total testosterone serum levels as dependent variable and the year of blood collection as independent variable, subjects’ age as co-factor, limiting the analysis to studies published in USA.

Mixed-Effects Model (k = 156; tau^2 estimator: REML):

- Lolik: -438.3325
- Deviance: 876.6650
- AIC: 886.6650
- BIC: 901.7844
- AICc: 887.0760

Results:

- tau^2 (estimated amount of residual heterogeneity): 1.8801 (SE = 2.1674)
- tau (square root of estimated tau^2 value): 1.3712
- I^2 (residual heterogeneity / unaccounted variability): 8.04%
- H^2 (unaccounted variability / sampling variability): 1.09
- R^2 (amount of heterogeneity accounted for): 38.85%
- Test for Residual Heterogeneity: QE (df = 152) = 79.9086, p-val = 1.0000
- Test of Moderators (coefficients 2:4): F (df1 = 3, df2 = 152) = 12.9867, p-val < .0001

Model Results:

|  | Estimate | SE | Tval | Df | p-value | ci.lb | ci.ub |
| --- | --- | --- | --- | --- | --- | --- | --- |
| Intercept | 305.92 | 58.12 | 6.26 | 152 | **<0.001** | 191.10 | 420.75 |
| Year of blood collection | -0.14 | 0.03 | -4.89 | 152 | **<0.001** | -0.20 | -0.08 |
| Subjects’ age | -0.08 | 0.02 | -4.65 | 152 | **<0.001** | -0.12 | -0.05 |

**Supplementary Figure 2**. Meta-regression analysis using total testosterone as effect of the regression, the year of sample collection as covariates and subjects’ number and age as co-factors.


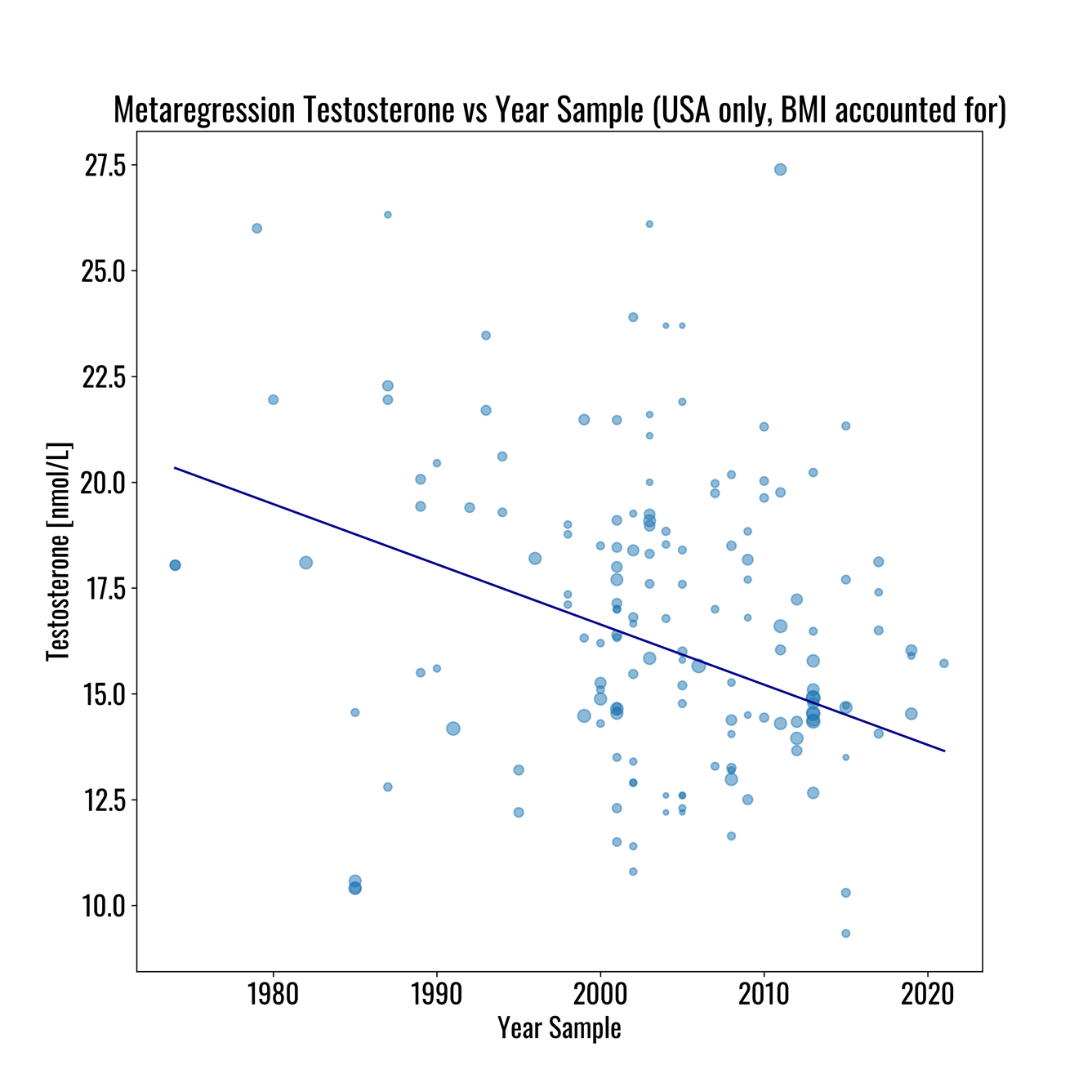


**Comment to figure**: the figure highlights a testosterone declining trend across of year of observation considering only studies published in USA and adjusting data for subjects’ age. The correction of meta-regression analysis for BMI was not performed, since the number of studies would be even more reduced considering studies in which BMI was available only in USA.

*Meta-regression analysis: luteinising hormone (LH) and year of blood collection*

Meta-regression analysis using LH as dependent variable and the year of blood collection as covariate and the subjects’ age as cofactor.

Mixed-Effects Model (k = 481; tau^2 estimator: REML)

- logLik: -1106.2712
- deviance: 2212.5424
- AIC: 2220.5424
- BIC: 2237.2209
- AICc: 2220.6270

Results:

- tau^2 (estimated amount of residual heterogeneity): 2.3261 (SE = 0.3026)
- tau (square root of estimated tau^2 value): 1.5252
- I^2 (residual heterogeneity / unaccounted variability): 78.24%
- H^2 (unaccounted variability / sampling variability): 4.60
- R^2 (amount of heterogeneity accounted for): 2.01%
- Test for Residual Heterogeneity: QE (df = 478) = 1910.5532, p-value < .0001
- Test of Moderators (coefficients 2:3): F (df1 = 2, df2 = 478) = 15.1435, p-value < .0001

Model Results:

|  | Estimate | SE | Tval | Df | p-value | ci.lb | ci.ub |
| --- | --- | --- | --- | --- | --- | --- | --- |
| Intercept | 97.34 | 16.90 | 5.76 | 478 | **<0.001** | 64.13 | 130.55 |
| Year | -0.05 | 0.01 | -5.50 | 478 | **<0.001** | -0.06 | -0.03 |
| Subjects’ age | 0.01 | 0.01 | 0.60 | 478 | 0.551 | -0.01 | 0.02 |

*Meta-regression analysis: follicle-stimulating hormone (FSH) and year of blood collection*

Meta-regression analysis, using follicle-stimulating hormone (FSH) as dependent variable, and the year of blood collection as covariate and the subjects’ age as cofactor.

Mixed-Effects Model (k = 414; tau^2 estimator: REML)

- logLik: -991.1674
- deviance: 1982.3348
- AIC: 1990.3348
- BIC: 2006.4091
- AICc: 1990.4333

Results

tau^2 (estimated amount of residual heterogeneity): 2.1728 (SE = 0.3480)

tau (square root of estimated tau^2 value): 1.4741

I^2 (residual heterogeneity / unaccounted variability): 63.14%

H^2 (unaccounted variability / sampling variability): 2.71

R^2 (amount of heterogeneity accounted for): 19.13%

Test for Residual Heterogeneity: QE (df = 411) = 977.8159, p-value < .0001

Test of Moderators (coefficients 2:3): F (df1 = 2, df2 = 411) = 41.3982, p-value < .0001

Model Results:

|  | Estimate | SE | Tval | Df | p-value | ci.lb | ci.ub |
| --- | --- | --- | --- | --- | --- | --- | --- |
| Intercept | 27.45 | 17.87 | 1.54 | 411 | 0.125 | -7.68 | 62.57 |
| Year | -0.01 | 0.01 | -1.47 | 411 | 0.143 | -0.31 | 0.01 |
| Subjects’ age | 0.09 | 0.01 | 9.06 | 411 | **<0.001** | 0.07 | 0.11 |

**Supplementary Figure 3**. Meta-regression analysis using follicle-stimulating hormone (FSH) as effect of the regression, the year of sample collection as covariates and subjects’ number and age as co-factors.

**
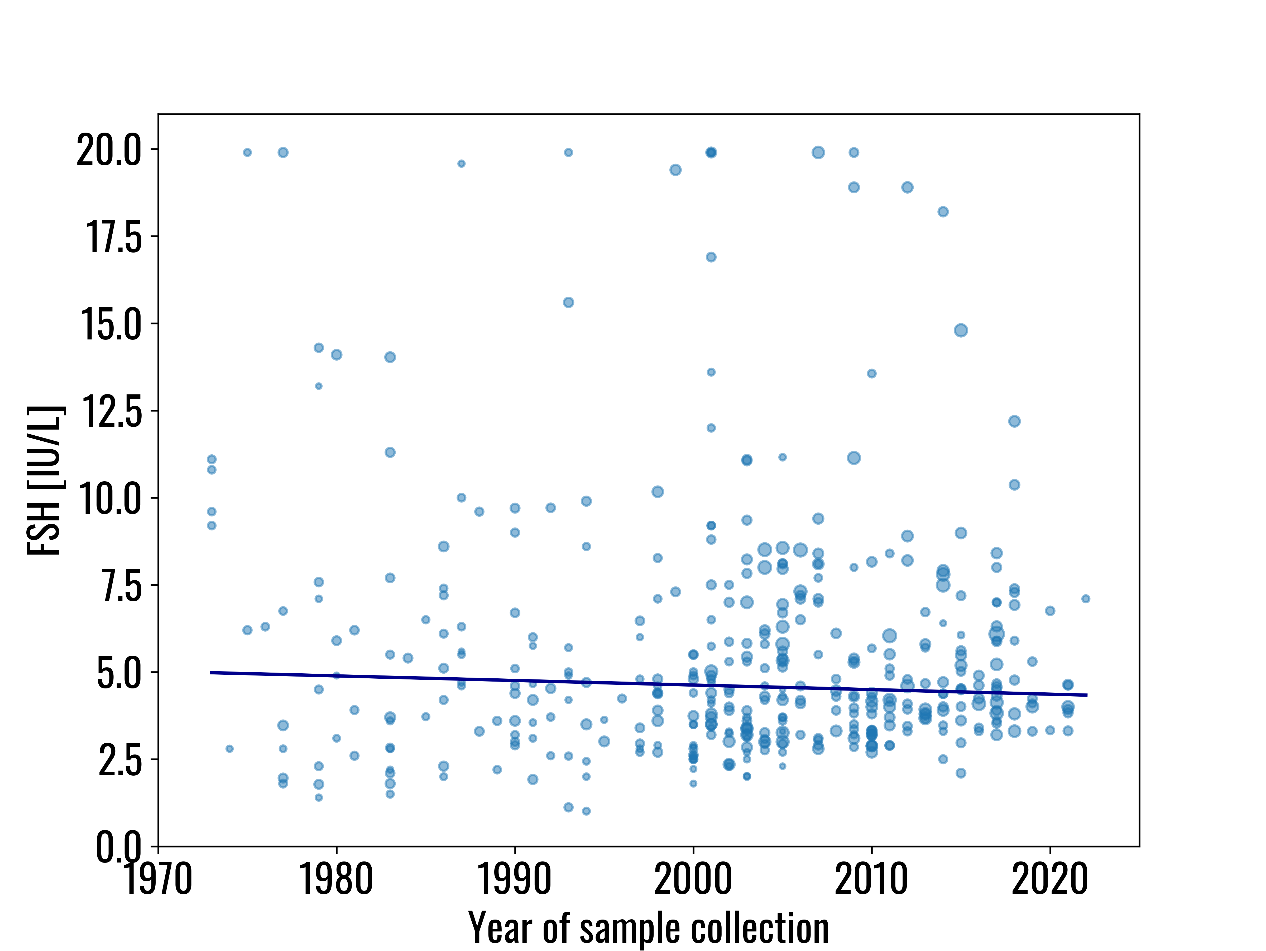
**

**Comment to figure**: the figure shows the lack of significant trend in follicle-stimulating hormone (FSH) serum levels across year of blood collection.

*Meta-regression analysis: testosterone and year of blood collection, dividing studies in groups according to subjects’ age*

Meta-regression analysis using total testosterone serum levels as dependent variable and the year of blood collection as covariates, dividing the entire dataset in different groups, according to subjects’ age.

**18-30 year:**

Mixed-Effects Model (k = 378; tau^2 estimator: REML)

- logLik: -1152.3016
- deviance: 2304.6032
- AIC: 2310.6032
- BIC: 2322.3919
- AICc: 2310.6677

Results:

- tau^2 (estimated amount of residual heterogeneity): 6.1046 (SE = 1.6559)
- tau (square root of estimated tau^2 value): 2.4708
- I^2 (residual heterogeneity / unaccounted variability): 26.86%
- H^2 (unaccounted variability / sampling variability): 1.37
- R^2 (amount of heterogeneity accounted for): 36.60%
- Test for Residual Heterogeneity: QE (df = 376) = 398.2792, p-value = 0.2058
- Test of Moderators (coefficient 2): F (df1 = 1, df2 = 376) = 113.7668, p-value < .0001

Model Results:

estimate se tval df p-value ci.lb ci.ub

intercept 330.2732 29.0600 11.3652 376 <.0001 273.1327 387.4136

x -0.1551 0.0145 -10.6662 376 <.0001 -0.1837 -0.1265

**31-40 year:**

Mixed-Effects Model (k = 414; tau^2 estimator: REML)

- logLik: -1213.4059
- deviance: 2426.8118
- AIC: 2432.8118
- BIC: 2444.8749
- AICc: 2432.8706

Results:

- tau^2 (estimated amount of residual heterogeneity): 3.3324 (SE = 1.2409)
- tau (square root of estimated tau^2 value): 1.8255
- I^2 (residual heterogeneity / unaccounted variability): 16.99%
- H^2 (unaccounted variability / sampling variability): 1.20
- R^2 (amount of heterogeneity accounted for): 23.44%
- Test for Residual Heterogeneity: QE(df = 412) = 319.4542, p-value = 0.9997
- Test of Moderators (coefficient 2): F(df1 = 1, df2 = 412) = 66.9111, p-value < .0001

Model Results:

estimate se tval df p-value ci.lb ci.ub

intercept 234.8047 26.4886 8.8644 412 <.0001 182.7350 286.8745

x -0.1083 0.0132 -8.1799 412 <.0001 -0.1344 -0.0823

**41-50 year:**

Mixed-Effects Model (k = 236; tau^2 estimator: REML)

logLik: -670.8307

deviance: 1341.6614

AIC: 1347.6614

BIC: 1358.0273

AICc: 1347.7657

Results:

- tau^2 (estimated amount of residual heterogeneity): 0.9891 (SE = 1.1118)
- tau (square root of estimated tau^2 value): 0.9945
- I^2 (residual heterogeneity / unaccounted variability): 5.43%
- H^2 (unaccounted variability / sampling variability): 1.06
- R^2 (amount of heterogeneity accounted for): 51.71%
- Test for Residual Heterogeneity: QE(df = 234) = 108.0167, p-value = 1.0000
- Test of Moderators (coefficient 2): F(df1 = 1, df2 = 234) = 65.3098, p-value < .0001

Model Results:

estimate se tval df p-value ci.lb ci.ub

intercept 277.8029 32.2901 8.6034 234 <.0001 214.1865 341.4193

x -0.1304 0.0161 -8.0814 234 <.0001 -0.1621 -0.0986

**51-60 year:**

Mixed-Effects Model (k = 246; tau^2 estimator: REML)

- logLik: -708.6959
- deviance: 1417.3917
- AIC: 1423.3917
- BIC: 1433.8833
- AICc: 1423.4917

Results:

- tau^2 (estimated amount of residual heterogeneity): 4.4113 (SE = 1.9640)
- tau (square root of estimated tau^2 value): 2.1003
- I^2 (residual heterogeneity / unaccounted variability): 20.12%
- H^2 (unaccounted variability / sampling variability): 1.25
- R^2 (amount of heterogeneity accounted for): 25.90%
- Test for Residual Heterogeneity: QE (df = 244) = 201.6946, p-value = 0.9777
- Test of Moderators (coefficient 2): F (df1 = 1, df2 = 244) = 59.8523, p-value < .0001

Model Results:

estimate se tval df p-value ci.lb ci.ub

intercept 360.0128 44.4459 8.1000 244 <.0001 272.4662 447.5594

x -0.1715 0.0222 -7.7364 244 <.0001 -0.2152 -0.1278

**61-70 year:**

Mixed-Effects Model (k = 147; tau^2 estimator: REML)

- logLik: -422.8239
- deviance: 845.6477
- AIC: 851.6477
- BIC: 860.5779
- AICc: 851.8179

Results:

- tau^2 (estimated amount of residual heterogeneity): 1.6647 (SE = 1.6247)
- tau (square root of estimated tau^2 value): 1.2902
- I^2 (residual heterogeneity / unaccounted variability): 9.34%
- H^2 (unaccounted variability / sampling variability): 1.10
- R^2 (amount of heterogeneity accounted for): 18.47%
- Test for Residual Heterogeneity: QE (df = 145) = 100.0805, p-value = 0.9983
- Test of Moderators (coefficient 2): F(df1 = 1, df2 = 145) = 6.4825, p-value = 0.0119

Model Results:

estimate se tval df p-value ci.lb ci.ub

intercept 154.4471 54.7132 2.8228 145 0.0054 46.3086 262.5856

x -0.0695 0.0273 -2.5461 145 0.0119 -0.1235 -0.0156

**>70 year:**

Mixed-Effects Model (k = 75; tau^2 estimator: REML)

- logLik:
- deviance: 431.4184
- AIC: 437.4184
- BIC: 444.2897
- AICc: 437.7662

Results:

- tau^2 (estimated amount of residual heterogeneity): 3.5913 (SE = 3.1163)
- tau (square root of estimated tau^2 value): 1.8951
- I^2 (residual heterogeneity / unaccounted variability): 17.84%
- H^2 (unaccounted variability / sampling variability): 1.22
- R^2 (amount of heterogeneity accounted for): 22.13%
- Test for Residual Heterogeneity: QE (df = 73) = 53.7618, p-value = 0.9556
- Test of Moderators (coefficient 2): F (df1 = 1, df2 = 73) = 9.5202, p-value = 0.0029

Model Results:

estimate se tval df p-value ci.lb ci.ub

intercept 223.5850 67.7130 3.3020 73 0.0015 88.6332 358.5368

x -0.1047 0.0339 -3.0855 73 0.0029 -0.1723 -0.0371


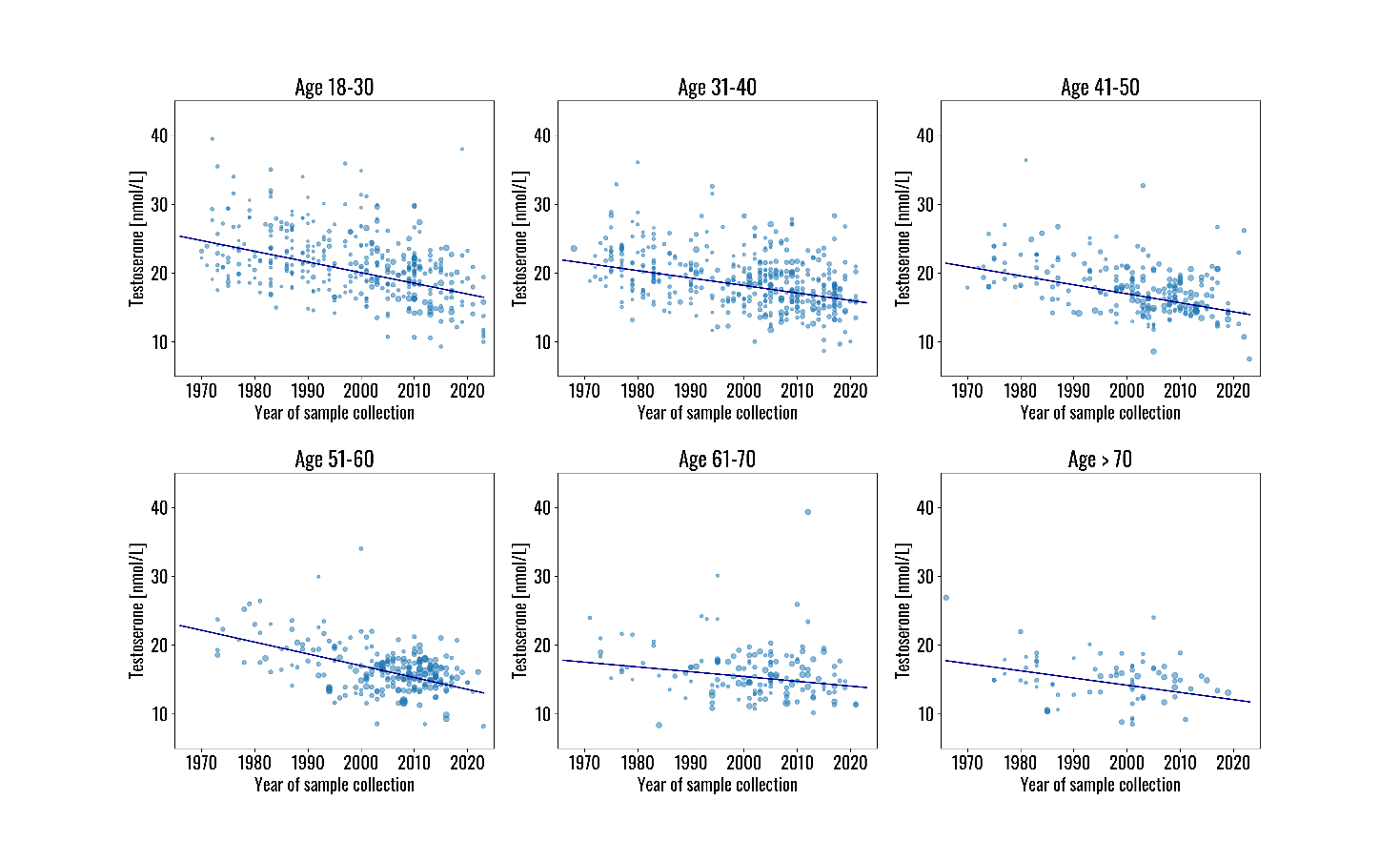


**Comment to figure**: the figure shows that testosterone serum levels maintain significant declining trend across year of observation in each of subgroups generated according to subjects’ age. Body mass index (BMI) was not used to adjust analyses, since it was available in a limited number of studies, reducing significantly analyses’ accuracy.

*Meta-regression analysis: luteinising hormone (LH) and year of blood collection, dividing studies in groups according to subjects’ age*

Meta-regression analysis, using LH serum levels as dependent variable, the year of blood collection, dividing the entire dataset in different groups, according to subjects’ age.

**18-30 year:**

Mixed-Effects Model (k = 146; tau^2 estimator: REML)

- logLik: -328.2860
- deviance: 656.5720
- AIC: 662.5720
- BIC: 671.4815
- AICc: 662.7435

Results:

- tau^2 (estimated amount of residual heterogeneity): 2.4822 (SE = 0.5290)
- tau (square root of estimated tau^2 value): 1.5755
- I^2 (residual heterogeneity / unaccounted variability): 79.30%
- H^2 (unaccounted variability / sampling variability): 4.83
- R^2 (amount of heterogeneity accounted for): 0.00%
- Test for Residual Heterogeneity: QE (df = 144) = 527.3610, p-value < .0001
- Test of Moderators (coefficient 2): F (df1 = 1, df2 = 144) = 4.0861, p-value = 0.0451

Model Results:

estimate se tval df p-value ci.lb ci.ub

intercept 70.8457 32.8306 2.1579 144 **0.0326** 5.9535 135.7378

x -0.0332 0.0164 -2.0214 144 **0.0451** -0.0656 -0.0007

**31-40 year:**

Mixed-Effects Model (k = 185; tau^2 estimator: REML)

- logLik: -420.3487
- deviance: 840.6974
- AIC: 846.6974
- BIC: 856.3259
- AICc: 846.8315

Results:

- tau^2 (estimated amount of residual heterogeneity): 2.5150 (SE = 0.5141)
- tau (square root of estimated tau^2 value): 1.5859
- I^2 (residual heterogeneity / unaccounted variability): 68.28%
- H^2 (unaccounted variability / sampling variability): 3.15
- R^2 (amount of heterogeneity accounted for): 15.71%
- Test for Residual Heterogeneity: QE (df = 183) = 637.6258, p-value < .0001
- Test of Moderators (coefficient 2): F (df1 = 1, df2 = 183) = 30.9236, p-value < .0001

Model Results:

estimate se tval df p-value ci.lb ci.ub

intercept 152.4198 26.6236 5.7250 183 **<.0001** 99.8911 204.9485

x -0.0740 0.0133 -5.5609 183 **<.0001** -0.1002 -0.0477

**41-50 year:**

Mixed-Effects Model (k = 57; tau^2 estimator: REML)

- logLik: -118.4736
- deviance: 236.9472
- AIC: 242.9472
- BIC: 248.9692
- AICc: 243.4178

Results:

- tau^2 (estimated amount of residual heterogeneity): 1.2795 (SE = 0.5446)
- tau (square root of estimated tau^2 value): 1.1312
- I^2 (residual heterogeneity / unaccounted variability): 65.46%
- H^2 (unaccounted variability / sampling variability): 2.90
- R^2 (amount of heterogeneity accounted for): 0.00%
- Test for Residual Heterogeneity: QE (df = 55) = 201.0130, p-value < .0001
- Test of Moderators (coefficient 2): F (df1 = 1, df2 = 55) = 1.3527, p-value = 0.2498

Model Results:

estimate se tval df p-value ci.lb ci.ub

intercept 52.9799 42.0307 1.2605 55 0.2128 -31.2514 137.2113

x -0.0244 0.0210 -1.1631 55 0.2498 -0.0664 0.0176

**51-60 year:**

Mixed-Effects Model (k = 44; tau^2 estimator: REML)

- logLik: -88.2517
- deviance: 176.5033
- AIC: 182.5033
- BIC: 187.7163
- AICc: 183.1349

Results:

- tau^2 (estimated amount of residual heterogeneity): 0 (SE = 0.6224)
- tau (square root of estimated tau^2 value): 0
- I^2 (residual heterogeneity / unaccounted variability): 0.00%
- H^2 (unaccounted variability / sampling variability): 1.00
- R^2 (amount of heterogeneity accounted for): 0.00%
- Test for Residual Heterogeneity: QE (df = 42) = 18.7425, p-value = 0.9993
- Test of Moderators (coefficient 2): F (df1 = 1, df2 = 42) = 0.1394, p-value = 0.7108

Model Results:

estimate se tval df p-value ci.lb ci.ub

intercept -11.9792 44.1693 -0.2712 42 0.7876 -101.1164 77.1581

x 0.0082 0.0220 0.3734 42 0.7108 -0.0363 0.0527

**61-70 year:**

Mixed-Effects Model (k = 24; tau^2 estimator: REML)

- logLik: -56.0965
- deviance: 112.1929
- AIC: 118.1929
- BIC: 121.4661
- AICc: 119.5263

Results:

- tau^2 (estimated amount of residual heterogeneity): 2.0151 (SE = 1.3273)
- tau (square root of estimated tau^2 value): 1.4195
- I^2 (residual heterogeneity / unaccounted variability): 93.03%
- H^2 (unaccounted variability / sampling variability): 14.35
- R^2 (amount of heterogeneity accounted for): 0.00%
- Test for Residual Heterogeneity: QE (df = 22) = 130.3555, p-value < .0001
- Test of Moderators (coefficient 2): F (df1 = 1, df2 = 22) = 0.4697, p-value = 0.5003

Model Results:

estimate se tval df p-value ci.lb ci.ub

intercept 57.0361 78.2276 0.7291 22 0.4736 -105.1981 219.2703

x -0.0268 0.0390 -0.6853 22 0.5003 -0.1077 0.0542

**>70 year:**

Mixed-Effects Model (k = 23; tau^2 estimator: REML)

- logLik: -60.0797
- deviance: 120.1595
- AIC: 126.1595
- BIC: 129.2931
- AICc: 127.5713

Results:

- tau^2 (estimated amount of residual heterogeneity): 1.5983 (SE = 2.2404)
- tau (square root of estimated tau^2 value): 1.2642
- I^2 (residual heterogeneity / unaccounted variability): 19.52%
- H^2 (unaccounted variability / sampling variability): 1.24
- R^2 (amount of heterogeneity accounted for): 57.30%
- Test for Residual Heterogeneity: QE (df = 21) = 14.8064, p-value = 0.8325
- Test of Moderators (coefficient 2): F (df1 = 1, df2 = 21) = 11.6085, p-value = 0.0027

Model Results:

estimate se tval df p-value ci.lb ci.ub

intercept -310.3292 92.6436 -3.3497 21 **0.0030** -502.9922 -117.6662

x 0.1583 0.0465 3.4071 21 **0.0027** 0.0617 0.2549

*References of studies included in the analysis*

*[1-1256]*

1. Global, regional, and national burden of stroke and its risk factors, 1990-2019: a systematic analysis for the Global Burden of Disease Study 2019. Lancet Neurol **20**(10), 795-820 (2021). doi:10.1111/andr.13081

10.1016/s1474-4422(21)00252-0

2. Aafjes, J.H., van der Vijver, J.C., Docter, R., Schenck, P.E.: Serum gonadotrophins, testosterone and spermatogenesis in subfertile men. Acta Endocrinol (Copenh) **86**(3), 651-658 (1977). doi:10.1530/acta.0.0860651

3. Abbatecola, A.M., Ferrucci, L., Ceda, G., Russo, C.R., Lauretani, F., Bandinelli, S., Barbieri, M., Valenti, G., Paolisso, G.: Insulin resistance and muscle strength in older persons. J Gerontol A Biol Sci Med Sci **60**(10), 1278-1282 (2005). doi:10.1093/gerona/60.10.1278

4. Abdel Hamid, F.F., Soliman, A.F., Lashin, F.E.S., Yao, F.J., Zhang, Y.D., Wan, Z., Li, W., Lin, H., Deng, C.H., Zhang, Y.: Circulating 25-hydroxy vitamin D correlates with serum level of anti-Müllerian hormone in male patients with chronic kidney disease

Erectile dysfunction is associated with subclinical carotid vascular disease in young men lacking widely-known risk factors. Andrologia **20**(4), 400-404 (2018). doi:10.1111/and.12972

10.4103/aja.aja_73_17

5. Abid, S., Maitra, A., Meherji, P., Patel, Z., Kadam, S., Shah, J., Shah, R., Kulkarni, V., Baburao, V., Gokral, J.: Clinical and laboratory evaluation of idiopathic male infertility in a secondary referral center in India. J Clin Lab Anal **22**(1), 29-38 (2008). doi:10.1002/jcla.20216

6. Acar, D., Cayan, S., Bozlu, M., Akbay, E.: Is routine hormonal measurement necessary in initial evaluation of men with erectile dysfunction? Arch Androl **50**(4), 247-253 (2004). doi:10.1080/01485010490448769

7. Adler, L., Wedekind, D., Pilz, J., Weniger, G., Huether, G.: Endocrine correlates of personality traits: a comparison between emotionally stable and emotionally labile healthy young men. Neuropsychobiology **35**(4), 205-210 (1997). doi:10.1159/000119346

8. Adoamnei, E., Mendiola, J., Moñino-García, M., Vela-Soria, F., Iribarne-Durán, L.M., Fernández, M.F., Olea, N., Jørgensen, N., Swan, S.H., Torres-Cantero, A.M.: Urinary concentrations of benzophenone-type ultra violet light filters and reproductive parameters in young men. Int J Hyg Environ Health **221**(3), 531-540 (2018). doi:10.4103/aja.aja_73_17

10.1016/j.ijheh.2018.02.002

9. Adoamnei, E., Mendiola, J., Vela-Soria, F., Fernández, M.F., Olea, N., Jørgensen, N., Swan, S.H., Torres-Cantero, A.M.: Urinary bisphenol A concentrations are associated with reproductive parameters in young men. Environ Res **161**, 122-128 (2018). doi:10.1016/j.envres.2017.11.002

10. Agha-Mohammadhasani, P., Mokhtaree, M., Nazari, A., Rahnama, A., Lysenko, E.A., Popov, D.V., Vepkhvadze, T.F., Sharova, A.P., Vinogradova, O.L.: Comparison of Sexual Function and Serum Testosterone Levels in Men Opiate Addicts, under Methadone Maintenance Therapy, and Healthy Men

Signaling responses to high and moderate load strength exercise in trained muscle. Addict Health **10**(2), 76-85 (2018). doi:10.22122/ahj.v10i2.540

10.14814/phy2.14100

11. Agledahl, I., Skjaerpe, P.A., Hansen, J.B., Svartberg, J.: Low serum testosterone in men is inversely associated with non-fasting serum triglycerides: the Tromsø study. Nutr Metab Cardiovasc Dis **18**(4), 256-262 (2008). doi:10.1016/j.numecd.2007.01.014

12. Agledahl, I., Skjaerpe, P.A., Hansen, J.B., Svartberg, J.: Low serum testosterone in men is inversely associated with non-fasting serum triglycerides: the TromsÃ¸ study. Nutr Metab Cardiovasc Dis **18**(4), 256-262 (2008). doi:10.1016/j.numecd.2007.01.014

13. Aguilar, C.E., Soliman, A.S., McConnell, D.S., Zekri, A.R., Banerjee, M., Omar, A., Sharawy, M., Omar, S., Raouf, A., Sowers, M.R.: Androgen profiles among Egyptian adults considering liver status. J Gastroenterol Hepatol **23**(7 Pt 2), e137-145 (2008). doi:10.1111/j.1440-1746.2007.04949.x

14. Ahmadi, H., Allameh, F., Baradaran, N., Montaser-Kouhsari, L., Bazargan-Hejazi, S., Salem, S., Mehrsai, A., Pourmand, G.: Circulating sex hormones play no role in the association between sexual activity and the risk of prostate cancer. J Sex Med **8**(3), 905-913 (2011). doi:10.1111/j.1743-6109.2010.02115.x

15. Ahokas, E.K., Ihalainen, J.K., Kyröläinen, H., Mero, A.A.: Effects of Water Immersion Methods on Postexercise Recovery of Physical and Mental Performance. J Strength Cond Res **33**(6), 1488-1495 (2019). doi:10.1519/jsc.0000000000003134

16. Ahtiainen, J.P., Hulmi, J.J., Kraemer, W.J., Lehti, M., Nyman, K., Selänne, H., Alen, M., Pakarinen, A., Komulainen, J., Kovanen, V., Mero, A.A., Häkkinen, K.: Heavy resistance exercise training and skeletal muscle androgen receptor expression in younger and older men. Steroids **76**(1-2), 183-192 (2011). doi:10.1016/j.steroids.2010.10.012

17. Aizawa, H., Niimura, M.: Serum hormone levels in men with severe acne. J Dermatol **19**(7), 404-407 (1992). doi:10.1111/j.1346-8138.1992.tb03249.x

18. AkbaÅŸ, T., Karakurt, S., UnlÃ¼gÃ¼zel, G., Celikel, T., Akalin, S.: The endocrinologic changes in critically ill chronic obstructive pulmonary disease patients. Copd **7**(4), 240-247 (2010). doi:10.3109/15412555.2010.496815

19. Akbaş, T., Karakurt, S., Unlügüzel, G., Celikel, T., Akalin, S.: The endocrinologic changes in critically ill chronic obstructive pulmonary disease patients. Copd **7**(4), 240-247 (2010). doi:10.3109/15412555.2010.496815

20. Akehi, Y., Tanabe, M., Yano, H., Takashi, Y., Kawanami, D., Nomiyama, T., Yanase, T.: A simple questionnaire for the detection of testosterone deficiency in men with late-onset hypogonadism. Endocrine journal **69**(11), 1303-1312 (2022). doi:10.1507/endocrj.EJ22-0073

21. Akinloye, O., Blessing Popoola, B., Bolanle Ajadi, M., Gregory Uchechukwu, J., Pius Oparinde, D.: Hypogonadism and metabolic syndrome in nigerian male patients with both type 2 diabetes and hypertension. Int J Endocrinol Metab **12**(1), e10749 (2014). doi:10.5812/ijem.10749

22. Akishita, M., Fukai, S., Hashimoto, M., Kameyama, Y., Nomura, K., Nakamura, T., Ogawa, S., Iijima, K., Eto, M., Ouchi, Y.: Association of low testosterone with metabolic syndrome and its components in middle-aged Japanese men. Hypertens Res **33**(6), 587-591 (2010). doi:10.1038/hr.2010.43

23. Akishita, M., Hashimoto, M., Ohike, Y., Ogawa, S., Iijima, K., Eto, M., Ouchi, Y.: Low testosterone level as a predictor of cardiovascular events in Japanese men with coronary risk factors. Atherosclerosis **210**(1), 232-236 (2010). doi:10.1016/j.atherosclerosis.2009.10.037

24. Akl, E.M., Salah, A.A.: Effect of new oral direct acting antiviral therapy on sexual function in male patients with hepatitis C virus. Andrologia **52**(11), e13835 (2020). doi:10.1111/and.13835

25. Aksglaede, L., Olesen, I.A., Carlsen, E., Petersen, J.H., Juul, A., JÃ¸rgensen, N.: Serum concentration of anti-MÃ¼llerian hormone is not associated with semen quality. Andrology **6**(2), 286-292 (2018). doi:10.1111/andr.12456

26. Aksglaede, L., Olesen, I.A., Carlsen, E., Petersen, J.H., Juul, A., Jørgensen, N.: Serum concentration of anti-Müllerian hormone is not associated with semen quality. Andrology **6**(2), 286-292 (2018). doi:10.1111/andr.12456

27. Al-Eisa, E., Alghadir, A.H., Gabr, S.A., Iqbal, Z.A.: Exercise intervention as a protective modulator against metabolic disorders in cigarette smokers. J Phys Ther Sci **28**(3), 983-991 (2016). doi:10.1589/jpts.28.983

28. Alexander, G.M., Swerdloff, R.S., Wang, C., Davidson, T., McDonald, V., Steiner, B., Hines, M.: Androgen-behavior correlations in hypogonadal men and eugonadal men. II. Cognitive abilities. Horm Behav **33**(2), 85-94 (1998). doi:10.1006/hbeh.1998.1439

29. Ali Hamza, M., Abdulhameed, A., Ali Mansour, A.: Total Testosterone to Estradiol Ratio as a Predictor Marker of Metabolic Syndrome in Males. Arch Razi Inst **77**(1), 351-357 (2022). doi:10.22092/ari.2021.356607.1878

30. Allen, N.E., Appleby, P.N., Davey, G.K., Key, T.J.: Lifestyle and nutritional determinants of bioavailable androgens and related hormones in British men. Cancer Causes Control **13**(4), 353-363 (2002). doi:10.1023/a:1015238102830

31. Al-Nawd, E.A., Alshowafi, F.K., Abdullateef, A.A., Noman, M.M.A., Albadani, R.H., Al-Mansoub, M.A.: Serum leptin correlates in fertile and idiopathic infertile Yemeni males: a comparative cross-sectional study. Syst Biol Reprod Med **68**(5-6), 348-356 (2022). doi:10.1080/19396368.2022.2113930

32. Aluja, A., García, L.F., García, Ó., Blanco, E.: Testosterone and disinhibited personality in healthy males. Physiol Behav **164**(Pt A), 227-232 (2016). doi:10.1016/j.physbeh.2016.06.007

33. Amatruda, J.M., Harman, S.M., Pourmotabbed, G., Lockwood, D.H.: Depressed plasma testosterone and fractional binding of testosterone in obese males. J Clin Endocrinol Metab **47**(2), 268-271 (1978). doi:10.1210/jcem-47-2-268

34. Ambrosi, B., Bara, R., Travaglini, P., Weber, G., Beck Peccoz, P., Rondena, M., Elli, R., Faglia, G.: Study of the effects of bromocriptine on sexual impotence. Clin Endocrinol (Oxf) **7**(5), 417-421 (1977). doi:10.1111/j.1365-2265.1977.tb03351.x

35. Ambroży, T., Rydzik, Ł., Obmiński, Z., Błach, W., Serafin, N., Błach, B., Jaszczur-Nowicki, J., Ozimek, M.: The Effect of High-Intensity Interval Training Periods on Morning Serum Testosterone and Cortisol Levels and Physical Fitness in Men Aged 35-40 Years. J Clin Med **10**(10) (2021). doi:10.3390/jcm10102143

36. Amjad, S., Baig, M., Zahid, N., Tariq, S., Rehman, R.: Association between leptin, obesity, hormonal interplay and male infertility. Andrologia **51**(1), e13147 (2019). doi:10.1111/and.13147

37. Amory, J.K., Page, S.T., Anawalt, B.D., Coviello, A.D., Matsumoto, A.M., Bremner, W.J.: Elevated end-of-treatment serum INSL3 is associated with failure to completely suppress spermatogenesis in men receiving male hormonal contraception. J Androl **28**(4), 548-554 (2007). doi:10.2164/jandrol.106.002345

38. Amory, J.K., Page, S.T., Bremner, W.J.: Oral testosterone in oil: pharmacokinetic effects of 5alpha reduction by finasteride or dutasteride and food intake in men. J Androl **27**(1), 72-78 (2006). doi:10.2164/jandrol.05058

39. Anand-Ivell, R., Heng, K., Severn, K., Antonio, L., Bartfai, G., Casanueva, F.F., Huhtaniemi, I.T., Giwercman, A., Maggi, M., O'Neill, T.W., Punab, M., Rastrelli, G., Slowikowska-Hilczer, J., Tournoy, J., Vanderschueren, D., Wu, F.C.W., Ivell, R.: Association of age, hormonal, and lifestyle factors with the Leydig cell biomarker INSL3 in aging men from the European Male Aging Study cohort. Andrology **10**(7), 1328-1338 (2022). doi:10.1111/andr.13220

40. Anapliotou, M.L., Liparaki, M., Americanos, N., Goulandris, N., Papaioannou, D.: Increased 17-OH-progesterone levels following hCG stimulation in men with idiopathic oligozoospermia and raised FSH levels. Int J Androl **17**(4), 192-198 (1994). doi:10.1111/j.1365-2605.1994.tb01242.x

41. Andersen, A.N., Semczuk, M., Tabor, A.: Prolactin and pituitary-gonadal function in cigarette smoking infertile patients. Andrologia **16**(5), 391-396 (1984). doi:10.1111/j.1439-0272.1984.tb00381.x

42. Andersen, J.M., Herning, H., Witczak, O., Haugen, T.B.: Anti-Müllerian hormone in seminal plasma and serum: association with sperm count and sperm motility. Hum Reprod **31**(8), 1662-1667 (2016). doi:10.1093/humrep/dew121

43. Anderson, K.E., Rosner, W., Khan, M.S., New, M.I., Pang, S.Y., Wissel, P.S., Kappas, A.: Diet-hormone interactions: protein/carbohydrate ratio alters reciprocally the plasma levels of testosterone and cortisol and their respective binding globulins in man. Life Sci **40**(18), 1761-1768 (1987). doi:10.1016/0024-3205(87)90086-5

44. Anderson, R.A., Cormier, J., Thieroff-Ekerdt, R., Boyce, M., van den Berg, F., Grau, D., Turnquist, D., Corzo, D., Graham, P.: Pharmacodynamic Activity of the Novel Neurokinin-3 Receptor Antagonist SJX-653 in Healthy Men. J Clin Endocrinol Metab **105**(12), e4857-4865 (2020). doi:10.1210/clinem/dgaa657

45. Anderson, R.A., Kinniburgh, D., Baird, D.T.: Suppression of spermatogenesis by etonogestrel implants with depot testosterone: potential for long-acting male contraception. J Clin Endocrinol Metab **87**(8), 3640-3649 (2002). doi:10.1210/jcem.87.8.8773

46. Andersson, B., MÃ¥rin, P., Lissner, L., Vermeulen, A., BjÃ¶rntorp, P.: Testosterone concentrations in women and men with NIDDM. Diabetes Care **17**(5), 405-411 (1994). doi:10.2337/diacare.17.5.405

47. Andersson, B., Mårin, P., Lissner, L., Vermeulen, A., Björntorp, P.: Testosterone concentrations in women and men with NIDDM. Diabetes Care **17**(5), 405-411 (1994). doi:10.2337/diacare.17.5.405

48. Andò, S., Giacchetto, C., Colpi, G., Panno, M.L., Beraldi, E., Lombardi, A., Sposato, G.: Plasma levels of 17-OH-progesterone and testosterone in patients with varicoceles. Acta Endocrinol (Copenh) **102**(3), 463-469 (1983). doi:10.1530/acta.0.1020463

49. Andò, S., Rubens, R., Rottiers, R.: Androgen plasma levels in male diabetics. J Endocrinol Invest **7**(1), 21-24 (1984). doi:10.1007/bf03348370

50. Andrade, S.R.L., Mucida, Y.M., Xavier, J.D.C., Fernandes, L.N., Silva, R.O., Bandeira, F.: Bone mineral density, trabecular bone score and muscle strength in transgender men receiving testosterone therapy versus cisgender men. Steroids **178**, 108951 (2022). doi:10.1016/j.steroids.2021.108951

51. Ansari, M.A., Begum, D., Islam, F.: Serum sex steroids, gonadotrophins and sex hormone-binding globulin in prostatic hyperplasia. Ann Saudi Med **28**(3), 174-178 (2008). doi:10.5144/0256-4947.2008.174

52. Antonio, L., Priskorn, L., Nordkap, L., Bang, A.K., Jensen, T.K., Skakkebaek, N.E., Petersen, J.H., Vanderschueren, D., Jørgensen, N.: Bone mineral density is preserved in men with idiopathic infertility. Andrology **8**(2), 315-322 (2020). doi:10.1111/andr.12688

53. Antonio, L., Wu, F.C.W., Moors, H., MatheÃ¯, C., Huhtaniemi, I.T., Rastrelli, G., Dejaeger, M., O'Neill, T.W., Pye, S.R., Forti, G., Maggi, M., Casanueva, F.F., Slowikowska-Hilczer, J., Punab, M., Tournoy, J., Vanderschueren, D.: Erectile dysfunction predicts mortality in middle-aged and older men independent of their sex steroid status. Age Ageing **51**(4) (2022). doi:10.1093/ageing/afac094

54. Antonio, L., Wu, F.C.W., Moors, H., Matheï, C., Huhtaniemi, I.T., Rastrelli, G., Dejaeger, M., O'Neill, T.W., Pye, S.R., Forti, G., Maggi, M., Casanueva, F.F., Slowikowska-Hilczer, J., Punab, M., Tournoy, J., Vanderschueren, D.: Erectile dysfunction predicts mortality in middle-aged and older men independent of their sex steroid status. Age Ageing **51**(4) (2022). doi:10.1093/ageing/afac094

55. Appiah, D., Luitel, S., Nwabuo, C.C., Ebong, I., Winters, S.J.: Low endogenous estradiol levels are associated with elevated risk of cardiovascular disease mortality in young and middle-aged men in the United States. Atherosclerosis **361**, 34-40 (2022). doi:10.1016/j.atherosclerosis.2022.09.006

56. Araujo, A.B., Travison, T.G., Leder, B.Z., McKinlay, J.B.: Correlations between serum testosterone, estradiol, and sex hormone-binding globulin and bone mineral density in a diverse sample of men. The Journal of clinical endocrinology and metabolism **93**(6), 2135-2141 (2008). doi:10.1210/jc.2007-1469

57. Aref, A.T., Vincent, A.D., O'Callaghan, M.E., Martin, S.A., Sutherland, P.D., Hoy, A.J., Butler, L.M., Wittert, G.A.: The inverse relationship between prostate specific antigen (PSA) and obesity. Endocr Relat Cancer **25**(11), 933-941 (2018). doi:10.1530/erc-17-0438

58. Arias-Santiago, S., Gutiérrez-Salmerón, M.T., Castellote-Caballero, L., Buendía-Eisman, A., Naranjo-Sintes, R.: Androgenetic alopecia and cardiovascular risk factors in men and women: a comparative study. J Am Acad Dermatol **63**(3), 420-429 (2010). doi:10.1016/j.jaad.2009.10.018

59. Arnlöv, J., Pencina, M.J., Amin, S., Nam, B.H., Benjamin, E.J., Murabito, J.M., Wang, T.J., Knapp, P.E., D'Agostino, R.B., Sr., Bhasin, S., Vasan, R.S.: Endogenous sex hormones and cardiovascular disease incidence in men. Ann Intern Med **145**(3), 176-184 (2006). doi:10.7326/0003-4819-145-3-200608010-00005

60. Arrabal-Polo, M., Arias-Santiago, S., López-Carmona Pintado, F., Merino-Salas, S., Lahoz-García, C., Zuluaga-Gómez, A., Arrabal-Martin, M.: Metabolic syndrome, hormone levels, and inflammation in patients with erectile dysfunction. ScientificWorldJournal **2012**, 272769 (2012). doi:10.1100/2012/272769

61. Asare-Anane, H., Ofori, E., Agyemang, Y., Oppong, S., Tagoe, E., Bani, S., Ateku, R., Bawa, T.: Obesity and Testosterone Levels in Ghanaian Men With Type 2 Diabetes. Clin Diabetes **32**(2), 61-65 (2014). doi:10.2337/diaclin.32.2.61

62. Asselmann, E., Kische, H., Haring, R., Hertel, J., Schmidt, C.O., Nauck, M., Beesdo-Baum, K., Grabe, H.J., Pané-Farré, C.A.: Prospective associations of androgens and sex hormone-binding globulin with 12-month, lifetime and incident anxiety and depressive disorders in men and women from the general population. J Affect Disord **245**, 905-911 (2019). doi:10.1016/j.jad.2018.11.052

63. Attia, A.A.A., Amer, M., Hassan, M., Din, S.F.G.: Low serum folic acid can be a potential independent risk factor for erectile dysfunction: a prospective case-control study. Int Urol Nephrol **51**(2), 223-229 (2019). doi:10.1007/s11255-018-2055-y

64. Auyeung, T.W., Kwok, T., Leung, J., Lee, J.S., Ohlsson, C., Vandenput, L., Wing, Y.K., Woo, J.: Sleep Duration and Disturbances Were Associated With Testosterone Level, Muscle Mass, and Muscle Strength--A Cross-Sectional Study in 1274 Older Men. J Am Med Dir Assoc **16**(7), 630.e631-636 (2015). doi:10.1016/j.jamda.2015.04.006

65. Averbeck, M.A., Colares, C., de Lira, G.H., Selbach, T., Rhoden, E.L.: Evaluation of endothelial function with brachial artery ultrasound in men with or without erectile dysfunction and classified as intermediate risk according to the Framingham Score. J Sex Med **9**(3), 849-856 (2012). doi:10.1111/j.1743-6109.2011.02591.x

66. Awad, H., Halawa, F., Mostafa, T., Atta, H.: Melatonin hormone profile in infertile males. Int J Androl **29**(3), 409-413 (2006). doi:10.1111/j.1365-2605.2005.00624.x

67. Ayers, J.W., Komesu, Y., Romani, T., Ansbacher, R.: Anthropomorphic, hormonal, and psychologic correlates of semen quality in endurance-trained male athletes. Fertil Steril **43**(6), 917-921 (1985). doi:10.1016/s0015-0282(16)48622-x

68. Baccarelli, A., Morpurgo, P.S., Corsi, A., Vaghi, I., Fanelli, M., Cremonesi, G., Vaninetti, S., Beck-Peccoz, P., Spada, A.: Activin A serum levels and aging of the pituitary-gonadal axis: a cross-sectional study in middle-aged and elderly healthy subjects. Exp Gerontol **36**(8), 1403-1412 (2001). doi:10.1016/s0531-5565(01)00117-6

69. Bachman, E., Feng, R., Travison, T., Li, M., Olbina, G., Ostland, V., Ulloor, J., Zhang, A., Basaria, S., Ganz, T., Westerman, M., Bhasin, S.: Testosterone suppresses hepcidin in men: a potential mechanism for testosterone-induced erythrocytosis. J Clin Endocrinol Metab **95**(10), 4743-4747 (2010). doi:10.1210/jc.2010-0864

70. Bagatell, C.J., Bremner, W.J.: Sperm counts and reproductive hormones in male marathoners and lean controls. Fertil Steril **53**(4), 688-692 (1990). doi:10.1016/s0015-0282(16)53465-7

71. Banerjee, C., Ulloor, J., Dillon, E.L., Dahodwala, Q., Franklin, B., Storer, T., Sebastiani, P., Sheffield-Moore, M., Urban, R.J., Bhasin, S., Montano, M.: Identification of serum biomarkers for aging and anabolic response. Immun Ageing **8**(1), 5 (2011). doi:10.1186/1742-4933-8-5

72. Barberia, J.M., Giner, J., Cortes-Gallegos, V.: Diurnal variations of plasma testosterone in men. Steroids **22**(5), 615-626 (1973). doi:10.1016/0039-128x(73)90110-4

73. BarregÃ¥rd, L., Lindstedt, G., SchÃ¼tz, A., SÃ¤llsten, G.: Endocrine function in mercury exposed chloralkali workers. Occup Environ Med **51**(8), 536-540 (1994). doi:10.1136/oem.51.8.536

74. Barregård, L., Lindstedt, G., Schütz, A., Sällsten, G.: Endocrine function in mercury exposed chloralkali workers. Occup Environ Med **51**(8), 536-540 (1994). doi:10.1136/oem.51.8.536

75. Barth, J.D., Jansen, H., Hugenholtz, P.G., Birkenhäger, J.C.: Post-heparin lipases, lipids and related hormones in men undergoing coronary arteriography to assess atherosclerosis. Atherosclerosis **48**(3), 235-241 (1983). doi:10.1016/0021-9150(83)90041-2

76. Barut, O., Seyithanoglu, M., Kucukdurmaz, F., Demir, B.T., Olmez, C., Dogan, N.T., Resim, S.: Relationship between the G protein-coupled oestrogen receptor and spermatogenesis, and its correlation with male infertility. Andrologia **52**(10), e13779 (2020). doi:10.1111/and.13779

77. Basu, M., Pal, K., Prasad, R., Malhotra, A.S., Rao, K.S., Sawhney, R.C.: Pituitary, gonadal and adrenal hormones after prolonged residence at extreme altitude in man. Int J Androl **20**(3), 153-158 (1997). doi:10.1046/j.1365-2605.1997.00046.x

78. Bataille, V., Perret, B., Evans, A., Amouyel, P., Arveiler, D., DucimetiÃ¨re, P., Bard, J.M., FerriÃ¨res, J.: Sex hormone-binding globulin is a major determinant of the lipid profile: the PRIME study. Atherosclerosis **179**(2), 369-373 (2005). doi:10.1016/j.atherosclerosis.2004.10.029

79. Bataille, V., Perret, B., Evans, A., Amouyel, P., Arveiler, D., Ducimetière, P., Bard, J.M., Ferrières, J.: Sex hormone-binding globulin is a major determinant of the lipid profile: the PRIME study. Atherosclerosis **179**(2), 369-373 (2005). doi:10.1016/j.atherosclerosis.2004.10.029

80. Bauer, J., Stoffel-Wagner, B., FlÃ¼gel, D., Kluge, M., Schramm, J., Bidlingmaier, F., Elger, C.E.: Serum androgens return to normal after temporal lobe epilepsy surgery in men. Neurology **55**(6), 820-824 (2000). doi:10.1212/wnl.55.6.820

81. Bauer, J., Stoffel-Wagner, B., Flügel, D., Kluge, M., Schramm, J., Bidlingmaier, F., Elger, C.E.: Serum androgens return to normal after temporal lobe epilepsy surgery in men. Neurology **55**(6), 820-824 (2000). doi:10.1212/wnl.55.6.820

82. Bauman, W.A., La Fountaine, M.F., Cirnigliaro, C.M., Kirshblum, S.C., Spungen, A.M.: Provocative stimulation of the hypothalamic-pituitary-testicular axis in men with spinal cord injury. Spinal Cord **54**(11), 961-966 (2016). doi:10.1038/sc.2016.50

83. Bawor, M., Dennis, B.B., Samaan, M.C., Plater, C., Worster, A., Varenbut, M., Daiter, J., Marsh, D.C., Desai, D., Steiner, M., Anglin, R., Coote, M., Pare, G., Thabane, L., Samaan, Z.: Methadone induces testosterone suppression in patients with opioid addiction. Sci Rep **4**, 6189 (2014). doi:10.1038/srep06189

84. Behre, H.M., Simoni, M., Nieschlag, E.: Strong association between serum levels of leptin and testosterone in men. Clin Endocrinol (Oxf) **47**(2), 237-240 (1997). doi:10.1046/j.1365-2265.1997.2681067.x

85. Beitins, I.Z., Bayard, F., Kowarski, A., Migeon, C.J.: The effect of ACTH administration on plasma testosterone, dihydrotestosterone and serum LH concentrations in normal men. Steroids **21**(4), 553-564 (1973). doi:10.1016/0039-128x(73)90044-5

86. Bejerot, S., Eriksson, J.M., Bonde, S., Carlström, K., Humble, M.B., Eriksson, E.: The extreme male brain revisited: gender coherence in adults with autism spectrum disorder. Br J Psychiatry **201**, 116-123 (2012). doi:10.1192/bjp.bp.111.097899

87. Bélanger, A., Locong, A., Noel, C., Cusan, L., Dupont, A., Prévost, J., Caron, S., Sévigny, J.: Influence of diet on plasma steroids and sex hormone-binding globulin levels in adult men. J Steroid Biochem **32**(6), 829-833 (1989). doi:10.1016/0022-4731(89)90459-7

88. BelavÃ½, D.L., Seibel, M.J., Roth, H.J., Armbrecht, G., Rittweger, J., Felsenberg, D.: The effects of bed-rest and countermeasure exercise on the endocrine system in male adults: evidence for immobilization-induced reduction in sex hormone-binding globulin levels. J Endocrinol Invest **35**(1), 54-62 (2012). doi:10.3275/7606

89. Belavý, D.L., Seibel, M.J., Roth, H.J., Armbrecht, G., Rittweger, J., Felsenberg, D.: The effects of bed-rest and countermeasure exercise on the endocrine system in male adults: evidence for immobilization-induced reduction in sex hormone-binding globulin levels. J Endocrinol Invest **35**(1), 54-62 (2012). doi:10.3275/7606

90. Belgorosky, A., Escobar, M.E., Rivarola, M.A.: Validity of the calculation of non-sex hormone-binding globulin-bound estradiol from total testosterone, total estradiol and sex hormone-binding globulin concentrations in human serum. J Steroid Biochem **28**(4), 429-432 (1987). doi:10.1016/0022-4731(87)91061-2

91. Bellastella, G., Maiorino, M.I., Olita, L., Capuano, A., Rafaniello, C., Giugliano, D., Esposito, K.: Vitamin D deficiency in type 2 diabetic patients with hypogonadism. J Sex Med **11**(2), 536-542 (2014). doi:10.1111/jsm.12384

92. Bellastella, G., Pane, E., Iorio, S., De Bellis, A., Sinisi, A.A.: Seasonal variations of plasma gonadotropin, prolactin, and testosterone levels in primary and secondary hypogonadism: evidence for an independent testicular role. J Endocrinol Invest **36**(5), 339-342 (2013). doi:10.3275/8620

93. Belli, S., Santi, D., Leoni, E., Dall'Olio, E., Fanelli, F., Mezzullo, M., Pelusi, C., Roli, L., Tagliavini, S., Trenti, T., Granata, A.R., Pagotto, U., Pasquali, R., Rochira, V., Carani, C., Simoni, M.: Human chorionic gonadotropin stimulation gives evidence of differences in testicular steroidogenesis in Klinefelter syndrome, as assessed by liquid chromatography-tandem mass spectrometry. Eur J Endocrinol **174**(6), 801-811 (2016). doi:10.1530/eje-15-1224

94. Belva, F., Roelants, M., De Schepper, J., Van Steirteghem, A., Tournaye, H., Bonduelle, M.: Reproductive hormones of ICSI-conceived young adult men: the first results. Human reproduction (Oxford, England) **32**(2), 439-446 (2017). doi:10.1093/humrep/dew324

95. Bercea, R.M., Mihaescu, T., Cojocaru, C., Bjorvatn, B.: Fatigue and serum testosterone in obstructive sleep apnea patients. Clin Respir J **9**(3), 342-349 (2015). doi:10.1111/crj.12150

96. Berchtold, P., Berger, M., Cüppers, H.J., Herrmann, J., Nieschlag, E., Rudorff, K., Zimmermann, H., Krüskemper, H.L.: Non-glucoregulatory hormones (T4, T3, rT3, TSH, testosterone) during physical exercise in juvenile type diabetics. Horm Metab Res **10**(4), 269-273 (1978). doi:10.1055/s-0028-1093412

97. Bercovici, J.P., Mauvais-Jarvis, P.: Hyperthyroidism and gynecomastia: metabolic studies. J Clin Endocrinol Metab **35**(5), 671-677 (1972). doi:10.1210/jcem-35-5-671

98. Bercovici, J.P., Nahoul, K., Tater, D., Charles, J.F., Scholler, R.: Hormonal profile of Leydig cell tumors with gynecomastia. J Clin Endocrinol Metab **59**(4), 625-630 (1984). doi:10.1210/jcem-59-4-625

99. Bergeron, M.F., Maresh, C.M., Kraemer, W.J., Abraham, A., Conroy, B., Gabaree, C.: Tennis: a physiological profile during match play. Int J Sports Med **12**(5), 474-479 (1991). doi:10.1055/s-2007-1024716

100. Berglund, L.H., Prytz, H.S., Perski, A., Svartberg, J.: Testosterone levels and psychological health status in men from a general population: the Tromsø study. Aging Male **14**(1), 37-41 (2011). doi:10.3109/13685538.2010.522276

101. Berglund, L.H., Prytz, H.S., Perski, A., Svartberg, J.: Testosterone levels and psychological health status in men from a general population: the TromsÃ¸ study. Aging Male **14**(1), 37-41 (2011). doi:10.3109/13685538.2010.522276

102. Berkseth, K.E., Rubinow, K.B., Melhorn, S.J., Webb, M.F., Rosalynn, B.D.L.M., Marck, B.T., Matsumoto, A.M., Amory, J.K., Page, S.T., Schur, E.A.: Hypothalamic Gliosis by MRI and Visceral Fat Mass Negatively Correlate with Plasma Testosterone Concentrations in Healthy Men. Obesity (Silver Spring) **26**(12), 1898-1904 (2018). doi:10.1002/oby.22324

103. Beshir, S., Ibrahim, K.S., Shaheen, W., Shahy, E.M.: Hormonal Perturbations in Occupationally Exposed Nickel Workers. Open Access Maced J Med Sci **4**(2), 307-311 (2016). doi:10.3889/oamjms.2016.046

104. Besiroglu, H., Otunctemur, A., Dursun, M., Ozbek, E.: The prevalence and severity of varicocele in adult population over the age of forty years old: a cross-sectional study. Aging Male **22**(3), 207-213 (2019). doi:10.1080/13685538.2018.1465913

105. Betts, J.A., Beelen, M., Stokes, K.A., Saris, W.H., van Loon, L.J.: Endocrine responses during overnight recovery from exercise: impact of nutrition and relationships with muscle protein synthesis. Int J Sport Nutr Exerc Metab **21**(5), 398-409 (2011). doi:10.1123/ijsnem.21.5.398

106. Beutel, M.E., Wiltink, J., Hauck, E.W., Auch, D., Behre, H.M., BrÃ¤hler, E., Weidner, W.: Correlations between hormones, physical, and affective parameters in aging urologic outpatients. Eur Urol **47**(6), 749-755 (2005). doi:10.1016/j.eururo.2005.02.015

107. Beutel, M.E., Wiltink, J., Hauck, E.W., Auch, D., Behre, H.M., Brähler, E., Weidner, W.: Correlations between hormones, physical, and affective parameters in aging urologic outpatients. Eur Urol **47**(6), 749-755 (2005). doi:10.1016/j.eururo.2005.02.015

108. Bhasin, S., Woodhouse, L., Casaburi, R., Singh, A.B., Mac, R.P., Lee, M., Yarasheski, K.E., Sinha-Hikim, I., Dzekov, C., Dzekov, J., Magliano, L., Storer, T.W.: Older men are as responsive as young men to the anabolic effects of graded doses of testosterone on the skeletal muscle. J Clin Endocrinol Metab **90**(2), 678-688 (2005). doi:10.1210/jc.2004-1184

109. Bilha, S.C., Branisteanu, D., Buzduga, C., Constantinescu, D., Cianga, P., Anisie, E., Covic, A., Ungureanu, M.C.: Body composition and circulating estradiol are the main bone density predictors in healthy young and middle-aged men. J Endocrinol Invest **41**(8), 995-1003 (2018). doi:10.1007/s40618-018-0826-z

110. Biolchi, V., Silva Neto, B., Pianta, D.B., Koff, W.J., Berger, M., Brum, I.S.: Androgen receptor GGC polymorphism and testosterone levels associated with high risk of prostate cancer and benign prostatic hyperplasia. Mol Biol Rep **40**(3), 2749-2756 (2013). doi:10.1007/s11033-012-2293-5

111. Bird, S.P., Mabon, T., Pryde, M., Feebrey, S., Cannon, J.: Triphasic multinutrient supplementation during acute resistance exercise improves session volume load and reduces muscle damage in strength-trained athletes. Nutr Res **33**(5), 376-387 (2013). doi:10.1016/j.nutres.2013.03.002

112. Birk, L., Williams, G.H., Chasin, M., Rose, L.I.: Serum testosterone levels in homosexual men. N Engl J Med **289**(23), 1236-1238 (1973). doi:10.1056/nejm197312062892308

113. Birzniece, V., Sata, A., Sutanto, S., Ho, K.K.: Neuroendocrine regulation of growth hormone and androgen axes by selective estrogen receptor modulators in healthy men. J Clin Endocrinol Metab **95**(12), 5443-5448 (2010). doi:10.1210/jc.2010-1477

114. Birzniece, V., Sutanto, S., Ho, K.K.: Gender difference in the neuroendocrine regulation of growth hormone axis by selective estrogen receptor modulators. J Clin Endocrinol Metab **97**(4), E521-527 (2012). doi:10.1210/jc.2011-3347

115. Biswas, M., Hampton, D., Turkes, A., Newcombe, R.G., Aled Rees, D.: Reduced total testosterone concentrations in young healthy South Asian men are partly explained by increased insulin resistance but not by altered adiposity. Clin Endocrinol (Oxf) **73**(4), 457-462 (2010). doi:10.1111/j.1365-2265.2010.03824.x

116. Blanco-MuÃ±oz, J., LacasaÃ±a, M., Aguilar-GarduÃ±o, C.: Effect of current tobacco consumption on the male reproductive hormone profile. Sci Total Environ **426**, 100-105 (2012). doi:10.1016/j.scitotenv.2012.03.071

117. Blanco-Muñoz, J., Lacasaña, M., Aguilar-Garduño, C.: Effect of current tobacco consumption on the male reproductive hormone profile. Sci Total Environ **426**, 100-105 (2012). doi:10.1016/j.scitotenv.2012.03.071

118. Bliesener, N., Albrecht, S., Schwager, A., Weckbecker, K., Lichtermann, D., KlingmÃ¼ller, D.: Plasma testosterone and sexual function in men receiving buprenorphine maintenance for opioid dependence. J Clin Endocrinol Metab **90**(1), 203-206 (2005). doi:10.1210/jc.2004-0929

119. Bliesener, N., Albrecht, S., Schwager, A., Weckbecker, K., Lichtermann, D., Klingmüller, D.: Plasma testosterone and sexual function in men receiving buprenorphine maintenance for opioid dependence. J Clin Endocrinol Metab **90**(1), 203-206 (2005). doi:10.1210/jc.2004-0929

120. Bobjer, J., Bogefors, K., Isaksson, S., Leijonhufvud, I., Akesson, K., Giwercman, Y.L., Giwercman, A.: High prevalence of hypogonadism and associated impaired metabolic and bone mineral status in subfertile men. Clinical endocrinology **85**(2), 189-195 (2016). doi:10.1111/cen.13038

121. Bobjer, J., Katrinaki, M., Dermitzaki, E., Margioris, A.N., Giwercman, A., Tsatsanis, C.: Serum chemerin levels are negatively associated with male fertility and reproductive hormones. Hum Reprod **33**(12), 2168-2174 (2018). doi:10.1093/humrep/dey310

122. Bodenheimer, S., Winter, J.S., Faiman, C.: Diurnal rhythms of serum gonadotropins, testosterone, estradiol and cortisol in blind men. J Clin Endocrinol Metab **37**(3), 472-475 (1973). doi:10.1210/jcem-37-3-472

123. Boeri, L., Capogrosso, P., Ventimiglia, E., Cazzaniga, W., Pederzoli, F., Moretti, D., Dehò, F., Montanari, E., Montorsi, F., Salonia, A.: Does Calculated Free Testosterone Overcome Total Testosterone in Protecting From Sexual Symptom Impairment? Findings of a Cross-Sectional Study. J Sex Med **14**(12), 1549-1557 (2017). doi:10.1016/j.jsxm.2017.10.070

124. Bojesen, A., Birkebæk, N., Kristensen, K., Heickendorff, L., Mosekilde, L., Christiansen, J.S., Gravholt, C.H.: Bone mineral density in Klinefelter syndrome is reduced and primarily determined by muscle strength and resorptive markers, but not directly by testosterone. Osteoporos Int **22**(5), 1441-1450 (2011). doi:10.1007/s00198-010-1354-7

125. Bojesen, A., Kristensen, K., Birkebaek, N.H., Fedder, J., Mosekilde, L., Bennett, P., Laurberg, P., Frystyk, J., Flyvbjerg, A., Christiansen, J.S., Gravholt, C.H.: The metabolic syndrome is frequent in Klinefelter's syndrome and is associated with abdominal obesity and hypogonadism. Diabetes care **29**(7), 1591-1598 (2006). doi:10.2337/dc06-0145

126. Bonithon-Kopp, C., Scarabin, P.Y., Bara, L., Castanier, M., Jacqueson, A., Roger, M.: Relationship between sex hormones and haemostatic factors in healthy middle-aged men. Atherosclerosis **71**(1), 71-76 (1988). doi:10.1016/0021-9150(88)90303-6

127. Boonen, S., Vanderschueren, D., Cheng, X.G., Verbeke, G., Dequeker, J., Geusens, P., Broos, P., Bouillon, R.: Age-related (type II) femoral neck osteoporosis in men: biochemical evidence for both hypovitaminosis D- and androgen deficiency-induced bone resorption. J Bone Miner Res **12**(12), 2119-2126 (1997). doi:10.1359/jbmr.1997.12.12.2119

128. Booth, J.D., Merriam, G.R., Clark, R.V., Loriaux, D.L., Sherins, R.J.: Evidence for Leydig cell dysfunction in infertile men with a selective increase in plasma follicle-stimulating hormone. J Clin Endocrinol Metab **64**(6), 1194-1198 (1987). doi:10.1210/jcem-64-6-1194

129. Bosco, C., Iacovelli, M., Tsarpela, O., Cardinale, M., Bonifazi, M., Tihanyi, J., Viru, M., De Lorenzo, A., Viru, A.: Hormonal responses to whole-body vibration in men. Eur J Appl Physiol **81**(6), 449-454 (2000). doi:10.1007/s004210050067

130. Boucher, D., Grizard, G., Hermabessiere, J., Gaillard, G., Pays, J.: Pituitary gonadal function in infertile men with varicocele. Andrologia **15**(1), 78-89 (1983). doi:10.1111/j.1439-0272.1983.tb00120.x

131. Boyar, R.M., Rosenfeld, R.S., Kapen, S., Finkelstein, J.W., Roffwarg, H.P., Weitzman, E.D., Hellman, L.: Human puberty. Simultaneous augmented secretion of luteinizing hormone and testosterone during sleep. J Clin Invest **54**(3), 609-618 (1974). doi:10.1172/jci107798

132. Boyle, W.J., Compton, A.A., Rigg, G., Menon, K.M., McCann, D.S.: Serum testosterone measurements. Am J Clin Pathol **81**(6), 754-761 (1984). doi:10.1093/ajcp/81.6.754

133. Bozic, B., Loncar, G., Prodanovic, N., Radojicic, Z., Cvorovic, V., Dimkovic, S., Popovic-Brkic, V.: Relationship between high circulating adiponectin with bone mineral density and bone metabolism in elderly males with chronic heart failure. J Card Fail **16**(4), 301-307 (2010). doi:10.1016/j.cardfail.2009.12.015

134. Brady, B.M., Amory, J.K., Perheentupa, A., Zitzmann, M., Hay, C.J., Apter, D., Anderson, R.A., Bremner, W.J., Pollanen, P., Nieschlag, E., Wu, F.C., Kersemaekers, W.M.: A multicentre study investigating subcutaneous etonogestrel implants with injectable testosterone decanoate as a potential long-acting male contraceptive. Hum Reprod **21**(1), 285-294 (2006). doi:10.1093/humrep/dei300

135. Brady, B.M., Walton, M., Hollow, N., Kicman, A.T., Baird, D.T., Anderson, R.A.: Depot testosterone with etonogestrel implants result in induction of azoospermia in all men for long-term contraception. Hum Reprod **19**(11), 2658-2667 (2004). doi:10.1093/humrep/deh491

136. Braga-Basaria, M., Dobs, A.S., Muller, D.C., Carducci, M.A., John, M., Egan, J., Basaria, S.: Metabolic syndrome in men with prostate cancer undergoing long-term androgen-deprivation therapy. J Clin Oncol **24**(24), 3979-3983 (2006). doi:10.1200/jco.2006.05.9741

137. Braga-Basaria, M., Muller, D.C., Carducci, M.A., Dobs, A.S., Basaria, S.: Lipoprotein profile in men with prostate cancer undergoing androgen deprivation therapy. Int J Impot Res **18**(5), 494-498 (2006). doi:10.1038/sj.ijir.3901471

138. Brand, J.S., Wareham, N.J., Dowsett, M., Folkerd, E., van der Schouw, Y.T., Luben, R.N., Khaw, K.T.: Associations of endogenous testosterone and SHBG with glycated haemoglobin in middle-aged and older men. Clin Endocrinol (Oxf) **74**(5), 572-578 (2011). doi:10.1111/j.1365-2265.2010.03951.x

139. Breuer, B., Trungold, S., Martucci, C., Wallenstein, S., Likourezos, A., Libow, L.S., Zumoff, B.: Relationships of sex hormone levels to dependence in activities of daily living in the frail elderly. Maturitas **39**(2), 147-159 (2001). doi:10.1016/s0378-5122(01)00208-0

140. Brodie, H.K., Gartrell, N., Doering, C., Rhue, T.: Plasma testosterone levels in heterosexual and homosexual men. Am J Psychiatry **131**(1), 82-83 (1974). doi:10.1176/ajp.131.1.82

141. Brown, T.R., Bullock, L.P., Bardin, C.W.: The biological actions and metabolism of 6 alpha-methylprogesterone: a progestin that mimics and modifies the effects of testosterone. Endocrinology **109**(6), 1814-1820 (1981). doi:10.1210/endo-109-6-1814

142. Brownlee, K.K., Moore, A.W., Hackney, A.C.: Relationship between circulating cortisol and testosterone: influence of physical exercise. J Sports Sci Med **4**(1), 76-83 (2005).

143. Bruno, B., Francavilla, S., Properzi, G., Martini, M., Fabbrini, A.: Hormonal and seminal parameters in infertile men. Andrologia **18**(6), 595-600 (1986). doi:10.1111/j.1439-0272.1986.tb01837.x

144. Buckman, M.T., Peake, G.T., Srivastava, S.: Indomethacin fails to alter basal or phenothiazine-induced prolactin concentrations in man. Horm Metab Res **11**(6), 395-398 (1979). doi:10.1055/s-0028-1092747

145. Buettner, R., Bollheimer, L.C., Zietz, B., Drobnik, W., Lackner, K., Schmitz, G., SchÃ¶lmerich, J., Palitzsch, K.D.: Definition and characterization of relative hypo- and hyperleptinemia in a large Caucasian population. J Endocrinol **175**(3), 745-756 (2002). doi:10.1677/joe.0.1750745

146. Buettner, R., Bollheimer, L.C., Zietz, B., Drobnik, W., Lackner, K., Schmitz, G., Schölmerich, J., Palitzsch, K.D.: Definition and characterization of relative hypo- and hyperleptinemia in a large Caucasian population. J Endocrinol **175**(3), 745-756 (2002). doi:10.1677/joe.0.1750745

147. Buhrich, N., Theile, H., Yaw, A., Crawford, A.: Plasma testosterone, serum FSH, and serum LH levels in transvestism. Arch Sex Behav **8**(1), 49-53 (1979). doi:10.1007/bf01541212

148. Bujan, L., Mieusset, R., Audran, F., Lumbroso, S., Sultan, C.: Increased oestradiol level in seminal plasma in infertile men. Hum Reprod **8**(1), 74-77 (1993). doi:10.1093/oxfordjournals.humrep.a137878

149. Bulut, S.D., Bulut, S., Atalan, D.G., Tulaci, R.G., Türker, T., Gürçay, E., Aydemir, Ç.: The Effect of Antipsychotics on Bone Mineral Density and Sex Hormones in Male Patients with Schizophrenia. Psychiatr Danub **28**(3), 255-262 (2016).

150. Bunt, J.C., Bahr, J.M., Bemben, D.A.: Comparison of estradiol and testosterone levels during and immediately following prolonged exercise in moderately active and trained males and females. Endocr Res **13**(2), 157-172 (1987). doi:10.3109/07435808709023670

151. Burnett-Bowie, S.M., Mendoza, N., Leder, B.Z.: Effects of gonadal steroid withdrawal on serum phosphate and FGF-23 levels in men. Bone **40**(4), 913-918 (2007). doi:10.1016/j.bone.2006.10.016

152. Burris, A.S., Banks, S.M., Carter, C.S., Davidson, J.M., Sherins, R.J.: A long-term, prospective study of the physiologic and behavioral effects of hormone replacement in untreated hypogonadal men. J Androl **13**(4), 297-304 (1992).

153. Cabral, R.D., Busin, L., Rosito, T.E., Koff, W.J.: Performance of Massachusetts Male Aging Study (MMAS) and androgen deficiency in the aging male (ADAM) questionnaires in the prediction of free testosterone in patients aged 40 years or older treated in outpatient regimen. Aging Male **17**(3), 147-154 (2014). doi:10.3109/13685538.2014.908460

154. Calbet, J.A.L., Ponce-González, J.G., Calle-Herrero, J., Perez-Suarez, I., Martin-Rincon, M., Santana, A., Morales-Alamo, D., Holmberg, H.C.: Exercise Preserves Lean Mass and Performance during Severe Energy Deficit: The Role of Exercise Volume and Dietary Protein Content. Front Physiol **8**, 483 (2017). doi:10.1016/j.jsxm.2017.06.015

10.3389/fphys.2017.00483

155. Calogero, A.E., La Vignera, S., Condorelli, R.A., Perdichizzi, A., Valenti, D., Asero, P., Carbone, U., Boggia, B., De Rosa, N., Lombardi, G., D'Agata, R., Vicari, L.O., Vicari, E., De Rosa, M.: Environmental car exhaust pollution damages human sperm chromatin and DNA. J Endocrinol Invest **34**(6), e139-143 (2011). doi:10.1007/bf03346722

156. Cameron, J.L., Weltzin, T.E., McConaha, C., Helmreich, D.L., Kaye, W.H.: Slowing of pulsatile luteinizing hormone secretion in men after forty-eight hours of fasting. J Clin Endocrinol Metab **73**(1), 35-41 (1991). doi:10.1210/jcem-73-1-35

157. Campbell, B., Gray, P.B., Leslie, P.: Age-related changes in body composition among Turkana males of Kenya. Am J Hum Biol **17**(5), 601-610 (2005). doi:10.1002/ajhb.20422

158. Can, O., Özbir, S., Atalay, H.A., Çakır, S.S., Culha, M.G., Canat, H.L.: The relationship between testosterone levels and Peyronie's disease. Andrologia **52**(9), e13727 (2020). doi:10.1111/and.13727

159. Cannarella, R., Condorelli, R.A., Dall'Oglio, F., La Vignera, S., Mongioì, L.M., Micali, G., Calogero, A.E., Lima, T.F.N., Patel, P., Blachman-Braun, R., Madhusoodanan, V., Ramasamy, R.: Increased DHEAS and Decreased Total Testosterone Serum Levels in a Subset of Men with Early-Onset Androgenetic Alopecia: Does a Male PCOS-Equivalent Exist?

Serum 17-Hydroxyprogesterone is a Potential Biomarker for Evaluating Intratesticular Testosterone. Int J Endocrinol **2020**(3), 1942126 (2020). doi:10.1155/2020/1942126

10.1097/ju.0000000000001016

160. Canpolat, U., Tokgözoğlu, L., Aydin, K., Dural, M., Gürses, K.M., Yorgun, H., Canpolat, A.G., Kaya, E.B., Kabakçi, G., Usman, A., Oto, A., Aytemir, K.: Impaired aortic elastic properties in patients with adult-onset hypogonadism. Blood Press **22**(2), 114-119 (2013). doi:10.3109/08037051.2012.732777

161. Cao, W., Xu, Y., Shen, Y., Wang, Y., Ma, X., Bao, Y.: Associations between sex hormones and metabolic-associated fatty liver disease in a middle-aged and elderly community. Endocr J **69**(8), 1007-1014 (2022). doi:10.1507/endocrj.EJ21-0559

162. Carani, C., Granata, A.R., Fustini, M.F., Marrama, P.: Prolactin and testosterone: their role in male sexual function. Int J Androl **19**(1), 48-54 (1996). doi:10.1111/j.1365-2605.1996.tb00434.x

163. Carlström, K., Eriksson, A., Stege, R., Rannevik, G.: Relationship between serum testosterone and sex hormone-binding globulin in adult men with intact or absent gonadal function. Int J Androl **13**(1), 67-73 (1990). doi:10.1111/j.1365-2605.1990.tb00961.x

164. Caron, P.J., Bennet, A.P., Plantavid, M.M., Louvet, J.P.: Luteinizing hormone secretory pattern before and after removal of Leydig cell tumor of the testis. Eur J Endocrinol **131**(2), 156-159 (1994). doi:10.1530/eje.0.1310156

165. Carter, J.R., Durocher, J.J., Larson, R.A., DellaValla, J.P., Yang, H.: Sympathetic neural responses to 24-hour sleep deprivation in humans: sex differences. Am J Physiol Heart Circ Physiol **302**(10), H1991-1997 (2012). doi:10.1152/ajpheart.01132.2011

166. Catford, S.R., Halliday, J., Lewis, S., O'Bryan, M.K., Handelsman, D.J., Hart, R.J., McBain, J., Rombauts, L., Amor, D.J., Saffery, R., McLachlan, R.I.: Reproductive function in men conceived with in vitro fertilization and intracytoplasmic sperm injection. Fertil Steril **117**(4), 727-737 (2022). doi:10.1016/j.fertnstert.2021.12.026

167. Cauley, J.A., Gutai, J.P., Kuller, L.H., Dai, W.S.: Usefulness of sex steroid hormone levels in predicting coronary artery disease in men. Am J Cardiol **60**(10), 771-777 (1987). doi:10.1016/0002-9149(87)91021-6

168. Celani, M.F., Montanini, V., Baraghini, G.F., Carani, C., Marrama, P.: Effects of acute stimulation with gonadotropin releasing hormone (GnRH) on biologically active serum luteinizing hormone (LH) in elderly men. J Endocrinol Invest **7**(6), 589-595 (1984). doi:10.1007/bf03349491

169. Celik-Ozenci, C., Tasatargil, A., Tekcan, M., Sati, L., Gungor, E., Isbir, M., Usta, M.F., Akar, M.E., Erler, F.: Effect of abamectin exposure on semen parameters indicative of reduced sperm maturity: a study on farmworkers in Antalya (Turkey). Andrologia **44**(6), 388-395 (2012). doi:10.1111/j.1439-0272.2012.01297.x

170. Cetin, A., GÃ¶kÃ§e-Kutsal, Y., Celiker, R.: Predictors of bone mineral density in healthy males. Rheumatol Int **21**(3), 85-88 (2001). doi:10.1007/s00296-001-0142-2

171. Cetin, A., Gökçe-Kutsal, Y., Celiker, R.: Predictors of bone mineral density in healthy males. Rheumatol Int **21**(3), 85-88 (2001). doi:10.1007/s00296-001-0142-2

172. Cetinkaya, M., Cetinkaya, H., Ulusoy, E., Baz, S., MemiÅŸ, A., YaÅŸa, H., Yanik, B., OztÃ¼rk, B., UzunalimoÄŸlu, O.: Effect of postnecrotic and alcoholic hepatic cirrhosis on development of benign prostatic hyperplasia. Prostate **36**(2), 80-84 (1998). doi:10.1002/(sici)1097-0045(19980701)36:2<80::aid-pros2>3.0.co;2-i

173. Cetinkaya, M., Cetinkaya, H., Ulusoy, E., Baz, S., Memiş, A., Yaşa, H., Yanik, B., Oztürk, B., Uzunalimoğlu, O.: Effect of postnecrotic and alcoholic hepatic cirrhosis on development of benign prostatic hyperplasia. Prostate **36**(2), 80-84 (1998). doi:10.1002/(sici)1097-0045(19980701)36:2<80::aid-pros2>3.0.co;2-i

174. Chan, Y.X., Alfonso, H., Chubb, S.A., Handelsman, D.J., Fegan, P.G., Hankey, G.J., Golledge, J., Flicker, L., Yeap, B.B.: Higher Dihydrotestosterone Is Associated with the Incidence of Lung Cancer in Older Men. Horm Cancer **8**(2), 119-126 (2017). doi:10.1007/s12672-017-0287-4

175. Chang, S., Goszczak, A.J., Skakkebæk, A., Fedder, J., Bojesen, A., Bor, M.V., de Maat, M.P.M., Gravholt, C.H., Münster, A.B., Benderradji, H., Barbotin, A.L., Leroy-Billiard, M., Prasivoravong, J., Marcelli, F., Decanter, C., Robin, G., Mitchell, V., Rigot, J.M., Bongiovanni, A., Sauve, F., Buée, L., Maurage, C.A., Cartigny, M., Villers, A., Prevot, V., Catteau-Jonard, S., Sergeant, N., Giacobini, P., Pigny, P., Leroy, C.: Reduced fibrin clot lysis in Klinefelter syndrome associated with hypogonadism

Defining Reference Ranges for Serum Anti-Müllerian Hormone on a Large Cohort of Normozoospermic Adult Men Highlights New Potential Physiological Functions of AMH on FSH Secretion and Sperm Motility. Endocr Connect **11**(5), 1878-1887 (2022). doi:10.1530/ec-21-0490

10.1210/clinem/dgac218

176. Chang, T.C., Tung, C.C., Hsiao, Y.L.: Hormonal changes in elderly men with non-insulin-dependent diabetes mellitus and the hormonal relationships to abdominal adiposity. Gerontology **40**(5), 260-267 (1994). doi:10.1159/000213594

177. Charchar, F.J., Tomaszewski, M., Lacka, B., Zakrzewski, J., Zukowska-Szczechowska, E., Grzeszczak, W., Dominiczak, A.F.: Association of the human Y chromosome with cholesterol levels in the general population. Arterioscler Thromb Vasc Biol **24**(2), 308-312 (2004). doi:10.1161/01.ATV.0000113291.39267.0a

178. Chasland, L.C., Knuiman, M.W., Divitini, M.L., Murray, K., Handelsman, D.J., Flicker, L., Hankey, G.J., Almeida, O.P., Golledge, J., Ridgers, N.D., Naylor, L.H., Green, D.J., Yeap, B.B.: Higher circulating androgens and higher physical activity levels are associated with less central adiposity and lower risk of cardiovascular death in older men. Clin Endocrinol (Oxf) **90**(2), 375-383 (2019). doi:10.1111/cen.13905

179. Chatzittofis, A., Boström, A.E., Öberg, K.G., Flanagan, J.N., Schiöth, H.B., Arver, S., Jokinen, J., Xu, Y., Ma, X., Shen, Y., Wang, Y., Zhou, J., Bao, Y.: Normal Testosterone but Higher Luteinizing Hormone Plasma Levels in Men With Hypersexual Disorder

Influence of Sex Hormones on the Relationship Between Body Fat and Glycated Albumin Levels. Sex Med **8**(2), 243-250 (2020). doi:10.1016/j.esxm.2020.02.005

10.1016/j.jsxm.2020.02.006

180. Chauhan, S., Collins, K., Kruger, M., Diamond, M.P.: Effect of gonadotropin-releasing hormone hypogonadism on insulin action as assessed by hyperglycemic clamp studies in men. Fertil Steril **81**(4), 1092-1098 (2004). doi:10.1016/j.fertnstert.2003.08.052

181. Chen, C., Wang, N., Nie, X., Han, B., Li, Q., Chen, Y., Zhai, H., Zhu, C., Chen, Y., Xia, F., Lu, M., Lin, D., Lu, Y.: Blood Cadmium Level Associates with Lower Testosterone and Sex Hormone-Binding Globulin in Chinese men: from SPECT-China Study, 2014. Biol Trace Elem Res **171**(1), 71-78 (2016). doi:10.1007/s12011-015-0526-x

182. Chen, C., Zhai, H., Cheng, J., Weng, P., Chen, Y., Li, Q., Wang, C., Xia, F., Wang, N., Lu, Y.: Causal Link Between Vitamin D and Total Testosterone in Men: A Mendelian Randomization Analysis. J Clin Endocrinol Metab **104**(8), 3148-3156 (2019). doi:10.1097/ju.0000000000000205

10.1210/jc.2018-01874

183. Chen, C.C., Wang, S.S., Jeng, F.S., Lee, S.D.: Metabolic bone disease of liver cirrhosis: is it parallel to the clinical severity of cirrhosis? J Gastroenterol Hepatol **11**(5), 417-421 (1996). doi:10.1111/j.1440-1746.1996.tb00284.x

184. Chen, H.R., Tian, R.H., Li, P., Chen, H.X., Xia, S.J., Li, Z.: Estradiol is an independent risk factor for organic erectile dysfunction in eugonadal young men. Asian journal of andrology **22**(6), 636-641 (2020). doi:10.4103/aja.aja_135_19

185. Chen, L., Chen, Y.M., Wang, L.J., Wei, J., Tan, Y.Z., Zhou, J.Y., Yang, Y., Chen, Y.M., Ling, W.H., Zhu, H.L.: Higher homocysteine and lower betaine increase the risk of microangiopathy in patients with diabetes mellitus carrying the GG genotype of PEMT G774C. Diabetes Metab Res Rev **29**(8), 607-617 (2013). doi:10.1002/dmrr.2432

186. Chen, R.Y., Nordin, B.E., Need, A.G., Scopacasa, F., Wishart, J., Morris, H.A., Horowitz, M.: Relationship between calcium absorption and plasma dehydroepiandrosterone sulphate (DHEAS) in healthy males. Clin Endocrinol (Oxf) **69**(6), 864-869 (2008). doi:10.1111/j.1365-2265.2008.03272.x

187. Chen, S.F., Yao, F.J., Sun, X.Z., Wu, R.P., Huang, Y.P., Zheng, F.F., Yang, Q.Y., Han, D.Y., Xie, M.Q., Ding, M., Zhang, Y., Liu, G.H., Deng, C.H.: Brachial artery flow-mediated dilatation and carotid intima-media thickness in young ED patients with insulin resistance. Int J Impot Res **28**(5), 194-199 (2016). doi:10.1038/ijir.2016.30

188. Chen, S.S.: Differences in the clinical characteristics between young and elderly men with varicocoele. Int J Androl **35**(5), 695-699 (2012). doi:10.1111/j.1365-2605.2012.01257.x

189. Chen, S.S., Huang, W.J.: Differences in biochemical markers and body mass index between patients with and without varicocele. J Chin Med Assoc **73**(4), 194-198 (2010). doi:10.1016/s1726-4901(10)70040-x

190. Chen, T., Wu, F., Wang, X., Ma, G., Xuan, X., Tang, R., Ding, S., Lu, J.: Different levels of estradiol are correlated with sexual dysfunction in adult men. Sci Rep **10**(1), 12660 (2020). doi:10.1038/s41598-020-69712-6

191. Chen, Y.P., Nie, L.L., Li, H.G., Liu, T.H., Fang, F., Zhao, K., Yang, R.F., Ma, X.L., Kong, X.B., Zhang, H.P., Guan, H.T., Xia, W., Hong, W.X., Duan, S., Zeng, X.C., Shang, X.J., Zhou, Y.Z., Gu, Y.Q., Wu, W.X., Xiong, C.L.: The rs5934505 single nucleotide polymorphism (SNP) is associated with low testosterone and late-onset hypogonadism, but the rs10822184 SNP is associated with overweight and obesity in a Chinese Han population: a case-control study. Andrology **4**(1), 68-74 (2016). doi:10.1111/andr.12127

192. Chen, Y.P., Wang, J., Zhao, K., Shang, X.J., Wu, H.Q., Qing, X.R., Fang, F., Zhang, Y., Shang, J., Li, H.G., Zhang, H.P., Guan, H.T., Zhou, Y.Z., Gu, Y.Q., Wu, W.X., Xiong, C.L.: The plasma miR-125a, miR-361 and miR-133a are promising novel biomarkers for Late-Onset Hypogonadism. Sci Rep **6**, 23531 (2016). doi:10.1038/srep23531

193. Chen, Z., Shen, X., Tian, K., Liu, Y., Xiong, S., Yu, Q., Dai, L., Shi, Y., Zhang, R., Zeng, R., Wan, Q., Xiong, C., Zhou, Y.: Bioavailable testosterone is associated with symptoms of depression in adult men. J Int Med Res **48**(8), 300060520941715 (2020). doi:10.1177/0300060520941715

194. Cheung, A.S., Baqar, S., Sia, R., Hoermann, R., Iuliano-Burns, S., Vu, T.D., Chiang, C., Hamilton, E.J., Gianatti, E., Seeman, E., Zajac, J.D., Grossmann, M.: Testosterone levels increase in association with recovery from acute fracture in men. Osteoporos Int **25**(8), 2027-2033 (2014). doi:10.1007/s00198-014-2727-0

195. Child, D.F., Bu'Lock, D.E., Hillier, V.F., Anderson, D.C.: Heterogeneity in adrenal steroidogenesis in normal men and women. Clin Endocrinol (Oxf) **11**(4), 383-389 (1979). doi:10.1111/j.1365-2265.1979.tb03090.x

196. Chin, K.Y., Ima-Nirwana, S., Mohamed, I.N., Hanapi Johari, M., Ahmad, F., Mohamed Ramli, E.S., Wan Ngah, W.Z.: Insulin-like growth factor-1 is a mediator of age-related decline of bone health status in men. Aging Male **17**(2), 102-106 (2014). doi:10.3109/13685538.2014.896895

197. Chin, K.Y., Soelaiman, I.N., Mohamed, I.N., Ngah, W.Z.: Serum testosterone, sex hormone-binding globulin and total calcium levels predict the calcaneal speed of sound in men. Clinics (Sao Paulo) **67**(8), 911-916 (2012). doi:10.6061/clinics/2012(08)10

198. Chin, K.Y., Soelaiman, I.N., Naina Mohamed, I., Shahar, S., Teng, N.I., Suhana Mohd Ramli, E., Ahmad, F., Aminuddin, A., Zurinah Wan Ngah, W.: Testosterone is associated with age-related changes in bone health status, muscle strength and body composition in men. Aging Male **15**(4), 240-245 (2012). doi:10.3109/13685538.2012.724740

199. Chock, B., Lin, T.C., Li, C.S., Swislocki, A.: Plasma testosterone is associated with Framingham risk score. Aging Male **15**(3), 134-139 (2012). doi:10.3109/13685538.2011.654369

200. Christ-Crain, M., Meier, C., Huber, P.R., Zimmerli, L., Mueller, B.: Value of gonadotropin-releasing hormone testing in the differential diagnosis of androgen deficiency in elderly men. J Clin Endocrinol Metab **90**(3), 1280-1286 (2005). doi:10.1210/jc.2004-0850

201. Christiansen, K., Knussmann, R.: Sex hormones and cognitive functioning in men. Neuropsychobiology **18**(1), 27-36 (1987). doi:10.1159/000118389

202. Christiansen, K., Knussmann, R.: Androgen levels and components of aggressive behavior in men. Horm Behav **21**(2), 170-180 (1987). doi:10.1016/0018-506x(87)90042-0

203. Christiansen, K., Knussmann, R., Couwenbergs, C.: Sex hormones and stress in the human male. Horm Behav **19**(4), 426-440 (1985). doi:10.1016/0018-506x(85)90039-x

204. Chu, L.W., Tam, S., Kung, A.W., Lo, S., Fan, S., Wong, R.L., Morley, J.E., Lam, K.S.: Serum total and bioavailable testosterone levels, central obesity, and muscle strength changes with aging in healthy Chinese men. J Am Geriatr Soc **56**(7), 1286-1291 (2008). doi:10.1111/j.1532-5415.2008.01746.x

205. Chueh, K.S., Huang, S.P., Lee, Y.C., Wang, C.J., Yeh, H.C., Li, W.M., Wu, W.J., Tsai, Y.F., Tsai, C.C., Juan, H.C., Huang, C.H., Liu, C.C.: The comparison of the aging male symptoms (AMS) scale and androgen deficiency in the aging male (ADAM) questionnaire to detect androgen deficiency in middle-aged men. J Androl **33**(5), 817-823 (2012). doi:10.2164/jandrol.111.015628

206. Cicero, T.J., Bell, R.D., Wiest, W.G., Allison, J.H., Polakoski, K., Robins, E.: Function of the male sex organs in heroin and methadone users. N Engl J Med **292**(17), 882-887 (1975). doi:10.1056/nejm197504242921703

207. Cimen, S., Dursun, M., Sulukaya, M., Besiroglu, H.: Could the monocyte/HDL cholesterol ratio be an early marker of erectile dysfunction? Aging Male **23**(5), 694-699 (2020). doi:10.1080/13685538.2019.1574735

208. Cinar, V., Baltaci, A.K., Mogulkoc, R., Kilic, M.: Testosterone levels in athletes at rest and exhaustion: effects of calcium supplementation. Biol Trace Elem Res **129**(1-3), 65-69 (2009). doi:10.1007/s12011-008-8294-5

209. Cinislioglu, A.E., Cinislioglu, N., Demirdogen, S.O., Sam, E., Akkas, F., Altay, M.S., Utlu, M., Sen, I.A., Yildirim, F., Kartal, S., Aydin, H.R., Karabulut, I., Ozbey, I.: The relationship of serum testosterone levels with the clinical course and prognosis of COVID-19 disease in male patients: A prospective study. Andrology **10**(1), 24-33 (2022). doi:10.1210/clinem/dgab481

10.1111/andr.13081

210. Cissewski, K., Tharandt, L., Benker, G., Reinwein, D.: Metabolism of GnRH in man: influence of estrogens. Horm Res **25**(4), 199-205 (1987). doi:10.1159/000180653

211. Clasey, J.L., Weltman, A., Patrie, J., Weltman, J.Y., Pezzoli, S., Bouchard, C., Thorner, M.O., Hartman, M.L.: Abdominal visceral fat and fasting insulin are important predictors of 24-hour GH release independent of age, gender, and other physiological factors. J Clin Endocrinol Metab **86**(8), 3845-3852 (2001). doi:10.1210/jcem.86.8.7731

212. Cobeta, P., Osorio, A., Cuadrado-Ayuso, M., García-Moreno, F., Pestaña, D., Galindo, J., Botella-Carretero, J.I.: Sleeve Gastrectomy and Gastric Bypass Decrease the Carotid Intima-Media Thickness in Obese Men: Association with Weight Loss, Cardiovascular Risk Factors, and Circulating Testosterone. Obes Surg **30**(3), 851-859 (2020). doi:10.1007/s11695-020-04405-7

213. Coelingh Bennink, H.J.T., Zimmerman, Y., Verhoeven, C., Dutman, A.E., Mensinga, T., Kluft, C., Reisman, Y., Debruyne, F.M.J.: A Dose-Escalating Study With the Fetal Estrogen Estetrol in Healthy Men. J Clin Endocrinol Metab **103**(9), 3239-3249 (2018). doi:10.1210/jc.2018-00147

214. Colao, A., De Rosa, M., Pivonello, R., Balestrieri, A., Cappabianca, P., Di Sarno, A., Rochira, V., Carani, C., Lombardi, G.: Short-term suppression of GH and IGF-I levels improves gonadal function and sperm parameters in men with acromegaly. The Journal of clinical endocrinology and metabolism **87**(9), 4193-4197 (2002). doi:10.1210/jc.2002-020453

215. Colao, A., Spiezia, S., Di Somma, C., Marzullo, P., Cerbone, G., Pivonello, R., Faggiano, A., Lombardi, G.: Effect of GH and/or testosterone deficiency on the prostate: an ultrasonographic and endocrine study in GH-deficient adult patients. Eur J Endocrinol **143**(1), 61-69 (2000). doi:10.1530/eje.0.1430061

216. Colao, A., Vitale, G., Cappabianca, P., Briganti, F., Ciccarelli, A., De Rosa, M., Zarrilli, S., Lombardi, G.: Outcome of cabergoline treatment in men with prolactinoma: effects of a 24-month treatment on prolactin levels, tumor mass, recovery of pituitary function, and semen analysis. J Clin Endocrinol Metab **89**(4), 1704-1711 (2004). doi:10.1210/jc.2003-030979

217. Colao, A., Vitale, G., Di Sarno, A., Spiezia, S., Guerra, E., Ciccarelli, A., Lombardi, G.: Prolactin and prostate hypertrophy: a pilot observational, prospective, case-control study in men with prolactinoma. J Clin Endocrinol Metab **89**(6), 2770-2775 (2004). doi:10.1210/jc.2003-032055

218. Collier, C.P., Morales, A., Clark, A., Lam, M., Wynne-Edwards, K., Black, A.: The significance of biological variation in the diagnosis of testosterone deficiency, and consideration of the relevance of total, free and bioavailable testosterone determinations. J Urol **183**(6), 2294-2299 (2010). doi:10.1016/j.juro.2010.02.011

219. Comhaire, F., Vermeulen, A.: Plasma testosterone in patients with varicocele and sexual inadequacy. J Clin Endocrinol Metab **40**(5), 824-829 (1975). doi:10.1210/jcem-40-5-824

220. Condorelli, R.A., Calogero, A.E., La Vignera, S.: Hyperestrogenism and low serum testosterone-17β-estradiol ratio are associated with non-bacterial male accessory gland inflammation. Int J Immunopathol Pharmacol **29**(3), 488-493 (2016). doi:10.1177/0394632016644446

221. Cook, M.B., Wood, S.N., Cash, B.D., Young, P., Acosta, R.D., Falk, R.T., Pfeiffer, R.M., Hu, N., Su, H., Wang, L., Wang, C., Gherman, B., Giffen, C., Dykes, C., Turcotte, V., Caron, P., Guillemette, C., Dawsey, S.M., Abnet, C.C., Hyland, P.L., Taylor, P.R.: Association between circulating levels of sex steroid hormones and Barrett's esophagus in men: a case-control analysis. Clin Gastroenterol Hepatol **13**(4), 673-682 (2015). doi:10.1016/j.cgh.2014.08.027

222. Corona, G., Boddi, V., Lotti, F., Gacci, M., Carini, M., De Vita, G., Sforza, A., Forti, G., Mannucci, E., Maggi, M.: The relationship of testosterone to prostate-specific antigen in men with sexual dysfunction. J Sex Med **7**(1 Pt 1), 284-292 (2010). doi:10.1111/j.1743-6109.2009.01549.x

223. Corona, G., Jannini, E.A., Mannucci, E., Fisher, A.D., Lotti, F., Petrone, L., Balercia, G., Bandini, E., Chiarini, V., Forti, G., Maggi, M.: Different testosterone levels are associated with ejaculatory dysfunction. J Sex Med **5**(8), 1991-1998 (2008). doi:10.1111/j.1743-6109.2008.00803.x

224. Corona, G., Mannucci, E., Lotti, F., Boddi, V., Jannini, E.A., Fisher, A.D., Monami, M., Sforza, A., Forti, G., Maggi, M.: Impairment of couple relationship in male patients with sexual dysfunction is associated with overt hypogonadism. J Sex Med **6**(9), 2591-2600 (2009). doi:10.1111/j.1743-6109.2009.01352.x

225. Corona, G., Mannucci, E., Ricca, V., Lotti, F., Boddi, V., Bandini, E., Balercia, G., Forti, G., Maggi, M.: The age-related decline of testosterone is associated with different specific symptoms and signs in patients with sexual dysfunction. Int J Androl **32**(6), 720-728 (2009). doi:10.1111/j.1365-2605.2009.00952.x

226. Corona, G., Rastrelli, G., Balercia, G., Sforza, A., Forti, G., Mannucci, E., Maggi, M.: Perceived reduced sleep-related erections in subjects with erectile dysfunction: psychobiological correlates. J Sex Med **8**(6), 1780-1788 (2011). doi:10.1111/j.1743-6109.2011.02241.x

227. Corpas, E., Harman, S.M., Piñeyro, M.A., Roberson, R., Blackman, M.R.: Growth hormone (GH)-releasing hormone-(1-29) twice daily reverses the decreased GH and insulin-like growth factor-I levels in old men. J Clin Endocrinol Metab **75**(2), 530-535 (1992). doi:10.1210/jcem.75.2.1379256

228. Corrales, J.J., Almeida, M., Cordero, M., Martín-Martín, L., Méndez, C., Miralles, J.M., Orfao, A.: Enhanced immunological response by dendritic cells in male hypogonadism. Eur J Clin Invest **42**(11), 1205-1212 (2012). doi:10.1111/j.1365-2362.2012.02712.x

229. Corrales-Hernández, J.J., González-Buitrago, J.M., Pastor-Encinas, I., García-Diez, L.C., Miralles, J.M.: Androgen environment and beta-glucuronidase activity in the human kidney. Arch Androl **20**(3), 185-191 (1988). doi:10.3109/01485018808987072

230. Costanzo, P.R., Suárez, S.M., Kozak, A.E., Knoblovits, P.: Seasonal Variations in Sex Steroids in a Young Male Population and Their Relationship with Plasma Levels of Vitamin D. World J Mens Health **40**(2), 308-315 (2022). doi:10.5534/wjmh.200156

231. Costanzo, P.R., Suarez, S.M., Scaglia, H.E., Zylbersztein, C., Litwak, L.E., Knoblovits, P.: Evaluation of the hypothalamic-pituitary-gonadal axis in eugonadal men with type 2 diabetes mellitus. Andrology **2**(1), 117-124 (2014). doi:10.1111/j.2047-2927.2013.00163.x

232. Couwenbergs, C., Knussmann, R., Christiansen, K.: Comparisons of the intra- and inter-individual variability in sex hormone levels of men. Ann Hum Biol **13**(1), 63-72 (1986). doi:10.1080/03014468600008201

233. Couwenbergs, C.J.: Acute effects of drinking beer or wine on the steroid hormones of healthy men. Journal of steroid biochemistry **31**(4a), 467-473 (1988). doi:10.1016/0022-4731(88)90317-2

234. Coviello, A.D., Bremner, W.J., Matsumoto, A.M., Herbst, K.L., Amory, J.K., Anawalt, B.D., Yan, X., Brown, T.R., Wright, W.W., Zirkin, B.R., Jarow, J.P.: Intratesticular testosterone concentrations comparable with serum levels are not sufficient to maintain normal sperm production in men receiving a hormonal contraceptive regimen. Journal of andrology **25**(6), 931-938 (2004).

235. Crispino, S., Tancini, G., Barni, S., Lissoni, P.: Evidence for altered hypothalamic-hypophyseal-gonadal axis in untreated patients with testicular germ-cell tumor. Tumori **75**(5), 505-509 (1989). doi:10.1177/030089168907500523

236. Dabbs, J.M., Jr.: Age and seasonal variation in serum testosterone concentration among men. Chronobiology international **7**(3), 245-249 (1990).

237. Dacal, K., Sereika, S.M., Greenspan, S.L.: Quality of life in prostate cancer patients taking androgen deprivation therapy. J Am Geriatr Soc **54**(1), 85-90 (2006). doi:10.1111/j.1532-5415.2005.00567.x

238. Dai, W.S., Kuller, L.H., LaPorte, R.E., Gutai, J.P., Falvo-Gerard, L., Caggiula, A.: The epidemiology of plasma testosterone levels in middle-aged men. American journal of epidemiology **114**(6), 804-816 (1981).

239. Dammann, H.G., Bethke, T., Burkhardt, F., Wolf, N., Khalil, H., Luehmann, R.: Effects of pantoprazole on endocrine function in healthy male volunteers. Aliment Pharmacol Ther **8**(5), 549-554 (1994). doi:10.1111/j.1365-2036.1994.tb00329.x

240. Daniell, H.W.: Hypogonadism in men consuming sustained-action oral opioids. J Pain **3**(5), 377-384 (2002). doi:10.1054/jpai.2002.126790

241. David, M., Jahan, S., Hussain, J., Rehman, H., Cloete, K.J., Afsar, T., Almajwal, A., Alruwaili, N.W., Razak, S.: Biochemical and reproductive biomarker analysis to study the consequences of heavy metal burden on health profile of male brick kiln workers. Sci Rep **12**(1), 7172 (2022). doi:10.1155/2022/3780741

10.1038/s41598-022-11304-7

242. Davidson, D.W., O'Carroll, R., Bancroft, J.: Increasing circulating androgens with oral testosterone undecanoate in eugonadal men. J Steroid Biochem **26**(6), 713-715 (1987). doi:10.1016/0022-4731(87)91044-2

243. Davies, T.F., Gomez-Pan, A., Watson, M.J., Mountjoy, C.Q., Hanker, J.P., Besser, G.M., Hall, R.: Reduced 'gonadotrophin response to releasing hormone' after chronic administration to impotent men. Clin Endocrinol (Oxf) **6**(3), 213-218 (1977). doi:10.1111/j.1365-2265.1977.tb03317.x

244. Davio, A., Woolcock, H., Nanba, A.T., Rege, J., O'Day, P., Ren, J., Zhao, L., Ebina, H., Auchus, R., Rainey, W.E., Turcu, A.F.: Sex Differences in 11-Oxygenated Androgen Patterns Across Adulthood. J Clin Endocrinol Metab **105**(8), e2921-2929 (2020). doi:10.1210/clinem/dgaa343

245. de Kretser, D.M., Burger, H.G., Hudson, B., Keogh: The pituitary-testicular response to luteinising hormone releasing hormone administration to normal men. Aust N Z J Med **5**(3), 227-230 (1975). doi:10.1111/j.1445-5994.1975.tb04573.x

246. de Kretser, D.M., McLachlan, R.I., Robertson, D.M., Burger, H.G.: Serum inhibin levels in normal men and men with testicular disorders. J Endocrinol **120**(3), 517-523 (1989). doi:10.1677/joe.0.1200517

247. de Neergaard, R., Nielsen, J.E., JÃ¸rgensen, A., Toft, B.G., Goetze, J.P., JÃ¸rgensen, N.: Positive association between cholesterol in human seminal plasma and sperm counts: results from a cross-sectional cohort study and immunohistochemical investigations. Andrology **6**(6), 817-828 (2018). doi:10.1111/andr.12532

248. de Neergaard, R., Nielsen, J.E., Jørgensen, A., Toft, B.G., Goetze, J.P., Jørgensen, N.: Positive association between cholesterol in human seminal plasma and sperm counts: results from a cross-sectional cohort study and immunohistochemical investigations. Andrology **6**(6), 817-828 (2018). doi:10.1111/andr.12532

249. de Ronde, W., van der Schouw, Y.T., Muller, M., Grobbee, D.E., Gooren, L.J., Pols, H.A., de Jong, F.H.: Associations of sex-hormone-binding globulin (SHBG) with non-SHBG-bound levels of testosterone and estradiol in independently living men. J Clin Endocrinol Metab **90**(1), 157-162 (2005). doi:10.1210/jc.2004-0422

250. De Rosa, M., Zarrilli, S., Vitale, G., Di Somma, C., Orio, F., Tauchmanova, L., Lombardi, G., Colao, A.: Six months of treatment with cabergoline restores sexual potency in hyperprolactinemic males: an open longitudinal study monitoring nocturnal penile tumescence. J Clin Endocrinol Metab **89**(2), 621-625 (2004). doi:10.1210/jc.2003-030852

251. de Vries, C.P., Gooren, L.J., Oe, P.L.: Haemodialysis and testicular function. Int J Androl **7**(2), 97-103 (1984). doi:10.1111/j.1365-2605.1984.tb00765.x

252. Defay, R., Papoz, L., Barny, S., Bonnot-Lours, S., CacÃ¨s, E., Simon, D.: Hormonal status and NIDDM in the European and Melanesian populations of New Caledonia: a case-control study. The CALedonia DIAbetes Mellitus (CALDIA) Study Group. Int J Obes Relat Metab Disord **22**(9), 927-934 (1998). doi:10.1038/sj.ijo.0800697

253. Defay, R., Papoz, L., Barny, S., Bonnot-Lours, S., Cacès, E., Simon, D.: Hormonal status and NIDDM in the European and Melanesian populations of New Caledonia: a case-control study. The CALedonia DIAbetes Mellitus (CALDIA) Study Group. Int J Obes Relat Metab Disord **22**(9), 927-934 (1998). doi:10.1038/sj.ijo.0800697

254. DeFina, L.F., Radford, N.B., Leonard, D., Wilson, R.K., Cooper, T.C., Clark, S.M., Willis, B.L., Vega, G.L., Barlow, C.E., Farrell, S.W., Gibbons, L.W., Yildiz, B.O., Gruntmanis, U.: The association of cardiorespiratory fitness, body mass index, and age with testosterone levels at screening of healthy men undergoing preventive medical examinations: The Cooper Center Longitudinal Study. Maturitas **118**, 1-6 (2018). doi:10.1016/j.maturitas.2018.09.004

255. Dehennin, L., PÃ©rÃ¨s, G.: Plasma and urinary markers of oral testosterone misuse by healthy men in presence of masking epitestosterone administration. Int J Sports Med **17**(5), 315-319 (1996). doi:10.1055/s-2007-972853

256. Dehennin, L., Pérès, G.: Plasma and urinary markers of oral testosterone misuse by healthy men in presence of masking epitestosterone administration. Int J Sports Med **17**(5), 315-319 (1996). doi:10.1055/s-2007-972853

257. Del Prete, M., Mauriello, M.C., Faggiano, A., Di Somma, C., Monfrecola, G., Fabbrocini, G., Colao, A.: Insulin resistance and acne: a new risk factor for men? Endocrine **42**(3), 555-560 (2012). doi:10.1007/s12020-012-9647-6

258. Delitala, G., Masala, A., Alagna, S., Lotti, G.: Luteinizing hormone, follicle stimulating hormone and testosterone in normal and impotent men following LHRH and HCG stimulation. Clin Endocrinol (Oxf) **6**(1), 11-15 (1977). doi:10.1111/j.1365-2265.1977.tb01991.x

259. Deschenes, M.R., Kraemer, W.J., Bush, J.A., Doughty, T.A., Kim, D., Mullen, K.M., Ramsey, K.: Biorhythmic influences on functional capacity of human muscle and physiological responses. Med Sci Sports Exerc **30**(9), 1399-1407 (1998). doi:10.1097/00005768-199809000-00008

260. Deslypere, J.P., Vermeulen, A.: Leydig cell function in normal men: effect of age, life-style, residence, diet, and activity. J Clin Endocrinol Metab **59**(5), 955-962 (1984). doi:10.1210/jcem-59-5-955

261. Dettling, A., Skopp, G., Graw, M., Haffner, H.T.: The influence of sex hormones on the elimination kinetics of ethanol. Forensic Sci Int **177**(2-3), 85-89 (2008). doi:10.1016/j.forsciint.2007.11.002

262. Di Luigi, L., Gentile, V., Pigozzi, F., Parisi, A., Giannetti, D., Romanelli, F.: Physical activity as a possible aggravating factor for athletes with varicocele: impact on the semen profile. Hum Reprod **16**(6), 1180-1184 (2001). doi:10.1093/humrep/16.6.1180

263. Di Stefano, M., Jorizzo, R.A., Brusco, G., Cecchetti, L., Sciarra, G., Loperfido, S., Brandi, G., Gasbarrini, G., Corazza, G.R.: Bone mass and metabolism in Whipple's disease: the role of hypogonadism. Scand J Gastroenterol **33**(11), 1180-1185 (1998). doi:10.1080/00365529850172548

264. Di Vincenzo, A., Silvestrin, V., Bertoli, E., Foletto, M., Pagano, C., Fabris, R., Vettor, R., Busetto, L., Rossato, M.: Short-term effects of surgical weight loss after sleeve gastrectomy on sex steroids plasma levels and PSA concentration in men with severe obesity. Aging Male **23**(5), 464-468 (2020). doi:10.1080/13685538.2018.1528445

265. Dockery, F., Bulpitt, C.J., Donaldson, M., Fernandez, S., Rajkumar, C.: The relationship between androgens and arterial stiffness in older men. J Am Geriatr Soc **51**(11), 1627-1632 (2003). doi:10.1046/j.1532-5415.2003.51515.x

266. Doering, C.H., Brodie, H.K., Kraemer, H.C., Moos, R.H., Becker, H.B., Hamburg, D.A.: Negative affect and plasma testosterone: a longitudinal human study. Psychosom Med **37**(6), 484-491 (1975). doi:10.1097/00006842-197511000-00003

267. Doering, C.H., Kraemer, H.C., Brodie, H.K., Hamburg, D.A.: A cycle of plasma testosterone in the human male. J Clin Endocrinol Metab **40**(3), 492-500 (1975). doi:10.1210/jcem-40-3-492

268. Doerr, P., Kockott, G., Vogt, H.J., Pirke, K.M., Dittmar, F.: Plasma testosterone, estradiol, and semen analysis in male homosexuals. Arch Gen Psychiatry **29**(6), 829-833 (1973). doi:10.1001/archpsyc.1973.04200060101016

269. Doğan Bulut, S., Bulut, S., Güriz, O.: The relationship between sex hormone profiles and symptoms of schizophrenia in men. Compr Psychiatry **69**, 186-192 (2016). doi:10.1016/j.comppsych.2016.06.005

270. Dolscheid-Pommerich, R.C., Stoffel-Wagner, B., Fimmers, R., Eichhorn, L.: Changes in hormones after apneic hypoxia/hypercapnia - An investigation in voluntary apnea divers. Respir Physiol Neurobiol **298**, 103845 (2022). doi:10.1016/j.resp.2022.103845

271. Dony, J.M., Smals, A.G., Rolland, R., Fauser, B.C., Thomas, C.M.: Differential effect of luteinizing hormone-releasing hormone infusion on testicular steroids in normal men and patients with idiopathic oligospermia. Fertil Steril **42**(2), 274-280 (1984). doi:10.1016/s0015-0282(16)48026-x

272. Dörner, G., Rohde, W., Stahl, F., Krell, L., Masius, W.G.: A neuroendocrine predisposition for homosexuality in men. Arch Sex Behav **4**(1), 1-8 (1975). doi:10.1007/bf01541882

273. Dote-Montero, M., De-la, O.A., Jurado-Fasoli, L., Ruiz, J.R., Castillo, M.J., Amaro-Gahete, F.J.: The effects of three types of exercise training on steroid hormones in physically inactive middle-aged adults: a randomized controlled trial. Eur J Appl Physiol **121**(8), 2193-2206 (2021). doi:10.1007/s00421-021-04692-7

274. Duarte, M.F., Luis, C., Baylina, P., Faria, M.I., Fernandes, R., La Fuente, J.M.: Clinical and metabolic implications of obesity in prostate cancer: is testosterone a missing link? Aging Male **22**(4), 228-240 (2019). doi:10.1080/13685538.2018.1519695

275. Dumbraveanu, I., Banov, P., Arian, I., Ceban, E.: The Correlations of Clinical and Biochemical Indices of Vitamin D with Erectile Dysfunction. J Med Life **13**(2), 144-150 (2020). doi:10.25122/jml-2020-0009

276. Dunajska, K., Milewicz, A., Szymczak, J., JÃªdrzejuk, D., Kuliczkowski, W., Salomon, P., Nowicki, P.: Evaluation of sex hormone levels and some metabolic factors in men with coronary atherosclerosis. Aging Male **7**(3), 197-204 (2004). doi:10.1080/13685530400004181

277. Duran, C., Yonem, A., Ustun, I., Ozcan, O., Ipcioglu, O.M., Basekim, C.C.: Plasma ghrelin levels in males with idiopathic hypogonadotropic hypogonadism. Endocrine **34**(1-3), 81-86 (2008). doi:10.1007/s12020-008-9102-x

278. Durazzo, M., Premoli, A., Di Bisceglie, C., Bertagna, A., Faga, E., Biroli, G., Manieri, C., Bo, S., Pagano, G.: Alterations of seminal and hormonal parameters: An extrahepatic manifestation of HCV infection? World J Gastroenterol **12**(19), 3073-3076 (2006). doi:10.3748/wjg.v12.i19.3073

279. Ebata, T., Itamura, R., Aizawa, H., Niimura, M.: Serum sex hormone levels in adult patients with atopic dermatitis. J Dermatol **23**(9), 603-605 (1996). doi:10.1111/j.1346-8138.1996.tb02662.x

280. Eendebak, R.J., Huhtaniemi, I.T., Pye, S.R., Ahern, T., O'Neill, T.W., Bartfai, G., Casanueva, F.F., Maggi, M., Forti, G., Alston, R.D., Giwercman, A., Han, T.S., Kula, K., Lean, M.E., Punab, M., Pendleton, N., Keevil, B.G., Vanderschueren, D., Rutter, M.K., Tampubolon, G., Goodacre, R., Wu, F.C.: The androgen receptor gene CAG repeat  in relation to 4-year changes in  androgen-sensitive endpoints in  community-dwelling older European men. Eur J Endocrinol **175**(6), 583-593 (2016). doi:10.1530/eje-16-0447

281. Egund, L., Isaksson, S., McGuigan, F.E., Giwercman, A., Ã…kesson, K.E., Del Giudice, F., Glover, F., Belladelli, F., De Berardinis, E., Sciarra, A., Salciccia, S., Kasman, A.M., Chen, T., Eisenberg, M.L.: High Luteinizing Hormone and Lower Levels of Sex Hormones in Younger Men With Distal Radius Fracture

Association of daily step count and serum testosterone among men in the United States. JBMR Plus **4**(11), e10421 (2020). doi:10.1002/jbm4.10421

10.1007/s12020-021-02631-2

282. Egund, L., Isaksson, S., McGuigan, F.E., Giwercman, A., Åkesson, K.E., Del Giudice, F., Glover, F., Belladelli, F., De Berardinis, E., Sciarra, A., Salciccia, S., Kasman, A.M., Chen, T., Eisenberg, M.L.: High Luteinizing Hormone and Lower Levels of Sex Hormones in Younger Men With Distal Radius Fracture

Association of daily step count and serum testosterone among men in the United States. JBMR Plus **4**(11), e10421 (2020). doi:10.1002/jbm4.10421

10.1007/s12020-021-02631-2

283. Ehala-Aleksejev, K., Punab, M.: The effect of metabolic syndrome on male reproductive health: A cross-sectional study in a group of fertile men and male partners of infertile couples. PLoS One **13**(3), e0194395 (2018). doi:10.6004/jnccn.2017.7046

10.1371/journal.pone.0194395

284. Ehrenkranz, J., Bliss, E., Sheard, M.H.: Plasma testosterone: correlation with aggressive behavior and social dominance in man. Psychosom Med **36**(6), 469-475 (1974). doi:10.1097/00006842-197411000-00002

285. El Maghraoui, A., Ouzzif, Z., Mounach, A., Ben-Ghabrit, A., Achemlal, L., Bezza, A., Ghozlani, I.: The relationship between sex steroids, bone turnover and vertebral fracture prevalence in asymptomatic men. Bone **49**(4), 853-857 (2011). doi:10.1016/j.bone.2011.06.022

286. El Maghraoui, A., Tellal, S., Chaouir, S., Lebbar, K., Bezza, A., Nouijai, A., Achemlal, L., Bouhssain, S., Derouiche el, M.: Bone turnover markers, anterior pituitary and gonadal hormones, and bone mass evaluation using quantitative computed tomography in ankylosing spondylitis. Clin Rheumatol **24**(4), 346-351 (2005). doi:10.1007/s10067-004-1039-8

287. Elias, A.N., Kayaleh, R.A., Pandian, M.R., Chune, G.: Inhibin and gonadotrophin secretion in physically active males after acute exercise. Human reproduction (Oxford, England) **6**(6), 747-750 (1991). doi:10.1093/oxfordjournals.humrep.a137422

288. Elias, A.N., Wilson, A.F., Pandian, M.R., Chune, G., Utsumi, A., Kayaleh, R., Stone, S.C.: Corticotropin releasing hormone and gonadotropin secretion in physically active males after acute exercise. Eur J Appl Physiol Occup Physiol **62**(3), 171-174 (1991). doi:10.1007/bf00643737

289. Elias, A.N., Wilson, A.F., Pandian, M.R., Rojas, F.J., Kayaleh, R., Stone, S.C., James, N.: Melatonin and gonadotropin secretion after acute exercise in physically active males. Eur J Appl Physiol Occup Physiol **66**(4), 357-361 (1993). doi:10.1007/bf00237782

290. Eller, N.H.: Total power and high frequency components of heart rate variability and risk factors for atherosclerosis. Auton Neurosci **131**(1-2), 123-130 (2007). doi:10.1016/j.autneu.2006.08.002

291. Elmlinger, M.W., Dengler, T., Weinstock, C., Kuehnel, W.: Endocrine alterations in the aging male. Clin Chem Lab Med **41**(7), 934-941 (2003). doi:10.1515/cclm.2003.142

292. Elzanaty, S., Rezanezhad, B., Dohle, G., Bermon, S., Garnier, P.Y.: Association between Serum Testosterone and PSA Levels in Middle-Aged Healthy Men from the General Population

Serum androgen levels and their relation to performance in track and field: mass spectrometry results from 2127 observations in male and female elite athletes. Curr Urol **10**(1), 40-44 (2017). doi:10.1159/000447149

10.1136/bjsports-2017-097792

293. Empen, K., Lorbeer, R., Dörr, M., Haring, R., Nauck, M., Gläser, S., Krebs, A., Reffelmann, T., Ewert, R., Völzke, H., Wallaschofski, H., Felix, S.B.: Association of testosterone levels with endothelial function in men: results from a population-based study. Arterioscler Thromb Vasc Biol **32**(2), 481-486 (2012). doi:10.1161/atvbaha.111.232876

294. Engeland, C.G., Sabzehei, B., Marucha, P.T.: Sex hormones and mucosal wound healing. Brain Behav Immun **23**(5), 629-635 (2009). doi:10.1016/j.bbi.2008.12.001

295. Enikeev, D., Taratkin, M., Morozov, A., Petov, V., Korolev, D., Shpikina, A., Spivak, L., Kharlamova, S., Shchedrina, I., Mestnikov, O., Fiev, D., Ganzha, T., Geladze, M., Mambetova, A., Kogan, E., Zharkov, N., Demyashkin, G., Shariat, S.F., Glybochko, P.: Prospective two-arm study of the testicular function in patients with COVID-19. Andrology **10**(6), 1047-1056 (2022). doi:10.1111/andr.13159

296. Erbler, H.C.: Suppression by the spironolactone metabolite canrenone of plasma testosterone in man. Naunyn Schmiedebergs Arch Pharmacol **285**(4), 403-406 (1974). doi:10.1007/bf00501468

297. Eriksson, A.L., Lorentzon, M., Vandenput, L., Labrie, F., Lindersson, M., Syvänen, A.C., Orwoll, E.S., Cummings, S.R., Zmuda, J.M., Ljunggren, O., Karlsson, M.K., Mellström, D., Ohlsson, C.: Genetic variations in sex steroid-related genes as predictors of serum estrogen levels in men. J Clin Endocrinol Metab **94**(3), 1033-1041 (2009). doi:10.1210/jc.2008-1283

298. Ermis, N., Deniz, F., Kepez, A., Kara, B., Azal, O., Kutlu, M.: Heart rate variability of young men with idiopathic hypogonadotropic hypogonadism. Auton Neurosci **152**(1-2), 84-87 (2010). doi:10.1016/j.autneu.2009.08.018

299. Errico, A.L., Parsons, O.A., Kling, O.R., King, A.C.: Investigation of the role of sex hormones in alcoholics' visuospatial deficits. Neuropsychologia **30**(5), 417-426 (1992). doi:10.1016/0028-3932(92)90089-5

300. ErsÃ¶z, H., Onde, M.E., Terekeci, H., Kurtoglu, S., Tor, H.: Causes of gynaecomastia in young adult males and factors associated with idiopathic gynaecomastia. Int J Androl **25**(5), 312-316 (2002). doi:10.1046/j.1365-2605.2002.00374.x

301. Ersöz, H., Onde, M.E., Terekeci, H., Kurtoglu, S., Tor, H.: Causes of gynaecomastia in young adult males and factors associated with idiopathic gynaecomastia. Int J Androl **25**(5), 312-316 (2002). doi:10.1046/j.1365-2605.2002.00374.x

302. Evans, S.F., Davie, M.W.: Low body size and elevated sex-hormone binding globulin distinguish men with idiopathic vertebral fracture. Calcif Tissue Int **70**(1), 9-15 (2002). doi:10.1007/s00223-001-2018-6

303. Fabbri, A., Jannini, E.A., Ulisse, S., Gnessi, L., Moretti, C., Frajese, G., Isidori, A.: Low serum bioactive luteinizing hormone in nonorganic male impotence: possible relationship with altered gonadotropin-releasing hormone pulsatility. J Clin Endocrinol Metab **67**(5), 867-875 (1988). doi:10.1210/jcem-67-5-867

304. Fabian, U.A., Charles-Davies, M.A., Fasanmade, A.A., Olaniyi, J.A., Oyewole, O.E., Owolabi, M.O., Adebusuyi, J.R., Hassan, O.O., Ajobo, B.M., Ebesunun, M.O., Adigun, K., Akinlade, K.S., Arinola, O.G., Agbedana, E.O.: Male Sexual Dysfunction, Leptin, Pituitary and Gonadal Hormones in Nigerian Males with Metabolic Syndrome and Type 2 Diabetes Mellitus. J Reprod Infertil **17**(1), 17-25 (2016).

305. Faiman, C., Winter, J.S.: Diurnal cycles in plasma FSH, testosterone and cortisol in men. J Clin Endocrinol Metab **33**(2), 186-192 (1971). doi:10.1210/jcem-33-2-186

306. Fallah, N., Mohammad, K., Nourijelyani, K., Eshraghian, M.R., Seyyedsalehi, S.A., Raiessi, M., Rahmani, M., Goodarzi, H.R., Darvish, S., Zeraati, H., Davoodi, G., Sadeghian, S.: Nonlinear association between serum testosterone levels and coronary artery disease in Iranian men. Eur J Epidemiol **24**(6), 297-306 (2009). doi:10.1007/s10654-009-9336-9

307. Fantus, R.J., Chang, C., Hehemann, M.C., Bennett, N.E., Brannigan, R.E., Helfand, B.T., Halpern, J.A.: The association between guideline-based exercise thresholds and low testosterone among men in the United States. Andrology **8**(6), 1712-1719 (2020). doi:10.1111/andr.12849

308. Fantus, R.J., Lokeshwar, S.D., Kohn, T.P., Ramasamy, R.: The effect of tetrahydrocannabinol on testosterone among men in the United States: results from the National Health and Nutrition Examination Survey. World J Urol **38**(12), 3275-3282 (2020). doi:10.1007/s00345-020-03110-5

309. Farag, A.G.A., Basha, M.A., Amin, S.A., Elnaidany, N.F., Elhelbawy, N.G., Mostafa, M.M.T., Khodier, S.A., Ibrahem, R.A., Mahfouz, R.Z.: Tramadol (opioid) abuse is associated with a dose- and time-dependent poor sperm quality and hyperprolactinaemia in young men. Andrologia **50**(6), e13026 (2018). doi:10.1111/and.13026

310. Farnsworth, W.H., Hoeg, J.M., Maher, M., Brittain, E.H., Sherins, R.J., Brewer, H.B., Jr.: Testicular function in type II hyperlipoproteinemic patients treated with lovastatin (mevinolin) or neomycin. J Clin Endocrinol Metab **65**(3), 546-550 (1987). doi:10.1210/jcem-65-3-546

311. Farzad, B., Gharakhanlou, R., Agha-Alinejad, H., Curby, D.G., Bayati, M., Bahraminejad, M., Mäestu, J.: Physiological and performance changes from the addition of a sprint interval program to wrestling training. J Strength Cond Res **25**(9), 2392-2399 (2011). doi:10.1519/JSC.0b013e3181fb4a33

312. Fauser, B.C., Bogers, J.W., Hop, W.C., De Jong, F.H.: Bioactive and immunoreactive FSH in serum of normal and oligospermic men. Clin Endocrinol (Oxf) **32**(4), 433-442 (1990). doi:10.1111/j.1365-2265.1990.tb00883.x

313. Felici, F., Bazzucchi, I., Sgrò, P., Quinzi, F., Conti, A., Aversa, A., Gizzi, L., Mezzullo, M., Romanelli, F., Pasquali, R., Lenzi, A., Di Luigi, L.: Acute severe male hypo-testosteronemia affects central motor command in humans. J Electromyogr Kinesiol **28**, 184-192 (2016). doi:10.1016/j.jelekin.2015.12.004

314. Ferlin, A., Selice, R., Angelini, S., Di Grazia, M., Caretta, N., Cavalieri, F., Di Mambro, A., Foresta, C.: Endocrine and psychological aspects of sexual dysfunction in Klinefelter patients. Andrology **6**(3), 414-419 (2018). doi:10.1111/andr.12474

315. Fernandez, M.F., Duran, I., Olea, N., Avivar, C., Vierula, M., Toppari, J., Skakkebaek, N.E., Jørgensen, N.: Semen quality and reproductive hormone levels in men from Southern Spain. Int J Androl **35**(1), 1-10 (2012). doi:10.1111/j.1365-2605.2010.01131.x

316. Ferrando, A.A., Green, N.R.: The effect of boron supplementation on lean body mass, plasma testosterone levels, and strength in male bodybuilders. Int J Sport Nutr **3**(2), 140-149 (1993). doi:10.1123/ijsn.3.2.140

317. Ferrando, A.A., Lane, H.W., Stuart, C.A., Davis-Street, J., Wolfe, R.R.: Prolonged bed rest decreases skeletal muscle and whole body protein synthesis. Am J Physiol **270**(4 Pt 1), E627-633 (1996). doi:10.1152/ajpendo.1996.270.4.E627

318. Ferrier, I.N., Cotes, P.M., Crow, T.J., Johnstone, E.C.: Gonadotropin secretion abnormalities in chronic schizophrenia. Psychol Med **12**(2), 263-273 (1982). doi:10.1017/s0033291700046596

319. Ficher, M., Zuckerman, M., Fishkin, R.E., Goldman, A., Neeb, M., Fink, P.J., Cohen, S.N., Jacobs, J.A., Weisberg, M.: Do endocrines play an etiological role in diabetic and nondiabetic sexual dysfunctions? J Androl **5**(1), 8-16 (1984). doi:10.1002/j.1939-4640.1984.tb00771.x

320. Fiers, T., Wu, F., Moghetti, P., Vanderschueren, D., Lapauw, B., Kaufman, J.M.: Reassessing Free-Testosterone Calculation by Liquid Chromatography-Tandem Mass Spectrometry Direct Equilibrium Dialysis. J Clin Endocrinol Metab **103**(6), 2167-2174 (2018). doi:10.1210/jc.2017-02360

321. Finkelstein, J.S., Lee, H., Leder, B.Z., Burnett-Bowie, S.A., Goldstein, D.W., Hahn, C.W., Hirsch, S.C., Linker, A., Perros, N., Servais, A.B., Taylor, A.P., Webb, M.L., Youngner, J.M., Yu, E.W.: Gonadal steroid-dependent effects on bone turnover and bone mineral density in men. J Clin Invest **126**(3), 1114-1125 (2016). doi:10.1172/jci84137

322. Firtser, S., Juonala, M., Magnussen, C.G., Jula, A., Loo, B.M., Marniemi, J., Viikari, J.S., Toppari, J., Perheentupa, A., Hutri-KÃ¤hÃ¶nen, N., Raitakari, O.T.: Relation of total and free testosterone and sex hormone-binding globulin with cardiovascular risk factors in men aged 24-45 years. The Cardiovascular Risk in Young Finns Study. Atherosclerosis **222**(1), 257-262 (2012). doi:10.1016/j.atherosclerosis.2012.02.020

323. Firtser, S., Juonala, M., Magnussen, C.G., Jula, A., Loo, B.M., Marniemi, J., Viikari, J.S., Toppari, J., Perheentupa, A., Hutri-Kähönen, N., Raitakari, O.T.: Relation of total and free testosterone and sex hormone-binding globulin with cardiovascular risk factors in men aged 24-45 years. The Cardiovascular Risk in Young Finns Study. Atherosclerosis **222**(1), 257-262 (2012). doi:10.1016/j.atherosclerosis.2012.02.020

324. Fischer, F., Schulte, H., Mohan, S., Tataru, M.C., KÃ¶hler, E., Assmann, G., von Eckardstein, A.: Associations of insulin-like growth factors, insulin-like growth factor binding proteins and acid-labile subunit with coronary heart disease. Clin Endocrinol (Oxf) **61**(5), 595-602 (2004). doi:10.1111/j.1365-2265.2004.02136.x

325. Fischer, F., Schulte, H., Mohan, S., Tataru, M.C., Köhler, E., Assmann, G., von Eckardstein, A.: Associations of insulin-like growth factors, insulin-like growth factor binding proteins and acid-labile subunit with coronary heart disease. Clin Endocrinol (Oxf) **61**(5), 595-602 (2004). doi:10.1111/j.1365-2265.2004.02136.x

326. Fogari, R., Zoppi, A., Preti, P., Rinaldi, A., Marasi, G., Vanasia, A., Mugellini, A.: Sexual activity and plasma testosterone levels in hypertensive males. Am J Hypertens **15**(3), 217-221 (2002). doi:10.1016/s0895-7061(01)02280-4

327. Folland, J.P., Mc Cauley, T.M., Phypers, C., Hanson, B., Mastana, S.S.: Relationship of 2D:4D finger ratio with muscle strength, testosterone, and androgen receptor CAG repeat genotype. Am J Phys Anthropol **148**(1), 81-87 (2012). doi:10.1002/ajpa.22044

328. Folland, J.P., Mc Cauley, T.M., Phypers, C., Hanson, B., Mastana, S.S.: The relationship of testosterone and AR CAG repeat genotype with knee extensor muscle function of young and older men. Exp Gerontol **47**(6), 437-443 (2012). doi:10.1016/j.exger.2012.03.013

329. Folomeev, M., Dougados, M., Beaune, J., Kouyoumdjian, J.C., Nahoul, K., Amor, B., Alekberova, Z.: Plasma sex hormones and aromatase activity in tissues of patients with systemic lupus erythematosus. Lupus **1**(3), 191-195 (1992). doi:10.1177/096120339200100312

330. Fonda, S.J., Bertrand, R., O'Donnell, A., Longcope, C., McKinlay, J.B.: Age, hormones, and cognitive functioning among middle-aged and elderly men: cross-sectional evidence from the Massachusetts Male Aging Study. J Gerontol A Biol Sci Med Sci **60**(3), 385-390 (2005). doi:10.1093/gerona/60.3.385

331. Fontana, L., Klein, S., Holloszy, J.O.: Effects of long-term calorie restriction and endurance exercise on glucose tolerance, insulin action, and adipokine production. Age (Dordr) **32**(1), 97-108 (2010). doi:10.1007/s11357-009-9118-z

332. Foresta, C., Caretta, N., Palego, P., Ferlin, A., Zuccarello, D., Lenzi, A., Selice, R.: Reduced artery diameters in Klinefelter syndrome. International journal of andrology **35**(5), 720-725 (2012). doi:10.1111/j.1365-2605.2012.01269.x

333. Foresta, C., De Toni, L., Selice, R., Garolla, A., Di Mambro, A.: Increased osteocalcin-positive endothelial progenitor cells in hypogonadal male patients. J Endocrinol Invest **33**(7), 439-442 (2010). doi:10.1007/bf03346620

334. Foresta, C., Zuccarello, D., De Toni, L., Garolla, A., Caretta, N., Ferlin, A.: Androgens stimulate endothelial progenitor cells through an androgen receptor-mediated pathway. Clin Endocrinol (Oxf) **68**(2), 284-289 (2008). doi:10.1111/j.1365-2265.2007.03036.x

335. Forti, G., Borghi, A., Giusti, G., Pazzagli, M., Giannotti, P., Mannelli, M., Fusi, S., Serio, M.: Klinefelter's syndrome: effects of short-term testosterone administration on hypothalamic-pituitary axis function. J Endocrinol Invest **1**(3), 239-244 (1978). doi:10.1007/bf03350387

336. Forti, G., Giusti, G., Borghi, A., Pazzagli, M., Fiorelli, G., Cabresi, E., Mannelli, M., Bassi, F., Giannotti, P., Fusi, S., Serio, M.: Klinefelter's syndrome: a study of its hormonal plasma pattern. Journal of endocrinological investigation **1**(2), 149-154 (1978). doi:10.1007/bf03350363

337. Francis, K.T., Hamrick, M.E.: Effects of dantrolene on adrenal cortical function. Biochem Pharmacol **29**(12), 1669-1672 (1980). doi:10.1016/0006-2952(80)90122-7

338. Francis, R.M., Peacock, M., Marshall, D.H., Horsman, A., Aaron, J.E.: Spinal osteoporosis in men. Bone Miner **5**(3), 347-357 (1989). doi:10.1016/0169-6009(89)90012-3

339. Freedman, D.S., O'Brien, T.R., Flanders, W.D., DeStefano, F., Barboriak, J.J.: Relation of serum testosterone levels to high density lipoprotein cholesterol and other characteristics in men. Arterioscler Thromb **11**(2), 307-315 (1991). doi:10.1161/01.atv.11.2.307

340. Freischem, C.W., Knuth, U.A., Langer, K., Schneider, H.P., Nieschlag, E.: The lack of discriminant seminal and endocrine variables in the partners of fertile and infertile women. Arch Gynecol **236**(1), 1-12 (1984). doi:10.1007/bf02114863

341. Friedl, K.E., Jones, R.E., Hannan, C.J., Jr., Plymate, S.R.: The administration of pharmacological doses of testosterone or 19-nortestosterone to normal men is not associated with increased insulin secretion or impaired glucose tolerance. J Clin Endocrinol Metab **68**(5), 971-975 (1989). doi:10.1210/jcem-68-5-971

342. Friedrich, N., Rosskopf, D., Brabant, G., Völzke, H., Nauck, M., Wallaschofski, H.: Associations of anthropometric parameters with serum TSH, prolactin, IGF-I, and testosterone levels: results of the study of health in Pomerania (SHIP). Exp Clin Endocrinol Diabetes **118**(4), 266-273 (2010). doi:10.1055/s-0029-1225616

343. Frokjaer, V.G., Erritzoe, D., Juul, A., Nielsen, F.A., Holst, K., Svarer, C., Madsen, J., Paulson, O.B., Knudsen, G.M.: Endogenous plasma estradiol in healthy men is positively correlated with cerebral cortical serotonin 2A receptor binding. Psychoneuroendocrinology **35**(9), 1311-1320 (2010). doi:10.1016/j.psyneuen.2010.03.002

344. Fujisawa, M., Dobashi, M., Yamasaki, T., Kanzaki, M., Okada, H., Arakawa, S., Kamidono, S.: Significance of serum inhibin B concentration for evaluating improvement in spermatogenesis after varicocelectomy. Hum Reprod **16**(9), 1945-1949 (2001). doi:10.1093/humrep/16.9.1945

345. Fujisawa, M., Yamanaka, K., Okada, H., Arakawa, S., Kamidono, S.: Growth hormone releasing hormone test for infertile men with spermatogenetic maturation arrest. J Urol **168**(5), 2083-2085 (2002). doi:10.1016/s0022-5347(05)64301-7

346. Fujisawa, M., Yamasaki, T., Okada, H., Kamidono, S.: The significance of anti-Müllerian hormone concentration in seminal plasma for spermatogenesis. Hum Reprod **17**(4), 968-970 (2002). doi:10.1093/humrep/17.4.968

347. Fujisawa, M., Yamasaki, T., Okada, H., Kamidono, S.: The significance of anti-MÃ¼llerian hormone concentration in seminal plasma for spermatogenesis. Hum Reprod **17**(4), 968-970 (2002). doi:10.1093/humrep/17.4.968

348. Fukai, S., Akishita, M., Miyao, M., Ishida, K., Toba, K., Ouchi, Y.: Age-related changes in plasma androgen levels and their association with cardiovascular risk factors in male Japanese office workers. Geriatr Gerontol Int **10**(1), 32-39 (2010). doi:10.1111/j.1447-0594.2009.00552.x

349. Fukai, S., Akishita, M., Yamada, S., Hama, T., Ogawa, S., Iijima, K., Eto, M., Kozaki, K., Toba, K., Ouchi, Y.: Association of plasma sex hormone levels with functional decline in elderly men and women. Geriatr Gerontol Int **9**(3), 282-289 (2009). doi:10.1111/j.1447-0594.2009.00534.x

350. Gaber, H.D., El-Beeh, K.A.M., Abd Al-Naser, F.A.W., Hosny, A.: Erectile dysfunction in patients with first-episode psychosis. Andrologia **52**(11), e13793 (2020). doi:10.1111/and.13793

351. Gades, N.M., Jacobson, D.J., McGree, M.E., St Sauver, J.L., Lieber, M.M., Nehra, A., Girman, C.J., Klee, G.G., Jacobsen, S.J.: The associations between serum sex hormones, erectile function, and sex drive: the Olmsted County Study of Urinary Symptoms and Health Status among Men. The journal of sexual medicine **5**(9), 2209-2220 (2008). doi:10.1111/j.1743-6109.2008.00924.x

352. Gaffney, G.R., Berlin, F.S.: Is there hypothalamic-pituitary-gonadal dysfunction in paedophilia? A pilot study. Br J Psychiatry **145**, 657-660 (1984). doi:10.1192/bjp.145.6.657

353. Gagnon, S.S., Nindl, B.C., Vaara, J.P., Santtila, M., Häkkinen, K., Kyröläinen, H., Eltaweel, A., Mustafa, A.I., El-Shimi, O.S., Algaod, F.A.: Basal Endogenous Steroid Hormones, Sex Hormone-Binding Globulin, Physical Fitness, and Health Risk Factors in Young Adult Men

Sex hormones, erectile dysfunction, and psoriasis; a bad friendship! Front Physiol **9**(12), 1005 (2018). doi:10.3389/fphys.2018.01005

10.1111/ijd.14178

354. Gahutu, J.B.: Male reproductive hormone profile in Rwandan students. Andrologia **46**(10), 1198-1199 (2014). doi:10.1111/and.12201

355. Galusca, B., Leca, V., Germain, N., Frere, D., Khalfallah, Y., Lang, F., Estour, B.: Normal inhibin B levels suggest partial preservation of gonadal function in adult male patients with anorexia nervosa. J Sex Med **9**(5), 1442-1447 (2012). doi:10.1111/j.1743-6109.2011.02514.x

356. Gamberale, F., Olson, B.A., Eneroth, P., Lindh, T., Wennberg, A.: Acute effects of ELF electromagnetic fields: a field study of linesmen working with 400 kV power lines. Br J Ind Med **46**(10), 729-737 (1989). doi:10.1136/oem.46.10.729

357. Gangwar, P.K., Sankhwar, S.N., Pant, S., Krishna, A., Singh, B.P., Mahdi, A.A., Singh, R.: Increased Gonadotropins and prolactin are linked to infertility in males. Bioinformation **16**(2), 176-182 (2020). doi:10.1016/j.archger.2020.104040

10.6026/97320630016176

358. GannagÃ©-Yared, M.H., Fares, F., Semaan, M., Khalife, S., Jambart, S.: Circulating osteoprotegerin is correlated with lipid profile, insulin sensitivity, adiponectin and sex steroids in an ageing male population. Clin Endocrinol (Oxf) **64**(6), 652-658 (2006). doi:10.1111/j.1365-2265.2006.02522.x

359. GannagÃ©-Yared, M.H., Khalife, S., Semaan, M., Fares, F., Jambart, S., Halaby, G.: Serum adiponectin and leptin levels in relation to the metabolic syndrome, androgenic profile and somatotropic axis in healthy non-diabetic elderly men. Eur J Endocrinol **155**(1), 167-176 (2006). doi:10.1530/eje.1.02175

360. Gannagé-Yared, M.H., Fares, F., Semaan, M., Khalife, S., Jambart, S.: Circulating osteoprotegerin is correlated with lipid profile, insulin sensitivity, adiponectin and sex steroids in an ageing male population. Clin Endocrinol (Oxf) **64**(6), 652-658 (2006). doi:10.1111/j.1365-2265.2006.02522.x

361. Gannagé-Yared, M.H., Khalife, S., Semaan, M., Fares, F., Jambart, S., Halaby, G.: Serum adiponectin and leptin levels in relation to the metabolic syndrome, androgenic profile and somatotropic axis in healthy non-diabetic elderly men. Eur J Endocrinol **155**(1), 167-176 (2006). doi:10.1530/eje.1.02175

362. Garcia, J.M., Li, H., Mann, D., Epner, D., Hayes, T.G., Marcelli, M., Cunningham, G.R.: Hypogonadism in male patients with cancer. Cancer **106**(12), 2583-2591 (2006). doi:10.1002/cncr.21889

363. Gates, M.A., Mekary, R.A., Chiu, G.R., Ding, E.L., Wittert, G.A., Araujo, A.B.: Sex steroid hormone levels and body composition in men. J Clin Endocrinol Metab **98**(6), 2442-2450 (2013). doi:10.1210/jc.2012-2582

364. Gawel, M.J., Park, D.M., Alaghband-Zadeh, J., Rose, F.C.: Exercise and hormonal secretion. Postgrad Med J **55**(644), 373-376 (1979). doi:10.1136/pgmj.55.644.373

365. Gettler, L.T., McDade, T.W., Kuzawa, C.W.: Cortisol and testosterone in Filipino young adult men: evidence for co-regulation of both hormones by fatherhood and relationship status. Am J Hum Biol **23**(5), 609-620 (2011). doi:10.1002/ajhb.21187

366. Ghanadian, R., Lewis, J.G., Chisholm, G.D., O'Donoghue, E.P.: Serum dihydrotestosterone in patients with benign prostatic hypertrophy. Br J Urol **49**(6), 541-544 (1977). doi:10.1111/j.1464-410x.1977.tb04202.x

367. Ghanadian, R., Puah, C.M., Williams, G., Shah, P.J., McWhinney, N.: Suppressive effects of surgical stress on circulating androgens during and after prostatectomy. Br J Urol **53**(2), 147-149 (1981). doi:10.1111/j.1464-410x.1981.tb03155.x

368. Giagulli, V.A., Carbone, D.: Hormonal control of inhibin B in men. J Endocrinol Invest **29**(8), 706-713 (2006). doi:10.1007/bf03344180

369. Giagulli, V.A., Kaufman, J.M., Vermeulen, A.: Pathogenesis of the decreased androgen levels in obese men. J Clin Endocrinol Metab **79**(4), 997-1000 (1994). doi:10.1210/jcem.79.4.7962311

370. Giagulli, V.A., Vermeulen, A.: Leydig cell function in infertile men with idiopathic oligospermic infertility. J Clin Endocrinol Metab **66**(1), 62-67 (1988). doi:10.1210/jcem-66-1-62

371. Giannouli, C., Goulis, D.G., Lambropoulos, A., Lissens, W., Tarlatzis, B.C., Bontis, J.N., Papadimas, J.: Idiopathic non-obstructive azoospermia or severe oligozoospermia: a cross-sectional study in 61 Greek men. Int J Androl **27**(2), 101-107 (2004). doi:10.1046/j.1365-2605.2003.00456.x

372. Gibb, F.W., Homer, N.Z., Faqehi, A.M., Upreti, R., Livingstone, D.E., McInnes, K.J., Andrew, R., Walker, B.R.: Aromatase Inhibition Reduces Insulin Sensitivity in Healthy Men. J Clin Endocrinol Metab **101**(5), 2040-2046 (2016). doi:10.1210/jc.2015-4146

373. Gill, J.K., Wilkens, L.R., Pollak, M.N., Stanczyk, F.Z., Kolonel, L.N.: Androgens, growth factors, and risk of prostate cancer: the Multiethnic Cohort. Prostate **70**(8), 906-915 (2010). doi:10.1002/pros.21125

374. Gillberg, P., Olofsson, H., Mallmin, H., Blum, W.F., Ljunghall, S., Nilsson, A.G.: Bone mineral density in femoral neck is positively correlated to circulating insulin-like growth factor (IGF)-I and IGF-binding protein (IGFBP)-3 in Swedish men. Calcif Tissue Int **70**(1), 22-29 (2002). doi:10.1007/s002230020048

375. Gioia, A., Ceccoli, L., Ronconi, V., Turchi, F., Marcheggiani, M., Boscaro, M., Giacchetti, G., Balercia, G.: Vitamin D levels and bone mineral density: are LH levels involved in the pathogenesis of bone impairment in hypogonadal men? J Endocrinol Invest **37**(12), 1225-1231 (2014). doi:10.1007/s40618-014-0187-1

376. Giotakos, O., Markianos, M., Vaidakis, N., Christodoulou, G.N.: Sex hormones and biogenic amine turnover of sex offenders in relation to their temperament and character dimensions. Psychiatry Res **127**(3), 185-193 (2004). doi:10.1016/j.psychres.2003.06.003

377. Girgis, S.M., Abdalla, M.I., Ibrahim, A.A., Ibrahim, II, Osman, M.I., Byad, M.A., Tawadros, G.: FSH, LH, E2, and T in semen and serum in patients with idiopathic oligozoospermia. Arch Androl **7**(4), 293-296 (1981). doi:10.3109/01485018108999320

378. Gladue, B.A., Green, R., Hellman, R.E.: Neuroendocrine response to estrogen and sexual orientation. Science **225**(4669), 1496-1499 (1984). doi:10.1126/science.6089349

379. Glover, F.E., Caudle, W.M., Del Giudice, F., Belladelli, F., Mulloy, E., Lawal, E., Eisenberg, M.L.: The association between caffeine intake and testosterone: NHANES 2013-2014. Nutrition journal **21**(1), 33 (2022). doi:10.1186/s12937-022-00783-z

380. Goh, V.H., Tong, T.Y.: The moderating impact of lifestyle factors on sex steroids, sexual activities and aging in Asian men. Asian J Androl **13**(4), 596-604 (2011). doi:10.1038/aja.2010.121

381. Gökçen, K., Kılıçarslan, H., Coşkun, B., Ersoy, A., Kaygısız, O., Kordan, Y.: Effect of ADMA levels on severity of erectile dysfunction in chronic kidney disease and other risk factors. Can Urol Assoc J **10**(1-2), E41-45 (2016). doi:10.5489/cuaj.3170

382. Goldman, J., Wajchenberg, B.L., Liberman, B., Nery, M., Achando, S., Germek, O.A.: Contrast analysis for the evaluation of the circadian rhythms of plasma cortisol, androstenedione, and testosterone in normal men and the possible influence of meals. J Clin Endocrinol Metab **60**(1), 164-167 (1985). doi:10.1210/jcem-60-1-164

383. Gomez-Merino, D., Chennaoui, M., Drogou, C., Bonneau, D., Guezennec, C.Y.: Decrease in serum leptin after prolonged physical activity in men. Med Sci Sports Exerc **34**(10), 1594-1599 (2002). doi:10.1097/00005768-200210000-00010

384. Gomez-Merino, D., Drogou, C., Chennaoui, M., Tiollier, E., Mathieu, J., Guezennec, C.Y.: Effects of combined stress during intense training on cellular immunity, hormones and respiratory infections. Neuroimmunomodulation **12**(3), 164-172 (2005). doi:10.1159/000084849

385. Goncharov, A., Rej, R., Negoita, S., Schymura, M., Santiago-Rivera, A., Morse, G., Carpenter, D.O.: Lower serum testosterone associated with elevated polychlorinated biphenyl concentrations in Native American men. Environ Health Perspect **117**(9), 1454-1460 (2009). doi:10.1289/ehp.0800134

386. Gonen, M.S., De Bellis, A., Durcan, E., Bellastella, G., Cirillo, P., Scappaticcio, L., Longo, M., Bircan, B.E., Sahin, S., Sulu, C., Ozkaya, H.M., Konukoglu, D., Kartufan, F.F., Kelestimur, F.: Assessment of Neuroendocrine Changes and Hypothalamo-Pituitary Autoimmunity in Patients with COVID-19. Horm Metab Res **54**(3), 153-161 (2022). doi:10.1016/j.scitotenv.2022.154395

10.1055/a-1764-1260

387. Gonzales, G.F., Lozano-Hernández, R., Gasco, M., Gonzales-Castañeda, C., Tapia, V.: Resistance of sperm motility to serum testosterone in men with excessive erythrocytosis at high altitude. Horm Metab Res **44**(13), 987-992 (2012). doi:10.1055/s-0032-1321854

388. Gonzales, G.F., Tapia, V., Gasco, M., Gonzales-Castañeda, C.: Serum testosterone levels and score of chronic mountain sickness in Peruvian men natives at 4340 m. Andrologia **43**(3), 189-195 (2011). doi:10.1111/j.1439-0272.2010.01046.x

389. Gonzales, G.F., Tapia, V., Gasco, M., Gonzales-Castañeda, C.: Aromatase activity after a short-course of letrozole administration in adult men at sea level and at high altitude (with or without excessive erythrocytosis). Horm Metab Res **44**(2), 140-145 (2012). doi:10.1055/s-0031-1301280

390. Gonzales, G.F., Tapia, V., Gasco, M., Rubio, J., Gonzales-Castañeda, C.: High serum zinc and serum testosterone levels were associated with excessive erythrocytosis in men at high altitudes. Endocrine **40**(3), 472-480 (2011). doi:10.1007/s12020-011-9482-1

391. Gonzalo, I.T., Swerdloff, R.S., Nelson, A.L., Clevenger, B., Garcia, R., Berman, N., Wang, C.: Levonorgestrel implants (Norplant II) for male contraception clinical trials: combination with transdermal and injectable testosterone. J Clin Endocrinol Metab **87**(8), 3562-3572 (2002). doi:10.1210/jcem.87.8.8710

392. Gooren, L.J., van der Veen, E.A., van Kessel, H., Harmsen-Louman, W.: Estrogens in the feedback regulation of gonadotropin secretion in men: effects of administration of estrogen to agonadal subjects and the antiestrogen tamoxifen and the aromatase inhibitor delta'-testolactone to eugonadal subjects. Andrologia **16**(6), 568-577 (1984). doi:10.1111/j.1439-0272.1984.tb00414.x

393. Gorostiaga, E.M., Izquierdo, M., Iturralde, P., Ruesta, M., IbÃ¡Ã±ez, J.: Effects of heavy resistance training on maximal and explosive force production, endurance and serum hormones in adolescent handball players. Eur J Appl Physiol Occup Physiol **80**(5), 485-493 (1999). doi:10.1007/s004210050622

394. Gorostiaga, E.M., Izquierdo, M., Iturralde, P., Ruesta, M., Ibáñez, J.: Effects of heavy resistance training on maximal and explosive force production, endurance and serum hormones in adolescent handball players. Eur J Appl Physiol Occup Physiol **80**(5), 485-493 (1999). doi:10.1007/s004210050622

395. Goto, A., Morita, A., Goto, M., Sasaki, S., Miyachi, M., Aiba, N., Terauchi, Y., Noda, M., Watanabe, S.: Associations of sex hormone-binding globulin and testosterone with diabetes among men and women (the Saku Diabetes study): a case control study. Cardiovasc Diabetol **11**, 130 (2012). doi:10.1186/1475-2840-11-130

396. Goudriaan, A.E., Lapauw, B., Ruige, J., Feyen, E., Kaufman, J.M., Brand, M., Vingerhoets, G.: The influence of high-normal testosterone levels on risk-taking in healthy males in a 1-week letrozole administration study. Psychoneuroendocrinology **35**(9), 1416-1421 (2010). doi:10.1016/j.psyneuen.2010.04.005

397. Goutou, M., Sakka, C., Stakias, N., Stefanidis, I., Koukoulis, G.N.: AR CAG repeat length is not associated with serum gonadal steroids and lipid levels in healthy men. Int J Androl **32**(6), 616-622 (2009). doi:10.1111/j.1365-2605.2008.00908.x

398. Gräf, K.J., Schmidt-Gollwitzer, M., Horowski, R., Dorow, R.: Effect of metoclopramide and lisuride on hypophyseal and gonadal function in men. Clin Endocrinol (Oxf) **17**(3), 243-251 (1982). doi:10.1111/j.1365-2265.1982.tb01586.x

399. Graff, R.E., Meisner, A., Ahearn, T.U., Fiorentino, M., Loda, M., Giovannucci, E.L., Mucci, L.A., Pettersson, A.: Pre-diagnostic circulating sex hormone levels and risk of prostate cancer by ERG tumour protein expression. Br J Cancer **114**(8), 939-944 (2016). doi:10.1038/bjc.2016.61

400. Grajewski, B., Whelan, E.A., Schnorr, T.M., Mouradian, R., Alderfer, R., Wild, D.K.: Evaluation of reproductive function among men occupationally exposed to a stilbene derivative: I. Hormonal and physical status. Am J Ind Med **29**(1), 49-57 (1996). doi:10.1002/(sici)1097-0274(199601)29:1<49::Aid-ajim7>3.0.Co;2-u

401. Grandys, M., Majerczak, J., Duda, K., Zapart-Bukowska, J., Kulpa, J., Zoladz, J.A.: Endurance training of moderate intensity increases testosterone concentration in young, healthy men. Int J Sports Med **30**(7), 489-495 (2009). doi:10.1055/s-0029-1202340

402. Grandys, M., Majerczak, J., Zapart-Bukowska, J., Kulpa, J., Zoladz, J.A.: Gonadal hormone status in highly trained sprinters and in untrained men. J Strength Cond Res **25**(4), 1079-1084 (2011). doi:10.1519/JSC.0b013e3181d4d3f4

403. Gray, A.B., Telford, R.D., Weidemann, M.J.: Endocrine response to intense interval exercise. Eur J Appl Physiol Occup Physiol **66**(4), 366-371 (1993). doi:10.1007/bf00237784

404. Gray, P., Franken, D.R., Slabber, C.F., Potgieter, G.M.: A comparison of endocrine function and semen analysis in fertile and subfertile men. Andrologia **13**(3), 260-264 (1981). doi:10.1111/j.1439-0272.1981.tb00046.x

405. Greene, F.J., Han, L., Martin, S., Zhang, S., Wittert, G.: Testosterone is associated with self-employment among Australian men. Econ Hum Biol **13**, 76-84 (2014). doi:10.1016/j.ehb.2013.02.003

406. Greimel, E., Herpertz-Dahlmann, B., Günther, T., Vitt, C., Konrad, K.: Attentional functions in children and adolescents with attention-deficit/hyperactivity disorder with and without comorbid tic disorder. J Neural Transm (Vienna) **115**(2), 191-200 (2008). doi:10.1007/s00702-007-0815-4

407. Griffiths, A.W., Marks, V., Fry, D., Morley, G., Lewis, G.: Prisoners of XYY constitution: biochemical studies. Br J Psychiatry **121**(563), 365-368 (1972). doi:10.1192/bjp.121.4.365

408. Grigorova, M., Punab, M., Ausmees, K., Laan, M.: FSHB promoter polymorphism within evolutionary conserved element is associated with serum FSH level in men. Human reproduction (Oxford, England) **23**(9), 2160-2166 (2008). doi:10.1093/humrep/den216

409. Grigorova, M., Punab, M., Punab, A.M., Poolamets, O., Vihljajev, V., ZilaitienÄ—, B., Erenpreiss, J., MatuleviÄius, V., Laan, M.: Reproductive physiology in young men is cumulatively affected by FSH-action modulating genetic variants: FSHR -29G/A and c.2039 A/G, FSHB -211G/T. PLoS One **9**(4), e94244 (2014). doi:10.1371/journal.pone.0094244

410. Grigorova, M., Punab, M., Punab, A.M., Poolamets, O., Vihljajev, V., Zilaitienė, B., Erenpreiss, J., Matulevičius, V., Laan, M.: Reproductive physiology in young men is cumulatively affected by FSH-action modulating genetic variants: FSHR -29G/A and c.2039 A/G, FSHB -211G/T. PLoS One **9**(4), e94244 (2014). doi:10.1371/journal.pone.0094244

411. Grodum, E., Andersen, M., Hangaard, J., Koldkjaer, O., Hagen, C.: Lack of effect of the dopamine D1 antagonist, NNC 01-0687, on unstimulated and stimulated release of anterior pituitary hormones in males. J Endocrinol Invest **21**(5), 291-297 (1998). doi:10.1007/bf03350331

412. Groepenhoff, F., Diez Benavente, E., Boltjes, A., Timmerman, N., Waissi, F., Hartman, R.J.G., Onland-Moret, N.C., Pasterkamp, G., Den Ruijter, H., Wu, K.C., Ewing, S.K., Li, X., Sigurðsson, S., Guðnason, V., Kado, D.M., Hue, T.F., Woods, G.N., Veldhuis-Vlug, A.G., Vittinghoff, E., Zaidi, M., Rosen, C.J., Lang, T., Kim, T.Y., Schwartz, A.V., Schafer, A.L.: Plasma Testosterone Levels and Atherosclerotic Plaque Gene Expression in Men With Advanced Atherosclerosis

FSH Level and Changes in Bone Mass and Body Composition in Older Women and Men. Front Cardiovasc Med **8**(10), 693351 (2021). doi:10.3389/fcvm.2021.693351

10.1210/clinem/dgab481

413. Gruschwitz, M.S., Brezinschek, R., Brezinschek, H.P.: Cytokine levels in the seminal plasma of infertile males. J Androl **17**(2), 158-163 (1996).

414. Gu, B., Wang, S., Liu, F., Song, Y., Li, J., Ni, Y., Chen, M., Hu, J., Ouzhu, L., Li, Z., Liu, L., Li, X., Liu, X.: Same total normal forms sperm counts of males from Lhasa and Shanghai, China. Environ Sci Pollut Res Int **29**(13), 18820-18831 (2022). doi:10.1038/s41598-021-99571-8

10.1007/s11356-021-17083-4

415. Guadalupe-Grau, A., Larsen, S., Guerra, B., Calbet, J.A., Dela, F., Helge, J.W.: Influence of age on leptin induced skeletal muscle signalling. Acta Physiol (Oxf) **211**(1), 214-228 (2014). doi:10.1111/apha.12273

416. Gubbels, C.S., Welt, C.K., Dumoulin, J.C., Robben, S.G., Gordon, C.M., Dunselman, G.A., Rubio-Gozalbo, M.E., Berry, G.T.: The male reproductive system in classic galactosemia: cryptorchidism and low semen volume. J Inherit Metab Dis **36**(5), 779-786 (2013). doi:10.1007/s10545-012-9539-1

417. Gupta, K., Gill, G.S., Mahajan, R., Punab, M., Poolamets, O., Paju, P., Vihljajev, V., Pomm, K., Ladva, R., Korrovits, P., Laan, M.: Possible role of elevated serum testosterone in pathogenesis of renal stone formation

Causes of male infertility: a 9-year prospective monocentre study on 1737 patients with reduced total sperm counts. Int J Appl Basic Med Res **6**(4), 241-244 (2016). doi:10.4103/2229-516x.192593

418. Gustafson, A., Hedner, P., Schütz, A., Skerfving, S.: Occupational lead exposure and pituitary function. Int Arch Occup Environ Health **61**(4), 277-281 (1989). doi:10.1007/bf00381426

419. Gutorova, N.V., Kleshchyov, M.A., Tipisova, E.V., Osadchuk, L.V.: Effects of overweight and obesity on the spermogram values and levels of reproductive hormones in the male population of the European north of Russia. Bull Exp Biol Med **157**(1), 95-98 (2014). doi:10.1007/s10517-014-2500-6

420. Gyawali, P., Martin, S.A., Heilbronn, L.K., Vincent, A.D., Jenkins, A.J., Januszewski, A.S., Taylor, A.W., Adams, R.J.T., O'Loughlin, P.D., Wittert, G.A.: Cross-sectional and longitudinal determinants of serum sex hormone binding globulin (SHBG) in a cohort of community-dwelling men. PLoS One **13**(7), e0200078 (2018). doi:10.1371/journal.pone.0200078

421. Gyawali, P., Martin, S.A., Heilbronn, L.K., Vincent, A.D., Taylor, A.W., Adams, R.J.T., O'Loughlin, P.D., Wittert, G.A.: The role of sex hormone-binding globulin (SHBG), testosterone, and other sex steroids, on the development of type 2 diabetes in a cohort of community-dwelling middle-aged to elderly men. Acta diabetologica **55**(8), 861-872 (2018). doi:10.1007/s00592-018-1163-6

422. HÃ¤kkinen, K., Pakarinen, A.: Serum hormones in male strength athletes during intensive short term strength training. Eur J Appl Physiol Occup Physiol **63**(3-4), 194-199 (1991). doi:10.1007/bf00233847

423. HÃ¤kkinen, K., Pakarinen, A.: Acute hormonal responses to two different fatiguing heavy-resistance protocols in male athletes. J Appl Physiol (1985) **74**(2), 882-887 (1993). doi:10.1152/jappl.1993.74.2.882

424. HÃ¤kkinen, K., Pakarinen, A.: Muscle strength and serum testosterone, cortisol and SHBG concentrations in middle-aged and elderly men and women. Acta Physiol Scand **148**(2), 199-207 (1993). doi:10.1111/j.1748-1716.1993.tb09549.x

425. HÃ¤kkinen, K., Pakarinen, A.: Acute hormonal responses to heavy resistance exercise in men and women at different ages. Int J Sports Med **16**(8), 507-513 (1995). doi:10.1055/s-2007-973045

426. HÃ¤kkinen, K., Pakarinen, A., Kraemer, W.J., Newton, R.U., Alen, M.: Basal concentrations and acute responses of serum hormones and strength development during heavy resistance training in middle-aged and elderly men and women. J Gerontol A Biol Sci Med Sci **55**(2), B95-105 (2000). doi:10.1093/gerona/55.2.b95

427. HÃ¤mÃ¤lÃ¤inen, E., Tikkanen, H., HÃ¤rkÃ¶nen, M., NÃ¤veri, H., Adlercreutz, H.: Serum lipoproteins, sex hormones and sex hormone binding globulin in middle-aged men of different physical fitness and risk of coronary heart disease. Atherosclerosis **67**(2-3), 155-162 (1987). doi:10.1016/0021-9150(87)90275-9

428. Habito, R.C., Montalto, J., Leslie, E., Ball, M.J.: Effects of replacing meat with soyabean in the diet on sex hormone concentrations in healthy adult males. Br J Nutr **84**(4), 557-563 (2000). doi:10.1017/s0007114500001872

429. Hackney, A.C., Fahrner, C.L., Stupnicki, R.: Reproductive hormonal responses to maximal exercise in endurance-trained men with low resting testosterone levels. Exp Clin Endocrinol Diabetes **105**(5), 291-295 (1997). doi:10.1055/s-0029-1211767

430. Hackney, A.C., Sinning, W.E., Bruot, B.C.: Reproductive hormonal profiles of endurance-trained and untrained males. Med Sci Sports Exerc **20**(1), 60-65 (1988). doi:10.1249/00005768-198802000-00009

431. Hackney, A.C., Sinning, W.E., Bruot, B.C.: Hypothalamic-pituitary-testicular axis function in endurance-trained males. Int J Sports Med **11**(4), 298-303 (1990). doi:10.1055/s-2007-1024811

432. Hadlow, N.C., Brown, S.J., Lim, E.M., Prentice, D., Pettigrew, S., Cronin, S.L., Prescott, S.L., Silva, D., Yeap, B.B.: Anti-Müllerian hormone concentration is associated with central adiposity and reproductive hormones in expectant fathers. Clin Endocrinol (Oxf) **97**(5), 634-642 (2022). doi:10.1111/cen.14725

433. Haffner, S.M., KarhapÃ¤Ã¤, P., MykkÃ¤nen, L., Laakso, M.: Insulin resistance, body fat distribution, and sex hormones in men. Diabetes **43**(2), 212-219 (1994). doi:10.2337/diab.43.2.212

434. Haffner, S.M., Laakso, M., Miettinen, H., MykkÃ¤nen, L., KarhapÃ¤Ã¤, P., Rainwater, D.L.: Low levels of sex hormone-binding globulin and testosterone are associated with smaller, denser low density lipoprotein in normoglycemic men. J Clin Endocrinol Metab **81**(10), 3697-3701 (1996). doi:10.1210/jcem.81.10.8855825

435. Haffner, S.M., Laakso, M., Miettinen, H., Mykkänen, L., Karhapää, P., Rainwater, D.L.: Low levels of sex hormone-binding globulin and testosterone are associated with smaller, denser low density lipoprotein in normoglycemic men. J Clin Endocrinol Metab **81**(10), 3697-3701 (1996). doi:10.1210/jcem.81.10.8855825

436. Haffner, S.M., Miettinen, H., KarhapÃ¤Ã¤, P., MykkÃ¤nen, L., Laakso, M.: Leptin concentrations, sex hormones, and cortisol in nondiabetic men. J Clin Endocrinol Metab **82**(6), 1807-1809 (1997). doi:10.1210/jcem.82.6.3978

437. Haffner, S.M., Miettinen, H., Karhapää, P., Mykkänen, L., Laakso, M.: Leptin concentrations, sex hormones, and cortisol in nondiabetic men. J Clin Endocrinol Metab **82**(6), 1807-1809 (1997). doi:10.1210/jcem.82.6.3978

438. Haffner, S.M., MykkÃ¤nen, L., Valdez, R.A., Katz, M.S.: Relationship of sex hormones to lipids and lipoproteins in nondiabetic men. J Clin Endocrinol Metab **77**(6), 1610-1615 (1993). doi:10.1210/jcem.77.6.8263149

439. Haffner, S.M., Mykkänen, L., Valdez, R.A., Katz, M.S.: Relationship of sex hormones to lipids and lipoproteins in nondiabetic men. J Clin Endocrinol Metab **77**(6), 1610-1615 (1993). doi:10.1210/jcem.77.6.8263149

440. Haffner, S.M., Mykkänen, L., Valdez, R.A., Stern, M.P.: Evaluation of two insulin assays in insulin resistance syndrome (syndrome X). Arterioscler Thromb **14**(9), 1430-1437 (1994). doi:10.1161/01.atv.14.9.1430

441. Haffner, S.M., Shaten, J., Stern, M.P., Smith, G.D., Kuller, L.: Low levels of sex hormone-binding globulin and testosterone predict the development of non-insulin-dependent diabetes mellitus in men. MRFIT Research Group. Multiple Risk Factor Intervention Trial. Am J Epidemiol **143**(9), 889-897 (1996). doi:10.1093/oxfordjournals.aje.a008832

442. Haffner, S.M., Valdez, R.A., MykkÃ¤nen, L., Stern, M.P., Katz, M.S.: Decreased testosterone and dehydroepiandrosterone sulfate concentrations are associated with increased insulin and glucose concentrations in nondiabetic men. Metabolism **43**(5), 599-603 (1994). doi:10.1016/0026-0495(94)90202-x

443. Haffner, S.M., Valdez, R.A., Mykkänen, L., Stern, M.P., Katz, M.S.: Decreased testosterone and dehydroepiandrosterone sulfate concentrations are associated with increased insulin and glucose concentrations in nondiabetic men. Metabolism **43**(5), 599-603 (1994). doi:10.1016/0026-0495(94)90202-x

444. Häkkinen, K., Pakarinen, A.: Muscle strength and serum testosterone, cortisol and SHBG concentrations in middle-aged and elderly men and women. Acta Physiol Scand **148**(2), 199-207 (1993). doi:10.1111/j.1748-1716.1993.tb09549.x

445. Häkkinen, K., Pakarinen, A., Alén, M., Kauhanen, H., Komi, P.V.: Relationships between training volume, physical performance capacity, and serum hormone concentrations during prolonged training in elite weight lifters. Int J Sports Med **8 Suppl 1**, 61-65 (1987). doi:10.1055/s-2008-1025705

446. Häkkinen, K., Pakarinen, A., Kraemer, W.J., Newton, R.U., Alen, M.: Basal concentrations and acute responses of serum hormones and strength development during heavy resistance training in middle-aged and elderly men and women. J Gerontol A Biol Sci Med Sci **55**(2), B95-105 (2000). doi:10.1093/gerona/55.2.b95

447. Halim, M.M., Meyrick, G., Jeans, W.D., Murphy, D., Burton, J.L.: Myocardial infarction, androgen and the skin. Br J Dermatol **98**(1), 63-68 (1978). doi:10.1111/j.1365-2133.1978.tb07334.x

448. Hall, S.A., Araujo, A.B., Esche, G.R., Williams, R.E., Clark, R.V., Travison, T.G., McKinlay, J.B.: Treatment of symptomatic androgen deficiency: results from the Boston Area Community Health Survey. Arch Intern Med **168**(10), 1070-1076 (2008). doi:10.1001/archinte.168.10.1070

449. Hall, S.A., Page, S.T., Travison, T.G., Montgomery, R.B., Link, C.L., McKinlay, J.B.: Do statins affect androgen levels in men? Results from the Boston area community health survey. Cancer Epidemiol Biomarkers Prev **16**(8), 1587-1594 (2007). doi:10.1158/1055-9965.Epi-07-0306

450. Halmenschlager, G., Rhoden, E.L., Riedner, C.E.: The influence of age on bioavailable and free testosterone is independent of body mass index and glucose levels. World J Urol **29**(4), 541-546 (2011). doi:10.1007/s00345-011-0724-x

451. Halmenschlager, G., Rhoden, E.L., Riedner, C.E.: Calculated free testosterone and radioimmunoassay free testosterone as a predictor of subnormal levels of total testosterone. Int Urol Nephrol **44**(3), 673-681 (2012). doi:10.1007/s11255-011-0066-z

452. Halpern, J.A., Fantus, R.J., Chang, C., Keeter, M.K., Helfand, B., Bennett, N.E., Brannigan, R.E.: Effects of nonsteroidal anti-inflammatory drug (NSAID) use upon male gonadal function: A national, population-based study. Andrologia **52**(4), e13542 (2020). doi:10.1111/and.13542

453. Hämäläinen, E., Adlercreutz, H., Ehnholm, C., Puska, P.: Relationships of serum lipoproteins and apoproteins to sex hormones and to the binding capacity of sex hormone binding globulin in healthy Finnish men. Metabolism **35**(6), 535-541 (1986). doi:10.1016/0026-0495(86)90011-9

454. Hämäläinen, E., Adlercreutz, H., Puska, P., Pietinen, P.: Diet and serum sex hormones in healthy men. J Steroid Biochem **20**(1), 459-464 (1984). doi:10.1016/0022-4731(84)90254-1

455. Hämäläinen, E., Tikkanen, H., Härkönen, M., Näveri, H., Adlercreutz, H.: Serum lipoproteins, sex hormones and sex hormone binding globulin in middle-aged men of different physical fitness and risk of coronary heart disease. Atherosclerosis **67**(2-3), 155-162 (1987). doi:10.1016/0021-9150(87)90275-9

456. Hämäläinen, E.K., Adlercreutz, H., Puska, P., Pietinen, P.: Decrease of serum total and free testosterone during a low-fat high-fibre diet. J Steroid Biochem **18**(3), 369-370 (1983). doi:10.1016/0022-4731(83)90117-6

457. Hamdi, S.M., Walschaerts, M., Bujan, L., Rostaing, L., Kamar, N.: A prospective study in male recipients of kidney transplantation reveals divergent patterns for inhibin B and testosterone secretions. Basic Clin Androl **24**, 11 (2014). doi:10.1186/2051-4190-24-11

458. Hampl, R., Lachman, M., Novák, Z., Sulcová, J., Stárka, L.: Serum levels of steroid hormones in men with varicocele and oligospermia as compared to normozoospermic men. Exp Clin Endocrinol **100**(3), 117-119 (1992). doi:10.1055/s-0029-1211189

459. Handelsman, D.J., Conway, A.J., Boylan, L.M., Turtle, J.R.: Testicular function in potential sperm donors: normal ranges and the effects of smoking and varicocele. Int J Androl **7**(5), 369-382 (1984). doi:10.1111/j.1365-2605.1984.tb00794.x

460. Handelsman, D.J., Conway, A.J., Boylan, L.M., van Nunen, S.A.: Testicular function and fertility in men with homozygous alpha-1 antitrypsin deficiency. Andrologia **18**(4), 406-412 (1986). doi:10.1111/j.1439-0272.1986.tb01799.x

461. Handelsman, D.J., Spaliviero, J.A., Turtle, J.R.: Bioactive luteinizing hormone in plasma of uraemic men and men with primary testicular damage. Clin Endocrinol (Oxf) **24**(3), 259-266 (1986). doi:10.1111/j.1365-2265.1986.tb03266.x

462. Handelsman, D.J., Wishart, S., Conway, A.J.: Oestradiol enhances testosterone-induced suppression of human spermatogenesis. Hum Reprod **15**(3), 672-679 (2000). doi:10.1093/humrep/15.3.672

463. Hansen, S., Kvorning, T., Kjaer, M., SjÃ¸gaard, G.: The effect of short-term strength training on human skeletal muscle: the importance of physiologically elevated hormone levels. Scand J Med Sci Sports **11**(6), 347-354 (2001). doi:10.1034/j.1600-0838.2001.110606.x

464. Hansen, S., Kvorning, T., Kjaer, M., Sjøgaard, G.: The effect of short-term strength training on human skeletal muscle: the importance of physiologically elevated hormone levels. Scand J Med Sci Sports **11**(6), 347-354 (2001). doi:10.1034/j.1600-0838.2001.110606.x

465. Haring, R., VÃ¶lzke, H., Spielhagen, C., Nauck, M., Wallaschofski, H.: The role of sex hormone-binding globulin and testosterone in the risk of incident metabolic syndrome. Eur J Prev Cardiol **20**(6), 1061-1068 (2013). doi:10.1177/2047487312452965

466. Haring, R., Völzke, H., Spielhagen, C., Nauck, M., Wallaschofski, H.: The role of sex hormone-binding globulin and testosterone in the risk of incident metabolic syndrome. Eur J Prev Cardiol **20**(6), 1061-1068 (2013). doi:10.1177/2047487312452965

467. Harper, P., Penny, R., Foley, T.P., Jr., Migeon, C.J., Blizzard, R.M.: Gonadal function in males with myotonic dystrophy. J Clin Endocrinol Metab **35**(6), 852-856 (1972). doi:10.1210/jcem-35-6-852

468. Hartman, T.J., Dorgan, J.F., Woodson, K., Virtamo, J., Tangrea, J.A., Heinonen, O.P., Taylor, P.R., Barrett, M.J., Albanes, D.: Effects of long-term alpha-tocopherol supplementation on serum hormones in older men. Prostate **46**(1), 33-38 (2001). doi:10.1002/1097-0045(200101)46:1<33::aid-pros1005>3.0.co;2-6

469. Hartoma, R.: Serum testosterone compared with serum zinc in man. Acta Physiol Scand **101**(3), 336-341 (1977). doi:10.1111/j.1748-1716.1977.tb06015.x

470. Hasegawa, K., Matsushita, Y., Hirai, K., Otomo, S., Okamoto, T., Morii, H., Wada, M.: Abnormal response of luteinizing hormone. Follicle stimulating hormone and testosterone to luteinizing hormone-releasing hormone in chronic renal failure. Acta Endocrinol (Copenh) **87**(3), 467-475 (1978). doi:10.1530/acta.0.0870467

471. Hatami, H., Parizadeh, D., Bidhendi Yarandi, R., Tohidi, M., Ramezani Tehrani, F.: Endogenous testosterone does not improve prediction of incident cardiovascular disease in a community-based cohort of adult men: results from the Tehran Lipid and Glucose Study. Aging Male **23**(4), 243-250 (2020). doi:10.1080/13685538.2018.1466876

472. Hauner, H., Stangl, K., Burger, K., Busch, U., Blömer, H., Pfeiffer, E.F.: Sex hormone concentrations in men with angiographically assessed coronary artery disease--relationship to obesity and body fat distribution. Klin Wochenschr **69**(14), 664-668 (1991). doi:10.1007/bf01649428

473. Hautanen, A., MÃ¤nttÃ¤ri, M., Manninen, V., Tenkanen, L., Huttunen, J.K., Frick, M.H., Adlercreutz, H.: Adrenal androgens and testosterone as coronary risk factors in the Helsinki Heart Study. Atherosclerosis **105**(2), 191-200 (1994). doi:10.1016/0021-9150(94)90049-3

474. Hautanen, A., Mänttäri, M., Manninen, V., Tenkanen, L., Huttunen, J.K., Frick, M.H., Adlercreutz, H.: Adrenal androgens and testosterone as coronary risk factors in the Helsinki Heart Study. Atherosclerosis **105**(2), 191-200 (1994). doi:10.1016/0021-9150(94)90049-3

475. Hautanen, A., Sarna, S., Pelkonen, R., Adlercreutz, H.: Serum sex hormone-binding globulin, cardiovascular risk factors, and adrenal cortisol responses to dexamethasone and corticotropin. Metabolism **42**(7), 870-874 (1993). doi:10.1016/0026-0495(93)90062-s

476. Hayami, S., Sasagawa, I., Nakada, T.: Influence of sex hormones on prostate volume in men on hemodialysis. J Androl **21**(2), 258-261 (2000).

477. Hayes, F.J., Seminara, S.B., Decruz, S., Boepple, P.A., Crowley, W.F., Jr.: Aromatase inhibition in the human male reveals a hypothalamic site of estrogen feedback. J Clin Endocrinol Metab **85**(9), 3027-3035 (2000). doi:10.1210/jcem.85.9.6795

478. Hayes, L.D., Herbert, P., Sculthorpe, N.F., Grace, F.M.: Exercise training improves free testosterone in lifelong sedentary aging men. Endocr Connect **6**(5), 306-310 (2017). doi:10.1530/ec-17-0082

479. Hayes, L.D., Sculthorpe, N., Herbert, P., Baker, J.S., Hullin, D.A., Kilduff, L.P., Grace, F.M.: Resting steroid hormone concentrations in lifetime exercisers and lifetime sedentary males. Aging Male **18**(1), 22-26 (2015). doi:10.3109/13685538.2014.977246

480. Hayes, L.D., Sculthorpe, N., Herbert, P., Baker, J.S., Spagna, R., Grace, F.M.: Six weeks of conditioning exercise increases total, but not free testosterone in lifelong sedentary aging men. Aging Male **18**(3), 195-200 (2015). doi:10.3109/13685538.2015.1046123

481. Heald, A.H., Ivison, F., Anderson, S.G., Cruickshank, K., Laing, I., Gibson, J.M.: Significant ethnic variation in total and free testosterone concentration. Clin Endocrinol (Oxf) **58**(3), 262-266 (2003). doi:10.1046/j.1365-2265.2003.01653.x

482. Henkel, R., Maass, G., Schuppe, H.C., Jung, A., Schubert, J., Schill, W.B.: Molecular aspects of declining sperm motility in older men. Fertil Steril **84**(5), 1430-1437 (2005). doi:10.1016/j.fertnstert.2005.05.020

483. Herbert, P., Hayes, L.D., Sculthorpe, N.F., Grace, F.M.: HIIT produces increases in muscle power and free testosterone in male masters athletes. Endocr Connect **6**(7), 430-436 (2017). doi:10.3389/fphys.2017.00483

10.1530/ec-17-0159

484. Herbst, K.L., Coviello, A.D., Page, S., Amory, J.K., Anawalt, B.D., Bremner, W.J.: A single dose of the potent gonadotropin-releasing hormone antagonist acyline suppresses gonadotropins and testosterone for 2 weeks in healthy young men. J Clin Endocrinol Metab **89**(12), 5959-5965 (2004). doi:10.1210/jc.2003-032123

485. Hiam, D., Landen, S., Jacques, M., Voisin, S., Alvarez-Romero, J., Byrnes, E., Chubb, P., Levinger, I., Eynon, N.: Osteocalcin and its forms respond similarly to exercise in males and females. Bone **144**, 115818 (2021). doi:10.1002/ijc.33416

10.1016/j.bone.2020.115818

486. Hiéronimus, S., Lussiez, V., Le Duff, F., Ferrari, P., Bständig, B., Fénichel, P.: Klinefelter's syndrome and bone mineral density: is osteoporosis a constant feature? Ann Endocrinol (Paris) **72**(1), 14-18 (2011). doi:10.1016/j.ando.2010.10.002

487. Hildreth, K.L., Barry, D.W., Moreau, K.L., Vande Griend, J., Meacham, R.B., Nakamura, T., Wolfe, P., Kohrt, W.M., Ruscin, J.M., Kittelson, J., Cress, M.E., Ballard, R., Schwartz, R.S.: Effects of testosterone and progressive resistance exercise in healthy, highly functioning older men with low-normal testosterone levels. J Clin Endocrinol Metab **98**(5), 1891-1900 (2013). doi:10.1210/jc.2013-2227

488. Hill, M., Zárubová, J., Marusič, P., Vrbíková, J., Velíková, M., Kancheva, R., Kancheva, L., Kubátová, J., Dušková, M., Zamrazilová, L., Kazihnitková, H., Simůnková, K., Stárka, L.: Effects of valproate and carbamazepine monotherapy on neuroactive steroids, their precursors and metabolites in adult men with epilepsy. J Steroid Biochem Mol Biol **122**(4), 239-252 (2010). doi:10.1016/j.jsbmb.2010.06.003

489. Hirsh, A.V., Tyler, J.P., Landon, G., Pugh, R.C., Cameron, K.M., Pryor, J.P., Collins, W.P.: Testicular testosterone concentration, interstitial cell density and spermatogenesis in infertile men. Int J Androl **4**(4), 409-420 (1981). doi:10.1111/j.1365-2605.1981.tb00725.x

490. Hislop, M.S., Ratanjee, B.D., Soule, S.G., Marais, A.D.: Effects of anabolic-androgenic steroid use or gonadal testosterone suppression on serum leptin concentration in men. Eur J Endocrinol **141**(1), 40-46 (1999). doi:10.1530/eje.0.1410040

491. Hoefnagels, W.H., Drayer, J.I., Smals, A.G., Benraad, T.J., Kloppenborg, P.W.: Nocturnal, daytime, and postural changes of plasma aldosterone before and during dexamethasone in adenomatous and idiopathic aldosteronism. J Clin Endocrinol Metab **51**(6), 1330-1334 (1980). doi:10.1210/jcem-51-6-1330

492. Hogervorst, E., Bandelow, S., Combrinck, M., Smith, A.D.: Low free testosterone is an independent risk factor for Alzheimer's disease. Exp Gerontol **39**(11-12), 1633-1639 (2004). doi:10.1016/j.exger.2004.06.019

493. Holma, P., Adlercreutz, H.: Effect of an anabolic steroid (metandienon) on plasma LH-FSH, and testosterone and on the response to intravenous administration of LRH. Acta Endocrinol (Copenh) **83**(4), 856-864 (1976). doi:10.1530/acta.0.0830856

494. Holmboe, S.A., Priskorn, L., Jensen, T.K., Skakkebaek, N.E., Andersson, A.M., Jørgensen, N.: Use of e-cigarettes associated with lower sperm counts in a cross-sectional study of young men from the general population. Hum Reprod **35**(7), 1693-1701 (2020). doi:10.1093/humrep/deaa089

495. Ho-Pham, L.T., Nguyen, N.D., Nguyen, T.V.: Quantification of the relative contribution of estrogen to bone mineral density in men and women. BMC Musculoskelet Disord **14**, 366 (2013). doi:10.1186/1471-2474-14-366

496. Hori, T., Murawaki, Y., Hirayama, C.: Hypogonadism in liver cirrhosis: implication in altered amino acid metabolism in muscle. Biochem Med Metab Biol **39**(2), 168-175 (1988). doi:10.1016/0885-4505(88)90073-4

497. Horowitz, M., Wishart, J.M., O'Loughlin, P.D., Morris, H.A., Need, A.G., Nordin, B.E.: Osteoporosis and Klinefelter's syndrome. Clin Endocrinol (Oxf) **36**(1), 113-118 (1992). doi:10.1111/j.1365-2265.1992.tb02910.x

498. Horst, H.J., Bartsch, W., Dirksen-Thiedens, I.: Plasma testosterone, sex hormone binding globulin binding capacity and per cent binding of testosterone and 5alpha-dihydrotestosterone in prepubertal, pubertal and adult males. J Clin Endocrinol Metab **45**(3), 522-527 (1977). doi:10.1210/jcem-45-3-522

499. Hosseini, S.Y., Amini, E., Safarinejad, M.R., Soleimani, M., Lashay, A., Farokhpey, A.H.: Influence of opioid consumption on serum prostate-specific antigen levels in men without clinical evidence of prostate cancer. Urology **80**(1), 169-173 (2012). doi:10.1016/j.urology.2012.04.006

500. Hromadová, M., Hácik, T., Riecanský, I.: Concentration of lipid, apoprotein-B and testosterone in patients with coronarographic findings. Klin Wochenschr **63**(20), 1071-1074 (1985). doi:10.1007/bf01739675

501. HromadovÃ¡, M., HÃ¡cik, T., RiecanskÃ½, I.: Concentration of lipid, apoprotein-B and testosterone in patients with coronarographic findings. Klin Wochenschr **63**(20), 1071-1074 (1985). doi:10.1007/bf01739675

502. Hsu, B., Cumming, R.G., Blyth, F.M., Naganathan, V., Le Couteur, D.G., Seibel, M.J., Waite, L.M., Handelsman, D.J.: The longitudinal relationship of sexual function and androgen status in older men: the Concord Health and Ageing in Men Project. The Journal of clinical endocrinology and metabolism **100**(4), 1350-1358 (2015). doi:10.1210/jc.2014-4104

503. Huang, F., Li, Y., Cui, Y., Zhu, Z., Chen, J., Zeng, F., Li, Y., Chen, Z., Chen, H.: Relationship Between Serum Testosterone Levels and Kidney Stones Prevalence in Men. Frontiers in endocrinology **13**, 863675 (2022). doi:10.3389/fendo.2022.863675

504. Huang, Y., Dai, W., Li, Y.: Potential associations of testosterone/estradiol ratio, leukocyte hTERT expression and PBMC telomerase activity with aging and the presence of coronary artery disease in men. Exp Gerontol **117**, 38-44 (2019). doi:10.1016/j.exger.2018.08.008

505. Huang, Y.P., Liu, W., Chen, S.F., Liu, Y.D., Chen, B., Deng, C.H., Lu, M.J.: Free testosterone correlated with erectile dysfunction severity among young men with normal total testosterone. Int J Impot Res **31**(2), 132-138 (2019). doi:10.1038/s41443-018-0090-y

506. Huang, Y.P., Liu, W., Liu, Y.D., Wang, H.X., Hu, K., Chen, B., Lu, M.J., Li, W., Diao, X., Chen, C., Li, C., Zhang, Y., Li, Y.: Right testicular volume is a dominant predictor of testicular function determined by sperm parameters and total testosterone

Changes in hormones of the hypothalamic-pituitary-gonadal axis in migraine patients. Andrologia **50**, 165-171 (2018). doi:10.1111/and.12955

10.1016/j.jocn.2017.11.011

507. Hughes, A., Kumari, M.: Testosterone, risk, and socioeconomic position in British men: Exploring causal directionality. Soc Sci Med **220**, 129-140 (2019). doi:10.1016/j.socscimed.2018.11.004

508. Hughes, G.S., Ringer, T.V., Watts, K.C., DeLoof, M.J., Francom, S.F., Spillers, C.R.: Fish oil produces an atherogenic lipid profile in hypertensive men. Atherosclerosis **84**(2-3), 229-237 (1990). doi:10.1016/0021-9150(90)90095-z

509. Huhtaniemi, I.T., Tajar, A., Lee, D.M., O'Neill, T.W., Finn, J.D., Bartfai, G., Boonen, S., Casanueva, F.F., Giwercman, A., Han, T.S., Kula, K., Labrie, F., Lean, M.E., Pendleton, N., Punab, M., Silman, A.J., Vanderschueren, D., Forti, G., Wu, F.C.: Comparison of serum testosterone and estradiol measurements in 3174 European men using platform immunoassay and mass spectrometry; relevance for the diagnostics in aging men. Eur J Endocrinol **166**(6), 983-991 (2012). doi:10.1530/eje-11-1051

510. Hung, Y.J., Lee, C.H., Shieh, Y.S., Hsiao, F.C., Lin, F.H., Hsieh, C.H.: Gender differences in plasma growth arrest-specific protein 6 levels in adult subjects. Clin Chim Acta **441**, 1-5 (2015). doi:10.1016/j.cca.2014.12.001

511. Huuskonen, J., VÃ¤isÃ¤nen, S.B., KrÃ¶ger, H., Jurvelin, J.S., PenttilÃ¤, I., Alhava, E., Rauramaa, R.: Relation of sex hormones to bone mineral density in middle-aged men during a 4 year exercise intervention trial. Bone **31**(1), 51-56 (2002). doi:10.1016/s8756-3282(02)00795-0

512. Huuskonen, J., Väisänen, S.B., Kröger, H., Jurvelin, J.S., Penttilä, I., Alhava, E., Rauramaa, R.: Relation of sex hormones to bone mineral density in middle-aged men during a 4 year exercise intervention trial. Bone **31**(1), 51-56 (2002). doi:10.1016/s8756-3282(02)00795-0

513. I, I.J., Renken, R.J., Gietema, J.A., Slart, R.H., Mensink, M.G., Lefrandt, J.D., Ter Horst, G.J., Reyners, A.K.: Taste and smell function in testicular cancer survivors treated with cisplatin-based chemotherapy in relation to dietary intake, food preference, and body composition. Appetite **105**, 392-399 (2016). doi:10.1016/j.appet.2016.06.010

514. Idan, A., Griffiths, K.A., Harwood, D.T., Seibel, M.J., Turner, L., Conway, A.J., Handelsman, D.J.: Long-term effects of dihydrotestosterone treatment on prostate growth in healthy, middle-aged men without prostate disease: a randomized, placebo-controlled trial. Ann Intern Med **153**(10), 621-632 (2010). doi:10.7326/0003-4819-153-10-201011160-00004

515. Ilani, N., Roth, M.Y., Amory, J.K., Swerdloff, R.S., Dart, C., Page, S.T., Bremner, W.J., Sitruk-Ware, R., Kumar, N., Blithe, D.L., Wang, C.: A new combination of testosterone and nestorone transdermal gels for male hormonal contraception. J Clin Endocrinol Metab **97**(10), 3476-3486 (2012). doi:10.1210/jc.2012-1384

516. Iranmanesh, A., Lawson, D., Veldhuis, J.D.: Glucose ingestion acutely lowers pulsatile LH and basal testosterone secretion in men. Am J Physiol Endocrinol Metab **302**(6), E724-730 (2012). doi:10.1152/ajpendo.00520.2011

517. Iranmanesh, A., Veldhuis, J.D., Samojlik, E., Rogol, A.D., Johnson, M.L., Lizarralde, G.: Alterations in the pulsatile properties of gonadotropin secretion in alcoholic men. Journal of andrology **9**(3), 207-214 (1988). doi:10.1002/j.1939-4640.1988.tb01036.x

518. Irie, F., Strozyk, D., Peila, R., Korf, E.S., Remaley, A.T., Masaki, K., White, L.R., Launer, L.J.: Brain lesions on MRI and endogenous sex hormones in elderly men. Neurobiol Aging **27**(8), 1137-1144 (2006). doi:10.1016/j.neurobiolaging.2005.05.015

519. Ishigaki, T., Koyama, K., Tsujita, J., Tanaka, N., Hori, S., Oku, Y.: Plasma leptin levels of elite endurance runners after heavy endurance training. J Physiol Anthropol Appl Human Sci **24**(6), 573-578 (2005). doi:10.2114/jpa.24.573

520. Ishikawa, T., Yamaguchi, K., Chiba, K., Takenaka, A., Fujisawa, M.: Serum hormones in patients with nonobstructive azoospermia after microdissection testicular sperm extraction. J Urol **182**(4), 1495-1499 (2009). doi:10.1016/j.juro.2009.06.029

521. Ishimaru, T.: Plasma estradiol concentrations and effect of HCG on plasma estradiol and testosterone in normal subjects and patients with endocrine disorders. Endocrinol Jpn **22**(4), 287-296 (1975). doi:10.1507/endocrj1954.22.287

522. Ishimaru, T., Pages, L., Horton, R.: Altered metabolism of androgens in elderly men with benign prostatic hyperplasia. J Clin Endocrinol Metab **45**(4), 695-701 (1977). doi:10.1210/jcem-45-4-695

523. Isidori, A.M., Strollo, F., MorÃ¨, M., Caprio, M., Aversa, A., Moretti, C., Frajese, G., Riondino, G., Fabbri, A.: Leptin and aging: correlation with endocrine changes in male and female healthy adult populations of different body weights. J Clin Endocrinol Metab **85**(5), 1954-1962 (2000). doi:10.1210/jcem.85.5.6572

524. Isidori, A.M., Strollo, F., Morè, M., Caprio, M., Aversa, A., Moretti, C., Frajese, G., Riondino, G., Fabbri, A.: Leptin and aging: correlation with endocrine changes in male and female healthy adult populations of different body weights. J Clin Endocrinol Metab **85**(5), 1954-1962 (2000). doi:10.1210/jcem.85.5.6572

525. Isojärvi, J.I., Pakarinen, A.J., Myllylä, V.V.: Effects of carbamazepine therapy on serum sex hormone levels in male patients with epilepsy. Epilepsia **29**(6), 781-786 (1988). doi:10.1111/j.1528-1157.1988.tb04235.x

526. Isurugi, K., Kanazawa, M., Yanaihara, T., Kambegawa, A.: Responses of serum levels of testicular steroid hormones to hCG stimulation in patients with prostatic cancer and benign prostatic hypertrophy. Prostate Suppl **1**, 19-26 (1981). doi:10.1002/pros.2990020505

527. Ito, T., Horton, R.: The source of plasma dihydrotestosterone in man. J Clin Invest **50**(8), 1621-1627 (1971). doi:10.1172/jci106650

528. Izquierdo, M., HÃ¤kkinen, K., IbaÃ±ez, J., Garrues, M., AntÃ³n, A., ZÃºÃ±iga, A., LarriÃ³n, J.L., Gorostiaga, E.M.: Effects of strength training on muscle power and serum hormones in middle-aged and older men. J Appl Physiol (1985) **90**(4), 1497-1507 (2001). doi:10.1152/jappl.2001.90.4.1497

529. Izquierdo, M., Hakkinen, K., Ibanez, J., Garrues, M., Anton, A., Zuniga, A., Larrion, J.L., Gorostiaga, E.M.: Effects of strength training on muscle power and serum hormones in middle-aged and older men. Journal of applied physiology (Bethesda, Md. : 1985) **90**(4), 1497-1507 (2001). doi:10.1152/jappl.2001.90.4.1497

530. Jackaman, R., Ghanadian, R., Ansell, I.D., McLoughlin, P.V., Chisholm, G.D.: Relationships between spermatogenesis and serum hormone levels in subfertile men. Br J Obstet Gynaecol **84**(9), 692-696 (1977). doi:10.1111/j.1471-0528.1977.tb12682.x

531. Jackson, J.A., Riggs, M.W., Spiekerman, A.M.: Testosterone deficiency as a risk factor for hip fractures in men: a case-control study. Am J Med Sci **304**(1), 4-8 (1992). doi:10.1097/00000441-199207000-00003

532. Jahan, S., Falah, S., Ullah, H., Ullah, A., Rauf, N.: Antioxidant enzymes status and reproductive health of adult male workers exposed to brick kiln pollutants in Pakistan. Environ Sci Pollut Res Int **23**(13), 12932-12940 (2016). doi:10.1007/s11356-016-6454-2

533. Jakobsson, J., EkstrÃ¶m, L., Inotsume, N., Garle, M., Lorentzon, M., Ohlsson, C., Roh, H.K., CarlstrÃ¶m, K., Rane, A.: Large differences in testosterone excretion in Korean and Swedish men are strongly associated with a UDP-glucuronosyl transferase 2B17 polymorphism. J Clin Endocrinol Metab **91**(2), 687-693 (2006). doi:10.1210/jc.2005-1643

534. Jakobsson, J., Ekström, L., Inotsume, N., Garle, M., Lorentzon, M., Ohlsson, C., Roh, H.K., Carlström, K., Rane, A.: Large differences in testosterone excretion in Korean and Swedish men are strongly associated with a UDP-glucuronosyl transferase 2B17 polymorphism. J Clin Endocrinol Metab **91**(2), 687-693 (2006). doi:10.1210/jc.2005-1643

535. Jan, W., Gold, N.I.: Serum testosterone analysis by competitive protein binding: chromatography on silica-impregnated fiberglass and protein precipitation with ammonium sulfate. Biochem Med **6**(1), 7-18 (1972). doi:10.1016/0006-2944(72)90054-3

536. Jänne, O., Apter, D., Vihko, R.: Assay of testosterone, progesterone and 17alpha-hydroxyprogesterone in human plasma by radioimmunoassay after separation on hydroxyalkoxypropyl sephadex. J Steroid Biochem **5**(2), 155-162 (1974). doi:10.1016/0022-4731(74)90122-8

537. Jannini, E.A., Screponi, E., Carosa, E., Pepe, M., Lo Giudice, F., Trimarchi, F., Benvenga, S.: Lack of sexual activity from erectile dysfunction is associated with a reversible reduction in serum testosterone. International journal of andrology **22**(6), 385-392 (1999).

538. Jaspers, L., Dhana, K., Muka, T., Meun, C., Kiefte-de Jong, J.C., Hofman, A., Laven, J.S., Franco, O.H., Kavousi, M.: Sex Steroids, Sex Hormone-Binding Globulin and Cardiovascular Health in Men and Postmenopausal Women: The Rotterdam Study. J Clin Endocrinol Metab **101**(7), 2844-2852 (2016). doi:10.1210/jc.2016-1435

539. Jaworski, P.E., Ramos, A., Nicoleit, A.R., Bacarin, L.F., Olivo, P.N.: Importance of abdominal circumference and body mass index values in predicting male hypogonadism - A practical approach. Arch Endocrinol Metab **61**(1), 76-80 (2017). doi:10.1590/2359-3997000000203

540. Jeibmann, A., Zahedi, S., Simoni, M., Nieschlag, E., Byrne, M.M.: Glucagon-like peptide-1 reduces the pulsatile component of testosterone secretion in healthy males. Eur J Clin Invest **35**(9), 565-572 (2005). doi:10.1111/j.1365-2362.2005.01542.x

541. Jensen, J., Oftebro, H., Breigan, B., Johnsson, A., Ohlin, K., Meen, H.D., Strømme, S.B., Dahl, H.A.: Comparison of changes in testosterone concentrations after strength and endurance exercise in well trained men. Eur J Appl Physiol Occup Physiol **63**(6), 467-471 (1991). doi:10.1007/bf00868080

542. Jensen, T.K., Finne, K.F., Skakkebæk, N.E., Andersson, A.M., Olesen, I.A., Joensen, U.N., Bang, A.K., Nordkap, L., Priskorn, L., Krause, M., Jørgensen, N., Juul, A.: Self-reported onset of puberty and subsequent semen quality and reproductive hormones in healthy young men. Hum Reprod **31**(8), 1886-1894 (2016). doi:10.1093/humrep/dew122

543. Jeppesen, L.L., JÃ¸rgensen, H.S., Nakayama, H., Raaschou, H.O., Olsen, T.S., Winther, K.: Decreased serum testosterone in men with acute ischemic stroke. Arterioscler Thromb Vasc Biol **16**(6), 749-754 (1996). doi:10.1161/01.atv.16.6.749

544. Jeppesen, L.L., Jørgensen, H.S., Nakayama, H., Raaschou, H.O., Olsen, T.S., Winther, K.: Decreased serum testosterone in men with acute ischemic stroke. Arterioscler Thromb Vasc Biol **16**(6), 749-754 (1996). doi:10.1161/01.atv.16.6.749

545. Ji, B., Jin, X.B.: Varicocele is associated with hypogonadism and impaired erectile function: a prospective comparative study. Andrologia **49**(6) (2017). doi:10.1111/and.12683

546. Jin, B., Beilin, J., Zajac, J., Handelsman, D.J.: Androgen receptor gene polymorphism and prostate zonal volumes in Australian and Chinese men. J Androl **21**(1), 91-98 (2000).

547. Jin, B., Turner, L., Walters, W.A., Handelsman, D.J.: The effects of chronic high dose androgen or estrogen treatment on the human prostate [corrected]. J Clin Endocrinol Metab **81**(12), 4290-4295 (1996). doi:10.1210/jcem.81.12.8954029

548. Joensen, U.N., Jørgensen, N., Thyssen, J.P., Szecsi, P.B., Stender, S., Petersen, J.H., Andersson, A.M., Frederiksen, H.: Urinary excretion of phenols, parabens and benzophenones in young men: Associations to reproductive hormones and semen quality are modified by mutations in the Filaggrin gene. Environ Int **121**(Pt 1), 365-374 (2018). doi:10.1016/j.envint.2018.09.020

549. Johansson, A.G., Forslund, A., Hambraeus, L., Blum, W.F., Ljunghall, S.: Growth hormone-dependent insulin-like growth factor binding protein is a major determinant of bone mineral density in healthy men. J Bone Miner Res **9**(6), 915-921 (1994). doi:10.1002/jbmr.5650090617

550. Johnsonbaugh, R.E., Georges, L.P., Czerwinski, C.L., Edson, M.: Plasma testosterone, luteinizing hormone and follicle-stimulating hormone one day after vasectomy. Andrologia **11**(4), 294-296 (1979). doi:10.1111/j.1439-0272.1979.tb02208.x

551. Johnsonbaugh, R.E., O'Connell, K., Engel, S.B., Edson, M., Sode, J.: Plasma testosterone, luteinizing hormone, and follicle-stimulating hormone after vasectomy. Fertil Steril **26**(4), 329-330 (1975).

552. Jones, T.M., Fang, V.S., Landau, R.L., Rosenfield, R.: Direct inhibition of Leydig cell function by estradiol. J Clin Endocrinol Metab **47**(6), 1368-1373 (1978). doi:10.1210/jcem-47-6-1368

553. Jones, T.W., Howatson, G., Russell, M., French, D.N.: Effects of strength and endurance exercise order on endocrine responses to concurrent training. Eur J Sport Sci **17**(3), 326-334 (2017). doi:10.1080/17461391.2016.1236148

554. Judge, L.W., Bellar, D.M., Hoover, D.L., Biggs, D., Leitzelar, B.N., Craig, B.W.: Effects of acute androstenedione supplementation on testosterone levels in older men. Aging Male **19**(3), 161-167 (2016). doi:10.3109/13685538.2016.1167180

555. JurasoviÄ‡, J., CvitkoviÄ‡, P., Pizent, A., Colak, B., Telisman, S.: Semen quality and reproductive endocrine function with regard to blood cadmium in Croatian male subjects. Biometals **17**(6), 735-743 (2004). doi:10.1007/s10534-004-1689-7

556. Kabakci, G., Yildirir, A., Can, I., Unsal, I., Erbas, B.: Relationship between endogenous sex hormone levels, lipoproteins and coronary atherosclerosis in men undergoing coronary angiography. Cardiology **92**(4), 221-225 (1999). doi:10.1159/000006977

557. Kalman, D., Feldman, S., Martinez, M., Krieger, D.R., Tallon, M.J.: Effect of protein source and resistance training on body composition and sex hormones. J Int Soc Sports Nutr **4**, 4 (2007). doi:10.1186/1550-2783-4-4

558. Kamijima, M., Hibi, H., Gotoh, M., Taki, K., Saito, I., Wang, H., Itohara, S., Yamada, T., Ichihara, G., Shibata, E., Nakajima, T., Takeuchi, Y.: A survey of semen indices in insecticide sprayers. J Occup Health **46**(2), 109-118 (2004). doi:10.1539/joh.46.109

559. Kampmann, J.P., Jorgensen, F.S., Bennett, E.P., Johnsen, S.G.: Rise in dehydroepiandrosterone and oestrogens during clomiphene administration in normal men. Acta Endocrinol (Copenh) **83**(1), 166-172 (1976). doi:10.1530/acta.0.0830166

560. Kannan, V., Vijaya, G.: Endocrine testicular functions in leprosy. Horm Metab Res **16**(3), 146-150 (1984). doi:10.1055/s-2007-1014723

561. Karabakan, M., Erkmen, A.E., Guzel, O., Aktas, B.K., Bozkurt, A., Akdemir, S.: Association between serum folic acid level and erectile dysfunction. Andrologia **48**(5), 532-535 (2016). doi:10.1111/and.12474

562. Karakas, M., SchÃ¤fer, S., Appelbaum, S., Ojeda, F., Kuulasmaa, K., BrÃ¼ckmann, B., Berisha, F., Schulte-Steinberg, B., Jousilahti, P., Blankenberg, S., Palosaari, T., Salomaa, V., Zeller, T.: Testosterone Levels and Type 2 Diabetes-No Correlation with Age, Differential Predictive Value in Men and Women. Biomolecules **8**(3) (2018). doi:10.3390/biom8030076

563. Karakas, M., Schäfer, S., Appelbaum, S., Ojeda, F., Kuulasmaa, K., Brückmann, B., Berisha, F., Schulte-Steinberg, B., Jousilahti, P., Blankenberg, S., Palosaari, T., Salomaa, V., Zeller, T.: Testosterone Levels and Type 2 Diabetes-No Correlation with Age, Differential Predictive Value in Men and Women. Biomolecules **8**(3) (2018). doi:10.3390/biom8030076

564. Karavitakis, M., Komninos, C., Simaioforidis, V., Kontos, S., Lefakis, G., Politis, V., Koritsiadis, G., Konstantellou, K., Doumanis, G.: The relationship between androgens, regulators of collagen metabolism, and Peyronie's disease: a case control study. J Sex Med **7**(12), 4011-4017 (2010). doi:10.1111/j.1743-6109.2010.01915.x

565. Karlović, D., Serretti, A., Marčinko, D., Martinac, M., Silić, A., Katinić, K.: Serum testosterone concentration in combat-related chronic posttraumatic stress disorder. Neuropsychobiology **65**(2), 90-95 (2012). doi:10.1159/000329556

566. Karr, J.P., Wajsman, Z., Kirdani, R.Y., Murphy, G.P., Sandberg, A.A.: Effects of diethylstilbestrol and estramustine phosphate on serum sex hormone binding globulin and testosterone levels in prostate cancer patients. J Urol **124**(2), 232-236 (1980). doi:10.1016/s0022-5347(17)55383-5

567. KeleÅŸ, I., Aydin, G., BaÅŸar, M.M., Hayran, M., Atalar, E., Orkun, S., Batislam, E.: Endogenous sex steroids and bone mineral density in healthy men. Joint Bone Spine **73**(1), 80-85 (2006). doi:10.1016/j.jbspin.2005.04.003

568. Keleş, I., Aydin, G., Başar, M.M., Hayran, M., Atalar, E., Orkun, S., Batislam, E.: Endogenous sex steroids and bone mineral density in healthy men. Joint Bone Spine **73**(1), 80-85 (2006). doi:10.1016/j.jbspin.2005.04.003

569. Keleşoğlu, M., Kızılay, F., Barutçuoğlu, B., Başol, G., Saraç, F., Mutaf, I., Semerci, B., Zaid, M.A., Gathirua-Mwangi, W.G., Fung, C., Monahan, P.O., El-Charif, O., Williams, A.M., Feldman, D.R., Hamilton, R.J., Vaughn, D.J., Beard, C.J., Cook, R., Althouse, S.K., Ardeshir-Rouhani-Fard, S., Dinh, P.C., Sesso, H.D., Einhorn, L.H., Fossa, S.D., Travis, L.B.: The relationship between lipoprotein-associated phospholipase A2 with cardiovascular risk factors in testosterone deficiency

Clinical and Genetic Risk Factors for Adverse Metabolic Outcomes in North American Testicular Cancer Survivors. Turk J Urol **44**(2), 103-108 (2018). doi:10.5152/tud.2017.30633

10.6004/jnccn.2017.7046

570. Kendler, M., Makrantonaki, E., Tzellos, T., Kratzsch, J., Anderegg, U., Wetzig, T., Zouboulis, C., Simon, J.C.: Elevated sex steroid hormones in great saphenous veins in men. J Vasc Surg **51**(3), 639-646 (2010). doi:10.1016/j.jvs.2009.07.128

571. Kenny, A.M., Prestwood, K.M., Gruman, C.A., Marcello, K.M., Raisz, L.G.: Effects of transdermal testosterone on bone and muscle in older men with low bioavailable testosterone levels. J Gerontol A Biol Sci Med Sci **56**(5), M266-272 (2001). doi:10.1093/gerona/56.5.m266

572. Keshri, N., Nandeesha, H., Kattimani, S.: Elevated interleukin-17 and reduced testosterone in bipolar disorder. Relation with suicidal behaviour. Asian J Psychiatr **36**, 66-68 (2018). doi:10.1016/j.ajp.2018.06.011

573. Keskin, M.Z., Budak, S., Zeyrek, T., Çelik, O., Mertoglu, O., Yoldas, M., Ilbey, Y.: The relationship between serum hormone levels (follicle-stimulating hormone, luteinizing hormone, total testosterone) and semen parameters. Arch Ital Urol Androl **87**(3), 194-197 (2015). doi:10.4081/aiua.2015.3.194

574. Key, T.J., Roe, L., Thorogood, M., Moore, J.W., Clark, G.M., Wang, D.Y.: Testosterone, sex hormone-binding globulin, calculated free testosterone, and oestradiol in male vegans and omnivores. Br J Nutr **64**(1), 111-119 (1990). doi:10.1079/bjn19900014

575. Khan, H.L., Bhatti, S., Abbas, S., Khan, Y.L., Gonzalez, R.M.M., Aslamkhan, M., Gonzalez, G.R., Aydin, H.H.: Serotonin transporter (5-HTTLPR) genotypes and trinucleotide repeats of androgen receptor exert a combinatorial effect on hormonal milieu in patients with lifelong premature ejaculation. Andrology **6**(6), 916-926 (2018). doi:10.1111/andr.12518

576. Khan, H.L., Bhatti, S., Abbas, S., Khan, Y.L., Gonzalez, R.M.M., Aslamkhan, M., Gonzalez, G.R., Aydin, H.H., Savkovic, S., Lim, S., Jayadev, V., Conway, A., Turner, L., Curtis, D., Goebel, C., Handelsman, D.J.: Longer trinucleotide repeats of androgen receptor are associated with higher testosterone and low oxytocin levels in diabetic premature ejaculatory dysfunction patients

Urine and Serum Sex Steroid Profile in Testosterone-Treated Transgender and Hypogonadal and Healthy Control Men. Basic Clin Androl **28**(6), 3 (2018). doi:10.1186/s12610-018-0068-0

10.1210/jc.2018-00054

577. Khaw, K.T., Barrett-Connor, E.: Lower endogenous androgens predict central adiposity in men. Annals of epidemiology **2**(5), 675-682 (1992). doi:10.1016/1047-2797(92)90012-f

578. Khosla, S., Melton, L.J., 3rd, Atkinson, E.J., O'Fallon, W.M.: Relationship of serum sex steroid levels to longitudinal changes in bone density in young versus elderly men. J Clin Endocrinol Metab **86**(8), 3555-3561 (2001). doi:10.1210/jcem.86.8.7736

579. Kicovic, P.M., Luisi, M., Franchi, F., Krempl, S.: Circulating prolactin and its response to TRH following administration of testosterone undecanoate in normal men. J Endocrinol Invest **1**(4), 359-361 (1978). doi:10.1007/bf03350983

580. Kidd, G.S., Glass, A.R., Vigersky, R.A.: The hypothalamic-pituitary-testicular axis in thyrotoxicosis. J Clin Endocrinol Metab **48**(5), 798-802 (1979). doi:10.1210/jcem-48-5-798

581. Kikuchi, T.A., Skowsky, W.R., El-Toraei, I., Swerdloff, R.: The pituitary-gonadal axis in spinal cord injury. Fertil Steril **27**(10), 1142-1145 (1976). doi:10.1016/s0015-0282(16)42130-8

582. Kilciler, G., Ozata, M., Oktenli, C., Sanisoglu, S.Y., Bolu, E., Bingol, N., Kilciler, M., Ozdemir, I.C., Kutlu, M.: Diurnal leptin secretion is intact in male hypogonadotropic hypogonadism and is not influenced by exogenous gonadotropins. J Clin Endocrinol Metab **87**(11), 5023-5029 (2002). doi:10.1210/jc.2002-020516

583. Kim, B.J., Rhee, P.L., Park, J.H., Chang, D.K., Kim, Y.H., Son, H.J., Kim, J.J., Rhee, J.C., Lee, H.: Male sex hormones may influence the symptoms of irritable bowel syndrome in young men. Digestion **78**(2-3), 88-92 (2008). doi:10.1159/000166600

584. Kim, E.A., Cheong, H.K., Joo, K.D., Shin, J.H., Lee, J.S., Choi, S.B., Kim, M.O., Lee Iu, J., Kang, D.M.: Effect of manganese exposure on the neuroendocrine system in welders. Neurotoxicology **28**(2), 263-269 (2007). doi:10.1016/j.neuro.2006.07.013

585. Kim, H., Kang, J.W., Ku, S.Y., Kim, S.H., Cho, S.H., Koong, S.S., Kim, Y.D., Lee, C.H.: Effect of 'PC Game Room' use and polycyclic aromatic hydrocarbon exposure on plasma testosterone concentrations in young male Koreans. Hum Reprod **20**(3), 598-603 (2005). doi:10.1093/humrep/deh645

586. Kim, K.S., Kang, S.H., Kim, M.J., Kim, S.K., Kim, Y.L., Park, W.K., Park, S.W., Cho, Y.W.: Low serum testosterone concentrations in hospitalized men with poorly controlled type 2 diabetes. Endocrinol Metab (Seoul) **29**(4), 574-578 (2014). doi:10.3803/EnM.2014.29.4.574

587. Kim, Y.S., Hong, D., Lee, D.J., Joo, N.S., Kim, K.M.: Total testosterone may not decline with ageing in Korean men aged 40 years or older. Clin Endocrinol (Oxf) **77**(2), 296-301 (2012). doi:10.1111/j.1365-2265.2012.04375.x

588. Kirschner, M.A., Knorr, D.W.: Suppression of androgen and oestrogen production in normal men. Acta Endocrinol (Copenh) **70**(2), 342-350 (1972). doi:10.1530/acta.0.0700342

589. Kische, H., Gross, S., Wallaschofski, H., Grabe, H.J., Völzke, H., Nauck, M., Haring, R.: Associations of androgens with depressive symptoms and cognitive status in the general population. PLoS One **12**(5), e0177272 (2017). doi:10.1371/journal.pone.0177272

590. Kische, H., Pieper, L., Venz, J., Klotsche, J., März, W., Koch-Gromus, U., Pittrow, D., Lehnert, H., Silber, S., Stalla, G.K., Zeiher, A.M., Wittchen, H.U., Haring, R.: Longitudinal change instead of baseline testosterone predicts depressive symptoms. Psychoneuroendocrinology **89**, 7-12 (2018). doi:10.1016/j.psyneuen.2017.12.013

591. Klaiber, E.L., Broverman, D.M.: Dynamics of estradiol and testosterone and seminal fluid indexes in smokers and nonsmokers. Fertil Steril **50**(4), 630-634 (1988). doi:10.1016/s0015-0282(16)60196-6

592. Klaus, J., Reinshagen, M., Adler, G., Boehm, B., von Tirpitz, C.: Bones and Crohn's: estradiol deficiency in men with Crohn's disease is not associated with reduced bone mineral density. BMC Gastroenterol **8**, 48 (2008). doi:10.1186/1471-230x-8-48

593. Kletter, G.B., Foster, C.M., Beitins, I.Z., Marshall, J.C., Kelch, R.P.: Acute effects of testosterone infusion and naloxone on luteinizing hormone secretion in normal men. J Clin Endocrinol Metab **75**(5), 1215-1219 (1992). doi:10.1210/jcem.75.5.1430081

594. Kley, H.K., Deselaers, T., Peerenboom, H.: Evidence for hypogonadism in massively obese males due to decreased free testosterone. Horm Metab Res **13**(11), 639-641 (1981). doi:10.1055/s-2007-1019359

595. Kley, H.K., Edelmann, P., Krüskemper, H.L.: Relationship of plasma sex hormones to different parameters of obesity in male subjects. Metabolism **29**(11), 1041-1045 (1980). doi:10.1016/0026-0495(80)90214-0

596. Kley, H.K., Müller, A., Peerenboom, H., Krüskemper, H.L.: Digoxin does not alter plasma steroid levels in health men. Clin Pharmacol Ther **32**(1), 12-17 (1982). doi:10.1038/clpt.1982.120

597. Kley, H.K., Nieschlag, E., Wiegelmann, W., Krüskemper, H.L.: Oestrone, oestradiol and testosterone in normal and hypogonadal men following LH-RH or HCG stimulation. Acta Endocrinol (Copenh) **81**(3), 616-622 (1976). doi:10.1530/acta.0.0810616

598. Kley, H.K., Solbach, H.G., McKinnan, J.C., Krüskemper, H.L.: Testosterone decrease and oestrogen increase in male patients with obesity. Acta Endocrinol (Copenh) **91**(3), 553-563 (1979). doi:10.1530/acta.0.0910553

599. Klibanski, A., Beitins, I.Z., Badger, T., Little, R., McArthur, J.W.: Reproductive function during fasting in men. J Clin Endocrinol Metab **53**(2), 258-263 (1981). doi:10.1210/jcem-53-2-258

600. Knoblovits, P., Costanzo, P.R., Valzacchi, G.J., Gueglio, G., Layus, A.O., Kozak, A.E., Balzaretti, M.I., Litwak, L.E.: Erectile dysfunction, obesity, insulin resistance, and their relationship with testosterone levels in eugonadal patients in an andrology clinic setting. J Androl **31**(3), 263-270 (2010). doi:10.2164/jandrol.109.007757

601. Knuth, U.A., Kühne, J., Crosby, J., Bals-Pratsch, M., Kelly, R.W., Nieschlag, E.: Indomethacin and oxaprozin lower seminal prostaglandin levels but do not influence sperm motion characteristics and serum hormones of young healthy men in a placebo-controlled double-blind trial. J Androl **10**(2), 108-119 (1989). doi:10.1002/j.1939-4640.1989.tb00071.x

602. Kobori, Y., Koh, E., Sugimoto, K., Izumi, K., Narimoto, K., Maeda, Y., Konaka, H., Mizokami, A., Matsushita, T., Iwamoto, T., Namiki, M.: The relationship of serum and salivary cortisol levels to male sexual dysfunction as measured by the International Index of Erectile Function. Int J Impot Res **21**(4), 207-212 (2009). doi:10.1038/ijir.2009.14

603. Koh, K., Kim, S.S., Kim, J.S., Jung, J.G., Yoon, S.J., Suh, W.Y., Kim, H.G., Kim, N., Lee, J.H., Jung, H.D., Choi, J.D., Kang, J.Y., Yoo, T.K., Park, Y.W.: Relationship between Alcohol Consumption and Testosterone Deficiency according to Facial Flushes among Middle-Aged and Older Korean Men

Non-linear association between testosterone and LDL concentrations in men. Korean J Fam Med **43**(6), 381-387 (2022). doi:10.4082/kjfm.21.0173

10.1111/andr.13393

604. Kokot, F., Nieszporek, T.: Influence of hemoperfusion on the concentrations of calcitonin, testosterone and cortisol in blood plasma. Artif Organs **3**(4), 332-335 (1979). doi:10.1111/j.1525-1594.1979.tb01072.x

605. Kolodny, R.C., Masters, W.H., Hendryx, J., Toro, G.: Plasma testosterone and semen analysis in male homosexuals. N Engl J Med **285**(21), 1170-1174 (1971). doi:10.1056/nejm197111182852104

606. Kolodny, R.C., Masters, W.H., Kolodner, R.M., Toro, G.: Depression of plasma testosterone levels after chronic intensive marihuana use. N Engl J Med **290**(16), 872-874 (1974). doi:10.1056/nejm197404182901602

607. Kon, M., Ikeda, T., Homma, T., Suzuki, Y.: Effects of low-intensity resistance exercise under acute systemic hypoxia on hormonal responses. J Strength Cond Res **26**(3), 611-617 (2012). doi:10.1519/JSC.0b013e3182281c69

608. Kong, X.B., Guan, H.T., Li, H.G., Zhou, Y., Xiong, C.L.: The ageing males' symptoms scale for Chinese men: reliability,validation and applicability of the Chinese version. Andrology **2**(6), 856-861 (2014). doi:10.1111/j.2047-2927.2013.00145.x

609. Koşar, P.A., Ozçelik, N., Koşar, A.: Cytogenetic abnormalities detected in patients with non-obstructive azoospermia and severe oligozoospermia. J Assist Reprod Genet **27**(1), 17-21 (2010). doi:10.1007/s10815-009-9366-y

610. Kravos, A., Hočevar-Boltežar, I., Geršak, K.: Serum levels of sex hormones in males with Reinke's edema. Eur Arch Otorhinolaryngol **270**(1), 233-238 (2013). doi:10.1007/s00405-012-2156-7

611. Kreuz, L.E., Rose, R.M.: Assessment of aggressive behavior and plasma testosterone in a young criminal population. Psychosom Med **34**(4), 321-332 (1972). doi:10.1097/00006842-197207000-00006

612. Kruljac, M., Finnbogadóttir, H., Bobjer, J., Giraldi, A., Fugl-Meyer, K., Giwercman, A.: Symptoms of sexual dysfunction among men from infertile couples: prevalence and association with testosterone deficiency. Andrology **8**(1), 160-165 (2020). doi:10.4183/aeb.2016.418

10.1111/andr.12678

613. Książek, A., Mędraś, M., Zagrodna, A., Słowińska-Lisowska, M., Lwow, F.: Correlative studies on vitamin D and total, free bioavailable testosterone levels in young, healthy men. Sci Rep **11**(1), 20198 (2021). doi:10.1016/s1474-4422(21)00252-0

10.1038/s41598-021-99571-8

614. Kuchakulla, M., Nackeeran, S., Blachman-Braun, R., Ramasamy, R.: The association between plant-based content in diet and testosterone levels in US adults. World J Urol **39**(4), 1307-1311 (2021). doi:10.1007/s00345-020-03276-y

615. Kuchuk, N.O., van Schoor, N.M., Pluijm, S.M., Smit, J.H., de Ronde, W., Lips, P.: The association of sex hormone levels with quantitative ultrasound, bone mineral density, bone turnover and osteoporotic fractures in older men and women. Clin Endocrinol (Oxf) **67**(2), 295-303 (2007). doi:10.1111/j.1365-2265.2007.02882.x

616. Kucukdurmaz, F., Acar, G., Resim, S., Hart, R.J., Doherty, D.A., Keelan, J.A., Minaee, N.S., Thorstensen, E.B., Dickinson, J.E., Pennell, C.E., Newnham, J.P., McLachlan, R., Norman, R.J., Handelsman, D.J.: Deterioration of Chronotropic Responses and Heart Rate Recovery Indices in Men With Erectile Dysfunction

The impact of antenatal Bisphenol A exposure on male reproductive function at 20-22 years of age. Sex Med **6**(1), 8-14 (2018). doi:10.1016/j.esxm.2017.10.002

10.1016/j.rbmo.2017.11.009

617. Kukkonen-Harjula, K., Oja, P., Laustiola, K., Vuori, I., Jolkkonen, J., Siitonen, S., Vapaatalo, H.: Haemodynamic and hormonal responses to heat exposure in a Finnish sauna bath. Eur J Appl Physiol Occup Physiol **58**(5), 543-550 (1989). doi:10.1007/bf02330710

618. Kumanov, P., Nandipati, K., Tomova, A., Agarwal, A.: Inhibin B is a better marker of spermatogenesis than other hormones in the evaluation of male factor infertility. Fertil Steril **86**(2), 332-338 (2006). doi:10.1016/j.fertnstert.2006.01.022

619. Kupelian, V., Chiu, G.R., Araujo, A.B., Williams, R.E., Clark, R.V., McKinlay, J.B.: Association of sex hormones and C-reactive protein levels in men. Clin Endocrinol (Oxf) **72**(4), 527-533 (2010). doi:10.1111/j.1365-2265.2009.03713.x

620. Kurniawan, A.L., Hsu, C.Y., Chao, J.C., Paramastri, R., Lee, H.A., Lai, P.C., Hsieh, N.C., Wu, S.V.: Association of Testosterone-Related Dietary Pattern with Testicular Function among Adult Men: A Cross-Sectional Health Screening Study in Taiwan. Nutrients **13**(1) (2021). doi:10.3390/nu13010259

621. Kvorning, T., Andersen, M., Brixen, K., Schjerling, P., Suetta, C., Madsen, K.: Suppression of testosterone does not blunt mRNA expression of myoD, myogenin, IGF, myostatin or androgen receptor post strength training in humans. J Physiol **578**(Pt 2), 579-593 (2007). doi:10.1113/jphysiol.2006.122671

622. Laaksi, A., Laaksi, I., Pihlajamäki, H., Vaara, J.P., Luukkaala, T., Kyröläinen, H., Zueger, R., Annen, H., Ehlert, U.: Associations of serum 25(OH)D levels with physical performance and anabolic hormones in young men

Testosterone and cortisol responses to acute and prolonged stress during officer training school. Front Physiol **14**(1), 1049503 (2023). doi:10.3389/fphys.2023.1049503

10.1080/10253890.2023.2199886

623. Laaksonen, D.E., Niskanen, L., Punnonen, K., NyyssÃ¶nen, K., Tuomainen, T.P., Valkonen, V.P., Salonen, R., Salonen, J.T.: Testosterone and sex hormone-binding globulin predict the metabolic syndrome and diabetes in middle-aged men. Diabetes Care **27**(5), 1036-1041 (2004). doi:10.2337/diacare.27.5.1036

624. Laaksonen, D.E., Niskanen, L., Punnonen, K., Nyyssönen, K., Tuomainen, T.P., Valkonen, V.P., Salonen, R., Salonen, J.T.: Testosterone and sex hormone-binding globulin predict the metabolic syndrome and diabetes in middle-aged men. Diabetes Care **27**(5), 1036-1041 (2004). doi:10.2337/diacare.27.5.1036

625. Labropoulos, B., Velonakis, E., Oekonomakos, P., Laskaris, J., Katsimades, D.: Serum sex hormones in patients with coronary disease and their relationship to known factors causing atherosclerosis. Cardiology **69**(2), 98-103 (1982). doi:10.1159/000173490

626. Lagiou, P., Signorello, L.B., Mantzoros, C.S., Trichopoulos, D., Hsieh, C.C., Trichopoulou, A.: Hormonal, lifestyle, and dietary factors in relation to leptin among elderly men. Ann Nutr Metab **43**(1), 23-29 (1999). doi:10.1159/000012763

627. Lagiou, P., Signorello, L.B., Trichopoulos, D., Tzonou, A., Trichopoulou, A., Mantzoros, C.S.: Leptin in relation to prostate cancer and benign prostatic hyperplasia. Int J Cancer **76**(1), 25-28 (1998). doi:10.1002/(sici)1097-0215(19980330)76:1<25::aid-ijc5>3.0.co;2-#

628. Lakshman, K.M., Bhasin, S., Araujo, A.B.: Sex hormone-binding globulin as an independent predictor of incident type 2 diabetes mellitus in men. J Gerontol A Biol Sci Med Sci **65**(5), 503-509 (2010). doi:10.1093/gerona/glq002

629. Lakshman, K.M., Kaplan, B., Travison, T.G., Basaria, S., Knapp, P.E., Singh, A.B., LaValley, M.P., Mazer, N.A., Bhasin, S.: The effects of injected testosterone dose and age on the conversion of testosterone to estradiol and dihydrotestosterone in young and older men. J Clin Endocrinol Metab **95**(8), 3955-3964 (2010). doi:10.1210/jc.2010-0102

630. Lane, A.R., Hackney, A.C.: Relationship between salivary and serum testosterone levels in response to different exercise intensities. Hormones (Athens) **14**(2), 258-264 (2015). doi:10.14310/horm.2002.1561

631. Lapauw, B., Ouwens, M., t Hart, L.M., Wuyts, B., Holst, J.J., T'Sjoen, G., Kaufman, J.M., Ruige, J.B.: Sex steroids affect triglyceride handling, glucose-dependent insulinotropic polypeptide, and insulin sensitivity: a 1-week randomized clinical trial in healthy young men. Diabetes Care **33**(8), 1831-1833 (2010). doi:10.2337/dc10-0515

632. Lapauw, B., Taes, Y., Goemaere, S., Toye, K., Zmierczak, H.G., Kaufman, J.M.: Anthropometric and skeletal phenotype in men with idiopathic osteoporosis and their sons is consistent with deficient estrogen action during maturation. J Clin Endocrinol Metab **94**(11), 4300-4308 (2009). doi:10.1210/jc.2009-0568

633. Lapauw, B., Taes, Y., Simoens, S., Van Caenegem, E., Weyers, S., Goemaere, S., Toye, K., Kaufman, J.M., T'Sjoen, G.G.: Body composition, volumetric and areal bone parameters in male-to-female transsexual persons. Bone **43**(6), 1016-1021 (2008). doi:10.1016/j.bone.2008.09.001

634. Lapauw, B.M., Taes, Y., Bogaert, V., Vanbillemont, G., Goemaere, S., Zmierczak, H.G., De Bacquer, D., Kaufman, J.M.: Serum estradiol is associated with volumetric BMD and modulates the impact of physical activity on bone size at the age of peak bone mass: a study in healthy male siblings. J Bone Miner Res **24**(6), 1075-1085 (2009). doi:10.1359/jbmr.081260

635. Lašaitė, L., Ceponis, J., Preikša, R.T., Zilaitienė, B.: Impaired emotional state, quality of life and cognitive functions in young hypogonadal men. Andrologia **46**(10), 1107-1112 (2014). doi:10.1111/and.12199

636. Laughlin, G.A., Barrett-Connor, E., Bergstrom, J.: Low serum testosterone and mortality in older men. J Clin Endocrinol Metab **93**(1), 68-75 (2008). doi:10.1210/jc.2007-1792

637. Laughlin, G.A., Barrett-Connor, E., May, S.: Sex-specific association of the androgen to oestrogen ratio with adipocytokine levels in older adults: the Rancho Bernardo Study. Clin Endocrinol (Oxf) **65**(4), 506-513 (2006). doi:10.1111/j.1365-2265.2006.02624.x

638. Lawrence, D., Shaw, M., Katz, M.: Elevated free testosterone concentration in men and women with acne vulgaris. Clin Exp Dermatol **11**(3), 263-273 (1986). doi:10.1111/j.1365-2230.1986.tb00458.x

639. Lawrence, D.M., Swyer, G.I.: Plasma testosterone and testosterone binding affinities in men with impotence, oligospermia, azoospermia, and hypogonadism. Br Med J **1**(5904), 349-351 (1974). doi:10.1136/bmj.1.5904.349

640. Leal, A.M., Magalhães, P.K., Souza, C.S., Foss, N.T.: Pituitary-gonadal hormones and interleukin patterns in leprosy. Trop Med Int Health **11**(9), 1416-1421 (2006). doi:10.1111/j.1365-3156.2006.01692.x

641. Leal, D.V., Taylor, L., Hough, J.: Reproducibility of Acute Steroid Hormone Responses in Men to Short-Duration Running. Int J Sports Physiol Perform **14**(10), 1430-1437 (2019). doi:10.1123/ijspp.2018-1004

642. LeBlanc, E.S., Nielson, C.M., Marshall, L.M., Lapidus, J.A., Barrett-Connor, E., Ensrud, K.E., Hoffman, A.R., Laughlin, G., Ohlsson, C., Orwoll, E.S.: The effects of serum testosterone, estradiol, and sex hormone binding globulin levels on fracture risk in older men. The Journal of clinical endocrinology and metabolism **94**(9), 3337-3346 (2009). doi:10.1210/jc.2009-0206

643. LeBlanc, E.S., Wang, P.Y., Janowsky, J.S., Neiss, M.B., Fink, H.A., Yaffe, K., Marshall, L.M., Lapidus, J.A., Stefanick, M.L., Orwoll, E.S.: Association between sex steroids and cognition in elderly men. Clin Endocrinol (Oxf) **72**(3), 393-403 (2010). doi:10.1111/j.1365-2265.2009.03692.x

644. Leder, B.Z., Longcope, C., Catlin, D.H., Ahrens, B., Schoenfeld, D.A., Finkelstein, J.S.: Oral androstenedione administration and serum testosterone concentrations in young men. Jama **283**(6), 779-782 (2000). doi:10.1001/jama.283.6.779

645. Lee, A., Rubinow, K., Clark, R.V., Caricofe, R.B., Bush, M.A., Zhi, H., Roth, M.Y., Page, S.T., Bremner, W.J., Amory, J.K.: Pharmacokinetics of modified slow-release oral testosterone over 9 days in normal men with experimental hypogonadism. J Androl **33**(3), 420-426 (2012). doi:10.2164/jandrol.111.014514

646. Lee, D.M., Pye, S.R., Tajar, A., O'Neill, T.W., Finn, J.D., Boonen, S., Bartfai, G., Casanueva, F.F., Forti, G., Giwercman, A., Han, T.S., Huhtaniemi, I.T., Kula, K., Lean, M.E., Pendleton, N., Punab, M., Silman, A.J., Vanderschueren, D., Wu, F.C.: Cohort profile: the European Male Ageing Study. Int J Epidemiol **42**(2), 391-401 (2013). doi:10.1093/ije/dyr234

647. Lee, J.H., Lee, S.W.: Impact of prostate volume on erectile dysfunction and premature ejaculation. Aging Male **19**(2), 106-110 (2016). doi:10.3109/13685538.2016.1150993

648. Lee, J.H., Lee, S.W.: Monthly Variations in Serum Testosterone Levels: Results from Testosterone Screening of 8,367 Middle-Aged Men. J Urol **205**(5), 1438-1443 (2021). doi:10.1097/ju.0000000000001546

649. Lee, M.H., Seo, D.H., Lee, C.W., Choi, J.H., Jeh, S.U., Lee, S.W., Choi, S.M., Hwa, J.S., Hyun, J.S., Chung, K.H., Kam, S.C., Liu, Q., Zhao, Y., Gu, Y., Shang, X., Zhou, Y., Zhang, H., Zuo, L., Mei, G., Li, H., Xiong, C., Zafar, M.I.: Relationship between Hypogonadal Symptoms, Sexual Dysfunction and Chronic Prostatitis in Middle-Aged Men by Self-Reported Questionnaires, even without Biochemical Testosterone Deficiency

The association of age-related differences in serum total testosterone and sex hormone-binding globulin levels with the prevalence of diabetes. World J Mens Health **38**(2), 243-249 (2020). doi:10.5534/wjmh.190117

10.1016/j.archger.2020.104040

650. Lee, Y.C., Huang, S.P., Juan, Y.S., Huang, T.Y., Liu, C.C.: Impact of metabolic syndrome and its components on kidney stone in aging Taiwanese males. Aging Male **19**(3), 197-201 (2016). doi:10.1080/13685538.2016.1174987

651. Legrand, E., Hedde, C., Gallois, Y., Degasne, I., Boux de Casson, F., Mathieu, E., BaslÃ©, M.F., Chappard, D., Audran, M.: Osteoporosis in men: a potential role for the sex hormone binding globulin. Bone **29**(1), 90-95 (2001). doi:10.1016/s8756-3282(01)00478-1

652. Legrand, E., Hedde, C., Gallois, Y., Degasne, I., Boux de Casson, F., Mathieu, E., Baslé, M.F., Chappard, D., Audran, M.: Osteoporosis in men: a potential role for the sex hormone binding globulin. Bone **29**(1), 90-95 (2001). doi:10.1016/s8756-3282(01)00478-1

653. Lemcke, B., Zentgraf, J., Behre, H.M., Kliesch, S., Bramswig, J.H., Nieschlag, E.: Long-term effects on testicular function of high-dose testosterone treatment for excessively tall stature. J Clin Endocrinol Metab **81**(1), 296-301 (1996). doi:10.1210/jcem.81.1.8550767

654. Lennartsson, A.K., Kushnir, M.M., Bergquist, J., Billig, H., Jonsdottir, I.H.: Sex steroid levels temporarily increase in response to acute psychosocial stress in healthy men and women. Int J Psychophysiol **84**(3), 246-253 (2012). doi:10.1016/j.ijpsycho.2012.03.001

655. Lenters, V., Portengen, L., Smit, L.A., JÃ¶nsson, B.A., Giwercman, A., Rylander, L., Lindh, C.H., SpanÃ², M., Pedersen, H.S., Ludwicki, J.K., Chumak, L., Piersma, A.H., Toft, G., Bonde, J.P., Heederik, D., Vermeulen, R.: Phthalates, perfluoroalkyl acids, metals and organochlorines and reproductive function: a multipollutant assessment in Greenlandic, Polish and Ukrainian men. Occup Environ Med **72**(6), 385-393 (2015). doi:10.1136/oemed-2014-102264

656. Lenters, V., Portengen, L., Smit, L.A., Jönsson, B.A., Giwercman, A., Rylander, L., Lindh, C.H., Spanò, M., Pedersen, H.S., Ludwicki, J.K., Chumak, L., Piersma, A.H., Toft, G., Bonde, J.P., Heederik, D., Vermeulen, R.: Phthalates, perfluoroalkyl acids, metals and organochlorines and reproductive function: a multipollutant assessment in Greenlandic, Polish and Ukrainian men. Occup Environ Med **72**(6), 385-393 (2015). doi:10.1136/oemed-2014-102264

657. Leppäluoto, J., Korhonen, I., Huttunen, P., Hassi, J.: Serum levels of thyroid and adrenal hormones, testosterone, TSH, LH, GH and prolactin in men after a 2-h stay in a cold room. Acta Physiol Scand **132**(4), 543-548 (1988). doi:10.1111/j.1748-1716.1988.tb08363.x

658. Lerchbaum, E., Pilz, S., Trummer, C., Schwetz, V., Pachernegg, O., Heijboer, A.C., Obermayer-Pietsch, B.: Vitamin D and Testosterone in Healthy Men: A Randomized Controlled Trial. J Clin Endocrinol Metab **102**(11), 4292-4302 (2017). doi:10.1210/jc.2017-01428

659. Lessov-Schlaggar, C.N., Reed, T., Swan, G.E., Krasnow, R.E., DeCarli, C., Marcus, R., Holloway, L., Wolf, P.A., Carmelli, D.: Association of sex steroid hormones with brain morphology and cognition in healthy elderly men. Neurology **65**(10), 1591-1596 (2005). doi:10.1212/01.wnl.0000184512.08249.48

660. Lévi, F.A., Canon, C., Touitou, Y., Sulon, J., Mechkouri, M., Ponsart, E.D., Touboul, J.P., Vannetzel, J.M., Mowzowicz, I., Reinberg, A., et al.: Circadian rhythms in circulating T lymphocyte subtypes and plasma testosterone, total and free cortisol in five healthy men. Clin Exp Immunol **71**(2), 329-335 (1988).

661. Levitt, A.J., Joffe, R.T.: Total and free testosterone in depressed men. Acta Psychiatr Scand **77**(3), 346-348 (1988). doi:10.1111/j.1600-0447.1988.tb05132.x

662. Li, J., Xiao, W., Sha, W., Xian, K., Tang, X., Zhang, X.: Relationship of serum testosterone levels with cognitive function in chronic antipsychotic-treated male patients with schizophrenia. Asia Pac Psychiatry **7**(3), 323-329 (2015). doi:10.1111/appy.12168

663. Li, J.J., Wittert, G.A., Vincent, A., Atlantis, E., Shi, Z., Appleton, S.L., Hill, C.L., Jenkins, A.J., Januszewski, A.S., Adams, R.J.: Muscle grip strength predicts incident type 2 diabetes: Population-based cohort study. Metabolism **65**(6), 883-892 (2016). doi:10.1016/j.metabol.2016.03.011

664. Li, J.W., Gu, Y.Q.: Predictors for partial suppression of spermatogenesis of hormonal male contraception. Asian J Androl **10**(5), 723-730 (2008). doi:10.1111/j.1745-7262.2008.00432.x

665. Li, Z., Tang, T., Wu, W., Gu, L., Du, J., Zhao, T., Zhou, X., Wu, H., Qin, G.: Efficacy of nasal continuous positive airway pressure on patients with OSA with erectile dysfunction and low sex hormone levels. Respir Med **119**, 130-134 (2016). doi:10.1016/j.rmed.2016.09.001

666. Liao, C.H., Chiang, H.S., Yu, H.J.: Serum testosterone levels significantly correlate with nocturia in men aged 40-79 years. Urology **78**(3), 631-635 (2011). doi:10.1016/j.urology.2011.05.033

667. Liao, C.H., Huang, C.Y., Li, H.Y., Yu, H.J., Chiang, H.S., Liu, C.K.: Testosterone and sex hormone-binding globulin have significant association with metabolic syndrome in Taiwanese men. Aging Male **15**(1), 1-6 (2012). doi:10.3109/13685538.2011.597462

668. Liao, C.H., Li, H.Y., Chung, S.D., Chiang, H.S., Yu, H.J.: Significant association between serum dihydrotestosterone level and prostate volume among Taiwanese men aged 40-79 years. Aging Male **15**(1), 28-33 (2012). doi:10.3109/13685538.2010.550660

669. Liao, C.H., Li, H.Y., Yu, H.J., Chiang, H.S., Lin, M.S., Hua, C.H., Ma, W.Y.: Low serum sex hormone-binding globulin: marker of inflammation? Clin Chim Acta **413**(7-8), 803-807 (2012). doi:10.1016/j.cca.2012.01.021

670. Liao, M., Huang, X., Gao, Y., Tan, A., Lu, Z., Wu, C., Zhang, Y., Yang, X., Zhang, H., Qin, X., Mo, Z.: Testosterone is associated with erectile dysfunction: a cross-sectional study in Chinese men. PLoS One **7**(6), e39234 (2012). doi:10.1371/journal.pone.0039234

671. Liao, P.W., Wu, C.C., Chen, K.C., Jaw, F.S., Yu, H.J., Liu, S.P., Ho, C.H.: Testosterone Threshold for Increased Cardiovascular Risk in Middle-Aged and Elderly Men: A Locally Weighted Regression Analysis. J Sex Med **13**(12), 1872-1880 (2016). doi:10.1016/j.jsxm.2016.10.002

672. Lillo, P., Zitko, P., Godoy-Reyes, G., Asenjo, G., Sáez, D., Cea, G., Navarrete, P., Valenzuela, D., Hughes, R., Heverin, M., Logroscino, G., Hardiman, O.: Incidence of amyotrophic lateral sclerosis in Chile. Amyotroph Lateral Scler Frontotemporal Degener **25**(5-6), 528-532 (2024). doi:10.1080/21678421.2024.2329706

673. Lin, J.W., Lee, J.K., Wu, C.K., Caffrey, J.L., Chang, M.H., Hwang, J.J., Dowling, N., Lin, Y.S.: Metabolic syndrome, testosterone, and cardiovascular mortality in men. J Sex Med **8**(8), 2350-2360 (2011). doi:10.1111/j.1743-6109.2011.02343.x

674. Linna, M.S., Ahotupa, M., Irjala, K., Pöllänen, P., Huhtaniemi, I., Mäkinen, J., Perheentupa, A., Vasankari, T.J.: Smoking and low serum testosterone associates with high concentration of oxidized LDL. Ann Med **40**(8), 634-640 (2008). doi:10.1080/07853890802161007

675. Linnoila, M., Prinz, P.N., Wonsowicz, C.J., Leppaluoto, J.: Effect of moderate doses of ethanol and phenobarbital on pituitary and thyroid hormones and testosterone. Br J Addict **75**(2), 207-212 (1980). doi:10.1111/j.1360-0443.1980.tb02446.x

676. Lipshultz, L.I., Greenberg, S.H., Caminos-Torres, R., Snyder, P.J.: Supranormal FSH response to gonadotrophin-releasing hormone in oligospermic men with a normal basal serum FSH concentration. Clin Endocrinol (Oxf) **7**(2), 103-109 (1977). doi:10.1111/j.1365-2265.1977.tb01301.x

677. Liu, C.C., Lee, Y.C., Huang, S.P., Cheng, K.H., Hsieh, T.J., Huang, T.Y., Lee, C.H., Geng, J.H., Li, C.C., Wu, W.J.: Hepatocyte Nuclear Factor-4α P2 Promoter Variants Are Associated With the Risk of Metabolic Syndrome and Testosterone Deficiency in Aging Taiwanese Men. J Sex Med **15**(11), 1527-1536 (2018). doi:10.1016/j.jsxm.2018.09.012

678. Liu, C.C., Lee, Y.C., Wang, C.J., Yeh, H.C., Li, W.M., Wu, W.J., Huang, C.N., Bao, B.Y., Huang, C.H., Huang, S.P.: The impact of androgen receptor CAG repeat polymorphism on andropausal symptoms in different serum testosterone levels. J Sex Med **9**(9), 2429-2437 (2012). doi:10.1111/j.1743-6109.2012.02672.x

679. Liu, C.C., Wu, W.J., Lee, Y.C., Wang, C.J., Ke, H.L., Li, W.M., Hsiao, H.L., Yeh, H.C., Li, C.C., Chou, Y.H., Huang, C.H., Huang, S.P.: The prevalence of and risk factors for androgen deficiency in aging Taiwanese men. J Sex Med **6**(4), 936-946 (2009). doi:10.1111/j.1743-6109.2008.01171.x

680. Liu, N., Luo, X., Li, P., Xiong, W.: The Triglycerides and Glucose Index is not superior to HOMA-IR in predicting testosterone deficiency among adult males. Andrology **11**(2), 215-224 (2023). doi:10.1111/andr.13207

681. Liu, Z., Liu, J., Shi, X., Wang, L., Yang, Y., Tao, M., Fu, Q.: Comparing calculated free testosterone with total testosterone for screening and diagnosing late-onset hypogonadism in aged males: A cross-sectional study. J Clin Lab Anal **31**(5) (2017). doi:10.1002/jcla.22073

682. Lokaj-Berisha, V., Gacaferri-Lumezi, B., Berisha, N., Gashi-Hoxha, S.: A Pilot Study on BMI, Serum Testosterone and Estradiol Levels in Allergic Male Patients. Open Access Maced J Med Sci **3**(4), 595-600 (2015). doi:10.3889/oamjms.2015.101

683. Lokeshwar, S.D., Patel, P., Fantus, R.J., Halpern, J., Chang, C., Kargi, A.Y., Ramasamy, R.: Decline in Serum Testosterone Levels Among Adolescent and Young Adult Men in the USA. Eur Urol Focus **7**(4), 886-889 (2021). doi:10.1016/j.euf.2020.02.006

684. Longcope, C., Widrich, W., Sawin, C.T.: The secretion of estrone and estradiol-17 by human testis. Steroids **20**(4), 439-448 (1972). doi:10.1016/0039-128x(72)90042-6

685. Lookingbill, D.P., Egan, N., Santen, R.J., Demers, L.M.: Correlation of serum 3 alpha-androstanediol glucuronide with acne and chest hair density in men. J Clin Endocrinol Metab **67**(5), 986-991 (1988). doi:10.1210/jcem-67-5-986

686. Łopuszańska-Dawid, M., Szklarska, A., Kołodziej, H., Lipowicz, A., Jankowska, E.A.: The relationship between: occupational status, biological condition and androgen hormone level among Polish adult men: the Wroclaw Male Study. Aging Male **19**(4), 231-238 (2016). doi:10.1080/13685538.2016.1220519

687. Lormeau, C., Soudan, B., d'Herbomez, M., Pigny, P., Duquesnoy, B., Cortet, B.: Sex hormone-binding globulin, estradiol, and bone turnover markers in male osteoporosis. Bone **34**(6), 933-939 (2004). doi:10.1016/j.bone.2004.01.024

688. Lotti, F., Corona, G., Maseroli, E., Rossi, M., Silverii, A., Degl'innocenti, S., Rastrelli, G., Forti, G., Maggi, M.: Clinical implications of measuring prolactin levels in males of infertile couples. Andrology **1**(5), 764-771 (2013). doi:10.1111/j.2047-2927.2013.00114.x

689. Lu, Y., Kang, J., Li, Z., Wang, X., Liu, K., Zhou, K., Wang, W., Shen, C., Barbosa, L.P., da Silva Aguiar, S., Santos, P.A., Dos Santos Rosa, T., Maciel, L.A., de Deus, L.A., Neves, R.V.P., de Araújo Leite, P.L., Gutierrez, S.D., Sousa, C.V., Korhonen, M.T., Degens, H., Simões, H.G.: The association between plant-based diet and erectile dysfunction in Chinese men

Relationship between inflammatory biomarkers and testosterone levels in male master athletes and non-athletes. Basic Clin Androl **31**(1), 11 (2021). doi:10.1186/s12610-021-00129-5

10.1016/j.exger.2021.111407

690. Luboshitzky, R., Aviv, A., Hefetz, A., Herer, P., Shen-Orr, Z., Lavie, L., Lavie, P.: Decreased pituitary-gonadal secretion in men with obstructive sleep apnea. J Clin Endocrinol Metab **87**(7), 3394-3398 (2002). doi:10.1210/jcem.87.7.8663

691. Luboshitzky, R., Levi, M., Shen-Orr, Z., Blumenfeld, Z., Herer, P., Lavie, P.: Long-term melatonin administration does not alter pituitary-gonadal hormone secretion in normal men. **15**(1), 60-65 (2000).

692. Luboshitzky, R., Shen-Orr, Z., Herer, P.: Seminal plasma melatonin and gonadal steroids concentrations in normal men. Arch Androl **48**(3), 225-232 (2002). doi:10.1080/01485010252869324

693. Luboshitzky, R., Shen-Orr, Z., Herer, P.: Middle-aged men secrete less testosterone at night than young healthy men. J Clin Endocrinol Metab **88**(7), 3160-3166 (2003). doi:10.1210/jc.2002-021920

694. Luboshitzky, R., Wagner, O., Lavi, S., Herer, P., Lavie, P.: Decreased nocturnal melatonin secretion in patients with Klinefelter's syndrome. Clin Endocrinol (Oxf) **45**(6), 749-754 (1996). doi:10.1046/j.1365-2265.1996.8710881.x

695. Luderer, U., Bushley, A., Stover, B.D., Bremner, W.J., Faustman, E.M., Takaro, T.K., Checkoway, H., Brodkin, C.A.: Effects of occupational solvent exposure on reproductive hormone concentrations and fecundability in men. Am J Ind Med **46**(6), 614-626 (2004). doi:10.1002/ajim.20100

696. Luderer, U., Morgan, M.S., Brodkin, C.A., Kalman, D.A., Faustman, E.M.: Reproductive endocrine effects of acute exposure to toluene in men and women. Occup Environ Med **56**(10), 657-666 (1999). doi:10.1136/oem.56.10.657

697. Luo, K., Zhang, R., Aimuzi, R., Wang, Y., Nian, M., Zhang, J.: Exposure to Organophosphate esters and metabolic syndrome in adults. Environ Int **143**, 105941 (2020). doi:10.1016/j.envint.2020.105941

698. Luppa, P.B., Thaler, M., Schulte-Frohlinde, E., Schreiegg, A., Huber, U., Metzger, J.: Unchanged androgen-binding properties of sex hormone-binding globulin in male patients with liver cirrhosis. Clin Chem Lab Med **44**(8), 967-973 (2006). doi:10.1515/cclm.2006.186

699. Ly, L.P., Handelsman, D.J.: Empirical estimation of free testosterone from testosterone and sex hormone-binding globulin immunoassays. Eur J Endocrinol **152**(3), 471-478 (2005). doi:10.1530/eje.1.01844

700. MÃ¤rtner, E.M.C., Thimm, E., Guder, P., Schiergens, K.A., Rutsch, F., Roloff, S., Marquardt, I., Das, A.M., Freisinger, P., GrÃ¼nert, S.C., KrÃ¤mer, J., Baumgartner, M.R., Beblo, S., Haase, C., Dieckmann, A., Lindner, M., NÃ¤ke, A., Hoffmann, G.F., MÃ¼hlhausen, C., Walter, M., Garbade, S.F., Maier, E.M., KÃ¶lker, S., Boy, N.: The biochemical subtype is a predictor for cognitive function in glutaric aciduria type 1: a national prospective follow-up study. Sci Rep **11**(1), 19300 (2021). doi:10.1038/s41598-021-98809-9

701. MaÃ¯moun, L., Lumbroso, S., Manetta, J., Paris, F., Leroux, J.L., Sultan, C.: Testosterone is significantly reduced in endurance athletes without impact on bone mineral density. Horm Res **59**(6), 285-292 (2003). doi:10.1159/000070627

702. MacAdams, M.R., White, R.H., Chipps, B.E.: Reduction of serum testosterone levels during chronic glucocorticoid therapy. Ann Intern Med **104**(5), 648-651 (1986). doi:10.7326/0003-4819-104-5-648

703. MacGilchrist, A.J., Howden, C.W., Kenyon, C.J., Beastall, G.H., Reid, J.L.: The effects of omeprazole on endocrine function in man. Eur J Clin Pharmacol **32**(4), 423-425 (1987). doi:10.1007/bf00543980

704. Macphee, G.J., Larkin, J.G., Butler, E., Beastall, G.H., Brodie, M.J.: Circulating hormones and pituitary responsiveness in young epileptic men receiving long-term antiepileptic medication. Epilepsia **29**(4), 468-475 (1988). doi:10.1111/j.1528-1157.1988.tb03747.x

705. Madrigal, J.M., Sargis, R.M., Persky, V., Turyk, M.E.: Multiple organochlorine pesticide exposures and measures of sex steroid hormones in adult males: Cross-sectional findings from the 1999-2004 National Health and Nutrition Examination Survey. Int J Hyg Environ Health **231**, 113609 (2021). doi:10.1016/j.ijheh.2020.113609

706. Maes, M., Mommen, K., Hendrickx, D., Peeters, D., D'Hondt, P., Ranjan, R., De Meyer, F., ScharpÃ©, S.: Components of biological variation, including seasonality, in blood concentrations of TSH, TT3, FT4, PRL, cortisol and testosterone in healthy volunteers. Clin Endocrinol (Oxf) **46**(5), 587-598 (1997). doi:10.1046/j.1365-2265.1997.1881002.x

707. Maes, M., Mommen, K., Hendrickx, D., Peeters, D., D'Hondt, P., Ranjan, R., De Meyer, F., Scharpe, S.: Components of biological variation, including seasonality, in blood concentrations of TSH, TT3, FT4, PRL, cortisol and testosterone in healthy volunteers. Clinical endocrinology **46**(5), 587-598 (1997).

708. Mahmoud, A.M., Goemaere, S., De Bacquer, D., Comhaire, F.H., Kaufman, J.M.: Serum inhibin B levels in community-dwelling elderly men. Clin Endocrinol (Oxf) **53**(2), 141-147 (2000). doi:10.1046/j.1365-2265.2000.01063.x

709. Mahoudeau, J.A., Delassalle, A., Bricaire, H.: Secretion of dihydrotestosterone by human prostate in benign prostatic hypertrophy. Acta Endocrinol (Copenh) **77**(2), 401-407 (1974). doi:10.1530/acta.0.0770401

710. Mahoudeau, J.A., Valcke, J.C., Bricaire, H.: Dissociated responses of plasma testosterone and estradiol to human chorionic gonadotropin in adult men. J Clin Endocrinol Metab **41**(1), 13-20 (1975). doi:10.1210/jcem-41-1-13

711. Maïmoun, L., Lumbroso, S., Manetta, J., Paris, F., Leroux, J.L., Sultan, C.: Testosterone is significantly reduced in endurance athletes without impact on bone mineral density. Horm Res **59**(6), 285-292 (2003). doi:10.1159/000070627

712. Maiorino, M.I., Bellastella, G., Petrizzo, M., Della Volpe, E., Orlando, R., Giugliano, D., Esposito, K.: Circulating endothelial progenitor cells in type 1 diabetic patients with erectile dysfunction. Endocrine **49**(2), 415-421 (2015). doi:10.1007/s12020-014-0478-5

713. Majzoub, A., Elbardisi, H., Madani, S., Leisegang, K., Mahdi, M., Agarwal, A., Henkel, R., Khalafalla, K., ElSaid, S., Arafa, M.: Impact of body composition analysis on male sexual function: A metabolic age study. Front Endocrinol (Lausanne) **13**, 1050441 (2022). doi:10.3389/fendo.2022.1050441

714. Mäkinen, J.I., Perheentupa, A., Irjala, K., Pöllänen, P., Mäkinen, J., Huhtaniemi, I., Raitakari, O.T.: Endogenous testosterone and serum lipids in middle-aged men. Atherosclerosis **197**(2), 688-693 (2008). doi:10.1016/j.atherosclerosis.2007.05.009

715. Malan, N.T., Hamer, M., Schutte, A.E., Huisman, H.W., van Rooyen, J.M., Schutte, R., Mels, C.M., Steyn, H.S., Smith, W., Fourie, C.M., Glyn, M., Malan, L.: Low testosterone and hyperkinetic blood pressure responses in a cohort of South African men: the SABPA Study. Clin Exp Hypertens **35**(3), 228-235 (2013). doi:10.3109/10641963.2012.721839

716. Malan, N.T., Stalder, T., Schlaich, M.P., Lambert, G.W., Hamer, M., Schutte, A.E., Huisman, H.W., Schutte, R., Smith, W., Mels, C.M., van Rooyen, J.M., Malan, L.: Chronic distress and acute vascular stress responses associated with ambulatory blood pressure in low-testosterone African men: the SABPA Study. J Hum Hypertens **28**(6), 393-398 (2014). doi:10.1038/jhh.2013.124

717. Maneesh, M., Jayalakshmi, H., Singh, T.A., Chakrabarti, A.: Impaired hypothalamic-pituitary-gonadal axis function in men with diabetes mellitus. Indian J Clin Biochem **21**(1), 165-168 (2006). doi:10.1007/bf02913088

718. Manfo, F.P., Moundipa, P.F., Déchaud, H., Tchana, A.N., Nantia, E.A., Zabot, M.T., Pugeat, M.: Effect of agropesticides use on male reproductive function: a study on farmers in Djutitsa (Cameroon). Environ Toxicol **27**(7), 423-432 (2012). doi:10.1002/tox.20656

719. Mantzoros, C.S., Liolios, A.D., Tritos, N.A., Kaklamani, V.G., Doulgerakis, D.E., Griveas, I., Moses, A.C., Flier, J.S.: Circulating insulin concentrations, smoking, and alcohol intake are important independent predictors of leptin in young healthy men. Obes Res **6**(3), 179-186 (1998). doi:10.1002/j.1550-8528.1998.tb00335.x

720. Marberger, M., Wilson, T.H., Rittmaster, R.S.: Low serum testosterone levels are poor predictors of sexual dysfunction. BJU Int **108**(2), 256-262 (2011). doi:10.1111/j.1464-410X.2010.09766.x

721. Markianos, M., Moussas, G., Lykouras, L.L.: Normal testosterone plasma levels in non-abstinent alcoholics. Drug Alcohol Depend **20**(1), 81-85 (1987). doi:10.1016/0376-8716(87)90078-0

722. Markianos, M., Tripodianakis, J., Sarantidis, D., Hatzimanolis, J.: Plasma testosterone and dehydroepiandrosterone sulfate in male and female patients with dysthymic disorder. J Affect Disord **101**(1-3), 255-258 (2007). doi:10.1016/j.jad.2006.11.013

723. Marques-Vidal, P., Sie, P., Cambou, J.P., Chap, H., Perret, B.: Relationships of plasminogen activator inhibitor activity and lipoprotein(a) with insulin, testosterone, 17 beta-estradiol, and testosterone binding globulin in myocardial infarction patients and healthy controls. J Clin Endocrinol Metab **80**(6), 1794-1798 (1995). doi:10.1210/jcem.80.6.7775625

724. Marrama, P., Carani, C., Baraghini, G.F., Volpe, A., Zini, D., Celani, M.F., Montanini, V.: Circadian rhythm of testosterone and prolactin in the ageing. Maturitas **4**(2), 131-138 (1982). doi:10.1016/0378-5122(82)90039-1

725. Marrama, P., Montanini, V., Celani, M.F., Carani, C., Cioni, K., Bazzani, M., Cavani, D., Baraghini, G.F.: Decrease in luteinizing hormone biological activity/immunoreactivity ratio in elderly men. Maturitas **5**(4), 223-231 (1984). doi:10.1016/0378-5122(84)90015-x

726. Marriott, R.J., Murray, K., Flicker, L., Hankey, G.J., Matsumoto, A.M., Dwivedi, G., Antonio, L., Almeida, O.P., Bhasin, S., Dobs, A.S., Handelsman, D.J., Haring, R., O'Neill, T.W., Ohlsson, C., Orwoll, E.S., Vanderschueren, D., Wittert, G.A., Wu, F.C.W., Yeap, B.B.: Lower serum testosterone concentrations are associated with a higher incidence of dementia in men: The UK Biobank prospective cohort study. Alzheimers Dement **18**(10), 1907-1918 (2022). doi:10.1002/alz.12529

727. Marriott, R.J., Murray, K., Hankey, G.J., Manning, L., Dwivedi, G., Wu, F.C.W., Yeap, B.B.: Longitudinal changes in serum testosterone and sex hormone-binding globulin in men aged 40-69 years from the UK Biobank. Clin Endocrinol (Oxf) **96**(4), 589-598 (2022). doi:10.1111/cen.14648

728. MartÃ­nez DÃ­az-Guerra, G., Hawkins, F., Rapado, A., Ruiz DÃ­az, M.A., DÃ­az-Curiel, M.: Hormonal and anthropometric predictors of bone mass in healthy elderly men: major effect of sex hormone binding globulin, parathyroid hormone and body weight. Osteoporos Int **12**(3), 178-184 (2001). doi:10.1007/s001980170127

729. Martikainen, H., Vihko, R.: HCG-stimulation of testicular steroidogenesis during induced hyper- and hypoprolactinaemia in man. Clin Endocrinol (Oxf) **16**(3), 227-234 (1982). doi:10.1111/j.1365-2265.1982.tb00711.x

730. Martín, A., Bravo, M., Arrabal, M., Magán-Fernández, A., Mesa, F.: Chronic periodontitis is associated with erectile dysfunction. A case-control study in european population. J Clin Periodontol **45**(7), 791-798 (2018). doi:10.1111/jcpe.12909

731. Martin, D.M., Wittert, G., Burns, N.R., McPherson, J.: Endogenous testosterone levels, mental rotation performance, and constituent abilities in middle-to-older aged men. Horm Behav **53**(3), 431-441 (2008). doi:10.1016/j.yhbeh.2007.11.012

732. Martínez-Jabaloyas, J.M., Queipo-Zaragozá, A., Ferrandis-Cortes, C., Queipo-Zaragozá, J.A., Gil-Salom, M., Chuan-Nuez, P.: [Relationships between sex hormone levels in men over 50 years of age and body composition, bone quality, and quality of life]. Actas Urol Esp **35**(9), 515-522 (2011). doi:10.1016/j.acuro.2011.04.010

733. Märtner, E.M.C., Thimm, E., Guder, P., Schiergens, K.A., Rutsch, F., Roloff, S., Marquardt, I., Das, A.M., Freisinger, P., Grünert, S.C., Krämer, J., Baumgartner, M.R., Beblo, S., Haase, C., Dieckmann, A., Lindner, M., Näke, A., Hoffmann, G.F., Mühlhausen, C., Walter, M., Garbade, S.F., Maier, E.M., Kölker, S., Boy, N.: The biochemical subtype is a predictor for cognitive function in glutaric aciduria type 1: a national prospective follow-up study. Sci Rep **11**(1), 19300 (2021). doi:10.1038/s41598-021-98809-9

734. Mascaro, J.S., Hackett, P.D., Gouzoules, H., Lori, A., Rilling, J.K.: Behavioral and genetic correlates of the neural response to infant crying among human fathers. Soc Cogn Affect Neurosci **9**(11), 1704-1712 (2014). doi:10.1093/scan/nst166

735. Masdrakis, V.G., Papageorgiou, C., Markianos, M.: Associations of plasma testosterone with clinical manifestations in acute panic disorder. Psychoneuroendocrinology **101**, 216-222 (2019). doi:10.1016/j.psyneuen.2018.11.017

736. Mastrogiacomo, I., De Besi, L., Zucchetta, P., Serafini, E., Gasparotto, M.L., Marchini, P., Pisani, E., Dean, P., Chini, M.: Effect of hyperprolactinemia and age on the hypogonadism of uremic men on hemodialysis. Arch Androl **12**(2-3), 235-242 (1984). doi:10.3109/01485018409161182

737. Mastrogiacomo, I., De Besi, L., Zucchetta, P., Serafini, E., La Greca, G., Gasparotto, M.L., Lorenzi, S., Dean, P.: Male hypogonadism of uremic patients on hemodialysis. Arch Androl **20**(2), 171-175 (1988). doi:10.3109/01485018808987070

738. Matta, R.A., Farrage, H.M., Saedii, A.A., Abdelrahman, M.M.: Male subclinical hypogonadism and late-onset hypergonadotrophic hypogonadism: mechanisms, endothelial function, and interplay between reproductive hormones, undercarboxylated osteocalcin, and endothelial dysfunction. Aging Male **25**(1), 72-87 (2022). doi:10.1080/13685538.2022.2049744

739. Mauss, J., Börsch, G., Bormacher, K., Richter, E., Leyendecker, G., Nocke, W.: Effect of long-term testosterone oenanthate administration on male reproductive function: clinical evaluation, serum FSH, LH, testosterone, and seminal fluid analyses in normal men. Acta Endocrinol (Copenh) **78**(2), 373-384 (1975). doi:10.1530/acta.0.0780373

740. McCaulley, G.O., McBride, J.M., Cormie, P., Hudson, M.B., Nuzzo, J.L., Quindry, J.C., Travis Triplett, N.: Acute hormonal and neuromuscular responses to hypertrophy, strength and power type resistance exercise. Eur J Appl Physiol **105**(5), 695-704 (2009). doi:10.1007/s00421-008-0951-z

741. McGregor, A.J., Mason, H.J.: Chronic occupational lead exposure and testicular endocrine function. Hum Exp Toxicol **9**(6), 371-376 (1990). doi:10.1177/096032719000900602

742. McIntyre, R.S., Mancini, D., Eisfeld, B.S., Soczynska, J.K., Grupp, L., Konarski, J.Z., Kennedy, S.H.: Calculated bioavailable testosterone levels and depression in middle-aged men. Psychoneuroendocrinology **31**(9), 1029-1035 (2006). doi:10.1016/j.psyneuen.2006.06.005

743. McMullen, M.H., Hamilton-Reeves, J.M., Bonorden, M.J., Wangen, K.E., Phipps, W.R., Feirtag, J.M., Kurzer, M.S.: Consumption of Lactobacillus acidophilus and Bifidobacterium longum does not alter phytoestrogen metabolism and plasma hormones in men: a pilot study. J Altern Complement Med **12**(9), 887-894 (2006). doi:10.1089/acm.2006.12.887

744. Meikle, A.W., Smith, J.A., Stringham, J.D.: Estradiol and testosterone metabolism and production in men with prostatic cancer. J Steroid Biochem **33**(1), 19-24 (1989). doi:10.1016/0022-4731(89)90352-x

745. Meikle, A.W., Stanish, W.M.: Familial prostatic cancer risk and low testosterone. J Clin Endocrinol Metab **54**(6), 1104-1108 (1982). doi:10.1210/jcem-54-6-1104

746. Meikle, A.W., Stanish, W.M., Taylor, N., Edwards, C.Q., Bishop, C.T.: Familial effects on plasma sex-steroid content in man: testosterone, estradiol and Sex-hormone-binding globulin. Metabolism **31**(1), 6-9 (1982).

747. Mendelson, J.H., Mello, N.K., Teoh, S.K., Ellingboe, J., Cochin, J.: Cocaine effects on pulsatile secretion of anterior pituitary, gonadal, and adrenal hormones. J Clin Endocrinol Metab **69**(6), 1256-1260 (1989). doi:10.1210/jcem-69-6-1256

748. Mendiola, J., JÃ¸rgensen, N., Andersson, A.M., Calafat, A.M., Ye, X., Redmon, J.B., Drobnis, E.Z., Wang, C., Sparks, A., Thurston, S.W., Liu, F., Swan, S.H.: Are environmental levels of bisphenol a associated with reproductive function in fertile men? Environ Health Perspect **118**(9), 1286-1291 (2010). doi:10.1289/ehp.1002037

749. Mendiola, J., Jørgensen, N., Andersson, A.M., Calafat, A.M., Ye, X., Redmon, J.B., Drobnis, E.Z., Wang, C., Sparks, A., Thurston, S.W., Liu, F., Swan, S.H.: Are environmental levels of bisphenol a associated with reproductive function in fertile men? Environ Health Perspect **118**(9), 1286-1291 (2010). doi:10.1289/ehp.1002037

750. Meriggiola, M.C., Bremner, W.J., Costantino, A., Bertaccini, A., Morselli-Labate, A.M., Huebler, D., Kaufmann, G., Oettel, M., Flamigni, C.: Twenty-one day administration of dienogest reversibly suppresses gonadotropins and testosterone in normal men. J Clin Endocrinol Metab **87**(5), 2107-2113 (2002). doi:10.1210/jcem.87.5.8514

751. Meriggiola, M.C., Bremner, W.J., Paulsen, C.A., Valdiserri, A., Incorvaia, L., Motta, R., Pavani, A., Capelli, M., Flamigni, C.: A combined regimen of cyproterone acetate and testosterone enanthate as a potentially highly effective male contraceptive. J Clin Endocrinol Metab **81**(8), 3018-3023 (1996). doi:10.1210/jcem.81.8.8768868

752. Meriggiola, M.C., Costantino, A., Bremner, W.J., Morselli-Labate, A.M.: Higher testosterone dose impairs sperm suppression induced by a combined androgen-progestin regimen. J Androl **23**(5), 684-690 (2002).

753. Mermall, H., Sothern, R.B., Kanabrocki, E.L., Quadri, S.F., Bremner, F.W., Nemchausky, B.A., Scheving, L.E.: Temporal (circadian) and functional relationship between prostate-specific antigen and testosterone in healthy men. Urology **46**(1), 45-53 (1995). doi:10.1016/s0090-4295(99)80157-5

754. Meyer, T.E., Chu, L.W., Li, Q., Yu, K., Rosenberg, P.S., Menashe, I., Chokkalingam, A.P., Quraishi, S.M., Huang, W.Y., Weiss, J.M., Kaaks, R., Hayes, R.B., Chanock, S.J., Hsing, A.W.: The association between inflammation-related genes and serum androgen levels in men: the prostate, lung, colorectal, and ovarian study. Prostate **72**(1), 65-71 (2012). doi:10.1002/pros.21407

755. Meyer-Bahlburg, H.F., Boon, D.A., Sharma, M., Edwards, J.A.: Aggressiveness and testosterone measures in man. Psychosom Med **36**(3), 269-274 (1974). doi:10.1097/00006842-197405000-00010

756. Mićić, S., Illić, V., Iśvaneski, M.: Correlation of hormone and histologic parameters in infertile men with varicocele. Urol Int **38**(3), 187-190 (1983). doi:10.1159/000280888

757. Mikuma, N., Kumamoto, Y., Maruta, H., Nitta, T.: Role of the hypothalamic opioidergic system in the control of gonadotropin secretion in elderly men. Andrologia **26**(1), 39-45 (1994). doi:10.1111/j.1439-0272.1994.tb00752.x

758. Miller, G.J., Wheeler, M.J., Price, S.G., Beckles, G.L., Kirkwood, B.R., Carson, D.C.: Serum high density lipoprotein subclasses, testosterone and sex-hormone-binding globulin in Trinidadian men of African and Indian descent. Atherosclerosis **55**(3), 251-258 (1985). doi:10.1016/0021-9150(85)90104-2

759. Miller, N.E., Nordoy, A.: Effects of human plasma lipoproteins on platelet aggregation and platelet factor 3 activity in vitro. Atherosclerosis **28**(2), 181-186 (1977). doi:10.1016/0021-9150(77)90155-1

760. Mima, M., Huang, J.B., Andriole, G.L., Freedland, S.J., Ohlander, S.J., Moreira, D.M.: The impact of smoking on sexual function. BJU Int **130**(2), 186-192 (2022). doi:10.1111/bju.15711

761. Mistry, S.D., Woods, G.N., Sigurdsson, S., Ewing, S.K., Hue, T.F., Eiriksdottir, G., Xu, K., Hilton, J.F., Kado, D.M., Gudnason, V., Harris, T.B., Rosen, C.J., Lang, T.F., Li, X., Schwartz, A.V.: Sex hormones are negatively associated with vertebral bone marrow fat. Bone **108**, 20-24 (2018). doi:10.1016/j.bone.2017.12.009

762. Mitchell, L.E., Sprecher, D.L., Borecki, I.B., Rice, T., Laskarzewski, P.M., Rao, D.C.: Evidence for an association between dehydroepiandrosterone sulfate and nonfatal, premature myocardial infarction in males. Circulation **89**(1), 89-93 (1994). doi:10.1161/01.cir.89.1.89

763. Mkacher, W., Tabka, Z., Chaieb, F., Gueddes, M., Zaouali, M., Aouichaoui, C., Zbidi, A., Trabelsi, Y.: Effect of rehabilitation program on endocrinological parameters in patients with COPD and in healthy subjects. Copd **11**(6), 681-688 (2014). doi:10.3109/15412555.2014.898048

764. Mohseni, M.G., Hosseini, S.R., Alizadeh, F., Rangzan, N.: Serum testosterone and gonadotropins levels in patients with premature ejaculation: A comparison with normal men. Adv Biomed Res **3**, 6 (2014). doi:10.4103/2277-9175.124633

765. Mok, C.C., Lau, C.S.: Profile of sex hormones in male patients with systemic lupus erythematosus. Lupus **9**(4), 252-257 (2000). doi:10.1191/096120300680198926

766. Mokshagundam, S.L., Minocha, A.: Does concurrent acute ethanol ingestion during omeprazole therapy affect pituitary gonadal axis in male subjects? J Toxicol Clin Toxicol **35**(1), 55-61 (1997). doi:10.3109/15563659709001166

767. Moltz, L., Römmler, A., Post, K., Schwartz, U., Hammerstein, J.: Medium dose cyproterone acetate (CPA): effects on hormone secretion and on spermatogenesis in men. Contraception **21**(4), 393-413 (1980). doi:10.1016/s0010-7824(80)80017-5

768. Montagna, G., Balestra, S., D'Aurizio, F., Romanelli, F., Benagli, C., Tozzoli, R., Risch, L., Giovanella, L., Imperiali, M.: Establishing normal values of total testosterone in adult healthy men by the use of four immunometric methods and liquid chromatography-mass spectrometry. Clin Chem Lab Med **56**(11), 1936-1944 (2018). doi:10.1515/cclm-2017-1201

769. Montanini, V., Simoni, M., Chiossi, G., Baraghini, G.F., Velardo, A., Baraldi, E., Marrama, P.: Age-related changes in plasma dehydroepiandrosterone sulphate, cortisol, testosterone and free testosterone circadian rhythms in adult men. Horm Res **29**(1), 1-6 (1988). doi:10.1159/000180956

770. Morgentaler, A., Polzer, P., Althof, S., Bolyakov, A., Donatucci, C., Ni, X., Patel, A.B., Basaria, S.: Delayed Ejaculation and Associated Complaints: Relationship to Ejaculation Times and Serum Testosterone Levels. J Sex Med **14**(9), 1116-1124 (2017). doi:10.1530/ec-17-0159

10.1016/j.jsxm.2017.06.013

771. Morley, J.E., Distiller, L.A., Sagel, J., Kok, S.H., Kay, G., Carr, P., Katz, M.: Hormonal changes associated with testicular atrophy and gynaecomastia in patients with leprosy. Clin Endocrinol (Oxf) **6**(4), 299-303 (1977). doi:10.1111/j.1365-2265.1977.tb02015.x

772. Morley, J.E., Kaiser, F.E., Perry, H.M., 3rd, Patrick, P., Morley, P.M., Stauber, P.M., Vellas, B., Baumgartner, R.N., Garry, P.J.: Longitudinal changes in testosterone, luteinizing hormone, and follicle-stimulating hormone in healthy older men. Metabolism **46**(4), 410-413 (1997). doi:10.1016/s0026-0495(97)90057-3

773. Morote, J., Planas, J., Ramirez, C., Gómez, E., Raventós, C.X., Placer, J., Catalán, R., de Torres, I.M.: Evaluation of the serum testosterone to prostate-specific antigen ratio as a predictor of prostate cancer risk. BJU Int **105**(4), 481-484 (2010). doi:10.1111/j.1464-410X.2009.08761.x

774. Morrison, J.C., Schneider, J.M., Kraus, A.P., Kitabchi, A.E.: The prevalence of diabetes mellitus in sickle cell hemoglobinopathies. J Clin Endocrinol Metab **48**(2), 192-195 (1979). doi:10.1210/jcem-48-2-192

775. Morrow, A.F., Gyorki, S., Warne, G.L., Burger, H.G., Bangah, M.L., Outch, K.H., Mirovics, A., Baker, H.W.: Variable androgen receptor levels in infertile men. J Clin Endocrinol Metab **64**(6), 1115-1121 (1987). doi:10.1210/jcem-64-6-1115

776. Morse, H.C., Horike, N., Rowley, M.J., Heller, C.G.: Testosterone concentrations in testes of normal men: effects of testosterone propionate administration. J Clin Endocrinol Metab **37**(6), 882-886 (1973). doi:10.1210/jcem-37-6-882

777. Mosaad, Y.M., Shahin, D., Elkholy, A.A., Mosbah, A., Badawy, W.: CAG repeat length in androgen receptor gene and male infertility in Egyptian patients. Andrologia **44**(1), 26-33 (2012). doi:10.1111/j.1439-0272.2010.01100.x

778. Mougios, V., Kouidi, E., Kyparos, A., Deligiannis, A.: Effect of exercise on the proportion of unsaturated fatty acids in serum of untrained middle aged individuals. Br J Sports Med **32**(1), 58-62 (1998). doi:10.1136/bjsm.32.1.58

779. Mouser, J.G., Loprinzi, P.D., Loenneke, J.P.: The association between physiologic testosterone levels, lean mass, and fat mass in a nationally representative sample of men in the United States. Steroids **115**, 62-66 (2016). doi:10.1016/j.steroids.2016.08.009

780. Müezzinoğu, T., Gümüş, B., Temeltaş, G., Ari, Z., Büyüksu, C.: A relationship of sex hormone levels and erectile dysfunction: which tests should be done routinely? Yonsei Med J **48**(6), 1015-1019 (2007). doi:10.3349/ymj.2007.48.6.1015

781. Mulati, Y., Li, X., Maimaitiming, A., Apizi, A., Wang, Y.: Is there any predictive value of testicular shear wave elastic modulus in testicular functions for varicocele patients? Andrologia **54**(5), e14393 (2022). doi:10.1055/a-1764-1260

10.1111/and.14393

782. Mulligan, T., Iranmanesh, A., Gheorghiu, S., Godschalk, M., Veldhuis, J.D.: Amplified nocturnal luteinizing hormone (LH) secretory burst frequency with selective attenuation of pulsatile (but not basal) testosterone secretion in healthy aged men: possible Leydig cell desensitization to endogenous LH signaling--a clinical research center study. J Clin Endocrinol Metab **80**(10), 3025-3031 (1995). doi:10.1210/jcem.80.10.7559891

783. Mulligan, T., Iranmanesh, A., Johnson, M.L., Straume, M., Veldhuis, J.D.: Aging alters feed-forward and feedback linkages between LH and testosterone in healthy men. Am J Physiol **273**(4), R1407-1413 (1997). doi:10.1152/ajpregu.1997.273.4.R1407

784. Munabi, A.K., Feuillan, P., Staton, R.C., Rodbard, D., Chrousos, G.P., Anderson, R.E., Strober, M.D., Loriaux, D.L., Cutler, G.B., Jr.: Adrenal steroid responses to continuous intravenous adrenocorticotropin infusion compared to bolus injection in normal volunteers. J Clin Endocrinol Metab **63**(4), 1036-1040 (1986). doi:10.1210/jcem-63-4-1036

785. Muneyyirci-Delale, O., Dalloul, M., Nacharaju, V.L., Altura, B.M., Altura, B.T.: Serum ionized magnesium and calcium and sex hormones in healthy young men: importance of serum progesterone level. Fertil Steril **72**(5), 817-822 (1999). doi:10.1016/s0015-0282(99)00386-6

786. Murono, E.P., Nankin, H.R., Lin, T., Osterman, J.: The aging Leydig cell: VI. Response of testosterone precursors to gonadotrophin in men. Acta Endocrinol (Copenh) **100**(3), 455-461 (1982). doi:10.1530/acta.0.1000455

787. Murono, E.P., Nankin, H.R., Lin, T., Osterman, J.: The aging Leydig cell V. Diurnal rhythms in aged men. Acta Endocrinol (Copenh) **99**(4), 619-623 (1982). doi:10.1530/acta.0.0990619

788. Mustafa, M., Horuz, R., Celik, M., Kucukcan, A.: Is there an association between serum prostate-specific antigen values and serum testosterone levels in healthy men? Korean J Urol **55**(7), 465-468 (2014). doi:10.4111/kju.2014.55.7.465

789. Naftolin, F., Petraglia, F., Simoncini, T.: The International Society of Gynecological Endocrinology and its president. Gynecol Endocrinol **28 Suppl 1**, 1 (2012). doi:10.3109/09513590.2012.652417

790. Nahoul, K., Roger, M.: Age-related decline of plasma bioavailable testosterone in adult men. J Steroid Biochem **35**(2), 293-299 (1990). doi:10.1016/0022-4731(90)90287-3

791. Naifar, M., Rekik, N., Messedi, M., Chaabouni, K., Lahiani, A., Turki, M., Abid, M., Ayedi, F., Jamoussi, K.: Male hypogonadism and metabolic syndrome. Andrologia **47**(5), 579-586 (2015). doi:10.1111/and.12305

792. Najafi, M.R., Ansari, B., Zare, M., Fatehi, F., Sonbolestan, A.: Effects of antiepileptic drugs on sexual function and reproductive hormones of male epileptic patients. Iran J Neurol **11**(2), 37-41 (2012).

793. Nakagawa, K., Obara, T., Matsubara, M., Kubo, M.: Relationship of changes in serum concentrations of prolactin and testosterone during dopaminergic modulation in males. Clin Endocrinol (Oxf) **17**(4), 345-352 (1982). doi:10.1111/j.1365-2265.1982.tb01599.x

794. Nakashima, A., Koshiyama, K., Uozumi, T., Monden, Y., Hamanaka, Y.: Effects of general anaesthesia and severity of surgical stress on serum LH and testosterone in males. Acta Endocrinol (Copenh) **78**(2), 258-269 (1975). doi:10.1530/acta.0.0780258

795. Nakhai Pour, H.R., Grobbee, D.E., Muller, M., Emmelot-Vonk, M., van der Schouw, Y.T.: Serum sex hormone and plasma homocysteine levels in middle-aged and elderly men. Eur J Endocrinol **155**(6), 887-893 (2006). doi:10.1530/eje.1.02303

796. Naliato, E.C., Violante, A.H., Gaccione, M., Caldas, D., Lamounier Filho, A., Loureiro, C.R., Fontes, R., Schrank, Y., Costa, F.S., Colao, A.: Body fat in men with prolactinoma. J Endocrinol Invest **31**(11), 985-990 (2008). doi:10.1007/bf03345636

797. Nankin, H.R., Calkins, J.H.: Decreased bioavailable testosterone in aging normal and impotent men. J Clin Endocrinol Metab **63**(6), 1418-1420 (1986). doi:10.1210/jcem-63-6-1418

798. Nankin, H.R., Lin, T., Murono, E., Osterman, J., Troen, P.: Testosterone and 17 OH-progesterone responses in men to 3 h LH infusions. Acta Endocrinol (Copenh) **95**(1), 110-116 (1980). doi:10.1530/acta.0.0950110

799. Nardozza Júnior, A., Szelbracikowski Sdos, S., Nardi, A.C., Almeida, J.C.: Age-related testosterone decline in a Brazilian cohort of healthy military men. Int Braz J Urol **37**(5), 591-597 (2011). doi:10.1590/s1677-55382011000500004

800. Nawata, H., Kato, K., Ibayashi, H.: Age-dependent change of serum 5alpha-dihydrotestosterone and its relation to testosterone in man. Endocrinol Jpn **24**(1), 41-45 (1977). doi:10.1507/endocrj1954.24.41

801. Neale, S.M., Hocking, R., Biswas, M., Turkes, A., Rees, D., Rees, D.A., Evans, C.: Adult testosterone and calculated free testosterone reference ranges by tandem mass spectrometry. Ann Clin Biochem **50**(Pt 2), 159-161 (2013). doi:10.1258/acb.2012.012047

802. Needham, B.L., Kim, C., Mukherjee, B., Bagchi, P., Stanczyk, F.Z., Kanaya, A.M.: Endogenous sex steroid hormones and glucose in a South-Asian population without diabetes: the Metabolic Syndrome and Atherosclerosis in South-Asians Living in America pilot study. Diabet Med **32**(9), 1193-1200 (2015). doi:10.1111/dme.12642

803. Negaresh, R., Ranjbar, R., Baker, J.S., Habibi, A., Mokhtarzade, M., Gharibvand, M.M., Fokin, A., Anupam, B., Shivaprasad, C., Sridevi, A., Aiswarya, Y., Gautham, K., Ramdas, B., Kejal, S.: Skeletal Muscle Hypertrophy, Insulin-like Growth Factor 1, Myostatin and Follistatin in Healthy and Sarcopenic Elderly Men: The Effect of Whole-body Resistance Training

Association of total and calculated free testosterone with androgen deficiency symptoms in patients with type 2 diabetes. Int J Prev Med **10**(3), 29 (2019). doi:10.4103/ijpvm.IJPVM_310_17

10.1038/s41443-019-0144-9

804. Nemet, D., Meckel, Y., Bar-Sela, S., Zaldivar, F., Cooper, D.M., Eliakim, A.: Effect of local cold-pack application on systemic anabolic and inflammatory response to sprint-interval training: a prospective comparative trial. Eur J Appl Physiol **107**(4), 411-417 (2009). doi:10.1007/s00421-009-1138-y

805. Neri, A., Aygen, M., Zukerman, Z., Bahary, C.: Subjective assessment of sexual dysfunction of patients on long-term administration of digoxin. Arch Sex Behav **9**(4), 343-347 (1980). doi:10.1007/bf01541359

806. Nestler, J.E., Barlascini, C.O., Clore, J.N., Blackard, W.G.: Dehydroepiandrosterone reduces serum low density lipoprotein levels and body fat but does not alter insulin sensitivity in normal men. J Clin Endocrinol Metab **66**(1), 57-61 (1988). doi:10.1210/jcem-66-1-57

807. Netter, A., Hartoma, R., Nahoul, K.: Effect of zinc administration on plasma testosterone, dihydrotestosterone, and sperm count. Arch Androl **7**(1), 69-73 (1981). doi:10.3109/01485018109009378

808. Neuwirth, J., Stárka, L., Raboch, J.: Different chromosome variants of Klinefelter's syndrome and plasma testosterone. Humangenetik **15**(1), 93-95 (1972). doi:10.1007/bf00273439

809. New, G., Berry, K.L., Cameron, J.D., Harper, R.W., Meredith, I.T.: Long-term oestrogen treatment does not alter systemic arterial compliance and haemodynamics in biological males. Coron Artery Dis **11**(3), 253-259 (2000). doi:10.1097/00019501-200005000-00008

810. Newmark, S.R., Rose, L.I., Todd, R., Birk, L., Naftolin, F.: Gonadotropin, estradiol, and testosterone profiles in homosexual men. Am J Psychiatry **136**(6), 767-771 (1979). doi:10.1176/ajp.136.6.767

811. Ng, T.P., Goh, H.H., Ng, Y.L., Ong, H.Y., Ong, C.N., Chia, K.S., Chia, S.E., Jeyaratnam, J.: Male endocrine functions in workers with moderate exposure to lead. Br J Ind Med **48**(7), 485-491 (1991). doi:10.1136/oem.48.7.485

812. Nguyen Hoai, B., Hoang, L., Tran, D., Nguyen Cao, T., Doan Tien, L., Sansone, A., Jannini, E.A.: Ultrasonic testicular size of 24,440 adult Vietnamese men and the correlation with age and hormonal profiles. Andrologia **54**(2), e14333 (2022). doi:10.1111/and.14333

813. Nicklas, B.J., Ryan, A.J., Treuth, M.M., Harman, S.M., Blackman, M.R., Hurley, B.F., Rogers, M.A.: Testosterone, growth hormone and IGF-I responses to acute and chronic resistive exercise in men aged 55-70 years. Int J Sports Med **16**(7), 445-450 (1995). doi:10.1055/s-2007-973035

814. Nieschlag, E., Lammers, U., Freischem, C.W., Langer, K., Wickings, E.J.: Reproductive functions in young fathers and grandfathers. J Clin Endocrinol Metab **55**(4), 676-681 (1982). doi:10.1210/jcem-55-4-676

815. Nisula, B.C., Loriaux, D.L., Wilson, Y.A.: Solid phase method for measurement of the binding capacity of testosterone-estradiol binding globulin in human serum. Steroids **31**(5), 681-690 (1978). doi:10.1016/s0039-128x(78)80008-7

816. Nixon AndrÃ©asson, A., JernelÃ¶v, S., Szulkin, R., UndÃ©n, A.L., Brismar, K., Lekander, M.: Associations between leptin and self-rated health in men and women. Gend Med **7**(3), 261-269 (2010). doi:10.1016/j.genm.2010.05.001

817. Nixon Andréasson, A., Jernelöv, S., Szulkin, R., Undén, A.L., Brismar, K., Lekander, M.: Associations between leptin and self-rated health in men and women. Gend Med **7**(3), 261-269 (2010). doi:10.1016/j.genm.2010.05.001

818. Nordkap, L., Jensen, T.K., Hansen Å, M., Lassen, T.H., Bang, A.K., Joensen, U.N., Blomberg Jensen, M., Skakkebæk, N.E., Jørgensen, N.: Psychological stress and testicular function: a cross-sectional study of 1,215 Danish men. Fertil Steril **105**(1), 174-187.e171-172 (2016). doi:10.1016/j.fertnstert.2015.09.016

819. Novo, S., Iacona, R., Bonomo, V., Evola, V., Corrado, E., Di Piazza, M., Novo, G., Pavone, C.: Erectile dysfunction is associated with low total serum testosterone levels and impaired flow-mediated vasodilation in intermediate risk men according to the Framingham risk score. Atherosclerosis **238**(2), 415-419 (2015). doi:10.1016/j.atherosclerosis.2014.12.007

820. Nowak, J., Pawłowski, B., Borkowska, B., Augustyniak, D., Drulis-Kawa, Z.: No evidence for the immunocompetence handicap hypothesis in male humans. Sci Rep **8**(1), 7392 (2018). doi:10.1038/s41598-018-25694-0

821. Nyquist, F., GÃ¤rdsell, P., Sernbo, I., Jeppsson, J.O., Johnell, O.: Assessment of sex hormones and bone mineral density in relation to occurrence of fracture in men: a prospective population-based study. Bone **22**(2), 147-151 (1998). doi:10.1016/s8756-3282(97)00250-0

822. Nyquist, F., Gärdsell, P., Sernbo, I., Jeppsson, J.O., Johnell, O.: Assessment of sex hormones and bone mineral density in relation to occurrence of fracture in men: a prospective population-based study. Bone **22**(2), 147-151 (1998). doi:10.1016/s8756-3282(97)00250-0

823. O'Connor, D.B., Lee, D.M., Corona, G., Forti, G., Tajar, A., O'Neill, T.W., Pendleton, N., Bartfai, G., Boonen, S., Casanueva, F.F., Finn, J.D., Giwercman, A., Han, T.S., Huhtaniemi, I.T., Kula, K., Labrie, F., Lean, M.E., Punab, M., Silman, A.J., Vanderschueren, D., Wu, F.C.: The relationships between sex hormones and sexual function in middle-aged and older European men. J Clin Endocrinol Metab **96**(10), E1577-1587 (2011). doi:10.1210/jc.2010-2216

824. Okita, K., Kanahara, N., Nishimura, M., Yoshida, T., Yasui-Furukori, N., Niitsu, T., Yoshida, T., Ishikawa, M., Kimura, H., Nomura, F., Iyo, M.: Second-generation antipsychotics and bone turnover in schizophrenia. Schizophr Res **157**(1-3), 137-141 (2014). doi:10.1016/j.schres.2014.05.009

825. Onuk, Ö., Arslan, B., Gezmis, T.C., Çetin, B., Göv, T., Yazıcı, G., Gürkan, O., Ozdemir, E.: Is the plasma endocan level a reliable predictor for the severity of erectile dysfunction? Int Urol Nephrol **50**(9), 1577-1582 (2018). doi:10.1007/s11255-018-1946-2

826. Oppenheim, D.S., Greenspan, S.L., Zervas, N.T., Schoenfeld, D.A., Klibanski, A.: Elevated serum lipids in hypogonadal men with and without hyperprolactinemia. Ann Intern Med **111**(4), 288-292 (1989). doi:10.7326/0003-4819-111-4-288

827. Osadchuk, L., Vasiliev, G., Kleshchev, M., Osadchuk, A.: Androgen Receptor Gene CAG Repeat Length Varies and Affects Semen Quality in an Ethnic-Specific Fashion in Young Men from Russia. Int J Mol Sci **23**(18) (2022). doi:10.3390/ijms231810594

828. Ottarsdottir, K., Nilsson, A.G., Hellgren, M., Lindblad, U., Daka, B., Gettler, L.T., Sarma, M.S., Gengo, R.G., Oka, R.C., McKenna, J.J.: The association between serum testosterone and insulin resistance: a longitudinal study

Testosterone moderates the effects of social support on cardiovascular disease risk factors among older US men. Endocr Connect **7**(12), 1491-1500 (2018). doi:10.1530/ec-18-0480

10.1002/ajhb.23248

829. Ou, S.Y., Luo, H.L., Mailman, R.B., Li, Z.C., Zhang, Y.W., Cai, M., Huang, X.W., Li, S.J., Jiang, Y.M.: Effect of manganese on neural endocrine hormones in serum of welders and smelters. J Trace Elem Med Biol **50**, 1-7 (2018). doi:10.1016/j.jtemb.2018.05.018

830. Ozasa, K., Nakao, M., Watanabe, Y., Hayashi, K., Miki, T., Mikami, K., Mori, M., Sakauchi, F., Washio, M., Ito, Y., Suzuki, K., Wakai, K., Tamakoshi, A.: Serum phytoestrogens and prostate cancer risk in a nested case-control study among Japanese men. Cancer Sci **95**(1), 65-71 (2004). doi:10.1111/j.1349-7006.2004.tb03172.x

831. Ozata, M., Bulur, M., Bingol, N., Beyhan, Z., Corakci, A., Bolu, E., Gundogan, M.A.: Daytime plasma melatonin levels in male hypogonadism. J Clin Endocrinol Metab **81**(5), 1877-1881 (1996). doi:10.1210/jcem.81.5.8626851

832. Oztekin, U., Caniklioglu, M., Sari, S., Gurel, A., Selmi, V., Isikay, L., Boeri, L., Capogrosso, P., Cazzaniga, W., Ventimiglia, E., Pozzi, E., Belladelli, F., Schifano, N., Candela, L., Alfano, M., Pederzoli, F., Abbate, C., Montanari, E., Valsecchi, L., Papaleo, E., Viganò, P., Rovere-Querini, P., Montorsi, F., Salonia, A.: The impact of body mass index on reproductive hormones, testosterone/estradiol ratio and semen parameters

Infertile Men Have Higher Prostate-specific Antigen Values than Fertile Individuals of Comparable Age. Cent European J Urol **73**(2), 226-230 (2020). doi:10.5173/ceju.2020.0020

10.1016/j.eururo.2020.08.001

833. Oztürk, M., Koca, O., Tüken, M., Keleş, M.O., Ilktaç, A., Karaman, M.I.: Hormonal evaluation in premature ejaculation. Urol Int **88**(4), 454-458 (2012). doi:10.1159/000336137

834. Paasch, U., Salzbrunn, A., Glander, H.J., Plambeck, K., Salzbrunn, H., Grunewald, S., Stucke, J., Vierula, M., Skakkebaek, N.E., JÃ¸rgensen, N.: Semen quality in sub-fertile range for a significant proportion of young men from the general German population: a co-ordinated, controlled study of 791 men from Hamburg and Leipzig. Int J Androl **31**(2), 93-102 (2008). doi:10.1111/j.1365-2605.2007.00860.x

835. Paasch, U., Salzbrunn, A., Glander, H.J., Plambeck, K., Salzbrunn, H., Grunewald, S., Stucke, J., Vierula, M., Skakkebaek, N.E., Jørgensen, N.: Semen quality in sub-fertile range for a significant proportion of young men from the general German population: a co-ordinated, controlled study of 791 men from Hamburg and Leipzig. Int J Androl **31**(2), 93-102 (2008). doi:10.1111/j.1365-2605.2007.00860.x

836. Paccou, J., Dewailly, J., Cortet, B.: Reduced levels of serum IGF-1 is related to the presence of osteoporotic fractures in male idiopathic osteoporosis. Joint Bone Spine **79**(1), 78-82 (2012). doi:10.1016/j.jbspin.2011.06.002

837. Pachman, A.: Studies on the role of sex-hormone-binding globulin (SHBG) in benign prostatic hypertrophy in men. I. Clinical research. Int Urol Nephrol **16**(2), 141-147 (1984). doi:10.1007/bf02082778

838. Padron, R.S., Wischusen, J., Hudson, B., Burger, H.G., de Kretser, D.M.: Prolonged biphasic response of plasma testosterone to single intramuscular injections of human chorionic gonadotropin. The Journal of clinical endocrinology and metabolism **50**(6), 1100-1104 (1980).

839. Padungtod, C., Savitz, D.A., Overstreet, J.W., Christiani, D.C., Ryan, L.M., Xu, X.: Occupational pesticide exposure and semen quality among Chinese workers. J Occup Environ Med **42**(10), 982-992 (2000). doi:10.1097/00043764-200010000-00004

840. Page, S.T., Kalhorn, T.F., Bremner, W.J., Anawalt, B.D., Matsumoto, A.M., Amory, J.K.: Intratesticular androgens and spermatogenesis during severe gonadotropin suppression induced by male hormonal contraceptive treatment. Journal of andrology **28**(5), 734-741 (2007). doi:10.2164/jandrol.107.002790

841. Page, S.T., Lin, D.W., Mostaghel, E.A., Hess, D.L., True, L.D., Amory, J.K., Nelson, P.S., Matsumoto, A.M., Bremner, W.J.: Persistent intraprostatic androgen concentrations after medical castration in healthy men. J Clin Endocrinol Metab **91**(10), 3850-3856 (2006). doi:10.1210/jc.2006-0968

842. Page, S.T., Lin, D.W., Mostaghel, E.A., Marck, B.T., Wright, J.L., Wu, J., Amory, J.K., Nelson, P.S., Matsumoto, A.M.: Dihydrotestosterone administration does not increase intraprostatic androgen concentrations or alter prostate androgen action in healthy men: a randomized-controlled trial. J Clin Endocrinol Metab **96**(2), 430-437 (2011). doi:10.1210/jc.2010-1865

843. Page, S.T., Plymate, S.R., Bremner, W.J., Matsumoto, A.M., Hess, D.L., Lin, D.W., Amory, J.K., Nelson, P.S., Wu, J.D.: Effect of medical castration on CD4+ CD25+ T cells, CD8+ T cell IFN-gamma expression, and NK cells: a physiological role for testosterone and/or its metabolites. Am J Physiol Endocrinol Metab **290**(5), E856-863 (2006). doi:10.1152/ajpendo.00484.2005

844. Palonek, E., Gottlieb, C., Garle, M., BjÃ¶rkhem, I., CarlstrÃ¶m, K.: Serum and urinary markers of exogenous testosterone administration. J Steroid Biochem Mol Biol **55**(1), 121-127 (1995). doi:10.1016/0960-0760(95)00146-q

845. Palonek, E., Gottlieb, C., Garle, M., Björkhem, I., Carlström, K.: Serum and urinary markers of exogenous testosterone administration. J Steroid Biochem Mol Biol **55**(1), 121-127 (1995). doi:10.1016/0960-0760(95)00146-q

846. Pan, D., Xu, Z.H., Gao, Q., Li, M., Guan, Y., Zhao, S.T.: Relationship between penile erection and the ratio of estradiol to testosterone: A retrospective study. Andrologia **52**(9), e13701 (2020). doi:10.1111/and.13701

847. Pandit, S.L., Yaligar, D., Halemane, M., Bhat, A.: A proprietary blend of standardized Punica granatum fruit rind and Theobroma cocoa seed extracts mitigates aging males' symptoms: A randomized, double-blind, placebo-controlled study. Int J Med Sci **19**(8), 1290-1299 (2022). doi:10.1093/gerona/glac162

10.7150/ijms.73645

848. Panuwet, P., Ladva, C., Barr, D.B., Prapamontol, T., Meeker, J.D., D'Souza, P.E., Maldonado, H., Ryan, P.B., Robson, M.G.: Investigation of associations between exposures to pesticides and testosterone levels in Thai farmers. Arch Environ Occup Health **73**(4), 205-218 (2018). doi:10.1080/19338244.2017.1378606

849. Paoli, A., Cenci, L., Pompei, P., Sahin, N., Bianco, A., Neri, M., Caprio, M., Moro, T.: Effects of Two Months of Very Low Carbohydrate Ketogenic Diet on Body Composition, Muscle Strength, Muscle Area, and Blood Parameters in Competitive Natural Body Builders. Nutrients **13**(2) (2021). doi:10.3390/nu13020374

850. Paolisso, G., Rizzo, M.R., Mone, C.M., Tagliamonte, M.R., Gambardella, A., Riondino, M., Carella, C., Varricchio, M., D'Onofrio, F.: Plasma sex hormones are significantly associated with plasma leptin concentration in healthy subjects. Clin Endocrinol (Oxf) **48**(3), 291-297 (1998). doi:10.1046/j.1365-2265.1998.00383.x

851. Papadimas, J., Mantalenakis, S.: Hormone profile in infertile men. Arch Androl **11**(1), 73-80 (1983). doi:10.3109/01485018308987463

852. Park, B.J., Shim, J.Y., Lee, Y.J., Lee, J.H., Lee, H.R.: Association between sex hormone levels and leukoaraiosis (LA) in older Korean men. Arch Gerontol Geriatr **54**(2), e73-76 (2012). doi:10.1016/j.archger.2011.06.035

853. Park, S.H., Park, S.W., Cha, B.Y., Park Ie, B., Min, K.W., Sung, Y.A., Kim, T.H., Lee, J.M., Park, K.S.: Comparison of the efficacy and safety of once-daily dosing and on-demand use of udenafil for type 2 diabetic patients with erectile dysfunction. Asian J Androl **17**(1), 143-148 (2015). doi:10.4103/1008-682x.135983

854. Parmigiani, S., Dadomo, H., Bartolomucci, A., Brain, P.F., Carbucicchio, A., Costantino, C., Ferrari, P.F., Palanza, P., Volpi, R.: Personality traits and endocrine response as possible asymmetry factors of agonistic outcome in karate athletes. Aggress Behav **35**(4), 324-333 (2009). doi:10.1002/ab.20306

855. Parra, M.D., Mendiola, J., Jørgensen, N., Swan, S.H., Torres-Cantero, A.M.: Anogenital distance and reproductive parameters in young men. Andrologia **48**(1), 3-10 (2016). doi:10.1111/and.12403

856. Parsons, J.K., Palazzi-Churas, K., Bergstrom, J., Barrett-Connor, E.: Prospective study of serum dihydrotestosterone and subsequent risk of benign prostatic hyperplasia in community dwelling men: the Rancho Bernardo Study. J Urol **184**(3), 1040-1044 (2010). doi:10.1016/j.juro.2010.05.033

857. Pasquali, R., Casimirri, F., Cantobelli, S., Melchionda, N., Morselli Labate, A.M., Fabbri, R., Capelli, M., Bortoluzzi, L.: Effect of obesity and body fat distribution on sex hormones and insulin in men. Metabolism: clinical and experimental **40**(1), 101-104 (1991). doi:10.1016/0026-0495(91)90199-7

858. Pasquali, R., Casimirri, F., De Iasio, R., Mesini, P., Boschi, S., Chierici, R., Flamia, R., Biscotti, M., Vicennati, V.: Insulin regulates testosterone and sex hormone-binding globulin concentrations in adult normal weight and obese men. J Clin Endocrinol Metab **80**(2), 654-658 (1995). doi:10.1210/jcem.80.2.7852532

859. Pasquali, R., Macor, C., Vicennati, V., Novo, F., De lasio, R., Mesini, P., Boschi, S., Casimirri, F., Vettor, R.: Effects of acute hyperinsulinemia on testosterone serum concentrations in adult obese and normal-weight men. Metabolism **46**(5), 526-529 (1997). doi:10.1016/s0026-0495(97)90189-x

860. Pasqualotto, F.F., Sobreiro, B.P., Hallak, J., Pasqualotto, E.B., Lucon, A.M.: Sperm concentration and normal sperm morphology decrease and follicle-stimulating hormone level increases with age. BJU Int **96**(7), 1087-1091 (2005). doi:10.1111/j.1464-410X.2005.05806.x

861. Pastuszak, A.W., Kohn, T.P., Estis, J., Lipshultz, L.I.: Low Plasma Testosterone Is Associated With Elevated Cardiovascular Disease Biomarkers. J Sex Med **14**(9), 1095-1103 (2017). doi:10.1136/bjsports-2017-097792

10.1016/j.jsxm.2017.06.015

862. Patel, P., Shiff, B., Kohn, T.P., Ramasamy, R.: Impaired sleep is associated with low testosterone in US adult males: results from the National Health and Nutrition Examination Survey. World J Urol **37**(7), 1449-1453 (2019). doi:10.1007/s00345-018-2485-2

863. Patnaik, N., Mishra, K.G., Pradhan, N.R., Giannos, P., Prokopidis, K., Church, D.D., Kirk, B., Morgan, P.T., Lochlainn, M.N., Macpherson, H., Woods, D.R., Ispoglou, T.: Evaluation of Serum Testosterone Levels Following Three Months of SA3X (Spilanthes acmella) Supplementation

Associations of Bioavailable Serum Testosterone With Cognitive Function in Older Men: Results From the National Health and Nutrition Examination Survey. Cureus **14**(6), e26236 (2022). doi:10.7759/cureus.26236

10.1093/gerona/glac162

864. Pearce, M.S., Groom, A., Relton, C.L., Peaston, R.T., Pollard, T.M., Francis, R.M.: Birth weight and early socio-economic disadvantage as predictors of sex hormones and sex hormone binding globulin in men at age 49-51 years. Am J Hum Biol **23**(2), 185-189 (2011). doi:10.1002/ajhb.21099

865. Pelzmann, K.S., Brodie, H.K.: Circulating plasma testosterone in the XYY male. Life Sci **18**(11), 1207-1212 (1976). doi:10.1016/0024-3205(76)90195-8

866. Peng, X.S., Li, F.D., Miao, Z.R., Ye, X.M., Wong, Y., Hu, X.Z., Zhong, Z.Z., Zeng, F.X., Wu, X.Q., Lan, J., et al.: Plasma reproductive hormones in normal and vasectomized Chinese males. Int J Androl **10**(2), 471-479 (1987). doi:10.1111/j.1365-2605.1987.tb00221.x

867. Pennanen, C., Laakso, M.P., Kivipelto, M., Ramberg, J., Soininen, H.: Serum testosterone levels in males with Alzheimer's disease. J Neuroendocrinol **16**(2), 95-98 (2004). doi:10.1111/j.0953-8194.2004.01133.x

868. Perry, H.M., 3rd, Horowitz, M., Fleming, S., Kaiser, F.E., Patrick, P., Morley, J.E., Cushman, W., Bingham, S., Perry, H.M., Jr.: Effect of recent alcohol intake on parathyroid hormone and mineral metabolism in men. Alcohol Clin Exp Res **22**(6), 1369-1375 (1998).

869. Persky, H., O'Brien, C.P., Fine, E., Howard, W.J., Khan, M.A., Beck, R.W.: The effect of alcohol and smoking on testosterone function and aggression in chronic alcoholics. The American journal of psychiatry **134**(6), 621-625 (1977). doi:10.1176/ajp.134.6.621

870. Pesonen, E., Pussinen, P., Huhtaniemi, I.: Adaptation to acute coronary syndrome-induced stress with lowering of testosterone: a possible survival factor. Eur J Endocrinol **174**(4), 481-489 (2016). doi:10.1530/eje-15-0757

871. Petermann, T.S., Cartes, A., Maliqueo, M., Vantman, D., GutiÃ©rrez, C., Toloza, H., EchiburÃº, B., Recabarren, S.E.: Patterns of hormonal response to the GnRH agonist leuprolide in brothers of women with polycystic ovary syndrome: a pilot study. Hum Reprod **19**(12), 2742-2747 (2004). doi:10.1093/humrep/deh512

872. Petermann, T.S., Cartes, A., Maliqueo, M., Vantman, D., Gutiérrez, C., Toloza, H., Echiburú, B., Recabarren, S.E.: Patterns of hormonal response to the GnRH agonist leuprolide in brothers of women with polycystic ovary syndrome: a pilot study. Hum Reprod **19**(12), 2742-2747 (2004). doi:10.1093/humrep/deh512

873. Petersen, M.S., Halling, J., Jørgensen, N., Nielsen, F., Grandjean, P., Jensen, T.K., Weihe, P.: Reproductive Function in a Population of Young Faroese Men with Elevated Exposure to Polychlorinated Biphenyls (PCBs) and Perfluorinated Alkylate Substances (PFAS). Int J Environ Res Public Health **15**(9) (2018). doi:10.3390/ijerph15091880

874. Petrikis, P., Tigas, S., Tzallas, A.T., Karampas, A., Papadopoulos, I., Skapinakis, P.: Sex hormone levels in drug-naïve, first-episode patients with psychosis. Int J Psychiatry Clin Pract **24**(1), 20-24 (2020). doi:10.1080/13651501.2019.1699117

875. Phang, M., Sinclair, A.J., Lincz, L.F., Garg, M.L.: Gender-specific inhibition of platelet aggregation following omega-3 fatty acid supplementation. Nutr Metab Cardiovasc Dis **22**(2), 109-114 (2012). doi:10.1016/j.numecd.2010.04.012

876. Phillips, G.B.: Oestrogens in ischaemic heart-disease. Lancet **2**(7991), 904 (1976). doi:10.1016/s0140-6736(76)90559-6

877. Phillips, G.B.: Evidence for hyperestrogenemia as the link between diabetes mellitus and myocardial infarction. Am J Med **76**(6), 1041-1048 (1984). doi:10.1016/0002-9343(84)90855-6

878. Phillips, G.B.: Relationship between serum dehydroepiandrosterone sulfate, androstenedione, and sex hormones in men and women. Eur J Endocrinol **134**(2), 201-206 (1996). doi:10.1530/eje.0.1340201

879. Pietschmann, P., Kudlacek, S., Grisar, J., Spitzauer, S., Woloszczuk, W., Willvonseder, R., Peterlik, M.: Bone turnover markers and sex hormones in men with idiopathic osteoporosis. Eur J Clin Invest **31**(5), 444-451 (2001). doi:10.1046/j.1365-2362.2001.00836.x

880. Piirainen, J.M., Tanskanen, M., Nissilä, J., Kaarela, J., Väärälä, A., Sippola, N., Linnamo, V.: Effects of a heart rate-based recovery period on hormonal, neuromuscular, and aerobic performance responses during 7 weeks of strength training in men. J Strength Cond Res **25**(8), 2265-2273 (2011). doi:10.1519/JSC.0b013e3181ecd050

881. Pilz, S., Frisch, S., Koertke, H., Kuhn, J., Dreier, J., Obermayer-Pietsch, B., Wehr, E., Zittermann, A.: Effect of vitamin D supplementation on testosterone levels in men. Horm Metab Res **43**(3), 223-225 (2011). doi:10.1055/s-0030-1269854

882. Pirke, K.M., Doerr, P.: Plasma dihydrotestosterone in normal adult males and its relation to testosterone. Acta Endocrinol (Copenh) **79**(2), 357-365 (1975). doi:10.1530/acta.0.0790357

883. Pirke, K.M., Doerr, P.: Age related changes in free plasma testosterone, dihydrotestosterone and oestradiol. Acta Endocrinol (Copenh) **80**(1), 171-178 (1975). doi:10.1530/acta.0.0800171

884. Pitteloud, N., Mootha, V.K., Dwyer, A.A., Hardin, M., Lee, H., Eriksson, K.F., Tripathy, D., Yialamas, M., Groop, L., Elahi, D., Hayes, F.J.: Relationship between testosterone levels, insulin sensitivity, and mitochondrial function in men. Diabetes care **28**(7), 1636-1642 (2005). doi:10.2337/diacare.28.7.1636

885. Poggi, U.L., Argüelles, A.E., Rosner, J., de Laborde, N.P., Cassini, J.H., Volmer, M.C.: Plasma testosterone and serum lipids in male survivors of myocardial infarction. J Steroid Biochem **7**(3), 229-231 (1976). doi:10.1016/0022-4731(76)90206-5

886. Polderman, K.H., Gooren, L.J., van der Veen, E.A.: Effects of gonadal androgens and oestrogens on adrenal androgen levels. Clin Endocrinol (Oxf) **43**(4), 415-421 (1995). doi:10.1111/j.1365-2265.1995.tb02611.x

887. Popa, F.L., Stanciu, M., Banciu, A., Berteanu, M.: ASSOCIATION BETWEEN LOW BONE MINERAL DENSITY, METABOLIC SYNDROME AND SEX STEROIDS DEFICIENCY IN MEN. Acta Endocrinol (Buchar) **12**(4), 418-422 (2016). doi:10.14814/phy2.14100

10.4183/aeb.2016.418

888. Pugh, P.J., Channer, K.S., Parry, H., Downes, T., Jone, T.H.: Bio-available testosterone levels fall acutely following myocardial infarction in men: association with fibrinolytic factors. Endocr Res **28**(3), 161-173 (2002). doi:10.1081/erc-120015055

889. Punab, M., Zilaitiene, B., JÃ¸rgensen, N., Horte, A., Matulevicius, V., Peetsalu, A., Skakkebaek, N.E.: Regional differences in semen qualities in the Baltic region. Int J Androl **25**(4), 243-252 (2002). doi:10.1046/j.1365-2605.2002.00359.x

890. Punab, M., Zilaitiene, B., Jørgensen, N., Horte, A., Matulevicius, V., Peetsalu, A., Skakkebaek, N.E.: Regional differences in semen qualities in the Baltic region. Int J Androl **25**(4), 243-252 (2002). doi:10.1046/j.1365-2605.2002.00359.x

891. Pusateri, D.J., Roth, W.T., Ross, J.K., Shultz, T.D.: Dietary and hormonal evaluation of men at different risks for prostate cancer: plasma and fecal hormone-nutrient interrelationships. Am J Clin Nutr **51**(3), 371-377 (1990). doi:10.1093/ajcn/51.3.371

892. Qin, D.D., Yuan, W., Zhou, W.J., Cui, Y.Q., Wu, J.Q., Gao, E.S.: Do reproductive hormones explain the association between body mass index and semen quality? Asian J Androl **9**(6), 827-834 (2007). doi:10.1111/j.1745-7262.2007.00268.x

893. Qing, X.R., Wan, C.C., Shang, X.J., Li, H.G., Xiong, C.L., Zhan, X.X., Mo, D.S., Cai, H.C., Zhang, H.P., Guan, H.T., Kong, X.B., Chen, Y.P., Liu, T.H., Hao, B.J., Zong, S.Y.: Relative contributions of testosterone deficiency and metabolism syndrome at the risk of reduced quality of life: A cross-sectional study among Chinese mid-aged and elderly men. Andrologia **49**(9) (2017). doi:10.1111/and.12736

894. Qoubaitary, A., Meriggiola, C., Ng, C.M., Lumbreras, L., Cerpolini, S., Pelusi, G., Christensen, P.D., Hull, L., Swerdloff, R.S., Wang, C.: Pharmacokinetics of testosterone undecanoate injected alone or in combination with norethisterone enanthate in healthy men. J Androl **27**(6), 853-867 (2006). doi:10.2164/jandrol.106.000281

895. Quinlan, P., Nordlund, A., Lind, K., Gustafson, D., Edman, A., Wallin, A.: Thyroid hormones are associated with poorer cognition in mild cognitive impairment. Dement Geriatr Cogn Disord **30**(3), 205-211 (2010). doi:10.1159/000319746

896. RÃ¤sÃ¤nen, P., Hakko, H., Visuri, S., Paanila, J., Kapanen, P., Suomela, T., Tiihonen, J.: Serum testosterone levels, mental disorders and criminal behaviour. Acta Psychiatr Scand **99**(5), 348-352 (1999). doi:10.1111/j.1600-0447.1999.tb07240.x

897. Raastad, T., Glomsheller, T., BjÃ¸ro, T., HallÃ©n, J.: Changes in human skeletal muscle contractility and hormone status during 2 weeks of heavy strength training. Eur J Appl Physiol **84**(1-2), 54-63 (2001). doi:10.1007/s004210000328

898. Raastad, T., Glomsheller, T., Bjøro, T., Hallén, J.: Changes in human skeletal muscle contractility and hormone status during 2 weeks of heavy strength training. Eur J Appl Physiol **84**(1-2), 54-63 (2001). doi:10.1007/s004210000328

899. Rabat, A., Gomez-Merino, D., Roca-Paixao, L., Bougard, C., Van Beers, P., Dispersyn, G., Guillard, M., Bourrilhon, C., Drogou, C., Arnal, P.J., Sauvet, F., Leger, D., Chennaoui, M.: Differential Kinetics in Alteration and Recovery of Cognitive Processes from a Chronic Sleep Restriction in Young Healthy Men. Front Behav Neurosci **10**, 95 (2016). doi:10.3389/fnbeh.2016.00095

900. Rabelo-Júnior, C.N., Freire de Carvalho, J., Lopes Gallinaro, A., Bonfá, E., Cocuzza, M., Saito, O., Silva, C.A.: Primary antiphospholipid syndrome: morphofunctional penile abnormalities with normal sperm analysis. Lupus **21**(3), 251-256 (2012). doi:10.1177/0961203311422715

901. Rabiee, A., Dwyer, A.A., Caronia, L.M., Hayes, F.J., Yialamas, M.A., Andersen, D.K., Thomas, B., Torriani, M., Elahi, D.: Impact of acute biochemical castration on insulin sensitivity in healthy adult men. Endocr Res **35**(2), 71-84 (2010). doi:10.3109/07435801003705601

902. Rabijewski, M., Papierska, L., Maksym, R., Tomasiuk, R., Kajdy, A., Siekierski, B.P.: The Relationship Between Health-Related Quality of Life and Anabolic Hormone Levels in Middle-Aged and Elderly Men With Prediabetes: A Cross-Sectional Study. Am J Mens Health **12**(5), 1593-1603 (2018). doi:10.1177/1557988318777926

903. Rabijewski, M., Papierska, L., Piątkiewicz, P.: The prevalence of prediabetes in population of Polish men with late-onset hypogonadism. The aging male : the official journal of the International Society for the Study of the Aging Male **17**(3), 141-146 (2014). doi:10.3109/13685538.2014.936000

904. Raboch, J., Mellan, J., Stárka, L.: Plasma testosterone in male patients with sexual dysfunction. Arch Sex Behav **4**(5), 541-545 (1975). doi:10.1007/bf01542132

905. Raboch, J., Pondelickova, J., Starka, L.: Plasma testosterone values in hypopspadiacs. Andrologia **8**(3), 255-258 (1976). doi:10.1111/j.1439-0272.1976.tb02144.x

906. Raboch, J., Reisenauer, R.: Analysis of body height in 829 patients with different forms of testicular pathology. Andrologia **8**(3), 265-268 (1976). doi:10.1111/j.1439-0272.1976.tb02148.x

907. Radellini, S., Guarnotta, V., Sciabica, V., Pizzolanti, G., Giordano, C.: Metabolic Profile in a Cohort of Young Sicilian Patients with Klinefelter's Syndrome: The Role of Irisin. Int J Endocrinol **2022**, 3780741 (2022). doi:10.1210/clinem/dgac218

10.1155/2022/3780741

908. Raiko, J.R., Oikonen, M., Wendelin-Saarenhovi, M., Siitonen, N., Kähönen, M., Lehtimäki, T., Viikari, J., Jula, A., Loo, B.M., Huupponen, R., Saarikoski, L., Juonala, M., Raitakari, O.T.: Plasminogen activator inhitor-1 associates with cardiovascular risk factors in healthy young adults in the Cardiovascular Risk in Young Finns Study. Atherosclerosis **224**(1), 208-212 (2012). doi:10.1016/j.atherosclerosis.2012.06.062

909. Rajandram, R., Koong, J.K., Quek, K.F., Lee, E.G., Razack, A.H.A., Kuppusamy, S.: Ethnic differences in serum testosterone concentration among Malay, Chinese and Indian men: A cross-sectional study. Clin Endocrinol (Oxf) **97**(3), 303-309 (2022). doi:10.1111/cen.14682

910. Rantala, M.J., Moore, F.R., Skrinda, I., Krama, T., Kivleniece, I., Kecko, S., Krams, I.: Evidence for the stress-linked immunocompetence handicap hypothesis in humans. Nat Commun **3**, 694 (2012). doi:10.1038/ncomms1696

911. Rapado, A., Hawkins, F., Sobrinho, L., DÃ­az-Curiel, M., Galvao-Telles, A., Arver, S., Melo Gomes, J., Mazer, N., Garcia e Costa, J., Horcajada, C., LÃ³pez-Gavilanes, E., Mascarenhas, M., Papapietro, K., LÃ³pez Alvarez, M.B., Pereira, M.C., Martinez, G., Valverde, I., GarcÃ­a, J.J., Carballal, J.J., GarcÃ­a, I.: Bone mineral density and androgen levels in elderly males. Calcif Tissue Int **65**(6), 417-421 (1999). doi:10.1007/s002239900726

912. Rapado, A., Hawkins, F., Sobrinho, L., Díaz-Curiel, M., Galvao-Telles, A., Arver, S., Melo Gomes, J., Mazer, N., Garcia e Costa, J., Horcajada, C., López-Gavilanes, E., Mascarenhas, M., Papapietro, K., López Alvarez, M.B., Pereira, M.C., Martinez, G., Valverde, I., García, J.J., Carballal, J.J., García, I.: Bone mineral density and androgen levels in elderly males. Calcif Tissue Int **65**(6), 417-421 (1999). doi:10.1007/s002239900726

913. Räsänen, P., Hakko, H., Visuri, S., Paanila, J., Kapanen, P., Suomela, T., Tiihonen, J.: Serum testosterone levels, mental disorders and criminal behaviour. Acta Psychiatr Scand **99**(5), 348-352 (1999). doi:10.1111/j.1600-0447.1999.tb07240.x

914. Rasmussen, B.B., Volpi, E., Gore, D.C., Wolfe, R.R.: Androstenedione does not stimulate muscle protein anabolism in young healthy men. J Clin Endocrinol Metab **85**(1), 55-59 (2000). doi:10.1210/jcem.85.1.6322

915. Rasmussen, J.J., Schou, M., Madsen, P.L., Selmer, C., Johansen, M.L., Ulriksen, P.S., Dreyer, T., Kümler, T., Plesner, L.L., Faber, J., Gustafsson, F., Kistorp, C.: Cardiac systolic dysfunction in past illicit users of anabolic androgenic steroids. Am Heart J **203**, 49-56 (2018). doi:10.1016/j.ahj.2018.06.010

916. Rasmussen, J.J., Selmer, C., Østergren, P.B., Pedersen, K.B., Schou, M., Gustafsson, F., Faber, J., Juul, A., Kistorp, C.: Former Abusers of Anabolic Androgenic Steroids Exhibit Decreased Testosterone Levels and Hypogonadal Symptoms Years after Cessation: A Case-Control Study. PLoS One **11**(8), e0161208 (2016). doi:10.1371/journal.pone.0161208

917. Raven, G., de Jong, F.H., Kaufman, J.M., de Ronde, W.: In men, peripheral estradiol levels directly reflect the action of estrogens at the hypothalamo-pituitary level to inhibit gonadotropin secretion. The Journal of clinical endocrinology and metabolism **91**(9), 3324-3328 (2006). doi:10.1210/jc.2006-0462

918. Recker, R.R., Akhter, M.P., Lappe, J.M., Watson, P.: Bone histomorphometry in transiliac biopsies from 48 normal, healthy men. Bone **111**, 109-115 (2018). doi:10.1371/journal.pone.0194395

10.1016/j.bone.2018.03.019

919. Reed, M.J., Cheng, R.W., Simmonds, M., Richmond, W., James, V.H.: Dietary lipids: an additional regulator of plasma levels of sex hormone binding globulin. J Clin Endocrinol Metab **64**(5), 1083-1085 (1987). doi:10.1210/jcem-64-5-1083

920. Reid, I.R., Ibbertson, H.K., France, J.T., Pybus, J.: Plasma testosterone concentrations in asthmatic men treated with glucocorticoids. Br Med J (Clin Res Ed) **291**(6495), 574 (1985). doi:10.1136/bmj.291.6495.574

921. Reis, R.M., de Angelo, A.G., Sakamoto, A.C., Ferriani, R.A., Lara, L.A.: Altered sexual and reproductive functions in epileptic men taking carbamazepine. J Sex Med **10**(2), 493-499 (2013). doi:10.1111/j.1743-6109.2012.02951.x

922. Reiter, E.O., Kulin, H.E.: Plasma testosterone response to short-term human chorionic gonadotropin administration in men with follicle-stimulating hormone suppressed by exogenous estrogen. Fertil Steril **26**(4), 340-345 (1975). doi:10.1016/s0015-0282(16)41055-1

923. Remes, K., Vuopio, P., Järvinen, M., Härkönen, M., Adlercreutz, H.: Effect of short-term treatment with an anabolic steroid (methandienone) and dehydroepiandrosterone sulphate on plasma hormones, red cell volume and 2,3-diphosphoglycerate in athletes. Scand J Clin Lab Invest **37**(7), 577-586 (1977). doi:10.3109/00365517709100649

924. Remes, T., VÃ¤isÃ¤nen, S.B., Mahonen, A., Huuskonen, J., KrÃ¶ger, H., Jurvelin, J.S., PenttilÃ¤, I.M., Rauramaa, R.: The association of bone metabolism with bone mineral density, serum sex hormone concentrations, and regular exercise in middle-aged men. Bone **35**(2), 439-447 (2004). doi:10.1016/j.bone.2004.04.020

925. Remes, T., Väisänen, S.B., Mahonen, A., Huuskonen, J., Kröger, H., Jurvelin, J.S., Penttilä, I.M., Rauramaa, R.: The association of bone metabolism with bone mineral density, serum sex hormone concentrations, and regular exercise in middle-aged men. Bone **35**(2), 439-447 (2004). doi:10.1016/j.bone.2004.04.020

926. Reverter, J.L., Colomé, E., Holgado, S., Aguilera, E., Soldevila, B., Mateo, L., Sanmartí, A.: Bone mineral density and bone fracture in male patients receiving long-term suppressive levothyroxine treatment for differentiated thyroid carcinoma. Endocrine **37**(3), 467-472 (2010). doi:10.1007/s12020-010-9339-z

927. Rezanezhad, B., Borgquist, R., Willenheimer, R., Elzanaty, S.: Association between serum levels of testosterone and biomarkers of subclinical atherosclerosis. Aging Male **21**(3), 182-186 (2018). doi:10.1080/13685538.2017.1412422

928. Rezayat, A.A., Asadpour, A.A., Yarahmadi, A., Ahmadnia, H., Hakkak, A.M., Soltani, S.: Association Between Serum Vitamin D Concentration with Spermiogram Parameters and Reproductive Hormones Among Infertile Iranian Males: a Cross-sectional Study. Reprod Sci **29**(1), 270-276 (2022). doi:10.1007/s43032-021-00771-4

929. Rhee, E.J., Oh, K.W., Lee, W.Y., Kim, S.W., Oh, E.S., Baek, K.H., Kang, M.I., Park, C.Y., Choi, M.G., Yoo, H.J., Park, S.W.: Age, body mass index, current smoking history, and serum insulin-like growth factor-I levels associated with bone mineral density in middle-aged Korean men. J Bone Miner Metab **22**(4), 392-398 (2004). doi:10.1007/s00774-003-0500-0

930. Rhoden, E.L., Riedner, C.E., Fuchs, S.C., Ribeiro, E.P., Halmenschlager, G.: A cross-sectional study for the analysis of clinical, sexual and laboratory conditions associated to Peyronie's disease. J Sex Med **7**(4 Pt 1), 1529-1537 (2010). doi:10.1111/j.1743-6109.2009.01584.x

931. Riahi, F., Izadi-Mazidi, M., Ghaffari, A., Yousefi, E., Khademvatan, S.: Comparison of Plasma Neurosteroid and Prolactin Levels in Patients with Schizophrenia and Healthy Individuals. Scientifica (Cairo) **2016**, 3108689 (2016). doi:10.1155/2016/3108689

932. Ribeiro, G.G., Phillips, H.V., Skinner, L.G.: Serum oestradiol-17 beta, testosterone, luteinizing hormone and follicle-stimulating hormone in males with breast cancer. Br J Cancer **41**(3), 474-477 (1980). doi:10.1038/bjc.1980.72

933. Richter, J.G., Becker, A., Specker, C., Schneider, M.: Hypogonadism in Wegener's granulomatosis. Scand J Rheumatol **37**(5), 365-369 (2008). doi:10.1080/03009740801998796

934. Rinieris, P., Hatzimanolis, J., Markianos, M., Stefanis, C.: Effects of 4 weeks treatment with chlorpromazine and/or trihexyphenidyl on the pituitary-gonadal axis in male paranoid schizophrenics. Eur Arch Psychiatry Neurol Sci **237**(4), 189-193 (1988). doi:10.1007/bf00449905

935. Ritsner, M., Gibel, A., Ram, E., Maayan, R., Weizman, A.: Alterations in DHEA metabolism in schizophrenia: two-month case-control study. Eur Neuropsychopharmacol **16**(2), 137-146 (2006). doi:10.1016/j.euroneuro.2005.07.007

936. Rizvi, S.J., Kennedy, S.H., Ravindran, L.N., Giacobbe, P., Eisfeld, B.S., Mancini, D., McIntyre, R.S.: The relationship between testosterone and sexual function in depressed and healthy men. J Sex Med **7**(2 Pt 1), 816-825 (2010). doi:10.1111/j.1743-6109.2009.01504.x

937. Rjosk, H.K., Schill, W.B.: Serum prolactin in male infertility. Andrologia **11**(4), 297-304 (1979). doi:10.1111/j.1439-0272.1979.tb02209.x

938. Roberts, A.C., McClure, R.D., Weiner, R.I., Brooks, G.A.: Overtraining affects male reproductive status. Fertil Steril **60**(4), 686-692 (1993). doi:10.1016/s0015-0282(16)56223-2

939. Robeva, R., Kirilov, G., Tomova, A., Kumanov, P.: Low testosterone levels and unimpaired melatonin secretion in young males with metabolic syndrome. Andrologia **38**(6), 216-220 (2006). doi:10.1111/j.1439-0272.2006.00743.x

940. Robeva, R., Tomova, A., Kirilov, G., Kumanov, P.: Anti-Müllerian hormone and inhibin B levels reflect altered Sertoli cell function in men with metabolic syndrome. Andrologia **44 Suppl 1**, 329-334 (2012). doi:10.1111/j.1439-0272.2011.01185.x

941. Robinson, M.R., Thomas, B.S.: Effect of hormonal therapy on plasma testosterone levels in prostatic carcinoma. Br Med J **4**(5784), 391-394 (1971). doi:10.1136/bmj.4.5784.391

942. Rodgers, S., Grosse Holtforth, M., Hengartner, M.P., Müller, M., Aleksandrowicz, A.A., Rössler, W., Ajdacic-Gross, V.: Serum testosterone levels and symptom-based depression subtypes in men. Front Psychiatry **6**, 61 (2015). doi:10.3389/fpsyt.2015.00061

943. Roelfsema, F., Yang, R.J., Olson, T.P., Joyner, M.J., Takahashi, P.Y., Veldhuis, J.D.: Enhanced Coupling Within Gonadotropic and Adrenocorticotropic Axes by Moderate Exercise in Healthy Men. J Clin Endocrinol Metab **102**(7), 2482-2490 (2017). doi:10.1210/jc.2017-00036

944. Röjdmark, S., Asplund, A., Rössner, S.: Pituitary-testicular axis in obese men during short-term fasting. Acta Endocrinol (Copenh) **121**(5), 727-732 (1989). doi:10.1530/acta.0.1210727

945. Rosano, G.M., Sheiban, I., Massaro, R., Pagnotta, P., Marazzi, G., Vitale, C., Mercuro, G., Volterrani, M., Aversa, A., Fini, M.: Low testosterone levels are associated with coronary artery disease in male patients with angina. Int J Impot Res **19**(2), 176-182 (2007). doi:10.1038/sj.ijir.3901504

946. Rose, R.M., Kreuz, L.E., Holaday, J.W., Sulak, K.J., Johnson, C.E.: Diurnal variation of plasma testosterone and cortisol. J Endocrinol **54**(1), 177-178 (1972). doi:10.1677/joe.0.0540177

947. Rosen, R., Tomer, Y., Carel, R., Weinberger, A.: Serum 17-beta-estradiol and testosterone levels in asymptomatic hyperuricaemic men. Clin Rheumatol **13**(2), 219-223 (1994). doi:10.1007/bf02249015

948. Rosen, S.G., Berk, M.A., Popp, D.A., Serusclat, P., Smith, E.B., Shah, S.D., Ginsberg, A.M., Clutter, W.E., Cryer, P.E.: beta 2- and alpha 2-adrenergic receptors and receptor coupling to adenylate cyclase in human mononuclear leukocytes and platelets in relation to physiological variations of sex steroids. J Clin Endocrinol Metab **58**(6), 1068-1076 (1984). doi:10.1210/jcem-58-6-1068

949. Rosmond, R., BjÃ¶rntorp, P.: The hypothalamic-pituitary-adrenal axis activity as a predictor of cardiovascular disease, type 2 diabetes and stroke. J Intern Med **247**(2), 188-197 (2000). doi:10.1046/j.1365-2796.2000.00603.x

950. Rosmond, R., Björntorp, P.: The hypothalamic-pituitary-adrenal axis activity as a predictor of cardiovascular disease, type 2 diabetes and stroke. J Intern Med **247**(2), 188-197 (2000). doi:10.1046/j.1365-2796.2000.00603.x

951. Roth, M.Y., Dudley, R.E., Hull, L., Leung, A., Christenson, P., Wang, C., Swerdloff, R., Amory, J.K.: Steady-state pharmacokinetics of oral testosterone undecanoate with concomitant inhibition of 5α-reductase by finasteride. Int J Androl **34**(6 Pt 1), 541-547 (2011). doi:10.1111/j.1365-2605.2010.01120.x

952. Rotter, I., Rył, A., Grzesiak, K., Szylińska, A., Pawlukowska, W., Lubkowska, A., Sipak-Szmigiel, O., Pabisiak, K., Laszczyńska, M.: Cross-Sectional Inverse Associations of Obesity and Fat Accumulation Indicators with Testosterone in Non-Diabetic Aging Men. Int J Environ Res Public Health **15**(6) (2018). doi:10.3390/ijerph15061207

953. Roulier, R., Mattei, A., Duvivier, J., Franchimont, P.: Measurement of gonadotrophins, testosterone, delta4 androstenedione and dihydrotesterone in idiopathic oligospermia. Clin Endocrinol (Oxf) **9**(4), 303-311 (1978). doi:10.1111/j.1365-2265.1978.tb02215.x

954. Rowland, D.L., Greenleaf, W.J., Dorfman, L.J., Davidson, J.M.: Aging and sexual function in men. Arch Sex Behav **22**(6), 545-557 (1993). doi:10.1007/bf01543300

955. Rucker, D., Ezzat, S., Diamandi, A., Khosravi, J., Hanley, D.A.: IGF-I and testosterone levels as predictors of bone mineral density in healthy, community-dwelling men. Clin Endocrinol (Oxf) **60**(4), 491-499 (2004). doi:10.1111/j.1365-2265.2004.02006.x

956. Rudnicka, A., Adoamnei, E., Noguera-Velasco, J.A., Vioque, J., Cañizares-Hernández, F., Mendiola, J., Jørgensen, N., Chavarro, J.E., Swan, S.H., Torres-Cantero, A.M.: Vitamin D status is not associated with reproductive parameters in young Spanish men. Andrology **8**(2), 323-331 (2020). doi:10.1111/andr.12690

957. Ryan, A.S., Treuth, M.S., Rubin, M.A., Miller, J.P., Nicklas, B.J., Landis, D.M., Pratley, R.E., Libanati, C.R., Gundberg, C.M., Hurley, B.F.: Effects of strength training on bone mineral density: hormonal and bone turnover relationships. J Appl Physiol (1985) **77**(4), 1678-1684 (1994). doi:10.1152/jappl.1994.77.4.1678

958. SÃ¶derberg, S., Olsson, T., Eliasson, M., Johnson, O., Brismar, K., CarlstrÃ¶m, K., AhrÃ©n, B.: A strong association between biologically active testosterone and leptin in non-obese men and women is lost with increasing (central) adiposity. Int J Obes Relat Metab Disord **25**(1), 98-105 (2001). doi:10.1038/sj.ijo.0801467

959. Sachar, E.J., Halpern, F., Rosenfeld, R.S., Galligher, T.F., Hellman, L.: Plasma and urinary testosterone levels in depressed men. Arch Gen Psychiatry **28**(1), 15-18 (1973). doi:10.1001/archpsyc.1973.01750310007001

960. Sachidhanandam, M., Singh, S.N., Salhan, A.K., Ray, U.S.: Evaluation of plasma hormone concentrations using Enzyme-Immunoassay/Enzyme-linked Immunosorbent assay in healthy Indian men: Effect of ethnicity. Indian J Clin Biochem **25**(2), 153-157 (2010). doi:10.1007/s12291-010-0028-8

961. Sadowsky, M., Antonovsky, H., Sobel, R., Maoz, B.: Sexual activity and sex hormone levels in aging men. Int Psychogeriatr **5**(2), 181-186 (1993). doi:10.1017/s1041610293001516

962. Safarinejad, M.R.: Evaluation of semen quality, endocrine profile and hypothalamus-pituitary-testis axis in male patients with homozygous beta-thalassemia major. J Urol **179**(6), 2327-2332 (2008). doi:10.1016/j.juro.2008.01.103

963. Safarinejad, M.R.: Evaluation of endocrine profile, hypothalamic-pituitary-testis axis and semen quality in multiple sclerosis. J Neuroendocrinol **20**(12), 1368-1375 (2008). doi:10.1111/j.1365-2826.2008.01791.x

964. Safarinejad, M.R.: Evaluation of endocrine profile and hypothalamic-pituitary-testis axis in selective serotonin reuptake inhibitor-induced male sexual dysfunction. J Clin Psychopharmacol **28**(4), 418-423 (2008). doi:10.1097/JCP.0b013e31817e6f80

965. Safarinejad, M.R., Kolahi, A.A., Iravani, S.: Evaluation of semen variables, sperm chromosomal abnormalities and reproductive endocrine profile in patients with chronic hepatitis C. BJU Int **105**(1), 79-86 (2010). doi:10.1111/j.1464-410X.2009.08720.x

966. Safarinejad, M.R., Safarinejad, S., Bahk, J.Y., Jung, J.H., Jin, L.M., Min, S.K.: Plasma Chitotriosidase Activity and Arteriogenic Erectile Dysfunction: Association with the Presence, Severity, and Duration

Cut-off value of testes volume in young adults and correlation among testes volume, body mass index, hormonal level, and seminal profiles. J Sex Med **75**(6), 1318-1323 (2010). doi:10.1111/j.1743-6109.2009.01673.x

10.1016/j.urology.2009.12.007

967. Safarinejad, M.R., Safarinejad, S., Shafiei, N., Safarinejad, S.: Estrogen receptors alpha (rs2234693 and rs9340799), and beta (rs4986938 and rs1256049) genes polymorphism in prostate cancer: evidence for association with risk and histopathological tumor characteristics in Iranian men. Mol Carcinog **51 Suppl 1**, E104-117 (2012). doi:10.1002/mc.21870

968. Sagiroglu, S., Kılınc, M., Doganer, A., Bilal, N., Orhan, I., Kılıc, M.A.: G protein coupled oestrogen receptor 1, aromatase, 17β-HSD and cAMP level in mutational falsetto. Eur Arch Otorhinolaryngol **277**(4), 1121-1127 (2020). doi:10.1007/s00405-020-05795-1

969. Saha, S., Goswami, R., Ramakrishnan, L., Vishnubhatla, S., Mahtab, S., Kar, P., Srinivasan, S., Singh, N., Singh, U.: Vitamin D and calcium supplementation, skeletal muscle strength and serum testosterone in young healthy adult males: Randomized control trial. Clin Endocrinol (Oxf) **88**(2), 217-226 (2018). doi:10.1111/cen.13507

970. Saijo, H., Kitamura, T., Fujiwara, H., Nagata, O., Hagiwara-Oguchi, K., Ide, Y., Tagami, M., Hanaoka, K.: [Anesthetic management for gastrojejunostomy in a patient with hemiplegia and recurrent laryngeal nerve palsy]. Masui **50**(6), 662-665 (2001).

971. Saka, T., Sofikerim, M., Demirtas, A., Kulaksizoglu, S., Caniklioglu, M., Karacagil, M.: Rigorous bicycling does not increase serum levels of total and free prostate-specific antigen (PSA), the free/total PSA ratio, gonadotropin levels, or uroflowmetric parameters. Urology **74**(6), 1325-1330 (2009). doi:10.1016/j.urology.2009.07.1219

972. Sakamoto, H., Ogawa, Y., Yoshida, H.: Relationship between testicular volume and testicular function: comparison of the Prader orchidometric and ultrasonographic measurements in patients with infertility. Asian J Androl **10**(2), 319-324 (2008). doi:10.1111/j.1745-7262.2008.00340.x

973. Salama, N., Blgozah, S., Janke, E., Groh, A., Mühle, C., Dürsteler-MacFarland, K.M., Wiesbeck, G.A., Kornhuber, J., Jahn, K., Groeschl, M., Lichtinghagen, R., Frieling, H., Bleich, S., Hillemacher, T., Lenz, B., Heberlein, A.: Serum estradiol levels in infertile men with non-obstructive azoospermia

Association of Testosterone Levels and Steroid 5-Alpha-Reductase 2 Polymorphisms with Opioid Craving. Ther Adv Reprod Health **14**(1), 2633494120928342 (2020). doi:10.1177/2633494120928342

10.1159/000508681

974. Salama, N., Samir, M., Blgozah, S.: Evaluation of Normal and Varicocele-Bearing Testes Using Real-time Strain Elastography. J Ultrasound Med **38**(3), 621-627 (2019). doi:10.1002/jum.14730

975. Salameh, W., Bhasin, S., Steiner, B., McAdams, L.A., Peterson, M., Swerdloff, R.: Marked suppression of gonadotropins and testosterone by an antagonist analog of gonadotropin-releasing hormone in men. Fertility and sterility **55**(1), 156-164 (1991).

976. Saleem, T.H., Okasha, M., Ibrahim, H.M., Abu El-Hamd, M., Fayed, H.M., Hassan, M.H.: Biochemical Assessments of Seminal Plasma Zinc, Testis-Expressed Sequence 101 and Free Amino Acids and Their Correlations with Reproductive Hormones in Male Infertility. Biol Trace Elem Res **199**(5), 1729-1742 (2021). doi:10.1007/s12011-020-02310-9

977. Saltiki, K., Papageorgiou, G., Voidonikola, P., Mantzou, E., Xiromeritis, K., Papamichael, C., Alevizaki, M., Stamatelopoulos, K.: Endogenous estrogen levels are associated with endothelial function in males independently of lipid levels. Endocrine **37**(2), 329-335 (2010). doi:10.1007/s12020-010-9307-7

978. Samietz, S., Holtfreter, B., Friedrich, N., Mundt, T., Hoffmann, W., VÃ¶lzke, H., Nauck, M., Kocher, T., Biffar, R.: Prospective association of sex steroid concentrations with periodontal progression and incident tooth loss. J Clin Periodontol **43**(1), 10-18 (2016). doi:10.1111/jcpe.12493

979. Samipoor, F., Pakseresht, S., Rezasoltani, P., Mehrdad, M.: The association between hypogonadism symptoms with serum testosterone, FSH and LH in men. Aging Male **21**(1), 1-8 (2018). doi:10.1080/13685538.2017.1382468

980. Sancini, A., De Sio, S., Gioffrè, P.A., Casale, T., Giubilati, R., Pimpinella, B., Scala, B., Suppi, A., Bonomi, S., Samperi, I., Rosati, M.V., Tomei, G., Tomei, F., Caciari, T.: Correlation between urinary nickel and testosterone plasma values in workers occupationally exposed to urban stressors. Ann Ig **26**(3), 237-254 (2014). doi:10.7416/ai.2014.1982

981. Santen, R.J., DeKretser, D.M., Paulsen, C.A., Vorhees, J.: Gonadotrophins and testosterone in the XYY syndrome. Lancet **2**(7668), 371 (1970). doi:10.1016/s0140-6736(70)92911-9

982. Saroff, J., Kirdani, R.Y., Chu, T.M., Wajsman, Z., Murphy, G.P.: Measurements of prolactin and androgens in patients with prostatic diseases. Oncology **37**(1), 46-52 (1980). doi:10.1159/000225401

983. Sasagawa, I., Tateno, T., Suzuki, Y., Yazawa, H., Ichiyanagi, O., Nakada, T., Miura, H.: Circulating levels of inhibin in hemodialysis males. Arch Androl **41**(3), 167-171 (1998). doi:10.3109/01485019808994887

984. Sawada, N., Iwasaki, M., Inoue, M., Sasazuki, S., Yamaji, T., Shimazu, T., Tsugane, S.: Plasma testosterone and sex hormone-binding globulin concentrations and the risk of prostate cancer among Japanese men: a nested case-control study. Cancer Sci **101**(12), 2652-2657 (2010). doi:10.1111/j.1349-7006.2010.01721.x

985. Sawhney, R.C., Chhabra, P.C., Malhotra, A.S., Singh, T., Riar, S.S., Rai, R.M.: Hormone profiles at high altitude in man. Andrologia **17**(2), 178-184 (1985). doi:10.1111/j.1439-0272.1985.tb00983.x

986. Sawhney, R.C., Malhotra, A.S., Prasad, R., Pal, K., Kumar, R., Bajaj, A.C.: Pituitary-gonadal hormones during prolonged residency in Antarctica. International journal of biometeorology **42**(1), 51-54 (1998).

987. Scaglia, H.E., Carrere, C.A., Mariani, V.A., Zylbersztein, C.C., Rey-Valzacchi, G.J., Kelly, E.E., Aquilano, D.R.: Altered testicular hormone production in infertile patients with idiopathic oligoasthenospermia. J Androl **12**(5), 273-280 (1991).

988. Schaison, G., Durand, F., Mowszowicz, I.: Effect of glucocorticoids on plasma testosterone in men. Acta Endocrinol (Copenh) **89**(1), 126-131 (1978). doi:10.1530/acta.0.0890126

989. Schatzl, G., Madersbacher, S., Temml, C., Krenn-Schinkel, K., Nader, A., Sregi, G., Lapin, A., Hermann, M., Berger, P., Marberger, M.: Serum androgen levels in men: impact of health status and age. Urology **61**(3), 629-633 (2003). doi:10.1016/s0090-4295(02)02252-5

990. Schiavi, R.C., Fisher, C., White, D., Beers, P., Fogel, M., Szechter, R.: Hormonal variations during sleep in men with erectile dysfunction and normal controls. Arch Sex Behav **11**(3), 189-200 (1982). doi:10.1007/bf01544987

991. Schiavi, R.C., Schreiner-Engel, P., White, D., Mandeli, J.: Pituitary-gonadal function during sleep in men with hypoactive sexual desire and in normal controls. Psychosom Med **50**(3), 304-318 (1988). doi:10.1097/00006842-198805000-00008

992. Schiavi, R.C., Schreiner-Engel, P., White, D., Mandeli, J.: The relationship between pituitary-gonadal function and sexual behavior in healthy aging men. Psychosom Med **53**(4), 363-374 (1991). doi:10.1097/00006842-199107000-00002

993. Schiavi, R.C., White, D., Mandeli, J., Schreiner-Engel, P.: Hormones and nocturnal penile tumescence in healthy aging men. Arch Sex Behav **22**(3), 207-215 (1993). doi:10.1007/bf01541766

994. Schmidt, J.B.: Hormonal basis of male and female androgenic alopecia: clinical relevance. Skin Pharmacol **7**(1-2), 61-66 (1994). doi:10.1159/000211275

995. Schneider, G., Nienhaus, K., Gromoll, J., Heuft, G., Nieschlag, E., Zitzmann, M.: Aging males' symptoms in relation to the genetically determined androgen receptor CAG polymorphism, sex hormone levels and sample membership. Psychoneuroendocrinology **35**(4), 578-587 (2010). doi:10.1016/j.psyneuen.2009.09.008

996. Schneider, G., Nienhaus, K., Gromoll, J., Heuft, G., Nieschlag, E., Zitzmann, M.: Sex hormone levels, genetic androgen receptor polymorphism, and anxiety in ≥50-year-old males. J Sex Med **8**(12), 3452-3464 (2011). doi:10.1111/j.1743-6109.2011.02443.x

997. Schrader, S.M., Langford, R.E., Turner, T.W., Breitenstein, M.J., Clark, J.C., Jenkins, B.L., Lundy, D.O., Simon, S.D., Weyandt, T.B.: Reproductive function in relation to duty assignments among military personnel. Reprod Toxicol **12**(4), 465-468 (1998). doi:10.1016/s0890-6238(98)00023-9

998. Schumann, M., Mykkänen, O.P., Doma, K., Mazzolari, R., Nyman, K., Häkkinen, K.: Effects of endurance training only versus same-session combined endurance and strength training on physical performance and serum hormone concentrations in recreational endurance runners. Appl Physiol Nutr Metab **40**(1), 28-36 (2015). doi:10.1139/apnm-2014-0262

999. Schürmeyer, T., Knuth, U.A., Freischem, C.W., Sandow, J., Akhtar, F.B., Nieschlag, E.: Suppression of pituitary and testicular function in normal men by constant gonadotropin-releasing hormone agonist infusion. J Clin Endocrinol Metab **59**(1), 19-24 (1984). doi:10.1210/jcem-59-1-19

1000. Schwartz, M.F., Kolodny, R.C., Masters, W.H.: Plasma testosterone levels of sexually functional and dysfunctional men. Arch Sex Behav **9**(5), 355-366 (1980). doi:10.1007/bf02115938

1001. Sedliak, M., Finni, T., Cheng, S., Kraemer, W.J., HÃ¤kkinen, K.: Effect of time-of-day-specific strength training on serum hormone concentrations and isometric strength in men. Chronobiol Int **24**(6), 1159-1177 (2007). doi:10.1080/07420520701800686

1002. Sedliak, M., Finni, T., Cheng, S., Kraemer, W.J., Häkkinen, K.: Effect of time-of-day-specific strength training on serum hormone concentrations and isometric strength in men. Chronobiol Int **24**(6), 1159-1177 (2007). doi:10.1080/07420520701800686

1003. Segal, K.R., Dunaif, A., Gutin, B., Albu, J., Nyman, A., Pi-Sunyer, F.X.: Body composition, not body weight, is related to cardiovascular disease risk factors and sex hormone levels in men. J Clin Invest **80**(4), 1050-1055 (1987). doi:10.1172/jci113159

1004. Semmens, J., Rouse, I., Beilin, L.J., Masarei, J.R.: Relationship of plasma HDL-cholesterol to testosterone, estradiol, and sex-hormone-binding globulin levels in men and women. Metabolism **32**(5), 428-432 (1983). doi:10.1016/0026-0495(83)90002-1

1005. Semple, C.G., Beastall, G.H., Henderson, I.S., Thomson, J.A., Kennedy, A.C.: The pituitary-testicular axis of uraemic subjects on haemodialysis and continuous ambulatory peritoneal dialysis. Acta Endocrinol (Copenh) **101**(3), 464-467 (1982). doi:10.1530/acta.0.1010464

1006. Semple, C.G., Thomson, J.A., Beastall, G.H.: Endocrine responses to marathon running. Br J Sports Med **19**(3), 148-151 (1985). doi:10.1136/bjsm.19.3.148

1007. Sewdarsen, M., Jialal, I., Naidu, R.K.: The low plasma testosterone levels of young Indian infarct survivors are not due to a primary testicular defect. Postgrad Med J **64**(750), 264-266 (1988). doi:10.1136/pgmj.64.750.264

1008. Seyfart, T., Friedrich, N., Kische, H., Bülow, R., Wallaschofski, H., Völzke, H., Nauck, M., Keevil, B.G., Haring, R.: Association of sex hormones with physical, laboratory, and imaging markers of anthropometry in men and women from the general population. PLoS One **13**(1), e0189042 (2018). doi:10.1371/journal.pone.0189042

1009. Shakhssalim, N., Gilani, K.R., Parvin, M., Torbati, P.M., Kashi, A.H., Azadvari, M., Golestan, B., Basiri, A.: An assessment of parathyroid hormone, calcitonin, 1,25 (OH)2 vitamin D3, estradiol and testosterone in men with active calcium stone disease and evaluation of its biochemical risk factors. Urol Res **39**(1), 1-7 (2011). doi:10.1007/s00240-010-0276-3

1010. Shamim, M.O., Ali Khan, F.M., Arshad, R.: Association between serum total testosterone and Body Mass Index in middle aged healthy men. Pak J Med Sci **31**(2), 355-359 (2015). doi:10.12669/pjms.312.6130

1011. Shen, X., Wang, R., Yu, N., Shi, Y., Li, H., Xiong, C., Li, Y., Wells, E.M., Zhou, Y.: Reference Ranges and Association of Age and Lifestyle Characteristics with Testosterone, Sex Hormone Binding Globulin, and Luteinizing Hormone among 1166 Western Chinese Men. PLoS One **11**(10), e0164116 (2016). doi:10.1371/journal.pone.0164116

1012. Sherk, V.D., Sherk, K.A., Kim, S., Young, K.C., Bemben, D.A.: Hormone responses to a continuous bout of rock climbing in men. Eur J Appl Physiol **111**(4), 687-693 (2011). doi:10.1007/s00421-010-1685-2

1013. Shi, M.D., Chao, J.K., Ma, M.C., Chiang, S.K., Chao, I.C.: The connection between type 2 diabetes and erectile dysfunction in Taiwanese aboriginal males. Int J Impot Res **26**(6), 235-240 (2014). doi:10.1038/ijir.2014.26

1014. Shim, J.S., Kim, J.H., Yoon, Y.S., Choi, H., Park, J.Y., Bae, J.H.: Serum Testosterone Levels Are Negatively Correlated with International Prostate Symptom Score and Transitional Prostate Volume. Low Urin Tract Symptoms **10**(2), 143-147 (2018). doi:10.1111/luts.12150

1015. Shin, J., Sung, J., Lee, K., Song, Y.M.: Genetic influence on the association between bone mineral density and testosterone in Korean men. Osteoporos Int **27**(2), 643-651 (2016). doi:10.1007/s00198-015-3298-4

1016. Shin, Y.S., You, J.H., Cha, J.S., Park, J.K.: The relationship between serum total testosterone and free testosterone levels with serum hemoglobin and hematocrit levels: a study in 1221 men. Aging Male **19**(4), 209-214 (2016). doi:10.1080/13685538.2016.1229764

1017. Shkelzen, E., Paić, F., Stipoljev, F., Gashi, Z., Zeqiraj, A., Lila, A., Nikuševa Martić, T.: THE FREQUENCY OF FOLLICLE-STIMULATING HORMONE RECEPTOR 2039A>G GENE POLYMORPHISM AND THE RISK OF MALE INFERTILITY IN ALBANIAN POPULATION. Acta Clin Croat **59**(1), 37-49 (2020). doi:10.20471/acc.2020.59.01.05

1018. Shlykova, N., Davidson, E., Krakowsky, Y., Bolanos, J., Traish, A., Morgentaler, A.: Absent Diurnal Variation in Serum Testosterone in Young Men with Testosterone Deficiency. J Urol **203**(4), 817-823 (2020). doi:10.1097/ju.0000000000000630

1019. Shukla, A., Sharda, B., Sharma, S., Bhardwaj, S., Kailash, U., Kalani, R., Satyanarayana, L., Shrivastava, A., Lokeshwar, S.D., Patel, P., Fantus, R.J., Halpern, J., Chang, C., Kargi, A.Y., Ramasamy, R.: Association Between Serum Testosterone and Serum PSA Among Men With and Without Partial Androgen Deficiency

Decline in Serum Testosterone Levels Among Adolescent and Young Adult Men in the USA. Indian J Clin Biochem **35**(1), 127-131 (2020). doi:10.1007/s12291-018-0785-3

10.1016/j.euf.2020.02.006

1020. Siddique, S., Farhat, I., Kubwabo, C., Chan, P., Goodyer, C.G., Robaire, B., Chevrier, J., Hales, B.F.: Exposure of men living in the greater Montreal area to organophosphate esters: Association with hormonal balance and semen quality. Environ Int **166**, 107402 (2022). doi:10.1016/j.envint.2022.107402

1021. Sigman, M., Jarow, J.P.: Endocrine evaluation of infertile men. Urology **50**(5), 659-664 (1997). doi:10.1016/s0090-4295(97)00340-3

1022. Sigurjonsdottir, H.A., Axelson, M., Johannsson, G., Manhem, K., Nystrom, E., Wallerstedt, S.: Liquorice in moderate doses does not affect sex steroid hormones of biological importance although the effect differs between the genders. Horm Res **65**(2), 106-110 (2006). doi:10.1159/000091302

1023. Silva, A.L., Carmo, F., Bugalho, M.J.: RAC1b overexpression in papillary thyroid carcinoma: a role to unravel. Eur J Endocrinol **168**(6), 795-804 (2013). doi:10.1530/eje-12-0960

1024. Silva, G.P., Carneiro, F.P., Grangeiro, V.P.X.: Seminal transferrin in the seminal quality evaluation of hemodialytic patients. Arch Ital Urol Androl **90**(1), 49-53 (2018). doi:10.4081/aiua.2018.1.49

1025. Sim, M.Y., Kim, S.H., Kim, K.M., Holmboe, S.A., Skakkebæk, N.E., Juul, A., Scheike, T., Jensen, T.K., Linneberg, A., Thuesen, B.H., Andersson, A.M.: Seasonal Variations and Correlations between Vitamin D and Total Testosterone Levels

Individual testosterone decline and future mortality risk in men. Korean J Fam Med **38**(5), 270-275 (2017). doi:10.4082/kjfm.2017.38.5.270

10.1530/eje-17-0280

1026. Simão, R., Leite, R.D., Speretta, G.F., Maior, A.S., de Salles, B.F., de Souza Junior, T.P., Vingren, J.L., Willardson, J.M.: Influence of upper-body exercise order on hormonal responses in trained men. Appl Physiol Nutr Metab **38**(2), 177-181 (2013). doi:10.1139/apnm-2012-0040

1027. Simoni, M., Baraldi, E., Baraghini, G.F., Boraldi, V., Roli, L., Seghedoni, S., Velardo, A., Montanini, V.: Twenty-four-hour pattern of plasma SHBG, total proteins and testosterone in young and elderly men. Steroids **52**(4), 381-382 (1988). doi:10.1016/0039-128x(88)90157-2

1028. Simoni, M., Montanini, V., Fustini, M.F., Del Rio, G., Cioni, K., Marrama, P.: Circadian rhythm of plasma testosterone in men with idiopathic hypogonadotrophic hypogonadism before and during pulsatile administration of gonadotrophin-releasing hormone. Clin Endocrinol (Oxf) **36**(1), 29-34 (1992). doi:10.1111/j.1365-2265.1992.tb02899.x

1029. Sina, D., Schuhmann, R., Abraham, R., Taubert, H.D., Dericks-Tan, J.S.: Increased serum FSH levels correlated with low and high sperm counts in male infertile patients. Andrologia **7**(1), 31-37 (1975). doi:10.1111/j.1439-0272.1975.tb01223.x

1030. Sinclair, M., Gow, P.J., Angus, P.W., Hoermann, R., Handelsman, D.J., Wittert, G., Martin, S., Grossmann, M.: High circulating oestrone and low testosterone correlate with adverse clinical outcomes in men with advanced liver disease. Liver Int **36**(11), 1619-1627 (2016). doi:10.1111/liv.13122

1031. Singer, F., Zumoff, B.: Subnormal serum testosterone levels in male internal medicine residents. Steroids **57**(2), 86-89 (1992). doi:10.1016/0039-128x(92)90035-8

1032. Singh, S.K., Goyal, R., Pratyush, D.D.: Is hypoandrogenemia a component of metabolic syndrome in males? Exp Clin Endocrinol Diabetes **119**(1), 30-35 (2011). doi:10.1055/s-0030-1261918

1033. Skiba, R., Matyjek, A., Syryło, T., Niemczyk, S., Rymarz, A., Temiz, M.Z., Dincer, M.M., Hacibey, I., Yazar, R.O., Celik, C., Kucuk, S.H., Alkurt, G., Doganay, L., Yuruk, E., Muslumanoglu, A.Y.: Advanced Chronic Kidney Disease is a Strong Predictor of Hypogonadism and is Associated with Decreased Lean Tissue Mass

Investigation of SARS-CoV-2 in semen samples and the effects of COVID-19 on male sexual health by using semen analysis and serum male hormone profile: A cross-sectional, pilot study. Int J Nephrol Renovasc Dis **13**(2), 319-327 (2020). doi:10.2147/ijnrd.S275554

10.1111/and.13912

1034. Skiba, R., Rymarz, A., Matyjek, A., Dymus, J., Woźniak-Kosek, A., Syryło, T., Zieliński, H., Niemczyk, S.: Testosterone Replacement Therapy in Chronic Kidney Disease Patients. Nutrients **14**(16) (2022). doi:10.3390/nu14163444

1035. Słowínska-Srzednicka, J., Zgliczyński, S., Ciświcka-Sznajderman, M., Srzednicki, M., Soszyński, P., Biernacka, M., Woroszyłska, M., Ruzyłło, W., Sadowski, Z.: Decreased plasma dehydroepiandrosterone sulfate and dihydrotestosterone concentrations in young men after myocardial infarction. Atherosclerosis **79**(2-3), 197-203 (1989). doi:10.1016/0021-9150(89)90124-x

1036. Smals, A.E., Pieters, G.F., Smals, A.G., Benraad, T.J., Van Laarhoven, J., Kloppenborg, P.W.: Sex difference in human growth hormone (GH) response to intravenous human pancreatic GH-releasing hormone administration in young adults. J Clin Endocrinol Metab **62**(2), 336-341 (1986). doi:10.1210/jcem-62-2-336

1037. Smals, A.G., Kloppenborg, P.W., Benraad, T.J.: Circannual cycle in plasma testosterone levels in man. The Journal of clinical endocrinology and metabolism **42**(5), 979-982 (1976). doi:10.1210/jcem-42-5-979

1038. Smals, A.G., Kloppenborg, P.W., Lequin, R.M., Benraad, T.J.: The effect of oestrogen administration on plasma testosterone, FSH and LH levels in patients with Klinefelter's syndrome and normal men. Acta Endocrinol (Copenh) **77**(4), 765-783 (1974). doi:10.1530/acta.0.0770765

1039. Smals, A.G., Kloppenborg, P.W., Pieters, G.F., Losekoot, D.C., Benraad, T.J.: Basal and human chorionic gonadotropin-stimulated 17 alpha-hydroxyprogesterone and testosterone levels in Klinefelter's syndrome. The Journal of clinical endocrinology and metabolism **47**(5), 1144-1147 (1978). doi:10.1210/jcem-47-5-1144

1040. Smals, A.G., Pieters, G.F., Boers, G.H., Raemakers, J.M., Hermus, A.R., Benraad, T.J., Kloppenborg, P.W.: Differential effect of single high dose and divided small dose administration of human chorionic gonadotropin on Leydig cell steroidogenic desensitization. J Clin Endocrinol Metab **58**(2), 327-331 (1984). doi:10.1210/jcem-58-2-327

1041. Smals, A.G., Pieters, G.F., Lozekoot, D.C., Benraad, T.J., Kloppenborg, P.W.: Dissociated responses of plasma testosterone and 17-hydroxyprogesterone to single or repeated human chorionic gonadotropin administration in normal men. J Clin Endocrinol Metab **50**(1), 190-193 (1980). doi:10.1210/jcem-50-1-190

1042. Smathers, A.M., Bemben, M.G., Bemben, D.A.: Bone density comparisons in male competitive road cyclists and untrained controls. Med Sci Sports Exerc **41**(2), 290-296 (2009). doi:10.1249/MSS.0b013e318185493e

1043. Smith, R., Rutherford, O.M.: Spine and total body bone mineral density and serum testosterone levels in male athletes. Eur J Appl Physiol Occup Physiol **67**(4), 330-334 (1993). doi:10.1007/bf00357631

1044. Snyder, C.N., Clark, R.V., Caricofe, R.B., Bush, M.A., Roth, M.Y., Page, S.T., Bremner, W.J., Amory, J.K.: Pharmacokinetics of 2 novel formulations of modified-release oral testosterone alone and with finasteride in normal men with experimental hypogonadism. J Androl **31**(6), 527-535 (2010). doi:10.2164/jandrol.109.009746

1045. Snyder, P.J., Utiger, R.D.: Response to thyrotropin releasing hormone (TRH) in normal man. J Clin Endocrinol Metab **34**(2), 380-385 (1972). doi:10.1210/jcem-34-2-380

1046. Sokol, R.Z., Palacios, A., Campfield, L.A., Saul, C., Swerdloff, R.S.: Comparison of the kinetics of injectable testosterone in eugonadal and hypogonadal men. Fertil Steril **37**(3), 425-430 (1982). doi:10.1016/s0015-0282(16)46108-x

1047. Soriguer, F., Rubio-MartÃ­n, E., FernÃ¡ndez, D., ValdÃ©s, S., GarcÃ­a-Escobar, E., MartÃ­n-NÃºÃ±ez, G.M., Esteva, I., Almaraz, M.C., Rojo-MartÃ­nez, G.: Testosterone, SHBG and risk of type 2 diabetes in the second evaluation of the Pizarra cohort study. Eur J Clin Invest **42**(1), 79-85 (2012). doi:10.1111/j.1365-2362.2011.02559.x

1048. Soriguer, F., Rubio-Martín, E., Fernández, D., Valdés, S., García-Escobar, E., Martín-Núñez, G.M., Esteva, I., Almaraz, M.C., Rojo-Martínez, G.: Testosterone, SHBG and risk of type 2 diabetes in the second evaluation of the Pizarra cohort study. Eur J Clin Invest **42**(1), 79-85 (2012). doi:10.1111/j.1365-2362.2011.02559.x

1049. Soyupek, F., Soyupek, S., Perk, H., Ozorak, A.: Androgen deprivation therapy for prostate cancer: effects on hand function. Urol Oncol **26**(2), 141-146 (2008). doi:10.1016/j.urolonc.2006.12.014

1050. Spector, T.D., Ollier, W., Perry, L.A., Silman, A.J., Thompson, P.W., Edwards, A.: Free and serum testosterone levels in 276 males: a comparative study of rheumatoid arthritis, ankylosing spondylitis and healthy controls. Clin Rheumatol **8**(1), 37-41 (1989). doi:10.1007/bf02031066

1051. Spetz Holm, A.C., Fredrikson, M.G., Hammar, M.L.: Symptoms of testosterone deficiency in early middle aged men. Aging Male **15**(2), 78-84 (2012). doi:10.3109/13685538.2012.669435

1052. Spitz, I.M., Lavie, P., Calderon, N., Gordon, C.R., Oksenberg, A., Ron, M., Laufer, N., Livshin, Y., Schenker, J.: Dissociation between sleep-related and TRH-induced prolactin secretion in seminiferous tubule failure. Metabolism **31**(1), 10-13 (1982).

1053. Spitz, I.M., Margalioth, E.J., Yeger, Y., Livshin, Y., Zylber-Haran, E., Shilo, S.: Effect of non aromatizable androgens on LHRH and TRH responses in primary testicular failure. Horm Metab Res **16**(9), 492-497 (1984). doi:10.1055/s-2007-1014827

1054. Spratt, D.I., O'Dea, L.S., Schoenfeld, D., Butler, J., Rao, P.N., Crowley, W.F., Jr.: Neuroendocrine-gonadal axis in men: frequent sampling of LH, FSH, and testosterone. Am J Physiol **254**(5 Pt 1), E658-666 (1988). doi:10.1152/ajpendo.1988.254.5.E658

1055. Srinath, R., Gottesman, R.F., Hill Golden, S., Carson, K.A., Dobs, A.: Association Between Endogenous Testosterone and Cerebrovascular Disease in the ARIC Study (Atherosclerosis Risk in Communities). Stroke **47**(11), 2682-2688 (2016). doi:10.1161/strokeaha.116.014088

1056. Stahl, F., Schnorr, D., Pilz, C., Dörner, G.: Dehydroepiandrosterone (DHEA) levels in patients with prostatic cancer, heart diseases and under surgery stress. Exp Clin Endocrinol **99**(2), 68-70 (1992). doi:10.1055/s-0029-1211136

1057. Stefanick, M.L., Williams, P.T., Krauss, R.M., Terry, R.B., Vranizan, K.M., Wood, P.D.: Relationships of plasma estradiol, testosterone, and sex hormone-binding globulin with lipoproteins, apolipoproteins, and high density lipoprotein subfractions in men. J Clin Endocrinol Metab **64**(4), 723-729 (1987). doi:10.1210/jcem-64-4-723

1058. Stenholm, S., Metter, E.J., Roth, G.S., Ingram, D.K., Mattison, J.A., Taub, D.D., Ferrucci, L.: Relationship between plasma ghrelin, insulin, leptin, interleukin 6, adiponectin, testosterone and longevity in the Baltimore Longitudinal Study of Aging. Aging Clin Exp Res **23**(2), 153-158 (2011). doi:10.1007/bf03351078

1059. Stewart, T.M., Liu, D.Y., Garrett, C., JÃ¸rgensen, N., Brown, E.H., Baker, H.W.: Associations between andrological measures, hormones and semen quality in fertile Australian men: inverse relationship between obesity and sperm output. Hum Reprod **24**(7), 1561-1568 (2009). doi:10.1093/humrep/dep075

1060. Stewart, T.M., Liu, D.Y., Garrett, C., Jørgensen, N., Brown, E.H., Baker, H.W.: Associations between andrological measures, hormones and semen quality in fertile Australian men: inverse relationship between obesity and sperm output. Hum Reprod **24**(7), 1561-1568 (2009). doi:10.1093/humrep/dep075

1061. Strickland, A.L., Apland, M.: Studies of serum testosterone and its reduction products. South Med J **70**(4), 426-428 (1977). doi:10.1097/00007611-197704000-00017

1062. Stuckey, B.G., Walsh, J.P., Ching, H.L., Stuckey, A.W., Palmer, N.R., Thompson, P.L., Watts, G.F.: Erectile dysfunction predicts generalised cardiovascular disease: evidence from a case-control study. Atherosclerosis **194**(2), 458-464 (2007). doi:10.1016/j.atherosclerosis.2006.08.043

1063. Su, L., Chen, J., Qu, H., Luo, C., Wu, J., Jiao, Y.: Association between snoring frequency and male serum testosterone: Findings from the 2015-2016 National Health and Nutrition Examination Survey. Sleep Med **100**, 1-5 (2022). doi:10.7150/ijms.73645

10.1016/j.sleep.2022.07.016

1064. Subramanya, V., Ambale-Venkatesh, B., Ohyama, Y., Zhao, D., Nwabuo, C.C., Post, W.S., Guallar, E., Ouyang, P., Shah, S.J., Allison, M.A., Ndumele, C.E., Vaidya, D., Bluemke, D.A., Lima, J.A., Michos, E.D.: Relation of Sex Hormone Levels With Prevalent and 10-Year Change in Aortic Distensibility Assessed by MRI: The Multi-Ethnic Study of Atherosclerosis. Am J Hypertens **31**(7), 774-783 (2018). doi:10.1093/ajh/hpy024

1065. Subramanya, V., Zhao, D., Ouyang, P., Lima, J.A., Vaidya, D., Ndumele, C.E., Bluemke, D.A., Shah, S.J., Guallar, E., Nwabuo, C.C., Allison, M.A., Heckbert, S.R., Post, W.S., Michos, E.D.: Sex hormone levels and change in left ventricular structure among men and post-menopausal women: The Multi-Ethnic Study of Atherosclerosis (MESA). Maturitas **108**, 37-44 (2018). doi:10.1016/j.maturitas.2017.11.006

1066. Sulcová, J., Hill, M., Masek, Z., Ceska, R., Novácek, A., Hampl, R., Stárka, L.: Effects of transdermal application of 7-oxo-DHEA on the levels of steroid hormones, gonadotropins and lipids in healthy men. Physiol Res **50**(1), 9-18 (2001).

1067. SulcovÃ¡, J., Hill, M., Masek, Z., Ceska, R., NovÃ¡cek, A., Hampl, R., StÃ¡rka, L.: Effects of transdermal application of 7-oxo-DHEA on the levels of steroid hormones, gonadotropins and lipids in healthy men. Physiol Res **50**(1), 9-18 (2001).

1068. Sullivan, D.H., Roberson, P.K., Johnson, L.E., Bishara, O., Evans, W.J., Smith, E.S., Price, J.A.: Effects of muscle strength training and testosterone in frail elderly males. Med Sci Sports Exerc **37**(10), 1664-1672 (2005). doi:10.1249/01.mss.0000181840.54860.8b

1069. Sun, A.J., Jing, T., Heymsfield, S.B., Phillips, G.B.: Relationship of leptin and sex hormones to bone mineral density in men. Acta Diabetol **40 Suppl 1**, S101-105 (2003). doi:10.1007/s00592-003-0039-5

1070. Sun, W., Bi, L.K., Xie, D.D., Yu, D.X.: Serum nesfatin-1 is associated with testosterone and the severity of erectile dysfunction. Andrologia **52**(7), e13634 (2020). doi:10.6026/97320630016176

10.1111/and.13634

1071. Susheela, A.K., Jethanandani, P.: Circulating testosterone levels in skeletal fluorosis patients. J Toxicol Clin Toxicol **34**(2), 183-189 (1996). doi:10.3109/15563659609013768

1072. Svartberg, J., Midtby, M., BÃ¸naa, K.H., Sundsfjord, J., Joakimsen, R.M., Jorde, R.: The associations of age, lifestyle factors and chronic disease with testosterone in men: the TromsÃ¸ Study. Eur J Endocrinol **149**(2), 145-152 (2003). doi:10.1530/eje.0.1490145

1073. Svartberg, J., Midtby, M., Bønaa, K.H., Sundsfjord, J., Joakimsen, R.M., Jorde, R.: The associations of age, lifestyle factors and chronic disease with testosterone in men: the Tromsø Study. Eur J Endocrinol **149**(2), 145-152 (2003). doi:10.1530/eje.0.1490145

1074. Svartberg, J., Schirmer, H., Wilsgaard, T., Mathiesen, E.B., NjÃ¸lstad, I., LÃ¸chen, M.L., Jorde, R.: Single-nucleotide polymorphism, rs1799941 in the Sex Hormone-Binding Globulin (SHBG) gene, related to both serum testosterone and SHBG levels and the risk of myocardial infarction, type 2 diabetes, cancer and mortality in men: the TromsÃ¸ Study. Andrology **2**(2), 212-218 (2014). doi:10.1111/j.2047-2927.2013.00174.x

1075. Svartberg, J., Schirmer, H., Wilsgaard, T., Mathiesen, E.B., Njølstad, I., Løchen, M.L., Jorde, R.: Single-nucleotide polymorphism, rs1799941 in the Sex Hormone-Binding Globulin (SHBG) gene, related to both serum testosterone and SHBG levels and the risk of myocardial infarction, type 2 diabetes, cancer and mortality in men: the Tromsø Study. Andrology **2**(2), 212-218 (2014). doi:10.1111/j.2047-2927.2013.00174.x

1076. Svartberg, J., von MÃ¼hlen, D., Mathiesen, E., Joakimsen, O., BÃ¸naa, K.H., Stensland-Bugge, E.: Low testosterone levels are associated with carotid atherosclerosis in men. J Intern Med **259**(6), 576-582 (2006). doi:10.1111/j.1365-2796.2006.01637.x

1077. Svartberg, J., von Mühlen, D., Mathiesen, E., Joakimsen, O., Bønaa, K.H., Stensland-Bugge, E.: Low testosterone levels are associated with carotid atherosclerosis in men. J Intern Med **259**(6), 576-582 (2006). doi:10.1111/j.1365-2796.2006.01637.x

1078. Swiecicka, A., Lunt, M., Ahern, T., O'Neill, T.W., Bartfai, G., Casanueva, F.F., Forti, G., Giwercman, A., Han, T.S., Lean, M.E.J., Pendleton, N., Punab, M., Slowikowska-Hilczer, J., Vanderschueren, D., Huhtaniemi, I.T., Wu, F.C.W., Rutter, M.K.: Nonandrogenic Anabolic Hormones Predict Risk of Frailty: European Male Ageing Study Prospective Data. J Clin Endocrinol Metab **102**(8), 2798-2806 (2017). doi:10.1210/jc.2017-00090

1079. Szulc, P., Amri, E.Z., Varennes, A., Panaia-Ferrari, P., Fontas, E., Goudable, J., Chapurlat, R., Breuil, V.: High serum oxytocin is associated with metabolic syndrome in older men - The MINOS study. Diabetes Res Clin Pract **122**, 17-27 (2016). doi:10.1016/j.diabres.2016.09.022

1080. Szulc, P., Claustrat, B., Munoz, F., Marchand, F., Delmas, P.D.: Assessment of the role of 17beta-oestradiol in bone metabolism in men: does the assay technique matter? The MINOS study. Clin Endocrinol (Oxf) **61**(4), 447-457 (2004). doi:10.1111/j.1365-2265.2004.02117.x

1081. Szulc, P., Duboeuf, F., Marchand, F., Delmas, P.D.: Hormonal and lifestyle determinants of appendicular skeletal muscle mass in men: the MINOS study. Am J Clin Nutr **80**(2), 496-503 (2004). doi:10.1093/ajcn/80.2.496

1082. Szulc, P., Feyt, C., Chapurlat, R.: High risk of fall, poor physical function, and low grip strength in men with fracture-the STRAMBO study. J Cachexia Sarcopenia Muscle **7**(3), 299-311 (2016). doi:10.1002/jcsm.12066

1083. Szulc, P., Hofbauer, L.C., Heufelder, A.E., Roth, S., Delmas, P.D.: Osteoprotegerin serum levels in men: correlation with age, estrogen, and testosterone status. J Clin Endocrinol Metab **86**(7), 3162-3165 (2001). doi:10.1210/jcem.86.7.7657

1084. Szulc, P., Schoppet, M., Goettsch, C., Rauner, M., Dschietzig, T., Chapurlat, R., Hofbauer, L.C.: Endocrine and clinical correlates of myostatin serum concentration in men--the STRAMBO study. J Clin Endocrinol Metab **97**(10), 3700-3708 (2012). doi:10.1210/jc.2012-1273

1085. Szymczak, J., Bohdanowicz-Pawlak, A., Bednarek-Tupikowska, G., BidziÅ„ska, B., Dunajska, K.: [Thyroid function in organic lesions of the hypothalamo-pituitary area]. Wiad Lek **58**(5-6), 299-302 (2005).

1086. Szymczak, J., Bohdanowicz-Pawlak, A., Bednarek-Tupikowska, G., Bidzińska, B., Dunajska, K.: [Thyroid function in organic lesions of the hypothalamo-pituitary area]. Wiad Lek **58**(5-6), 299-302 (2005).

1087. Szyska-Skrobot, D., Marchlewska, K., Walczak-Jędrzejowska, R., Oszukowska, E., Filipiak, E., Kula, P., Mężyk, R., Kowalska, A., Jaszewski, R., Słowikowska-Hilczer, J., Kula, K.: Free and bioavailable fractions of sex steroids may influence bones in young men, depending on age and oestradiol level. Endokrynol Pol **65**(5), 357-364 (2014). doi:10.5603/ep.2014.0049

1088. Tagesen, E.C., Judge, L.W., Bellar, D.M.: A Comparison of Continuous and Interval Exercise on Cognition in Young Adults. Int J Exerc Sci **16**(5), 458-468 (2023).

1089. Tagesen, E.C., Judge, L.W., Bellar, D.M., Lillo, P., Zitko, P., Godoy-Reyes, G., Asenjo, G., Sáez, D., Cea, G., Navarrete, P., Valenzuela, D., Hughes, R., Heverin, M., Logroscino, G., Hardiman, O.: A Comparison of Continuous and Interval Exercise on Cognition in Young Adults

Incidence of amyotrophic lateral sclerosis in Chile. Int J Exerc Sci **16**(5), 458-468 (2023). doi:10.1080/21678421.2024.2329706

1090. Tahani, N., Nieddu, L., Prossomariti, G., Spaziani, M., Granato, S., Carlomagno, F., Anzuini, A., Lenzi, A., Radicioni, A.F., Romagnoli, E.: Long-term effect of testosterone replacement therapy on bone in hypogonadal men with Klinefelter Syndrome. Endocrine **61**(2), 327-335 (2018). doi:10.1007/s12020-018-1604-6

1091. Tahara, N., Imaizumi, T., Takeuchi, M., Yamagishi, S.: Insulin resistance is an independent correlate of high serum levels of advanced glycation end products (AGEs) and low testosterone in non-diabetic men. Oxid Med Cell Longev **3**(4), 262-265 (2010). doi:10.4161/oxim.3.4.12734

1092. Tajar, A., McBeth, J., Lee, D.M., Macfarlane, G.J., Huhtaniemi, I.T., Finn, J.D., Bartfai, G., Boonen, S., Casanueva, F.F., Forti, G., Giwercman, A., Han, T.S., Kula, K., Labrie, F., Lean, M.E.J., Pendleton, N., Punab, M., Silman, A.J., Vanderschueren, D., O'Neill, T.W., Wu, F.C.W.: Elevated levels of gonadotrophins but not sex steroids are associated with musculoskeletal pain in middle-aged and older European men. Pain **152**(7), 1495-1501 (2011). doi:10.1016/j.pain.2011.01.048

1093. Tajar, A., O'Connell, M.D., Mitnitski, A.B., O'Neill, T.W., Searle, S.D., Huhtaniemi, I.T., Finn, J.D., Bartfai, G., Boonen, S., Casanueva, F.F., Forti, G., Giwercman, A., Han, T.S., Kula, K., Labrie, F., Lean, M.E., Pendleton, N., Punab, M., Silman, A.J., Vanderschueren, D., Rockwood, K., Wu, F.C.: Frailty in relation to variations in hormone levels of the hypothalamic-pituitary-testicular axis in older men: results from the European male aging study. J Am Geriatr Soc **59**(5), 814-821 (2011). doi:10.1111/j.1532-5415.2011.03398.x

1094. Takahashi, P.Y., Liu, P.Y., Veldhuis, J.D.: Distinct roles of age and abdominal visceral fat in reducing androgen receptor-dependent negative feedback on LH secretion in healthy men. Andrology **2**(4), 588-595 (2014). doi:10.1111/j.2047-2927.2014.00218.x

1095. Takahashi, P.Y., Votruba, P., Abu-Rub, M., Mielke, K., Veldhuis, J.D.: Age attenuates testosterone secretion driven by amplitude-varying pulses of recombinant human luteinizing hormone during acute gonadotrope inhibition in healthy men. The Journal of clinical endocrinology and metabolism **92**(9), 3626-3632 (2007). doi:10.1210/jc.2006-2704

1096. Takeuchi, T., Tsutsumi, O.: Serum bisphenol a concentrations showed gender differences, possibly linked to androgen levels. Biochem Biophys Res Commun **291**(1), 76-78 (2002). doi:10.1006/bbrc.2002.6407

1097. Tamimi, R., Mucci, L.A., Spanos, E., Lagiou, A., Benetou, V., Trichopoulos, D.: Testosterone and oestradiol in relation to tobacco smoking, body mass index, energy consumption and nutrient intake among adult men. Eur J Cancer Prev **10**(3), 275-280 (2001). doi:10.1097/00008469-200106000-00012

1098. Tan, U.: Serum testosterone levels in male and female subjects with standard and anomalous dominance. Int J Neurosci **58**(3-4), 211-214 (1991). doi:10.3109/00207459108985436

1099. Tanabe, M., Akehi, Y., Nomiyama, T., Murakami, J., Yanase, T.: Total testosterone is the most valuable indicator of metabolic syndrome among various testosterone values in middle-aged Japanese men. Endocr J **62**(2), 123-132 (2015). doi:10.1507/endocrj.EJ14-0313

1100. Tanner, A.V., Nielsen, B.V., Allgrove, J.: Salivary and plasma cortisol and testosterone responses to interval and tempo runs and a bodyweight-only circuit session in endurance-trained men. J Sports Sci **32**(7), 680-689 (2014). doi:10.1080/02640414.2013.850594

1101. Taxel, P., Kennedy, D.G., Fall, P.M., Willard, A.K., Clive, J.M., Raisz, L.G.: The effect of aromatase inhibition on sex steroids, gonadotropins, and markers of bone turnover in older men. J Clin Endocrinol Metab **86**(6), 2869-2874 (2001). doi:10.1210/jcem.86.6.7541

1102. Taylor, A.P., Lee, H., Webb, M.L., Joffe, H., Finkelstein, J.S.: Effects of Testosterone and Estradiol Deficiency on Vasomotor Symptoms in Hypogonadal Men. J Clin Endocrinol Metab **101**(9), 3479-3486 (2016). doi:10.1210/jc.2016-1612

1103. Tchernof, A., DesprÃ©s, J.P., Dupont, A., BÃ©langer, A., Nadeau, A., Prud'homme, D., Moorjani, S., Lupien, P.J., Labrie, F.: Relation of steroid hormones to glucose tolerance and plasma insulin levels in men. Importance of visceral adipose tissue. Diabetes Care **18**(3), 292-299 (1995). doi:10.2337/diacare.18.3.292

1104. Tchernof, A., Després, J.P., Dupont, A., Bélanger, A., Nadeau, A., Prud'homme, D., Moorjani, S., Lupien, P.J., Labrie, F.: Relation of steroid hormones to glucose tolerance and plasma insulin levels in men. Importance of visceral adipose tissue. Diabetes Care **18**(3), 292-299 (1995). doi:10.2337/diacare.18.3.292

1105. Tchernof, A., Labrie, F., BÃ©langer, A., Prud'homme, D., Bouchard, C., Tremblay, A., Nadeau, A., DesprÃ©s, J.P.: Relationships between endogenous steroid hormone, sex hormone-binding globulin and lipoprotein levels in men: contribution of visceral obesity, insulin levels and other metabolic variables. Atherosclerosis **133**(2), 235-244 (1997). doi:10.1016/s0021-9150(97)00125-1

1106. Tchernof, A., Labrie, F., Bélanger, A., Prud'homme, D., Bouchard, C., Tremblay, A., Nadeau, A., Després, J.P.: Relationships between endogenous steroid hormone, sex hormone-binding globulin and lipoprotein levels in men: contribution of visceral obesity, insulin levels and other metabolic variables. Atherosclerosis **133**(2), 235-244 (1997). doi:10.1016/s0021-9150(97)00125-1

1107. Tegelman, R., Johansson, C., Hemmingsson, P., Eklöf, R., Carlström, K., Pousette, A.: Endogenous anabolic and catabolic steroid hormones in male and female athletes during off season. Int J Sports Med **11**(2), 103-106 (1990). doi:10.1055/s-2007-1024771

1108. Tegelman, R., Lindeskog, P., Carlström, K., Pousette, A., Blomstrand, R.: Peripheral hormone levels in healthy subjects during controlled fasting. Acta Endocrinol (Copenh) **113**(3), 457-462 (1986). doi:10.1530/acta.0.1130457

1109. Tenover, J.S., Matsumoto, A.M., Clifton, D.K., Bremner, W.J.: Age-related alterations in the circadian rhythms of pulsatile luteinizing hormone and testosterone secretion in healthy men. J Gerontol **43**(6), M163-169 (1988). doi:10.1093/geronj/43.6.m163

1110. Thirumalai, A., Cooper, L.A., Rubinow, K.B., Amory, J.K., Lin, D.W., Wright, J.L., Marck, B.T., Matsumoto, A.M., Page, S.T.: Stable Intraprostatic Dihydrotestosterone in Healthy Medically Castrate Men Treated With Exogenous Testosterone. J Clin Endocrinol Metab **101**(7), 2937-2944 (2016). doi:10.1210/jc.2016-1483

1111. Tidd, M.J., Horth, C.E., Ramsay, L.E., Shelton, J.R., Palmer, R.F.: Endocrine effects of spironolactone in man. Clin Endocrinol (Oxf) **9**(5), 389-399 (1978). doi:10.1111/j.1365-2265.1978.tb03578.x

1112. Tikkanen, H.O., HÃ¤mÃ¤lÃ¤inen, E., Sarna, S., Adlercreutz, H., HÃ¤rkÃ¶nen, M.: Associations between skeletal muscle properties, physical fitness, physical activity and coronary heart disease risk factors in men. Atherosclerosis **137**(2), 377-389 (1998). doi:10.1016/s0021-9150(97)00276-1

1113. Tikkanen, H.O., Hämäläinen, E., Sarna, S., Adlercreutz, H., Härkönen, M.: Associations between skeletal muscle properties, physical fitness, physical activity and coronary heart disease risk factors in men. Atherosclerosis **137**(2), 377-389 (1998). doi:10.1016/s0021-9150(97)00276-1

1114. Tissandier, O., PÃ©res, G., Fiet, J., Piette, F.: Testosterone, dehydroepiandrosterone, insulin-like growth factor 1, and insulin in sedentary and physically trained aged men. Eur J Appl Physiol **85**(1-2), 177-184 (2001). doi:10.1007/s004210100420

1115. Tissandier, O., Péres, G., Fiet, J., Piette, F.: Testosterone, dehydroepiandrosterone, insulin-like growth factor 1, and insulin in sedentary and physically trained aged men. Eur J Appl Physiol **85**(1-2), 177-184 (2001). doi:10.1007/s004210100420

1116. Tochimoto, S., Olivo, J., Southren, A.L., Gordon, G.G.: Studies of plasma beta-globulin: sex difference and effect of ethinyl estradiol and testosterone. Proc Soc Exp Biol Med **134**(3), 700-702 (1970). doi:10.3181/00379727-134-34864

1117. Tomaszewski, M., Charchar, F.J., Maric, C., Kuzniewicz, R., Gola, M., Grzeszczak, W., Samani, N.J., Zukowska-Szczechowska, E.: Association between lipid profile and circulating concentrations of estrogens in young men. Atherosclerosis **203**(1), 257-262 (2009). doi:10.1016/j.atherosclerosis.2008.06.002

1118. Tomaszewski, M., Charchar, F.J., Maric, C., Kuzniewicz, R., Gola, M., Grzeszczak, W., Samani, N.J., Zukowska-Szczechowska, E.: Inverse associations between androgens and renal function: the Young Men Cardiovascular Association (YMCA) study. Am J Hypertens **22**(1), 100-105 (2009). doi:10.1038/ajh.2008.307

1119. Toone, B.K., Edeh, J., Nanjee, M.N., Wheeler, M.: Hyposexuality and epilepsy: a community survey of hormonal and behavioural changes in male epileptics. Psychol Med **19**(4), 937-943 (1989). doi:10.1017/s0033291700005651

1120. Tourney, G., Hatfield, L.: Plasma androgens in male schizophrenics. Arch Gen Psychiatry **27**(6), 753-755 (1972). doi:10.1001/archpsyc.1972.01750300025004

1121. Trabado, S., Maione, L., Bry-Gauillard, H., Affres, H., Salenave, S., Sarfati, J., Bouvattier, C., Delemer, B., Chanson, P., Le Bouc, Y., Brailly-Tabard, S., Young, J.: Insulin-like peptide 3 (INSL3) in men with congenital hypogonadotropic hypogonadism/Kallmann syndrome and effects of different modalities of hormonal treatment: a single-center study of 281 patients. J Clin Endocrinol Metab **99**(2), E268-275 (2014). doi:10.1210/jc.2013-2288

1122. Trabert, B., Graubard, B.I., Nyante, S.J., Rifai, N., Bradwin, G., Platz, E.A., McQuillan, G.M., McGlynn, K.A.: Relationship of sex steroid hormones with body size and with body composition measured by dual-energy X-ray absorptiometry in US men. Cancer Causes Control **23**(12), 1881-1891 (2012). doi:10.1007/s10552-012-0024-9

1123. Trachtenberg, J.: The effects of ketoconazole on testosterone production and normal and malignant androgen dependent tissues of the adult rat. J Urol **132**(3), 599-601 (1984). doi:10.1016/s0022-5347(17)49756-4

1124. Travison, T.G., Araujo, A.B., Beck, T.J., Williams, R.E., Clark, R.V., Leder, B.Z., McKinlay, J.B.: Relation between serum testosterone, serum estradiol, sex hormone-binding globulin, and geometrical measures of adult male proximal femur strength. J Clin Endocrinol Metab **94**(3), 853-860 (2009). doi:10.1210/jc.2008-0668

1125. Tremblay, M.S., Copeland, J.L., Van Helder, W.: Influence of exercise duration on post-exercise steroid hormone responses in trained males. European journal of applied physiology **94**(5-6), 505-513 (2005). doi:10.1007/s00421-005-1380-x

1126. Tremblay, R.R., Beitins, I.Z., Kowarski, A., Migeon, C.J.: Measurement of plasma dihydrotestosterone by competitive protein-binding analysis. Steroids **16**(1), 29-40 (1970). doi:10.1016/s0039-128x(70)80093-9

1127. Tremellen, K., McPhee, N., Pearce, K., Benson, S., Schedlowski, M., Engler, H.: Endotoxin-initiated inflammation reduces testosterone production in men of reproductive age. Am J Physiol Endocrinol Metab **314**(3), E206-e213 (2018). doi:10.1152/ajpendo.00279.2017

1128. Tripodianakis, J., Markianos, M., Rouvali, O., Istikoglou, C.: Gonadal axis hormones in psychiatric male patients after a suicide attempt. Eur Arch Psychiatry Clin Neurosci **257**(3), 135-139 (2007). doi:10.1007/s00406-006-0686-y

1129. Tsai, E.C., Boyko, E.J., Leonetti, D.L., Fujimoto, W.Y.: Low serum testosterone level as a predictor of increased visceral fat in Japanese-American men. Int J Obes Relat Metab Disord **24**(4), 485-491 (2000). doi:10.1038/sj.ijo.0801183

1130. Tsai, L., Johansson, C., Pousette, A., Tegelman, R., Carlström, K., Hemmingsson, P.: Cortisol and androgen concentrations in female and male elite endurance athletes in relation to physical activity. Eur J Appl Physiol Occup Physiol **63**(3-4), 308-311 (1991). doi:10.1007/bf00233867

1131. Tsai, L., Karpakka, J., Aginger, C., Johansson, C., Pousette, A., CarlstrÃ¶m, K.: Basal concentrations of anabolic and catabolic hormones in relation to endurance exercise after short-term changes in diet. Eur J Appl Physiol Occup Physiol **66**(4), 304-308 (1993). doi:10.1007/bf00237773

1132. Tsai, L., Karpakka, J., Aginger, C., Johansson, C., Pousette, A., Carlström, K.: Basal concentrations of anabolic and catabolic hormones in relation to endurance exercise after short-term changes in diet. Eur J Appl Physiol Occup Physiol **66**(4), 304-308 (1993). doi:10.1007/bf00237773

1133. Tsatsoulis, A., Shalet, S.M., Robertson, W.R., Morris, I.D., Burger, H.G., De Kretser, D.M.: Plasma inhibin levels in men with chemotherapy-induced severe damage to the seminiferous epithelium. Clin Endocrinol (Oxf) **29**(6), 659-665 (1988). doi:10.1111/j.1365-2265.1988.tb03714.x

1134. Tse, J.Y., Yeung, W.S., Ng, E.H., Cheng, L.N., Zhu, H.B., Teng, X.M., Liu, Y.K., Ho, P.C.: A comparative study of Y chromosome microdeletions in infertile males from two Chinese populations. J Assist Reprod Genet **19**(8), 376-383 (2002). doi:10.1023/a:1016346421177

1135. Tuck, S.P., Scane, A.C., Fraser, W.D., Diver, M.J., Eastell, R., Francis, R.M.: Sex steroids and bone turnover markers in men with symptomatic vertebral fractures. Bone **43**(6), 999-1005 (2008). doi:10.1016/j.bone.2008.08.123

1136. Tyler, J.P., Richardson, D.W., Newton, J.R.: The hormonal and immunological status of vasectomised men. Contraception **19**(6), 599-611 (1979). doi:10.1016/0010-7824(79)90007-6

1137. Uihlein, A.V., Finkelstein, J.S., Lee, H., Leder, B.Z.: FSH suppression does not affect bone turnover in eugonadal men. The Journal of clinical endocrinology and metabolism **99**(7), 2510-2515 (2014). doi:10.1210/jc.2013-3246

1138. Unal, M., Aksoy, D.Y., Aydın, Y., Tanriover, M.D., Berker, D., Karakaya, J., Guler, S.: Carotid artery intima-media thickness and erectile dysfunction in patients with metabolic syndrome. Med Sci Monit **20**, 884-888 (2014). doi:10.12659/msm.889771

1139. Undén, F., Ljunggren, J.G., Beck-Friis, J., Kjellman, B.F., Wetterberg, L.: Hypothalamic-pituitary-gonadal axis in major depressive disorders. Acta Psychiatr Scand **78**(2), 138-146 (1988). doi:10.1111/j.1600-0447.1988.tb06313.x

1140. Urhausen, A., Kindermann, W.: Behaviour of testosterone, sex hormone binding globulin (SHBG), and cortisol before and after a triathlon competition. Int J Sports Med **8**(5), 305-308 (1987). doi:10.1055/s-2008-1025674

1141. Vaamonde, D., Da Silva-Grigoletto, M.E., Garcia-Manso, J.M., Barrera, N., Vaamonde-Lemos, R.: Physically active men show better semen parameters and hormone values than sedentary men. European journal of applied physiology **112**(9), 3267-3273 (2012). doi:10.1007/s00421-011-2304-6

1142. Vaara, J.P., Kalliomaa, R., Hynninen, P., Kyröläinen, H.: Physical Fitness and Hormonal Profile During an 11-Week Paratroop Training Period. J Strength Cond Res **29 Suppl 11**, S163-167 (2015). doi:10.1519/jsc.0000000000001033

1143. Vaara, J.P., Kokko, J., Isoranta, M., Kyröläinen, H.: Effects of Added Resistance Training on Physical Fitness, Body Composition, and Serum Hormone Concentrations During Eight Weeks of Special Military Training Period. J Strength Cond Res **29 Suppl 11**, S168-172 (2015). doi:10.1519/jsc.0000000000001034

1144. Vaidya, D., Dobs, A., Gapstur, S.M., Golden, S.H., Cushman, M., Liu, K., Ouyang, P.: Association of baseline sex hormone levels with baseline and longitudinal changes in waist-to-hip ratio: Multi-Ethnic Study of Atherosclerosis. Int J Obes (Lond) **36**(12), 1578-1584 (2012). doi:10.1038/ijo.2012.3

1145. Valancy, D., Blachman-Braun, R., Kuchakulla, M., Nackeeran, S., Ramasamy, R.: Association between low testosterone and anaemia: Analysis of the National Health and Nutrition Examination Survey. Andrologia **53**(11), e14210 (2021). doi:10.1007/s12020-021-02631-2

10.1111/and.14210

1146. Valenti, G., Denti, L., Maggio, M., Ceda, G., Volpato, S., Bandinelli, S., Ceresini, G., Cappola, A., Guralnik, J.M., Ferrucci, L.: Effect of DHEAS on skeletal muscle over the life span: the InCHIANTI study. J Gerontol A Biol Sci Med Sci **59**(5), 466-472 (2004). doi:10.1093/gerona/59.5.m466

1147. Valero-Politi, J., Fuentes-Arderiu, X.: Annual rhythmic variations of follitropin, lutropin, testosterone and sex-hormone-binding globulin in men. Clin Chim Acta **271**(1), 57-71 (1998). doi:10.1016/s0009-8981(97)00239-8

1148. Van de Velde, F., Reyns, T., Toye, K., Fiers, T., Kaufman, J.M., T'Sjoen, G., Lapauw, B.: The effects of age and obesity on postprandial dynamics of serum testosterone levels in men. Clin Endocrinol (Oxf) **92**(3), 214-221 (2020). doi:10.1111/cen.14141

1149. van den Beld, A., Huhtaniemi, I.T., Pettersson, K.S., Pols, H.A., Grobbee, D.E., de Jong, F.H., Lamberts, S.W.: Luteinizing hormone and different genetic variants, as indicators of frailty in healthy elderly men. J Clin Endocrinol Metab **84**(4), 1334-1339 (1999). doi:10.1210/jcem.84.4.5616

1150. Van Den Saffele, J.K., Goemaere, S., De Bacquer, D., Kaufman, J.M.: Serum leptin levels in healthy ageing men: are decreased serum testosterone and increased adiposity in elderly men the consequence of leptin deficiency? Clin Endocrinol (Oxf) **51**(1), 81-88 (1999). doi:10.1046/j.1365-2265.1999.00741.x

1151. van der Merwe, J., Brooks, N.E., Myburgh, K.H.: Three weeks of creatine monohydrate supplementation affects dihydrotestosterone to testosterone ratio in college-aged rugby players. Clin J Sport Med **19**(5), 399-404 (2009). doi:10.1097/JSM.0b013e3181b8b52f

1152. van der Spoel, E., Roelfsema, F., Jansen, S.W., Akintola, A.A., Ballieux, B.E., Cobbaert, C.M., Blauw, G.J., Slagboom, P.E., Westendorp, R.G., Pijl, H., van Heemst, D.: Familial Longevity Is Not Associated with Major Differences in the Hypothalamic-Pituitary-Gonadal Axis in Healthy Middle-Aged Men. Front Endocrinol (Lausanne) **7**, 143 (2016). doi:10.1093/humrep/dew284

1153. van Hemmen, J., Veltman, D.J., Hoekzema, E., Cohen-Kettenis, P.T., Dessens, A.B., Bakker, J.: Neural Activation During Mental Rotation in Complete Androgen Insensitivity Syndrome: The Influence of Sex Hormones and Sex Chromosomes. Cereb Cortex **26**(3), 1036-1045 (2016). doi:10.1093/cercor/bhu280

1154. Van Pottelbergh, I., Goemaere, S., Zmierczak, H., Kaufman, J.M.: Perturbed sex steroid status in men with idiopathic osteoporosis and their sons. J Clin Endocrinol Metab **89**(10), 4949-4953 (2004). doi:10.1210/jc.2003-032081

1155. Vanbillemont, G., Bogaert, V., De Bacquer, D., Lapauw, B., Goemaere, S., Toye, K., Van Steen, K., Taes, Y., Kaufman, J.M.: Polymorphisms of the SHBG gene contribute to the interindividual variation of sex steroid hormone blood levels in young, middle-aged and elderly men. Clin Endocrinol (Oxf) **70**(2), 303-310 (2009). doi:10.1111/j.1365-2265.2008.03365.x

1156. Vandenput, L., Mellström, D., Lorentzon, M., Swanson, C., Karlsson, M.K., Brandberg, J., Lönn, L., Orwoll, E., Smith, U., Labrie, F., Ljunggren, O., Tivesten, A., Ohlsson, C.: Androgens and glucuronidated androgen metabolites are associated with metabolic risk factors in men. J Clin Endocrinol Metab **92**(11), 4130-4137 (2007). doi:10.1210/jc.2007-0252

1157. Varma, B., Ogunmoroti, O., Ndumele, C.E., Kazzi, B., Rodriquez, C.P., Osibogun, O., Allison, M.A., Bertoni, A.G., Michos, E.D., Chute, C.G., Baron, J.A., Plymate, S.R., Kiel, D.P., Pavia, A.T., Lozner, E.C., O'Keefe, T., MacDonald, G.J.: Associations between endogenous sex hormone levels and adipokine levels in the Multi-Ethnic Study of Atherosclerosis

Sex hormones and coronary artery disease. Front Cardiovasc Med **9**(5), 1062460 (2022). doi:10.3389/fcvm.2022.1062460

10.1016/0002-9343(87)90642-5

1158. Vasankari, T.J., Rusko, H., Kujala, U.M., Huhtaniemi, I.T.: The effect of ski training at altitude and racing on pituitary, adrenal and testicular function in men. Eur J Appl Physiol Occup Physiol **66**(3), 221-225 (1993). doi:10.1007/bf00235097

1159. Vega, E., Ghiringhelli, G., Mautalen, C., Rey Valzacchi, G., Scaglia, H., Zylberstein, C.: Bone mineral density and bone size in men with primary osteoporosis and vertebral fractures. Calcif Tissue Int **62**(5), 465-469 (1998). doi:10.1007/s002239900462

1160. Veldhuis, J.D., Iranmanesh, A., Mulligan, T.: Age and testosterone feedback jointly control the dose-dependent actions of gonadotropin-releasing hormone in healthy men. J Clin Endocrinol Metab **90**(1), 302-309 (2005). doi:10.1210/jc.2004-1313

1161. Veldhuis, J.D., Reynolds, G.A., Iranmanesh, A., Bowers, C.Y.: Twenty-four hour continuous ghrelin infusion augments physiologically pulsatile, nycthemeral, and entropic (feedback-regulated) modes of growth hormone secretion. J Clin Endocrinol Metab **93**(9), 3597-3603 (2008). doi:10.1210/jc.2008-0620

1162. Venkat, K., Desai, M., Arora, M.M., Singh, P., Khatkhatay, M.I.: Age-related changes in sex steroid levels influence bone mineral density in healthy Indian men. Osteoporos Int **20**(6), 955-962 (2009). doi:10.1007/s00198-008-0765-1

1163. Vermeulen, A., Kaufman, J.M., Deslypere, J.P., Thomas, G.: Attenuated luteinizing hormone (LH) pulse amplitude but normal LH pulse frequency, and its relation to plasma androgens in hypogonadism of obese men. J Clin Endocrinol Metab **76**(5), 1140-1146 (1993). doi:10.1210/jcem.76.5.8496304

1164. Vigersky, R.A., Chapman, R.M., Berenberg, J., Glass, A.R.: Testicular dysfunction in untreated Hodgkin's disease. Am J Med **73**(4), 482-486 (1982). doi:10.1016/0002-9343(82)90325-4

1165. Vita, R., Capodicasa, G., Di Bari, F., Amadeo, G., Stagno D'Alcontres, F., Benvenga, S.: Biochemical features of eugonadal patients with idiopathic gynaecomastia: A retrospective cross-sectional study. Andrologia **53**(2), e13962 (2021). doi:10.1111/and.13962

1166. Vittek, J., L'Hommedieu, D.G., Gordon, G.G., Rappaport, S.C., Southren, A.L.: Direct radioimmunoassay (RIA) of salivary testosterone: correlation with free and total serum testosterone. Life Sci **37**(8), 711-716 (1985). doi:10.1016/0024-3205(85)90540-5

1167. Vlachopoulos, C., Ioakeimidis, N., Miner, M., Aggelis, A., Pietri, P., Terentes-Printzios, D., Tsekoura, D., Stefanadis, C.: Testosterone deficiency: a determinant of aortic stiffness in men. Atherosclerosis **233**(1), 278-283 (2014). doi:10.1016/j.atherosclerosis.2013.12.010

1168. Vlachopoulos, C., Pietri, P., Ioakeimidis, N., Aggelis, A., Terentes-Printzios, D., Abdelrasoul, M., Gourgouli, I., Stefanadis, C., Tousoulis, D.: Inverse association of total testosterone with central haemodynamics and left ventricular mass in hypertensive men. Atherosclerosis **250**, 57-62 (2016). doi:10.1016/j.atherosclerosis.2016.04.018

1169. Volek, J.S., Ratamess, N.A., Rubin, M.R., GÃ³mez, A.L., French, D.N., McGuigan, M.M., Scheett, T.P., Sharman, M.J., HÃ¤kkinen, K., Kraemer, W.J.: The effects of creatine supplementation on muscular performance and body composition responses to short-term resistance training overreaching. Eur J Appl Physiol **91**(5-6), 628-637 (2004). doi:10.1007/s00421-003-1031-z

1170. Volek, J.S., Ratamess, N.A., Rubin, M.R., Gómez, A.L., French, D.N., McGuigan, M.M., Scheett, T.P., Sharman, M.J., Häkkinen, K., Kraemer, W.J.: The effects of creatine supplementation on muscular performance and body composition responses to short-term resistance training overreaching. Eur J Appl Physiol **91**(5-6), 628-637 (2004). doi:10.1007/s00421-003-1031-z

1171. Volek, J.S., Sharman, M.J., Love, D.M., Avery, N.G., GÃ³mez, A.L., Scheett, T.P., Kraemer, W.J.: Body composition and hormonal responses to a carbohydrate-restricted diet. Metabolism **51**(7), 864-870 (2002). doi:10.1053/meta.2002.32037

1172. Volek, J.S., Sharman, M.J., Love, D.M., Avery, N.G., Gómez, A.L., Scheett, T.P., Kraemer, W.J.: Body composition and hormonal responses to a carbohydrate-restricted diet. Metabolism **51**(7), 864-870 (2002). doi:10.1053/meta.2002.32037

1173. von der, P.B., Sarkola, T., Seppa, K., Eriksson, C.J.: Testosterone, 5 alpha-dihydrotestosterone and cortisol in men with and without alcohol-related aggression. J Stud Alcohol **63**(5), 518-526 (2002). doi:10.15288/jsa.2002.63.518

1174. von MÃ¼hlen, D., Laughlin, G.A., Kritz-Silverstein, D., Bergstrom, J., Bettencourt, R.: Effect of dehydroepiandrosterone supplementation on bone mineral density, bone markers, and body composition in older adults: the DAWN trial. Osteoporos Int **19**(5), 699-707 (2008). doi:10.1007/s00198-007-0520-z

1175. Walsh, J.S., Paggiosi, M.A., Eastell, R.: Cortical consolidation of the radius and tibia in young men and women. J Clin Endocrinol Metab **97**(9), 3342-3348 (2012). doi:10.1210/jc.2012-1677

1176. Wang, A., Arver, S., Flanagan, J., Gyberg, V., Näsman, P., Ritsinger, V., Mellbin, L.G.: Dynamics of testosterone levels in patients with newly detected glucose abnormalities and acute myocardial infarction. Diab Vasc Dis Res **15**(6), 511-518 (2018). doi:10.1177/1479164118802543

1177. Wang, C., Catlin, D.H., Starcevic, B., Heber, D., Ambler, C., Berman, N., Lucas, G., Leung, A., Schramm, K., Lee, P.W., Hull, L., Swerdloff, R.S.: Low-fat high-fiber diet decreased serum and urine androgens in men. J Clin Endocrinol Metab **90**(6), 3550-3559 (2005). doi:10.1210/jc.2004-1530

1178. Wang, C., Chan, V., Tse, T.F., Yeung, R.T.: Effect of acute myocardial infarction on pituitary-testicular function. Clin Endocrinol (Oxf) **9**(3), 249-253 (1978). doi:10.1111/j.1365-2265.1978.tb02207.x

1179. Wang, C., Chan, V., Yeung, R.T.: The effect of heroin addiction on pituitary-testicular function. Clin Endocrinol (Oxf) **9**(5), 455-461 (1978). doi:10.1111/j.1365-2265.1978.tb03585.x

1180. Wang, C., Plymate, S., Nieschlag, E., Paulsen, C.A.: Salivary testosterone in men: further evidence of a direct correlation with free serum testosterone. J Clin Endocrinol Metab **53**(5), 1021-1024 (1981). doi:10.1210/jcem-53-5-1021

1181. Wang, C., Yeung, K.K.: Use of low-dosage oral cyproterone acetate as a male contraceptive. Contraception **21**(3), 245-272 (1980). doi:10.1016/0010-7824(80)90005-0

1182. Wang, C., Youatt, G., O'Connor, S., Dulmanis, A., Hudson, B.: A simple radioimmunoassay for plasma testosterone plus 5alpha-dihydrotestosterone. J Steroid Biochem **5**(6), 551-555 (1974). doi:10.1016/0022-4731(74)90103-4

1183. Wang, L., Chen, G., Hou, J., Wei, D., Liu, P., Nie, L., Fan, K., Wang, J., Xu, Q., Song, Y., Wang, M., Huo, W., Jing, T., Li, W., Guo, Y., Wang, C., Mao, Z.: Ambient ozone exposure combined with residential greenness in relation to serum sex hormone levels in Chinese rural adults. Environ Res **210**, 112845 (2022). doi:10.1016/j.envres.2022.112845

1184. Wang, W., Yang, X., Liang, J., Liao, M., Zhang, H., Qin, X., Mo, L., Lv, W., Mo, Z.: Cigarette smoking has a positive and independent effect on testosterone levels. Hormones (Athens) **12**(4), 567-577 (2013). doi:10.14310/horm.2002.1445

1185. Ward, K.A., Pye, S.R., Adams, J.E., Boonen, S., Vanderschueren, D., Borghs, H., Gaytant, J., Gielen, E., Bartfai, G., Casanueva, F.F., Finn, J.D., Forti, G., Giwercman, A., Han, T.S., Huhtaniemi, I.T., Kula, K., Labrie, F., Lean, M.E., Pendleton, N., Punab, M., Silman, A.J., Wu, F.C., O'Neill, T.W.: Influence of age and sex steroids on bone density and geometry in middle-aged and elderly European men. Osteoporos Int **22**(5), 1513-1523 (2011). doi:10.1007/s00198-010-1437-5

1186. Watson, J.M., Shrewsberry, A.B., Taghechian, S., Goodman, M., Pattaras, J.G., Ritenour, C.W., Ogan, K.: Serum testosterone may be associated with calcium oxalate urolithogenesis. J Endourol **24**(7), 1183-1187 (2010). doi:10.1089/end.2010.0113

1187. Watts, E.L., Fensom, G.K., Smith Byrne, K., Perez-Cornago, A., Allen, N.E., Knuppel, A., Gunter, M.J., Holmes, M.V., Martin, R.M., Murphy, N., Tsilidis, K.K., Yeap, B.B., Key, T.J., Travis, R.C.: Circulating insulin-like growth factor-I, total and free testosterone concentrations and prostate cancer risk in 200 000 men in UK Biobank. Int J Cancer **148**(9), 2274-2288 (2021). doi:10.1111/and.13912

10.1002/ijc.33416

1188. Webb, C.M., McNeill, J.G., Hayward, C.S., de Zeigler, D., Collins, P.: Effects of testosterone on coronary vasomotor regulation in men with coronary heart disease. Circulation **100**(16), 1690-1696 (1999). doi:10.1161/01.cir.100.16.1690

1189. Wehr, E., Pilz, S., Boehm, B.O., MÃ¤rz, W., Obermayer-Pietsch, B.: Association of vitamin D status with serum androgen levels in men. Clin Endocrinol (Oxf) **73**(2), 243-248 (2010). doi:10.1111/j.1365-2265.2009.03777.x

1190. Wehr, E., Pilz, S., Boehm, B.O., Marz, W., Obermayer-Pietsch, B.: Association of vitamin D status with serum androgen levels in men. Clinical endocrinology **73**(2), 243-248 (2010). doi:10.1111/j.1365-2265.2009.03777.x

1191. Weinstein, R.L., Reitz, R.E.: Pituitary-testicular responsiveness in male hypogonadotropic hypogonadism. J Clin Invest **53**(2), 408-415 (1974). doi:10.1172/jci107574

1192. Weiss, L.W., Cureton, K.J., Thompson, F.N.: Comparison of serum testosterone and androstenedione responses to weight lifting in men and women. Eur J Appl Physiol Occup Physiol **50**(3), 413-419 (1983). doi:10.1007/bf00423247

1193. Welch, L.S., Schrader, S.M., Turner, T.W., Cullen, M.R.: Effects of exposure to ethylene glycol ethers on shipyard painters: II. Male reproduction. Am J Ind Med **14**(5), 509-526 (1988). doi:10.1002/ajim.4700140503

1194. Wiciński, M., Kuźmiński, O., Kujawa, A., Słomko, W., Fajkiel-Madajczyk, A., Słupski, M., Jóźwik, A., Kubiak, K., Otto, S.W., Malinowski, B.: Does Intense Endurance Workout Have an Impact on Serum Levels of Sex Hormones in Males? Biology (Basel) **12**(4) (2023). doi:10.3390/biology12040531

1195. Williams, B.M., Horth, C.E., Palmer, R.F.: The measurement of testosterone in plasma. Clin Endocrinol (Oxf) **3**(4), 397-410 (1974). doi:10.1111/j.1365-2265.1974.tb02810.x

1196. Wilson, L.D., Truong, M.P., Barber, A.R., Aoki, T.T.: Anterior pitutiary and pitutiary-dependent target organ function in men infected with the human immunodeficiency virus. Metabolism **45**(6), 738-746 (1996). doi:10.1016/s0026-0495(96)90140-7

1197. Wishart, J.M., Need, A.G., Horowitz, M., Morris, H.A., Nordin, B.E.: Effect of age on bone density and bone turnover in men. Clin Endocrinol (Oxf) **42**(2), 141-146 (1995). doi:10.1111/j.1365-2265.1995.tb01854.x

1198. Wood, G.J.A., Tiseo, B.C., Paluello, D.V., de Martin, H., Santo, M.A., Nahas, W., Srougi, M., Cocuzza, M.: Bariatric Surgery Impact on Reproductive Hormones, Semen Analysis, and Sperm DNA Fragmentation in Men with Severe Obesity: Prospective Study. Obes Surg **30**(12), 4840-4851 (2020). doi:10.1007/s11695-020-04851-3

1199. Wu, A., Shi, Z., Martin, S., Vincent, A., Heilbronn, L., Wittert, G.: Age-related changes in estradiol and longitudinal associations with fat mass in men. PLoS One **13**(8), e0201912 (2018). doi:10.1371/journal.pone.0201912

1200. Wu, F., Chen, T., Mao, S., Jiang, H., Ding, Q., Xu, G.: Levels of estradiol and testosterone are altered in Chinese men with sexual dysfunction. Andrology **4**(5), 932-938 (2016). doi:10.1111/andr.12195

1201. Wu, F.C., Bancroft, J., Davidson, D.W., Nicol, K.: The behavioural effects of testosterone undecanoate in adult men with Klinefelter's syndrome: a controlled study. Clin Endocrinol (Oxf) **16**(5), 489-497 (1982). doi:10.1111/j.1365-2265.1982.tb02763.x

1202. Wu, F.C., Edmond, P., Raab, G., Hunter, W.M.: Endocrine assessment of the subfertile male. Clin Endocrinol (Oxf) **14**(5), 493-507 (1981). doi:10.1111/j.1365-2265.1981.tb00639.x

1203. Wu, F.C., Taylor, P.L., Sellar, R.E.: LHRH pulse frequency in normal and infertile men. J Endocrinol **123**(1), 149-158 (1989). doi:10.1677/joe.0.1230149

1204. Wu, H.T., Lee, C.H., Chen, C.J., Sun, C.K.: Penile arterial waveform analyzing system for early identification of young adults with high risk of erectile dysfunction. J Sex Med **9**(4), 1094-1105 (2012). doi:10.1111/j.1743-6109.2011.02594.x

1205. Wu, H.T., Lee, C.H., Chen, C.J., Tsai, I.T., Sun, C.K.: A simplified approach to assessing penile endothelial function in young individuals at risk of erectile dysfunction. J Androl **33**(6), 1254-1262 (2012). doi:10.2164/jandrol.112.016360

1206. Wu, W.H., Yuan, P., Zhang, S.J., Jiang, X., Wu, C., Li, Y., Liu, S.F., Liu, Q.Q., Li, J.H., Pudasaini, B., Hu, Q.H., Dupuis, J., Jing, Z.C.: Impact of Pituitary-Gonadal Axis Hormones on Pulmonary Arterial Hypertension in Men. Hypertension **72**(1), 151-158 (2018). doi:10.1161/hypertensionaha.118.10963

1207. Xing, Y., Qin, W., Li, F., Jia, X.F., Jia, J.: Associations between sex hormones and cognitive and neuropsychiatric manifestations in vascular dementia (VaD). Arch Gerontol Geriatr **56**(1), 85-90 (2013). doi:10.1016/j.archger.2012.10.003

1208. Yamacake, K.G., Cocuzza, M., Torricelli, F.C., Tiseo, B.C., Frati, R., Freire, G.C., Antunes, A.A., Srougi, M.: Impact of body mass index, age and varicocele on reproductive hormone profile from elderly men. Int Braz J Urol **42**(2), 365-372 (2016). doi:10.1590/s1677-5538.Ibju.2014.0594

1209. Yamaner, F., Atmaca, H., Bayraktaroglu, T., Aydin, M., Aydemir, S.: Gonadal function in male mountain bikers. J Strength Cond Res **25**(8), 2311-2315 (2011). doi:10.1519/JSC.0b013e3181f6b0dd

1210. Yan, B., A, J., Wang, G., Lu, H., Huang, X., Liu, Y., Zha, W., Hao, H., Zhang, Y., Liu, L., Gu, S., Huang, Q., Zheng, Y., Sun, J.: Metabolomic investigation into variation of endogenous metabolites in professional athletes subject to strength-endurance training. J Appl Physiol (1985) **106**(2), 531-538 (2009). doi:10.1152/japplphysiol.90816.2008

1211. Yan, W.J., Yu, N., Yin, T.L., Zou, Y.J., Yang, J.: A new potential risk factor in patients with erectile dysfunction and premature ejaculation: folate deficiency. Asian J Androl **16**(6), 902-906 (2014). doi:10.4103/1008-682x.135981

1212. Yang, B., Sun, H., Wan, Y., Wang, H., Qin, W., Yang, L., Zhao, H., Yuan, J., Yao, B.: Associations between testosterone, bone mineral density, vitamin D and semen quality in fertile and infertile Chinese men. Int J Androl **35**(6), 783-792 (2012). doi:10.1111/j.1365-2605.2012.01287.x

1213. Yang, X.C., Jing, T.Y., Resnick, L.M., Phillips, G.B.: Relation of hemostatic risk factors to other risk factors for coronary heart disease and to sex hormones in men. Arterioscler Thromb **13**(4), 467-471 (1993). doi:10.1161/01.atv.13.4.467

1214. Yang, Y.M., Lv, X.Y., Huang, W.D., Xu, Z.R., Wu, L.J.: Study of androgen and atherosclerosis in old-age male. J Zhejiang Univ Sci B **6**(9), 931-935 (2005). doi:10.1631/jzus.2005.B0931

1215. Yasui, T., Uemura, H., Irahara, M., Arai, M., Kojimahara, N., Okabe, R., Ishii, Y., Tashiro, S., Sato, H.: Associations of endogenous sex hormones and sex hormone-binding globulin with lipid profiles in aged Japanese men and women. Clin Chim Acta **398**(1-2), 43-47 (2008). doi:10.1016/j.cca.2008.08.006

1216. Yasui-Furukori, N., Fujii, A., Sugawara, N., Tsuchimine, S., Saito, M., Hashimoto, K., Kaneko, S.: No association between hormonal abnormality and sexual dysfunction in Japanese schizophrenia patients treated with antipsychotics. Hum Psychopharmacol **27**(1), 82-89 (2012). doi:10.1002/hup.1275

1217. Yavetz, H., Harash, B., Yogev, L., Homonnai, Z.T., Paz, G.: Fertility of men following inguinal hernia repair. Andrologia **23**(6), 443-446 (1991). doi:10.1111/j.1439-0272.1991.tb02595.x

1218. Yazici, M., Sahin, M., Bolu, E., Gok, D.E., Taslipinar, A., Tapan, S., Torun, D., Uckaya, G., Kutlu, M.: Evaluation of breast enlargement in young males and factors associated with gynecomastia and pseudogynecomastia. Ir J Med Sci **179**(4), 575-583 (2010). doi:10.1007/s11845-009-0345-1

1219. Yeap, B.B., Beilin, J., Shi, Z., Knuiman, M.W., Olynyk, J.K., Bruce, D.G., Milward, E.A.: Serum testosterone levels correlate with haemoglobin in middle-aged and older men. Intern Med J **39**(8), 532-538 (2009). doi:10.1111/j.1445-5994.2008.01789.x

1220. Yeap, B.B., Coward, R.M., Stetter, C., Kunselman, A., Trussell, J.C., Lindgren, M.C., Alvero, R.R., Casson, P., Christman, G.M., Coutifaris, C., Diamond, M.P., Hansen, K.R., Krawetz, S.A., Legro, R.S., Robinson, R.D., Smith, J.F., Steiner, A.Z., Wild, R.A., Zhang, H., Santoro, N.: Testosterone and growth hormone in older men: for better or for worse?

Fertility Related Quality of Life, Gonadal Function and Erectile Dysfunction in Male Partners of Couples with Unexplained Infertility. Expert Rev Endocrinol Metab **4**(4), 321-323 (2009). doi:10.1586/eem.09.15

10.1097/ju.0000000000000205

1221. Yeap, B.B., Hyde, Z., Almeida, O.P., Norman, P.E., Chubb, S.A., Jamrozik, K., Flicker, L., Hankey, G.J.: Lower testosterone levels predict incident stroke and transient ischemic attack in older men. J Clin Endocrinol Metab **94**(7), 2353-2359 (2009). doi:10.1210/jc.2008-2416

1222. Yeap, B.B., Marriott, R.J., Antonio, L., Bhasin, S., Dobs, A.S., Dwivedi, G., Flicker, L., Matsumoto, A.M., Ohlsson, C., Orwoll, E.S., Raj, S., Reid, C.M., Vanderschueren, D., Wittert, G.A., Wu, F.C.W., Murray, K.: Sociodemographic, lifestyle and medical influences on serum testosterone and sex hormone-binding globulin in men from UK Biobank. Clin Endocrinol (Oxf) **94**(2), 290-302 (2021). doi:10.1111/cen.14342

1223. Yeap, B.B., Marriott, R.J., Manning, L., Dwivedi, G., Hankey, G.J., Wu, F.C.W., Nicholson, J.K., Murray, K.: Higher premorbid serum testosterone predicts COVID-19-related mortality risk in men. Eur J Endocrinol **187**(1), 159-170 (2022). doi:10.1530/eje-22-0104

1224. Yeap, B.B., Wu, F.C.W.: Clinical practice update on testosterone therapy for male hypogonadism: Contrasting perspectives to optimize care. Clin Endocrinol (Oxf) **90**(1), 56-65 (2019). doi:10.1111/cen.13888

1225. Yeo, Y., Park, S.W., Lee, S.C., Song, Y.M.: The relationship between serum sex hormone and cardiac echocardiographic findings in healthy men. Sci Rep **12**(1), 12787 (2022). doi:10.1038/s41598-022-17101-6

1226. Yie, S.M., Wang, R., Zhu, Y.X., Liu, G.Y., Zheng, F.X.: Circadian variations of serum sex hormone binding globulin binding capacity in normal adult men and women. J Steroid Biochem **36**(1-2), 111-115 (1990). doi:10.1016/0022-4731(90)90120-h

1227. Yosha, S., Fay, M., Longcope, C., Braverman, L.E.: Effect of D-thyroxine on serum sex hormone binding globulin (SHBG), testosterone, and pituitary-thyroid function in euthyroid subjects. J Endocrinol Invest **7**(5), 489-494 (1984). doi:10.1007/bf03348455

1228. Yoshinaga, J., Imai, K., Shiraishi, H., Nozawa, S., Yoshiike, M., Mieno, M.N., Andersson, A.M., Iwamoto, T.: Pyrethroid insecticide exposure and reproductive hormone levels in healthy Japanese male subjects. Andrology **2**(3), 416-420 (2014). doi:10.1111/j.2047-2927.2014.00202.x

1229. Younes, A.K.: Low plasma testosterone in varicocele patients with impotence and male infertility. Arch Androl **45**(3), 187-195 (2000). doi:10.1080/01485010050193968

1230. Young, R.J., Ismail, A.H., Bradley, A., Corrigan, D.L.: Effect of prolonged exercise of serum testosterone levels in adult men. Br J Sports Med **10**(4), 230-235 (1976). doi:10.1136/bjsm.10.4.230

1231. Yucra, S., Rubio, J., Gasco, M., Gonzales, C., Steenland, K., Gonzales, G.F.: Semen quality and reproductive sex hormone levels in Peruvian pesticide sprayers. Int J Occup Environ Health **12**(4), 355-361 (2006). doi:10.1179/oeh.2006.12.4.355

1232. Yuen, F., Thirumalai, A., Pham, C., Swerdloff, R.S., Anawalt, B.D., Liu, P.Y., Amory, J.K., Bremner, W.J., Dart, C., Wu, H., Hull, L., Blithe, D.L., Long, J., Wang, C., Page, S.T.: Daily Oral Administration of the Novel Androgen 11β-MNTDC Markedly Suppresses Serum Gonadotropins in Healthy Men. J Clin Endocrinol Metab **105**(3), e835-847 (2020). doi:10.1210/clinem/dgaa032

1233. Zedan, H., El-Mekhlafi, A.W., El-Noweihi, A.M., Abd El-Azim, N.E., Mostafa, T.: Soluble Fas and gonadal hormones in infertile men with varicocele. Fertil Steril **91**(2), 420-424 (2009). doi:10.1016/j.fertnstert.2007.11.090

1234. Zeidler, A., Gelfand, R., Tamagna, E., Marrs, R., Chopp, R., Kletzky, O.: Pituitary gonadal function in diabetic male patients with and without impotence. Andrologia **14**(1), 62-68 (1982). doi:10.1111/j.1439-0272.1982.tb03096.x

1235. Zeng, Q., Zhou, B., Feng, W., Wang, Y.X., Liu, A.L., Yue, J., Li, Y.F., Lu, W.Q.: Associations of urinary metal concentrations and circulating testosterone in Chinese men. Reprod Toxicol **41**, 109-114 (2013). doi:10.1016/j.reprotox.2013.06.062

1236. Zeng, Q.S., Xu, C.L., Liu, Z.Y., Wang, H.Q., Yang, B., Xu, W.D., Jin, T.L., Wu, C.Y., Huang, G., Li, Z., Wang, B., Sun, Y.H.: Relationship between serum sex hormones levels and degree of benign prostate hyperplasia in Chinese aging men. Asian J Androl **14**(5), 773-777 (2012). doi:10.1038/aja.2012.32

1237. Zhang, M.H., Zhai, L.P., Fang, Z.Y., Li, A.N., Xiao, W., Qiu, Y.: Effect of scrotal heating on sperm quality, seminal biochemical substances, and reproductive hormones in human fertile men. J Cell Biochem **119**(12), 10228-10238 (2018). doi:10.1002/jcb.27365

1238. Zhang, N., Zhang, H., Zhang, X., Zhang, B., Wang, F., Wang, C., Zhao, M., Yu, C., Gao, L., Zhao, J., Guan, Q.: The relationship between endogenous testosterone and lipid profile in middle-aged and elderly Chinese men. Eur J Endocrinol **170**(4), 487-494 (2014). doi:10.1530/eje-13-0802

1239. Zhang, W., Piotrowska, K., Chavoshan, B., Wallace, J., Liu, P.Y.: Sleep Duration Is Associated With Testis Size in Healthy Young Men. J Clin Sleep Med **14**(10), 1757-1764 (2018). doi:10.5664/jcsm.7390

1240. Zhang, Z., Qiu, S., Huang, X., Jin, K., Zhou, X., Lin, T., Zou, X., Yang, Q., Yang, L., Wei, Q.: Association between testosterone and serum soluble α-klotho in U.S. males: a cross-sectional study. BMC Geriatr **22**(1), 570 (2022). doi:10.1186/s12877-022-03265-3

1241. Zhao, R., Li, Y., Dai, W.: Serum sex hormone and growth arrest-specific protein 6 levels in male patients with coronary heart disease. Asian J Androl **18**(4), 644-649 (2016). doi:10.4103/1008-682x.172825

1242. Zhao, S.P., Li, X.P.: The association of low plasma testosterone level with coronary artery disease in Chinese men. Int J Cardiol **63**(2), 161-164 (1998). doi:10.1016/s0167-5273(97)00295-7

1243. Zheng, H.Y., Li, Y., Dai, W., Wei, C.D., Sun, K.S., Tong, Y.Q.: Imbalance of testosterone/estradiol promotes male CHD development. Biomed Mater Eng **22**(1-3), 179-185 (2012). doi:10.3233/bme-2012-0705

1244. Zhou, N., Sun, L., Yang, H., Chen, Q., Wang, X., Yang, H., Tan, L., Chen, H., Zhang, G., Ling, X., Huang, L., Zou, P., Peng, K., Liu, T., Liu, J., Ao, L., Zhou, Z., Cui, Z., Cao, J.: Anogenital distance is associated with serum reproductive hormones, but not with semen quality in young men. Hum Reprod **31**(5), 958-967 (2016). doi:10.1093/humrep/dew052

1245. Zhou, Y.H., Guo, Y., Wang, F., Zhou, C.L., Tang, C.Y., Tang, H.N., Yan, D.W., Zhou, H.D., Chai, Z., Zhang, G., Ling, X., Dong, T., Wang, J., Zhang, Y., Zou, P., Yang, H., Zhou, N., Chen, Q., Zheng, Y., Liu, J., Cao, J., Ao, L.: Association of Sex Hormones and Fat Distribution in Men with Different Obese and Metabolic Statuses

Low-level and combined exposure to environmental metal elements affects male reproductive outcomes: Prospective MARHCS study in population of college students in Chongqing, China. Int J Gen Med **15**, 1225-1238 (2022). doi:10.2147/ijgm.S351282

10.1016/j.scitotenv.2022.154395

1246. Zhu, A., Andino, J., Daignault-Newton, S., Chopra, Z., Sarma, A., Dupree, J.M.: What Is a Normal Testosterone Level for Young Men? Rethinking the 300 ng/dL Cutoff for Testosterone Deficiency in Men 20-44 Years Old. J Urol **208**(6), 1295-1302 (2022). doi:10.1016/j.reprotox.2022.10.002

10.1097/ju.0000000000002928

1247. Zhu, H., Wang, N., Han, B., Li, Q., Chen, Y., Zhu, C., Chen, Y., Xia, F., Cang, Z., Lu, M., Chen, C., Lin, D., Lu, Y.: Low Sex Hormone-Binding Globulin Levels Associate with Prediabetes in Chinese Men Independent of Total Testosterone. PloS one **11**(9), e0162004 (2016). doi:10.1371/journal.pone.0162004

1248. Zhu, T.Y., Griffith, J.F., Qin, L., Hung, V.W., Fong, T.N., Au, S.K., Li, M., Lam, Y.Y., Wong, C.K., Kwok, A.W., Leung, P.C., Li, E.K., Tam, L.S.: Alterations of bone density, microstructure, and strength of the distal radius in male patients with rheumatoid arthritis: a case-control study with HR-pQCT. J Bone Miner Res **29**(9), 2118-2129 (2014). doi:10.1002/jbmr.2221

1249. Ziaeemehr, A., Sharebiani, H., Taheri, H., Fazeli, B., Tao, Z., Wang, Z., Zhu, S., Wang, S., Wang, Z.: Secondary Infertility: A Neglected Aspect of Buerger's Disease

Associations between benzophenone-3 and sex steroid hormones among United States adult men. Rep Biochem Mol Biol **11**(2), 246-251 (2022). doi:10.52547/rbmb.11.2.246

10.1016/j.reprotox.2022.10.002

1250. Ziaran, S., Goncalves, F.M., Breza, J., Sr.: Patients with prostate cancer treated by ADT have significantly higher fibrinogenemia than healthy control. World J Urol **31**(2), 289-292 (2013). doi:10.1007/s00345-012-0926-x

1251. Zitzmann, M., Brune, M., Nieschlag, E.: Vascular reactivity in hypogonadal men is reduced by androgen substitution. J Clin Endocrinol Metab **87**(11), 5030-5037 (2002). doi:10.1210/jc.2002-020504

1252. Zitzmann, M., Brune, M., Vieth, V., Nieschlag, E.: Monitoring bone density in hypogonadal men by quantitative phalangeal ultrasound. Bone **31**(3), 422-429 (2002). doi:10.1016/s8756-3282(02)00831-1

1253. Zmuda, J.M., Cauley, J.A., Kriska, A., Glynn, N.W., Gutai, J.P., Kuller, L.H.: Longitudinal relation between endogenous testosterone and cardiovascular disease risk factors in middle-aged men. A 13-year follow-up of former Multiple Risk Factor Intervention Trial participants. Am J Epidemiol **146**(8), 609-617 (1997). doi:10.1093/oxfordjournals.aje.a009326

1254. Zona, S., Guaraldi, G., Luzi, K., Beggi, M., Santi, D., Stentarelli, C., Madeo, B., Rochira, V.: Erectile dysfunction is more common in young to middle-aged HIV-infected men than in HIV-uninfected men. The journal of sexual medicine **9**(7), 1923-1930 (2012). doi:10.1111/j.1743-6109.2012.02750.x

1255. Zorbas, Y.G., Naexu, K.A., Federenko, Y.F.: Blood serum biochemical changes in physically conditioned and unconditioned subjects during bed rest and chronic hyperhydration. Clin Exp Pharmacol Physiol **19**(2), 137-145 (1992). doi:10.1111/j.1440-1681.1992.tb00432.x

1256. Zou, P., Wang, X., Sun, L., Chen, Q., Yang, H., Zhou, N., Chen, H., Zhang, G., Ling, X., Wang, Z., Gao, J., Mo, M., Huang, L., Peng, K., Chen, S., Cui, Z., Liu, J., Ao, L., Cao, J.: Semen Quality in Chinese College Students: Associations With Depression and Physical Activity in a Cross-Sectional Study. Psychosom Med **80**(6), 564-572 (2018). doi:10.1097/psy.0000000000000595
